# Optimal errors and phase transitions in high-dimensional generalized linear models

**DOI:** 10.1073/pnas.1802705116

**Published:** 2019-03-01

**Authors:** Jean Barbier, Florent Krzakala, Nicolas Macris, Léo Miolane, Lenka Zdeborová

**Affiliations:** ^a^Quantitative Life Sciences, International Center for Theoretical Physics, 34151 Trieste, Italy;; ^b^Laboratoire de Physique de l’Ecole Normale Supérieure, Université Paris-Sciences-et-Lettres, Centre National de la Recherche Scientifique, Sorbonne Université, Université Paris-Diderot, Sorbonne Paris Cité, 75005 Paris, France;; ^c^Communication Theory Laboratory, School of Computer and Communication Sciences, Ecole Polytechnique Fédérale de Lausanne, CH-1015 Lausanne, Switzerland;; ^d^Département d’Informatique de l’Ecole Normale Supérieure, Université Paris-Sciences-et-Lettres, Centre National de la Recherche Scientifique, Inria, 75005 Paris, France;; ^e^Institut de Physique Théorique, Centre National de la Recherche Scientifique et Commissariat à l’Energie Atomique, Université Paris-Saclay, 91191 Gif-sur-Yvette, France

**Keywords:** high-dimensional inference, generalized linear model, Bayesian inference, perceptron, approximate message-passing algorithm

## Abstract

Generalized linear models (GLMs) are used in high-dimensional machine learning, statistics, communications, and signal processing. In this paper we analyze GLMs when the data matrix is random, as relevant in problems such as compressed sensing, error-correcting codes, or benchmark models in neural networks. We evaluate the mutual information (or “free entropy”) from which we deduce the Bayes-optimal estimation and generalization errors. Our analysis applies to the high-dimensional limit where both the number of samples and the dimension are large and their ratio is fixed. Nonrigorous predictions for the optimal errors existed for special cases of GLMs, e.g., for the perceptron, in the field of statistical physics based on the so-called replica method. Our present paper rigorously establishes those decades-old conjectures and brings forward their algorithmic interpretation in terms of performance of the generalized approximate message-passing algorithm. Furthermore, we tightly characterize, for many learning problems, regions of parameters for which this algorithm achieves the optimal performance and locate the associated sharp phase transitions separating learnable and nonlearnable regions. We believe that this random version of GLMs can serve as a challenging benchmark for multipurpose algorithms.

## Significance

High-dimensional generalized linear models are basic building blocks of current data analysis tools including multilayers neural networks. They arise in signal processing, statistical inference, machine learning, communication theory, and other fields. We establish rigorously the intrinsic information-theoretic limitations of inference and learning for a class of randomly generated instances of generalized linear models, thus closing several decades-old conjectures. Moreover, we delimit regions of parameters for which the optimal error rates are efficiently achievable with currently known algorithms. Our proof technique is able to deal with the output nonlinearity and is hence of independent interest, opening ways to establish similar results for models of neural networks where nonlinearities are essential but in general difficult to account for.

As datasets grow larger and more complex, modern data analysis requires solving high-dimensional estimation problems with very many parameters. Developing algorithms for this task and understanding their limitations have become a major challenge in computer science, machine learning, statistics, signal processing, communications, and related fields.

In the present contribution, we address this challenge in the case of generalized linear estimation models (GLMs) (1, 2) where data are generated as follows: Given an n-dimensional vector ${\text{X}}^{*}$, hidden to statisticians, they observe instead an m-dimensional vector Y where each component reads

$$Y_{\mu }=\varphi \left(\frac{1}{\sqrt{n}}{\left[\Phi {\text{X}}^{*}\right]}_{\mu },A_{\mu }\right),\;1\leq \mu \leq m,$$ [1]

where Φ is an m×n “measurement” or “data” matrix, and the random variables $\left(A_{\mu }\right)\overset{iid}{∼}P_{A}$ account for noise/randomness of the model. The model is “linear” because the output $Y_{\mu }$ depends on a linear combination of the data $z_{\mu }=\frac{1}{\sqrt{n}}{\left[\Phi {\text{X}}^{*}\right]}_{\mu }=\frac{1}{\sqrt{n}}\sum_{i=1}^{n}\Phi_{\mu i}X_{i}^{*}$. The GLM generalizes the ordinary linear regression by allowing the output function φ(z,A) to be nonlinear and/or stochastic; in the case of a deterministic model we simply write φ(z). Explicit examples are given below.

GLMs belong to the realm of supervised learning and arise in a wide variety of scientific fields. In signal processing one usually observes $Y_{\mu }$ given as a linear combination of the signal elements ${\text{X}}^{*}$. In a range of applications these observations are obtained via a nonlinear function φ. In optics or X-ray crystallography one often measures only the amplitude of ${\left[\Phi {\text{X}}^{*}\right]}_{\mu }$, leading to the phase retrieval problem (3). A real-valued analog is the problem of sign retrieval when we observe only $\left|{\left[\Phi {\text{X}}^{*}\right]}_{\mu }\right|$ (4, 5). Observations are sometimes quantized to reduce the storage, leading for instance to the problem of 1-bit compressed sensing (6). In statistics and machine learning, classification is often described via a GLM where the output function φ is discrete and corresponds to the labels that classify the data points $\Phi_{\mu }$ (1, 2, 7). GLMs with nonlinear output functions are also the basic building blocks of each layer of neural networks (8): φ corresponds to the activation, the rows of the matrix Φ are different data samples, and ${\text{X}}^{*}$ is the set of synaptic weights to be learned.

There are two main learning problems in GLMs: (i) The estimation task requires, knowing the measured vector Y and the matrix Φ, inference of the unknown vector ${\text{X}}^{*}$; (*ii*) the prediction or generalization task instead requires, again knowing Y and Φ, accurate prediction of new values $Y_{new}$ when new rows (i.e., data points) are added to the matrix Φ.

In the present paper we build a rigorous theory for both of these tasks for random instances of the GLM. In this setting each element $\Phi_{\mu i}$ of the matrix is sampled independently from a probability distribution of zero mean and unit variance, and the unknown vector ${\text{X}}^{*}$ has been also created randomly from a probability distribution $P_{0}$, with each of its components $X_{1}^{*},\ldots ,X_{n}^{*}\overset{iid}{∼}P_{0}$. Since our main aim is to study the intrinsic information-theoretic and algorithmic limitations caused by the lack of samples and/or the amplitude of the noise, we assume throughout this paper that $P_{0}$ and φ are known to the statistician (if they are not, the task can only be harder). Our results are derived in the challenging and interesting high-dimensional limit where m,n→∞ and m/n→α a constant. Random instances of GLMs are both practically and theoretically relevant in many different contexts:

*i*) In signal processing, GLM estimation with a random matrix Φ has been studied with considerable attention in the context of compressed sensing (9–11), where an n-dimensional sparse signal is recovered from m<n noisy measurements. While standard compressed sensing focused on the linear case—where φ(z,A)=z+A with a Gaussian noise A—the generalized case was also widely studied (12, 13), especially for quantized output (14) and 1-bit compressed sensing (6, 15) where φ(z,A)=sign(z+A), as well as for compressive phase retrieval when φ(z,A)=|z+A| (16).
*ii*) In statistical learning, a substantial amount of activity is dedicated to understanding the limitation of learning with data generated by GLMs, both in the linear case, e.g., in the context of ridge regression or least absolute shrinkage and selection operator (LASSO) (17), or with nonlinear probabilistic output, e.g., logistic regression. Random instances were studied in particular in the context of so-called M estimators (18–21).
*iii*) In studies of artificial neural networks there has been a large amount of work using random instances of GLMs, with φ playing the role of a nonlinear activation function. In this context the random GLM was introduced as the teacher–student setting for the perceptron in the pioneering work of Gardner and Derrida (22). A large volume of work followed and is reviewed, e.g., in refs. 23–25. While initial works concentrated on a simple activation function φ(z)=sign(z−K) (K is the threshold constant), many other functions were considered, e.g., in refs. 26–28. Recently, the study of random instances of neural networks has emerged as a key ingredient in understanding the performance of deep-learning algorithms (29, 30). Computing mutual information in GLMs is also a critical issue in confirming the information bottleneck scenario of refs. 31 and 32.
*iv*) In communications, error-correcting codes that use random constructions are particularly efficient, as discussed by Shannon in his seminal paper (33). Random instances of GLMs describe both the setting of code-division multiple access—a multiuser access method used in communication technologies (34, 35)—and an error correction scheme called sparse superposition codes, which have been shown to achieve the Shannon capacity for any type of noisy channel (36–40).

Interestingly there is an important gap in the above volume of work. On the one hand there are studies that rely on the algorithmic performance of the so-called generalized approximate message-passing (GAMP) algorithm (11, 12, 41). GAMP is remarkable in that its asymptotic (n,m→∞, m/n→α) performance can be analyzed rigorously using the so-called state evolution (42–45). However, GAMP is not expected to be always information-theoretically optimal. On the other hand, other results are concerned with the linear case of the GLM with additive Gaussian noise for which the information-theoretically optimal performance was established in refs. 46–48 (the methodology of these works unfortunately does not generalize straightforwardly to the important nonlinear case or to other types of additive noise). All of the other works, which provide information-theoretic results for the nonlinear case, are based on powerful and sophisticated but nonrigorous techniques originating in statistical physics of disordered systems, such as the cavity and replica methods (49). Historically, the first of these nonrigorous, yet correct, results on information-theoretic limitations of learning was for the perceptron with binary weights and was established using the replica method in refs. 22, 50, and 51, including a discontinuous phase transition to perfect learning that appears as the ratio between the number of samples and the dimension exceeds α≈1.249.

In the present paper we close the above gap between mathematically rigorous work and conjectures (some of them several decades old) from statistical mechanics. In particular, we prove that the results for GLMs stemming from the replica method are indeed correct and imply the optimal value of both the estimation and generalization error. These results are summarized in *Main Results*. The proof is based on the adaptive interpolation method recently developed in ref. 52 and is of independent interest as it is applicable to a range of other models. We present it in *Methods and Proofs* and in *SI Appendix*. We compare our information-theoretic results to the performance of the GAMP algorithm and its state evolution (reviewed briefly in *Main Results*). We determine regions of parameters where this algorithm is or is not information-theoretically optimal. Up to technical assumptions (specified below), our results apply to all activation functions φ and priors $P_{0}$, thus unifying a large volume of previous work where many particular functions have been analyzed on a case-by-case basis. This generality allows us to provide a unifying understanding of the types of phase transitions and phase diagrams that we can encounter in GLMs, which is as well of independent interest and we devote *Application to Learning and Inference* to its presentation.

## Main Results

This section summarizes our main results. Their formal statement and all technical assumptions and full proofs are provided in *Methods and Proofs* and in *SI Appendix*.

For the random GLM problem as defined in the Introduction, the optimal way to estimate the ground-truth signal/weights ${\text{X}}^{*}$ relies on its posterior probability distribution

$$P\left(\text{x}|\text{Y},\Phi \right)=\frac{1}{Z\left(\text{Y},\Phi \right)}\overset{n}{\underset{i=1}{\prod }}P_{0}\left(x_{i}\right)\overset{m}{\underset{\mu =1}{\prod }}P_{out}\left(Y_{\mu }|\frac{{\left[\Phi \text{x}\right]}_{\mu }}{\sqrt{n}}\right),$$ [2]

where we used the prior $P_{0}$ of ${\text{X}}^{*}$ and introduced the likelihood $P_{out}$ that an output $Y_{\mu }$ is observed given $\frac{1}{\sqrt{n}}{\left[\Phi \text{x}\right]}_{\mu }$. $P_{out}\left(\cdot |z\right)$ is the probability density function of φ(z,A) [where again the random variable (r.v.) $A∼P_{A}$ accounts for noise]. We are concerned with the so-called Bayes-optimal setting where the prior $P_{0}$ and the likelihood $P_{out}$ that appear in the posterior **2** were also used to generate the ground-truth signal ${\text{X}}^{*}$ and the labels Y, with a known random matrix Φ.

A first quantity of interest is the free entropy (which is the free energy up to a sign) defined as $f_{n}\left(\text{Y},\Phi \right)\equiv \frac{1}{n}\ln Z\left(\text{Y},\Phi \right)$. The expectation of the free entropy is equal to minus the conditional entropy density of the observation $-\frac{1}{n}H\left(\text{Y}|\Phi \right)$, as well as (up to an additive constant) to the mutual information density between the signal and the observations $\frac{1}{n}I\left({\text{X}}^{*};\text{Y}|\Phi \right)$.

### The Free Entropy.

Our first result is the rigorous determination of the free entropy, in the high-dimensional asymptotic regime n,m→∞, m/n→α. For a random matrix Φ with independent entries of zero mean and unit variance, for output Y that was generated using **[1]**, and under appropriate technical assumptions stated precisely in *Methods and Proofs*, the free entropy converges in probability to

$$f_{n}\left(\text{Y},\Phi \right)\equiv \frac{1}{n}\ln Z\left(\text{Y},\Phi \right)\overset{P}{\underset{n\to \infty }{\to }}\underset{q\in \left[0,\rho \right]}{\sup}\underset{r\geq 0}{\inf}f_{RS}\left(q,r;\rho \right),$$ [3]

where $\rho \equiv E_{P_{0}}\left[{\left(X^{*}\right)}^{2}\right]$ and where the potential $f_{RS}\left(q,r;\rho \right)$ is

$$f_{\text{RS}}\left(q,r;\rho \right)\equiv \psi_{P_{0}}\left(r\right)+\alpha \Psi_{P_{\text{out}}}\left(q;\rho \right)-rq/2,$$ [4]

$$\text{with}\psi_{P_{0}}\left(r\right)\equiv \underset{\left[Z_{0},X_{0}\right]}{E}\ln\int dP_{0}\left(x\right)e^{rxX_{0}+\sqrt{r}xZ_{0}-rx^{2}/2},$$ [5]

$$\Psi_{P_{\text{out}}}\left(q;\rho \right)\equiv \underset{\left[V,W,{\tilde{Y}}_{0}\right]}{E}\ln\int DwP_{\text{out}}\left({\tilde{Y}}_{0}|\sqrt{q}V+\sqrt{\rho -q}w\right),$$ [6]

where $Dw=dw\exp\left(-w^{2}/2\right)/\sqrt{2\pi }$ is a standard Gaussian measure and the scalar r.v. are independently sampled from $X_{0}∼P_{0}$, then $V,W,Z_{0}\overset{iid}{∼}N\left(0,1\right)$ and ${\tilde{Y}}_{0}∼P_{out}\left(\cdot |\sqrt{q}V+\sqrt{\rho -q}W\right)$. Only the special linear case with Gaussian $P_{out}$ is known rigorously so far (46–48). Convergence of the averaged free entropy is precisely stated in *Theorem 1*; the one in probability follows from concentration results in *SI Appendix*.

One can check by explicit comparison that for specific choices of $P_{0}$ and $P_{out}$ the expression **4** is the replica-symmetric free entropy derived in numerous statistical physics papers (thus the RS in $f_{RS}$) and in particular in refs. 22, 41, 50, and 51 for φ(z)=sign(z). The formula for general $P_{0}$ and $P_{out}$ was conjectured based on the statistical physics derivation in ref. 13. Establishing [**3**] closes these old conjectures and yields an important step toward vindication of the cavity and replica methods for inference, along with, e.g., refs. 43 and 53. We now discuss the main consequences of this formula.

### Overlap and Optimal Estimation Error.

Our second result concerns the overlap between a sample x from the posterior **2** and the ground truth. We obtain that as n,m→∞, n/m→α,

$$\frac{1}{n}|\text{x}\cdot {\text{X}}^{*}|\overset{P}{\underset{n\to \infty }{\to }}q^{*}$$ [7]

whenever $q^{*}=q^{*}\left(\alpha \right)$ the maximizer in formula **3** is unique. This is the case for almost every α (*SI Appendix*).

It is a simple fact of Bayesian inference that, given the measurements Y and the measurement matrix Φ, the estimator $\hat{\text{X}}$ that minimizes the mean-square error with the ground-truth ${\text{X}}^{*}$ is the mean of the posterior distribution **2**; i.e., $\hat{\text{X}}=E_{P\left(\text{x}|\text{Y},\Phi \right)}\left[\text{x}\right]$. The minimum mean-square error (MMSE) that is achieved by such a “Bayes-optimal” estimator is deduced, again in the limit n→∞,m/n→α, as follows:

$$\text{MMSE}=\frac{1}{n}E\left[\|{\text{X}}^{*}-\hat{\text{X}}{\|}^{2}\right]\to \rho -q^{*}.$$ [8]

We refer to *Theorem 2* in *Methods and Proofs* for rigorous statements. Again the value of the MMSE is known rigorously so far only for the linear case with Gaussian noise (46–48) (and conjectured for the nonlinear case, e.g., in ref. 13).

### Optimal Generalization Error.

Our third result concerns the prediction error, also called generalization error. Consider again the statistical model **1**. To define the Bayes-optimal generalization error, one is given a new row of the matrix/data point, denoted $\Phi_{new}\in R^{n}$ (in addition to the data Φ and associated outputs Y used for the learning), and is asked to estimate the corresponding output value $Y_{new}$. We seek for an estimator ${Ŷ}_{new}={Ŷ}_{new}\left(\text{Y},\Phi ,\Phi_{new}\right)$ that achieves $E_{gen}\equiv \min_{{Ŷ}_{new}}E\left[{\left(Y_{new}-{Ŷ}_{new}\right)}^{2}\right]$, i.e., that minimizes the MSE with the true $Y_{new}$ obtained using the ground-truth weights ${\text{X}}^{*}$. Such an estimator is again obtained from the posterior: ${Ŷ}_{new}=E_{P_{A}\left(a\right)}E_{P\left(\text{x}|\text{Y},\Phi \right)}\varphi \left(\frac{1}{\sqrt{n}}\Phi_{new}\cdot \text{x},a\right)$. Note that this is different from the plug-in estimator ${\tilde{Y}}_{new}=\varphi \left(\frac{1}{\sqrt{n}}\Phi_{new}\cdot \hat{\text{X}}\right)$, which leads to a worse MSE than ${Ŷ}_{new}$. Yet it is often used in practice for deterministic models since most algorithms for generalized linear regression do not provide the full posterior distribution.

Our result states that the optimal generalization error follows from the I-MMSE theorem (54) applied to the free entropy **3** (see *SI Appendix* for details). The optimal generalization error reads as n→∞, m/n→α ($q^{*}$ is the maximizer in **[3]**),

$$E_{gen}\to \underset{V,a}{E}\left[\varphi {\left(\sqrt{\rho }V,a\right)}^{2}\right]-\underset{V}{E}\left[\;\underset{w,a}{E}{\left[\varphi \left(\sqrt{q^{*}}V+\sqrt{\rho -q^{*}}w,a\right)\right]}^{2}\right],$$ [9]

where $V,w\overset{iid}{∼}N\left(0,1\right)$ and $a∼P_{A}$. See again *Theorem 2* in *Methods and Proofs* for the precise statement (and *SI Appendix*, *Theorems 3* and *4*).

Note that for labels Y belonging to a discrete set the MSE might not be a suitable loss and we are more often interested in maximizing the so-called overlap, i.e., the probability of obtaining the correct label. In this case the Bayes-optimal estimator is computed as the argmax of the posterior marginals, rather than as their mean; i.e., for discrete labels ${Ȳ}_{new}={argmax}_{y}P\left(y=\varphi \left(\frac{1}{\sqrt{n}}\Phi_{new}\cdot \text{x},a\right)\right)$ where again x is distributed according to **[2]**, $a∼P_{A}$. The replica method has been used to compute the optimal generalization error for the perceptron where φ(x)=sign(z) in the pioneering works of refs. 23, 50, and 55. We note that in this special case the plug-in estimator ${\tilde{Y}}_{new}$ is actually equal to the optimal one ${Ȳ}_{new}$.

A final note concerns the issue of overfitting. In optimization-based approaches to learning overfitting may lead to a generalization error which is too large compared with the training error. In the Bayes-optimal setting the estimators are constructed to not overfit. This is related to general properties of Bayes-optimal inference and learning that are called “Nishimori conditions” in the physics literature (13) and that turn out to be crucial in our proofs.

### Optimality of Approximate Message Passing.

Although the three results stated above are of an information-theoretic nature, our fourth one concerns the performance of an algorithm for solving random instances of GLMs called GAMP (11–13), which is closely related to the Thouless–Anderson–Palmer (TAP) equations developed in statistical physics (41, 56, 57).

The GAMP algorithm can be summarized as follows (11–13): Given initial estimates ${\hat{\text{x}}}^{0},v^{0}$ for the marginal posterior means and variances of the unknown signal vector ${\text{X}}^{*}$ entries, GAMP iterates the following equations, with $g_{\mu }^{0}=0$:

$$\left\{\begin{array}{ccc} V^{t} & =\bar{v^{t-1}}\\ \omega^{t} & =\Phi {\hat{\text{x}}}^{t-1}/\sqrt{n}-V^{t}g^{t-1}\\ g_{\mu }^{t} & =g_{P_{out}}\left(Y_{\mu },\omega_{\mu }^{t},V^{t}\right) & \forall \mu =1,\ldots m\\ \lambda^{t} & =\alpha \bar{g_{P_{out}}^{2}\left(\text{Y},\omega^{t},V^{t}\right)}\\ R^{t} & ={\hat{\text{x}}}^{t-1}+{\left(\lambda^{t}\right)}^{-1}\Phi^{⊺}{\text{g}}^{t}/\sqrt{n}\\ {\hat{x}}_{i}^{t} & =g_{P_{0}}\left(R_{i}^{t},\lambda^{t}\right) & \forall i=1,\ldots n\\ v_{i}^{t} & ={\left(\lambda^{t}\right)}^{-1}\partial_{R}g_{P_{0}}\left(R,\lambda^{t}\right){|}_{R=R_{i}^{t}} & \forall i=1,\ldots n \end{array}\right.$$

(here we denote by $\bar{\text{u}}$ the average over all of the components of a vector u). The so-called thresholding function $g_{P_{0}}\left(R,\lambda \right)$ is defined as the mean of the normalized distribution $\propto P_{0}\left(x\right)\exp\left(-\lambda {\left(R-x\right)}^{2}/2\right)$ and the output function $g_{P_{out}}\left(Y,\omega ,V\right)$ is similarly the mean of the normalized distribution (of x) $\propto P_{out}\left(Y|\omega +\sqrt{V}x\right)\exp\left(-x^{2}/2\right)$.

The heuristic derivation of GAMP in statistical physics (13) suggests via the definition of the function $g_{P_{out}}$ that ω and V are the estimates of the means and average variance of the components of the variable z=Φx. This, in turn, suggests a GAMP prediction of labels of new data points,

$${Ŷ}_{new}^{GAMP,t}=\int yP_{out}\left(y|\omega_{new}^{t}+z\sqrt{V^{t}}\right)dyDz,$$

where $\omega_{new}^{t}\equiv \frac{1}{\sqrt{n}}\Phi_{new}\cdot {\hat{x}}^{t-1}$. Comparing it with the test-set labels, this serves to compute GAMP’s generalization error.

One of the strongest assets of GAMP is that its performance can be tracked via a closed-form procedure known as state evolution (SE), again in the asymptotic limit when n,m→∞, m/n→α. For proofs of SE see refs. 43 and 44 for the linear case and ref. 45 for the generalized one. In our notations, SE tracks the correlation (or “overlap”) between the true weights ${\text{X}}^{*}$ and their estimate ${\hat{x}}^{t}$ defined as $q^{t}\equiv \underset{n\to \infty }{\text{lim}}\frac{1}{n}X^{*}\cdot {\hat{x}}^{t}$ via

$$q^{t}=2\psi_{P_{0}}^{\prime }\left(r^{t}\right),\;r^{t}=2\alpha \Psi_{P_{\text{out}}}^{\prime }\left(q^{t-1};\rho \right).$$ [10]

The derivatives are with respect to (w.r.t.) the first argument. Similarly for the evolution of GAMP’s generalization error $E_{gen}^{GAMP,t}$ (*SI Appendix*) we obtain that it is asymptotically, and with high probability, given by the right-hand side (r.h.s.) of formula **9** but with $q^{*}$ replaced by $q^{t}$.

It is a simple algebraic fact that the fixed points of the SE Eqs. **10** correspond to the critical points of the potential **4**. The question of GAMP achieving asymptotically optimal MMSE or generalization error therefore reduces to the study of the extrema of the two-scalar-variables potential **4**. If the SE **10** converges to the same couple (q,r) as the extremizer $\left(q^{*},r^{*}\right)$ of **[3]**, then GAMP is optimal, and if it does not, then GAMP is suboptimal. In the next section we illustrate this result on several examples, delimiting regions where GAMP reaches optimality. We note that optimality of AMP-based algorithms in terms of the MMSE on the ground-truth vector ${\text{X}}^{*}$ was proved for several cases where the extremizer $q^{*}$ in **[3]** is unique, e.g., ref. 58, or in the linear case of GLM in ref. 47. Our results allow us to complete the characterization of regions of parameters where the algorithm reaches optimal performance in terms of the estimation and generalization errors. While the asymptotic value of the Bayes-optimal generalization error was predicted for some cases of $P_{out}$ and $P_{0}$ (55), and TAP-based algorithms were argued to reach this performance in refs. 59 and 60, it was not known whether this error can be achieved provably or for what exact regions of parameters the algorithm is suboptimal. Our present work settles this question due to the state evolution of the GAMP algorithm. Interestingly, heuristic arguments based on the glassy nature of the corresponding probability measure were used to argue that direct sampling or optimization-based approaches will not be able to match this performance (51). Whether this statement is correct goes beyond the scope of the present paper.

## Application to Learning and Inference

In this section, we report what our results imply for the information-theoretically optimal errors and those reached by the GAMP algorithm for several interesting cases of output functions φ and prior distributions $P_{0}$. We do not seek to be exhaustive in any way; we simply aim to illustrate the kind of insights about the GLM that can be obtained from our results. We focus on determination of phase transitions in performance as we vary parameters of the model, e.g., the number of samples or the sparsity of the signal. We use careful numerical procedures to compute the expectations required in formula **4** and check that the reported results are stable toward the choice of various precision parameters. In this section we, however, do not seek rigor in bounding formally the corresponding numerical errors. Many of the codes used in this section are given online in a github repository (62).

### General Observations About Fixed Points and Terminology.

#### Noninformative fixed point and its stability.

It is instrumental to analyze under what conditions $q^{*}=0$ is the optimizer in **[3]**. Our result **8** about the MMSE implies that if $q^{*}=0$, then the MMSE is as large as if we had no samples/measurements at our disposition. A necessary condition for $q^{*}=0$ is that it is a fixed point of the state evolution. In turn, a sufficient condition for the state evolution **10** to have such a fixed point is that (i) the output density $P_{out}\left(y|z\right)$ is even in the argument z and that (ii) the prior $P_{0}$ has zero mean. A proof of this is given in *SI Appendix*. For $q^{*}=0$ to be a fixed point to which the state evolution **10** converges, it needs to be stable. We detail in *SI Appendix* that under properties i and ii this fixed point is stable when

$$\alpha \int dy\frac{{\left(\int Dz\left(z^{2}-1\right)P_{out}\left(y|\sqrt{\rho }z\right)\right)}^{2}}{\int DzP_{out}\left(y|\sqrt{\rho }z\right)}<1.$$ [11]

In what follows we denote $\alpha_{c}$ the largest value of α for which the above condition holds. Consequently the error reachable by the GAMP algorithm is as bad as random guessing for both the estimation and generalization errors as long as $\alpha <\alpha_{c}$. For $\alpha >\alpha_{c}$, starting with infinitesimal positive q the state evolution will move toward larger q as in ref. 63. Note that condition **11** also appears in a recent work (61) as a barrier for performance of spectral algorithms.

Concerning the information-theoretically optimal error, we call the phase where MMSE=ρ, i.e., $q^{*}=0$ is the extremizer of **[4]**, the noninformative phase. Existing literature sometimes refers to such behavior as the retarded learning phase (64), in the sense that in this case a critical number of samples is required for the generalization error to be better than random guessing. Below we evaluate condition **11** explicitly for several examples.

#### Almost exact recovery fixed point.

Another fixed point of **[10]** that is worth our particular attention is the one corresponding to almost exact recovery, meaning with average error per coordinate going to 0 as n→∞, where $q^{*}=\rho$. A sufficient and necessary condition for this to be a fixed point is that $\underset{q\to \rho }{\text{lim}}\Psi_{P_{out}}^{\prime }\left(q;\rho \right)=+\infty$. This means that the integral of the Fisher information of the output channel diverges,

$$\int dyd\omega \frac{e^{-\frac{\omega^{2}}{2\rho }}}{\sqrt{2\pi \rho }}\frac{P_{out}^{\prime }{\left(y|\omega \right)}^{2}}{P_{out}\left(y|\omega \right)}=+\infty ,$$

where $P_{out}^{\prime }\left(y|\omega \right)$ denotes the partial derivative w.r.t. ω. This typically means that the output channel should be noiseless. For example, for the Gaussian channel with noise variance Δ, the above expression equals 1/Δ. For the probit channel where $P_{out}\left(y|z\right)=erfc\left(-yz/\sqrt{2\Delta }\right)/2$ the above expression at small Δ is proportional to $1/\sqrt{\Delta }$.

Stability of the almost exact recovery fixed point depends nontrivially on the properties of both the output channel and the prior. Below we give several examples where almost exact recovery either is or is not possible. In what follows we call the region of parameters for which MMSE=0, i.e., $q^{*}=\rho$ is the extremizer in **[3]**, the almost exact recovery phase.

#### Hard phase.

As can be anticipated from the statement of our main algorithmic result, there are regions of parameters for which the error reached by GAMP is asymptotically equal to the optimal error and regions where it is not. We call the hard phase the region of parameters where $MMSE<{MSE}_{AMP}$ with a strict inequality. Focusing on the ratio α between the number of samples and the dimensionality, we denote $\alpha_{IT}$ the ratio for which the hard phase appears and $\alpha_{AMP}>\alpha_{IT}$ the ratio for which it disappears. In other words, the hard phase is an interval $\left(\alpha_{IT},\alpha_{AMP}\right)$ and is associated to a first-order phase transition in the Bayes-optimal posterior probability distribution.

It remains a formidable open question of average computational complexity whether in the setting of this paper (and for problems that are NP complete in the worst case) there exists an efficient algorithm that achieves better performance than GAMP in the hard phase. We are not aware of any and tend to conjecture that there is not.

### Sensing Compressively with Nonlinear Outputs.

**Fig. 1.**
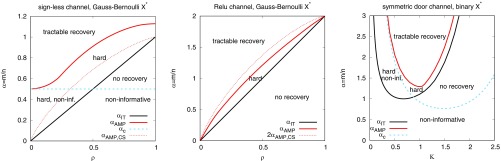
Phase diagrams showing boundaries of the region where almost exact recovery is possible (in absence of noise). (*Left*) The case of signless sparse recovery, φ(x)=|x| with a Gauss–Bernoulli signal, as a function of the ratio between number of samples/measurements and the dimension α=m/n, and the fraction of nonzero components ρ. Evaluating **[4]** for this case, we find that a recovery of the signal is information-theoretically impossible for $\alpha <\alpha_{IT}=\rho$. Recovery becomes possible starting from α>ρ, just as in the canonical compressed sensing. Algorithmically the signless case is much harder. Evaluating **[11]**, we conclude that GAMP is not able to perform better than a random guess as long as $\alpha <\alpha_{c}=1/2$, and the same is true for spectral algorithms (61). For larger values of α, the inference using GAMP leads to better results than a purely random guess. GAMP can recover the signal and generalize perfectly only for values of α larger than $\alpha_{AMP}$ (solid red line). The dotted red line shows for comparison the algorithmic phase transition of the canonical compressed sensing. (*Center*) Analogous to *Left*, for the ReLU output function, φ(x)=max(0,x). Here it is always possible to perform better than random guessing using GAMP. The dotted red line shows the algorithmic phase transition when using information only about the nonzero observations. (*Right*) Phase diagram for the symmetric door output function φ(z)=sign(|z|−K) for a Rademacher signal, as a function of α and K. The stability line $\alpha_{c}$ is depicted as a dashed blue line, the information-theoretic phase transition to almost exact recovery $\alpha_{IT}$ is a solid black line, and the algorithmic one $\alpha_{AMP}$ is a solid red line.

Existing literature covers in detail the case of noiseless compressed sensing, i.e., when the output function φ(z)=z. The representative sparse prior distribution is the Gauss–Bernoulli (GB) distribution $P_{0}=\rho N\left(0,1\right)+\left(1-\rho \right)\delta_{0}$, where ρ is the average fraction of nonzeros, which are in this case standard Gaussians. The phase diagram of this case is well known (67, 68). In noiseless compressed sensing with random i.i.d. matrices and GB prior, almost exact recovery of the signal is possible for $\alpha >\alpha_{IT}=\rho$ and GAMP recovers the signal for $\alpha >\alpha_{AMP,CS}$ where $\alpha_{AMP,CS}$ is plotted in Fig. 1 (*Left*) with a dotted red line, thus delimiting the hard phase of compressed sensing. We note that the Donoho–Tanner phase transition (9) known as the performance limit of the LASSO $\ell_{1}$ regularization is slightly higher than $\alpha_{AMP,CS}$.

#### Signless output channel.

The phase diagram of noiseless compressed sensing changes intriguingly when only the absolute value of the output is measured, i.e., when φ(z)=|z| instead of φ(z)=z. Such an output channel is reminiscent of the widely studied phase retrieval problem (3) where the signal is complex valued and only the amplitude is observed. The generalization of our results for the complex case would require extensions, as done for the algorithmic aspects in ref. 69. The real-valued case was studied under the name “sparse recovery from quadratic measurements” in the literature, e.g., ref. 70 and references therein, when the number of nonzero variables grows slower than linearly with the dimension n. Our results give access to the phase diagram of sparse recovery from quadratic (or equivalently signless) measurements that is presented in Fig. 1 (*Left*) for the GB prior.

We observe that the information-theoretical phase transition $\alpha_{IT}$ is the same in the signless sparse recovery as in the canonical linear case; i.e., almost exact recovery is possible whenever α>ρ. However, the algorithmic phase transition $\alpha_{AMP}$ above which GAMP is able to find the sparse signal is strikingly larger for the signless case (solid red line in Fig. 1, *Left*). (We note that to break the symmetry that prevents GAMP from finding the signal in a constant number of iteration steps, we mismatch infinitesimally the output function φ used in the algorithm from the symmetric one used to generate the data. Another way to deal with this issue is related to a spectral initialization as discussed recently in ref. 61.) We note that even for a dense signal ρ=1 almost exact recovery is algorithmically possible only for $\alpha >\alpha_{AMP}\left(\rho =1\right)\approx 1.128$. For very sparse signals, small ρ, the situation is even more striking because the measurement rate of at least $\alpha >\alpha_{c}=1/2$ is needed for algorithmically tractable almost exact recovery for every ρ. This is in sharp contrast with the canonical compressed sensing where $\alpha_{AMP,CS}\to 0$ as ρ→0. The nature of this algorithmic difficulty of GAMP is related to the symmetry of the output channel due to which the noninformative fixed point is stable for $\alpha <\alpha_{c}=1/2$. Summarizing this result in one sentence, tractable compressive sensing is impossible (for α<1/2) if we have lost the signs. We reiterate that this result holds in the setting of the present paper, i.e., in particular when the sparsity ρ is of constant order. For signals where ρ=O(1) the situation is expected to be different (70).

#### Rectified linear unit output channel.

Another case of output channel that attracted our interest is the rectified linear unit (ReLU), φ(z)=max(0,z), as widely used in multilayer feedforward neural networks. In the present single-layer case reconstruction with the ReLU output is interesting mathematically. With GB signals, roughly half of the measurements are given without noise, but the only information we have about the other half is its sign. A straightforward upper bound for both information-theoretic and tractable almost exact recovery is simply twice as many measurements than needed in the canonical noiseless compressed sensing. It is interesting to ask whether this bound is tight. Results in the present paper imply that for the information-theoretic performance this bound indeed is tight. However, the phase transition $\alpha_{AMP}$ above which almost exact recovery is possible with the GAMP algorithm is strictly lower than twice the phase transition of compressed sensing; both are depicted in Fig. 1, *Center*. This implies that while the negative outputs are not useful information theoretically, they do help to achieve better performance algorithmically.

### Perceptron and Similar Problems.

#### Binary and Gauss–Bernoulli perceptron.

**Fig. 2.**
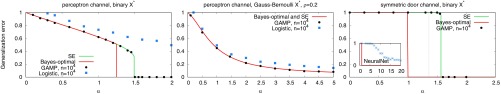
Optimal generalization error in three classification problems vs. the sample complexity α, the size of the training set being αn. The solid red line is the Bayes-optimal generalization error **9** while the solid green line shows the (asymptotic) performances of GAMP as predicted by the state evolution **10**. For comparison, we also show the results of GAMP (black circles) and the performance of a standard out-of-the-box solver (blue squares). (*Left*) Perceptron, with φ(x)=sign(x) and a binary Rademacher signal. While a perfect generalization is information-theoretically possible starting from $\alpha_{IT}\approx 1.249$, the state evolution predicts that GAMP will achieve such perfect prediction only above $\alpha_{AMP}\approx 1.493$. The results of a logistic regression with fine-tuned regularizations with the software scikit-learn (65) are shown for comparison. (*Center*) Perceptron with Gauss–Bernoulli distribution of the weights. No phase transition is observed in this case, but a smooth decrease of the error with α. The results of a logistic regression are very close to optimal. (*Right*) The symmetric door activation rule with parameter K chosen to observe the same number of occurrences of the two classes. In this case there is a sharp phase transition from as bad as random to perfect generalization at $\alpha_{IT}=1$. GAMP identifies the rule perfectly, starting only from $\alpha_{AMP}\approx 1.566$. The noninformative fixed point is stable up to $\alpha_{c}=1.36$ (dashed gray line). Interestingly, this nonlinear rule seems very hard to learn for standardly used solvers. Using Keras (66), a neural network with two hidden layers was able to learn only approximately the rule, only for considerably larger training set sizes and a much larger number of iterations than GAMP.

One of the most studied problems that fits in the setting of the present paper is the problem of the perceptron (71), where φ(z)=sign(z), that has been analyzed for random patterns Φ in the statistical physics literature; see refs. 23–25 for reviews. We plot in Fig. 2 the optimal generalization error **9** as follows from our results for the binary perceptron, i.e., weights taken from the Rademacher distribution $P_{0}=\frac{1}{2}\delta_{+1}+\frac{1}{2}\delta_{-1}$ (Fig. 2, *Left*) and for the GB perceptron where $P_{0}=\rho N\left(0,1\right)+\left(1-\rho \right)\delta_{0}$ (Fig. 2, *Center*). The information-theoretically optimal value of the generalization error that we report and prove agrees with existing predictions obtained by the nonrigorous replica method from refs. 50, 51, and 55. Notably, we see that for the GB case the optimal generalization error decreases smoothly as α increases, while for the binary case the generalization error has a first-order (i.e., discontinuous) phase transition toward perfect generalization at $\alpha_{IT}\approx 1.249$ as predicted already in ref. 50. Our results provide rigorous validation for these old conjectures.

Furthermore, our results together with recent literature on GAMP provide a refreshing clarification of the algorithmic questions. It is natural to ask for what region of parameters the optimal generalization error can be provably achieved with efficient algorithms. This question remained unanswered until now. Indeed, for the spherical perceptron the optimal generalization error was computed in ref. 55 and argued empirically in small instances to be achievable with a TAP-like algorithm (59). The state evolution of GAMP together with our formulas for the generalization error (**[9]** for the average optimal one and with $q^{t}$ replacing $q^{*}$ in this formula for GAMP) imply that the optimal generalization error is indeed achievable asymptotically for all α in the GB perceptron.

For the binary perceptron the optimal generalization error was computed in refs. 50 and 51. By comparing with the state evolution of GAMP we obtain that it can also be asymptotically achieved by GAMP, but this time only outside of the hard phase $\left(\alpha_{IT},\alpha_{AMP}\right)$ with $\alpha_{AMP}\approx 1.493$. The literature was unclear on the algorithmic question; ref. 50 identified the spinodal of the replica-symmetric solution to be at α≈1.493, but did not attribute it to any algorithmic or physical meaning. Ref. 51 argues that metastable states exist at least up to $\alpha_{RSB}\approx 1.628$ and speculates that Gibbs sampling-based algorithms will not be able to reach perfect generalization before that point (23). Taking our results into account, the main algorithmic question that remains open is whether efficient algorithms can reach perfect generalization for $\alpha_{IT}<\alpha <\alpha_{AMP}$.

#### Symmetric door.

Out of interest we explored an example of a binary output channel for which $P_{out}\left(y|z\right)$ is even in the argument z, so that the noninformative fixed point $q^{*}=0$ exists. Specifically we analyzed the symmetric door channel with φ(z)=sign(|z|−K) and Rademacher prior $P_{0}$. In literature such a perceptron is studied with the replica method in the context of lossy data compression (28). In Fig. 1, *Right* we report the phase diagram in terms of the stability line of the noninformative fixed point $\alpha_{c}$ (below which GAMP is not better than random guesses), the information-theoretic phase transition toward perfect generalization $\alpha_{IT}$, and the phase transition of GAMP to perfect generalization $\alpha_{AMP}$.

A simple-counting lower bound states that for binary outputs and weights $X_{i}^{*}$ perfect generalization is not possible for α<1. Thus it is interesting to note that the symmetric door channel is able to saturate this lower bound for K≈0.6745 for which the probability of $y_{\mu }=1$ is 1/2. This saturation was already stated in ref. 28. Our results also, however, imply that in that case the perfect generalization will not be achievable with the GAMP (and we conjecture no other efficient) algorithm unless $\alpha >\alpha_{AMP}\approx 1.566$. The generalization error that GAMP provides for this case is depicted in Fig. 2, *Right*.

### Empirical comparison with general-purpose algorithms.

In this section we argue that many cases that fit into the setting of the present paper could serve as useful benchmarks for existing machine-learning algorithms. We believe that the situation is perhaps similar to Shannon coding theorems that have driven algorithmic developments in error-correcting codes, achieving the Shannon bound being the primary goal in many works in communications. In machine learning, classification is a natural task and algorithms are usually benchmarked using open access databases. In current state-of-the-art applications of machine learning we usually have very little insight about what is the sample complexity, i.e., how many samples are truly needed so that a given generalization error can be achieved. In our setting the situation is different: We can present samples $\left(y_{\mu },\Phi_{\mu }\right)$ to generic out-of-the-box classification algorithms and see how their performances compare with the information-theoretic optimal performance and to the one of the GAMP algorithm that is fine-tuned to the problem.

In Fig. 2 we present examples of state-of-the-art classification algorithms that are compared with our results. In Fig. 2, *Left* and *Center* we compare the optimal and GAMP performances to a simple logistic regression, fine-tuned by manually optimizing the ridge penalty (for $\ell_{2}$ regularization) and LASSO penalty (for a sparsity-enhancing $\ell_{1}$ regularization) with the software scikit-learn (65). We observe that for the GB case the logistic regression is comparable to the performance of GAMP, whereas for binary weights perfect generalization is not achieved close to the GAMP phase transition.

In Fig. 2, *Right* we study classification for labels generated by the symmetric door channel. A general-purpose algorithm would not know about the form of the channel. A neural network with only two hidden units is in principle able to represent the corresponding function (each of the hidden neurons can learn one of the two planes that separate data in the symmetric door function). A more intriguing question is whether a more generic multilayer neural network is indeed able to learn this rule and how many samples it may need. In the example used in Fig. 2, using the software Keras (66) with a tensorflow backend, we show the performance of a network with two hidden layers, ReLU activation and dropout [the details for this particular run can be found in the github repository (62)]. The symmetric door function thus provides a challenging benchmark that could be used to study how to improve performance of the general-purpose multilayer neural network classifiers. In *SI Appendix* we provide additional examples comparing the optimal performance to general-purpose algorithms for regression.

## Methods and Proofs

In this section we give the main theorem for the free entropy and main ideas of the proof. An essential tool is the adaptive interpolation method recently introduced in ref. 52 which is a powerful evolution of the Guerra and Toninelli (72) interpolation method developed for spin glasses. Ref. 52 analyzed simpler inference problems. In particular, the proof for the upper bound in ref. 52 does not apply to GLMs and requires nontrivial additional ingredients. One such additional ingredient is to work with a potential $f_{RS}\left(q,r;\rho \right)$ depending on two parameters (q,r) instead of a single one as in ref. 52. This allows us to use convexity arguments that are crucial to finish the proof, discussed in *Matching Bounds and End of Proof*. We stress that the present analysis heavily relies on properties of Bayes-optimal inference that translate into remarkable identities between correlation functions (called Nishimori identities by physicists; see *SI Appendix* for their formulation) valid for all values of parameters. These identities are used in the derivation of **[17]** and **[18]** below, which are two essential steps of our proof. The formula from *Theorem 1* relies on the Nishimori identities and does not hold out of the Bayes-optimal setting.

### Main Theorems.

For the proof it is necessary to work with a slightly different model with an additive regularizing Gaussian noise with variance Δ≥0,

$$Y_{\mu }=\varphi \left(\frac{1}{\sqrt{n}}{\left[\Phi \text{X}*\right]}_{\mu },A_{\mu }\right)+\sqrt{\Delta }Z_{\mu },\;1\leq \mu \leq m,$$ [12]

where $\left(Z_{\mu }\right)\overset{iid}{∼}N\left(0,1\right)$, and $\left(A_{\mu }\right)\overset{iid}{∼}P_{A}$ are r.v. that represent the stochastic part of φ. It is also instrumental to think of the measurements as the outputs of a “channel” $Y_{\mu }∼P_{out}\left(\cdot |\frac{1}{\sqrt{n}}{\left[\Phi \text{X}*\right]}_{\mu }\right)$ with transition density $P_{out}\left(y|z\right)={\left(2\pi \Delta \right)}^{-1/2}\int dP_{A}\left(a\right)\exp\left\{-\frac{1}{2\Delta }{\left(y-\varphi \left(z,a\right)\right)}^{2}\right\}$ if Δ>0, or $P_{out}\left(y|z\right)=\int dP_{A}\left(a\right)1\left(y=\varphi \left(z,a\right)\right)$ else, where 1(⋅) is the indicator function. Our main theorem holds under the following rather general hypotheses:

h1) The prior distribution $P_{0}$ admits a finite third moment and has at least two points in its support.
h2) The sequence ${\left(E\left[{\left|\varphi \left(\frac{1}{\sqrt{n}}{\left[\Phi \text{X}*\right]}_{1},{\text{A}}_{1}\right)\right|}^{2+\gamma }\right]\right)}_{n\geq 1}$ is bounded for some γ>0.
h3) The r.v. $\left(\Phi_{\mu i}\right)$ are independent with zero mean, unit variance, and finite third moment bounded with n.
H4) For almost all values of a (w.r.t. the distribution $P_{A}$), the function x↦φ(x,a) is continuous almost everywhere.
h5) (Δ>0) or (Δ=0 and φ takes values in N).

In general, when φ is continuous, the condition Δ>0 (but arbitrarily small) is necessary for the existence of a finite limit of the free entropy [for particular choices of $\left(\varphi ,P_{A}\right)$ this might not be needed, e.g., φ(z,A)=z+A with $A∼N\left(0,\sigma^{2}\right)$]. We also assume that the kernel $P_{out}$ is informative; i.e., there exists y such that $P_{out}\left(y|\cdot \right)$ is not equal almost everywhere to a constant. If $P_{out}$ is not informative, it is not difficult to show that estimation is then impossible.

We define the set of the critical points of $f_{RS}$, **[4]**, also called “state evolution fixed points” (as is clear from **[10]**):

$$\Gamma \equiv \left\{\left(q,r\right)\in \left[0,\rho \right]\times \left(R_{+}\cup \left\{+\infty \right\}\right)|\begin{array}{cc} q & =2\psi_{P_{0}}^{\prime }\left(r\right)\\ r & =2\alpha \Psi_{P_{out}}^{\prime }\left(q;\rho \right) \end{array}\right\}.$$

Define $f_{n}\equiv Ef_{n}\left(\text{Y},\Phi \right)=\frac{1}{n}E\ln Z\left(\text{Y},\Phi \right)$. Then the main theorem of this paper is stated as follows:

### Theorem 1 (Replica-Symmetric Free Entropy).

*Suppose that* (*h*1)*–*(*h*2)*–*(*h*3)*–*(*h*4)*–*(*h*5) *hold. Then*, *for the GLM* **12***,*

$$\underset{n\to \infty }{\lim}f_{n}=\underset{q\in \left[0,\rho \right]}{\sup}\underset{r\geq 0}{\inf}f_{RS}\left(q,r\right)=\underset{\left(q,r\right)\in \Gamma }{\sup}f_{RS}\left(q,r\right).$$

Moreover, as one can see in *SI Appendix*, the “supinf” and the supremum over Γ above are achieved over the same couples. Under stronger assumptions on $P_{0}$ and $P_{out}$, one can show (*Theorem 6* in *SI Appendix*) that $f_{n}\left(Y,\Phi \right)$ concentrates around its mean $f_{n}$ and thus obtains convergence in probability **3**.

An immediate corollary of *Theorem 1* is the limiting expression of the mutual information I(X*;Y|Φ)≡E⁡ln⁡P(Y,X*|Φ)−E⁡ln(P(Y|Φ)P(X*)) between the observations and the unknown vector:

### Corollary 1 (Mutual Information).

*Under the same hypotheses as in Theorem 1*, *the mutual information for the GLM* **12** *verifies*

$$\begin{array}{l} \underset{n\to \infty }{\lim}\frac{1}{n}I\left(\text{X}*;\text{Y}|\Phi \right)=\underset{q\in \left[0,\rho \right]}{\inf}\underset{r\geq 0}{\sup}i_{RS}\left(q,r\right)=\underset{\left(q,r\right)\in \Gamma }{\inf}i_{RS}\left(q,r\right),\\ i_{RS}\left(q,r\right)\equiv \alpha \Psi_{P_{out}}\left(\rho ;\rho \right)-\alpha \Psi_{P_{out}}\left(q;\rho \right)-\psi_{P_{0}}\left(r\right)+rq/2. \end{array}$$

Finally, we gather our main results related to the optimal errors in a single theorem (see *SI Appendix* for more details), including results on the optimality of the GAMP algorithm:

### Theorem 2 (Optimal Errors).

*Assume the same hypotheses as in Theorem 1. Then formula* **9** *for the generalization error is true as* n,m→∞*,* m/n→α *whenever the maximizer* q*(α) *of* **[3]** *is unique*, *which is the case for almost every* α*. If moreover all of the moments of* $P_{0}$ *are finite*, *then formula* **7** *for the overlap and the matrix-MMSE formula*

$$\frac{1}{n^{2}}E\left[\|\text{X}*\text{X}{*}^{⊺}-E_{P\left(\text{x}|\text{Y},\Phi \right)}{\left[\text{x}{\text{x}}^{⊺}\right]\|}_{\text{F}}^{2}\right]\to \rho^{2}-q*{\left(\alpha \right)}^{2}$$ [13]

*are true*, *where* ${\left\|-\right\|}_{F}$ *is the Frobenius norm.*

There are cases of GLMs (e.g., the signless output channel $\text{Y}=|\Phi \text{X}*|/\sqrt{n}+\text{Z}$) where the sign of X* simply cannot be estimated (thus the absolute value in **[7]**). This is why our general theorem is related to an error metric **13** insensitive to this ± symmetry. Nevertheless formula **8** for the signal MSE is formally valid when there is no such sign symmetry.

### Sketch of Proof by the Adaptive Interpolation Method.

We now give the main ideas behind the proof of *Theorem 1*. We defer to *SI Appendix* the details, as well as those of *Corollary 1* and *Theorem 2*.

We note a clarification about notation. The r.v. Y (and also Φ, X*, A, and Z) are called quenched because once the measurements are acquired, they are fixed. The expectation w.r.t. all quenched r.v. is denoted by E without a subscript. In contrast, expectation of annealed variables w.r.t. a posterior distribution at fixed quenched variables is denoted by Gibbs brackets ⟨−⟩.

#### Two scalar inference channels.

An important role in the proof is played by two simple scalar inference channels. The free entropy is expressed in terms of the free entropies of these channels. This “decoupling property” stands at the root of the replica approach in statistical physics.

The first scalar channel is an additive Gaussian channel. Suppose that we observe $Y_{0}=\sqrt{r}X_{0}+Z_{0}$ where $X_{0}∼P_{0}$ and $Z_{0}∼N\left(0,1\right)$ are independent. Consider the inference problem consisting of retrieving $X_{0}$ from the observation $Y_{0}$. The free entropy associated with this channel is the expectation of the logarithm of the normalization factor of the associated posterior $dP\left(x|Y_{0}\right)$ that is given by **[5]** (up to a constant).

The second scalar channel that appears naturally in the problem is linked to the channel $P_{out}$ through the following inference model. Suppose that $V,W*\overset{iid}{∼}N\left(0,1\right)$ where V is known while the inference problem is to recover the unknown W* from the observation ${\tilde{Y}}_{0}∼P_{out}\left(\cdot |\sqrt{q}V+\sqrt{\rho -q}W*\right)$ where ρ>0 and q∈[0,ρ]. The free entropy for this model, again given by a normalization factor of the posterior of w given ${\tilde{Y}}_{0}$ and V, is exactly **[6]**.

#### Interpolating the estimation problem.

To carry out the proof, we introduce an “interpolating estimation problem” that interpolates between the original problem $Y_{\mu }∼P_{out}\left(\cdot |\frac{1}{\sqrt{n}}{\left[\Phi \text{X}*\right]}_{\mu }\right)$ at t=0, with t∈[0,1] being the interpolation parameter, and the two scalar problems described above at t=1. For t∈(0,1) the interpolating estimation problem is a mixture of the original and the scalar problems. This interpolation scheme is inspired by the interpolation paths used by Talagrand (73) to study the perceptron. Due to a novel ingredient specific to the adaptive interpolation method (52), it allows us to obtain in a unified manner a complete proof of the replica formula for the free entropy and in the whole phase diagram.

Let q(t) and r(t) be two interpolation functions. Moreover define $S_{t,\mu }=S_{t,\mu }\left(\text{X}*,W{*}_{\mu },V_{\mu },\Phi \right)$ as

$$S_{t,\mu }\equiv \sqrt{\frac{1-t}{n}}{\left[\Phi \text{X}*\right]}_{\mu }+\sqrt{\int_{0}^{t}q\left(v\right)dv}V_{\mu }+\sqrt{\int_{0}^{t}\left(\rho -q\left(v\right)\right)dv}W{*}_{\mu },$$

where $V_{\mu },W{*}_{\mu }\overset{iid}{∼}N\left(0,1\right)$. Consider the following observation channels, with two types of observations obtained through

$$\left\{\begin{array}{cc} Y_{t,\mu } & ∼\;P_{out}\left(\cdot |S_{t,\mu }\right),\;\text{for}1\leq \mu \leq m,\\ Y_{t,i}^{\prime } & =\;\sqrt{\int_{0}^{t}r\left(v\right)dv}X{*}_{i}+Z_{i}^{\prime },\;\text{for}1\leq i\leq n, \end{array}\right.$$ [14]

where $\left(Z_{i}^{\prime }\right)\overset{iid}{∼}N\left(0,1\right)$. We assume that $\text{V}={\left(V_{\mu }\right)}_{\mu =1}^{m}$ is known and that the inference problem is to recover both $\text{W}*={\left(W{*}_{\mu }\right)}_{\mu =1}^{m}$ and $\text{X}*={\left(X{*}_{i}\right)}_{i=1}^{n}$ from the “t-dependent” observations ${\text{Y}}_{t}={\left(Y_{t,\mu }\right)}_{\mu =1}^{m}$ and ${\text{Y}}_{t}^{\prime }={\left(Y_{t,i}^{\prime }\right)}_{i=1}^{n}$.

We now understand that the integral of r(t) appearing in the second set of measurements in **[14]** and 1−t as well as the two integrals appearing in the first set all play the role of signal-to-noise ratios (SNRs) in the interpolating problem, with t giving more and more “power” (or weight) to the scalar inference channels when increasing. Here is the first crucial ingredient of our interpolation scheme. In classical interpolations, these SNRs would all take a trivial form, i.e., be linear in t, but here, the nontrivial integral dependency in t of the two latter SNRs allows for much more flexibility when choosing the interpolation path. This will allow us to actually choose the “optimal interpolation path” (this will become clear below).

Define $u_{y}\left(x\right)\equiv \ln P_{out}\left(y|x\right)$ and, with a slight abuse of notations, $s_{t,\mu }=s_{t,\mu }\left(\text{x},w_{\mu },V_{\mu },\Phi \right)\equiv S_{t,\mu }\left(\text{x},w_{\mu },V_{\mu },\Phi \right)$, the expression above with $\text{X}*,W{*}_{\mu }$ replaced by $\text{x},w_{\mu }$. We introduce the interpolating Hamiltonian $H_{t}=H_{t}\left(\text{x},\text{w};{\text{Y}}_{t},{\text{Y}}_{t}^{\prime },\Phi ,\text{V}\right)$

$$H_{t}\equiv -\overset{m}{\underset{\mu =1}{\sum }}u_{Y_{t,\mu }}\left(s_{t,\mu }\right)+\frac{1}{2}\overset{n}{\underset{i=1}{\sum }}\left(Y_{t,i}^{\prime }-\sqrt{\int_{0}^{t}r\left(v\right)dv}x_{i}\right)2$$

and the corresponding (t-dependent) Gibbs bracket ${\left\langle -\right\rangle }_{t}$ which is the expectation w.r.t. the joint posterior distribution of (x,w) given the observations ${\text{Y}}_{t},{\text{Y}}_{t}^{\prime }$ (and Φ,V), defined as

$${\left\langle L\left(\text{x},\text{w}\right)\right\rangle }_{t}\equiv Z_{t}\left({\text{Y}}_{t},{\text{Y}}_{t}^{\prime },\Phi ,\text{V}\right)-1\int dP_{0}\left(\text{x}\right)D\text{w}L\left(\text{x},\text{w}\right)e^{-H_{t}},$$

for every continuous bounded test function L. Here $Z_{t}\equiv \int dP_{0}\left(\text{x}\right)D\text{w}\exp\left\{-H_{t}\left(\text{x},\text{w};{\text{Y}}_{t},{\text{Y}}_{t}^{\prime },\Phi ,\text{V}\right)\right\}$ is the appropriate normalization, and Dw is the standard Gaussian measure. Finally we introduce

$$f_{n}\left(t\right)\equiv \frac{1}{n}E\ln Z_{t}\left(\text{Y},\text{Y}\prime ,\Phi ,\text{V}\right)$$

which is the interpolating free entropy. One verifies easily that

$$\left\{\begin{array}{cc} f_{n}\left(0\right) & =f_{n}-\frac{1}{2},\\ f_{n}\left(1\right) & =\;\psi_{P_{0}}\left(\int_{0}^{1}r\left(t\right)dt\right)-\frac{1+\int_{0}^{1}r\left(t\right)dt\rho }{2}+\frac{m}{n}\Psi_{P_{out}}\left(\int_{0}^{1}q\left(t\right)dt;\rho \right). \end{array}\right.$$ [15]

Now comes another crucial property of the interpolating model: It is such that at t=0 we recover the original problem and thus $f_{n}\left(0\right)=f_{n}-1/2$ (the constant 1/2 comes from the purely noisy measurements of the second channel in **[14]**), while at t=1 we have the two scalar inference channels and thus the associated terms $\psi_{P_{0}}$ and $\Psi_{P_{out}}$ appear in $f_{n}\left(1\right)$. These are precisely the terms appearing in the free entropy potential **4**.

#### Entropy variation along the interpolation.

From the understanding of the previous section, it is natural to evaluate the variation of entropy along the interpolation, which allows us to “compare” the original and purely scalar models due to the identity

$$f_{n}=f_{n}\left(0\right)+\frac{1}{2}=f_{n}\left(1\right)-\int_{0}^{1}f_{n}^{\prime }\left(t\right)+\frac{1}{2},$$ [16]

where the first equality follows from **[15]** (the prime means the derivative). Then by choosing the optimal interpolation path due to the nontrivial SNR dependencies in t, we will be able to show the equality between the replica formula and the true free entropy $f_{n}$.

We thus compute the t derivative of the free entropy (see *SI Appendix* for the details of this calculation). It is given by

$$\begin{array}{ll} f_{n}^{\prime }\left(t\right) & =\frac{r\left(t\right)q\left(t\right)}{2}-\frac{r\left(t\right)\rho }{2}+O_{n}\left(1\right)\\ & -\frac{1}{2}E\langle \left(\frac{1}{n}\overset{m}{\underset{\mu =1}{\sum }}u_{Y_{t,\mu }}^{\prime }\left(S_{t,\mu }\right)u_{Y_{t,\mu }}^{\prime }\left(s_{t,\mu }\right)-r\left(t\right)\right)\left(Q-q\left(t\right)\right)\rangle_{t}, \end{array}$$ [17]

where $O_{n}\left(1\right)$ is a quantity that goes to 0 in the n,m→∞ limit, uniformly in t, and the overlap is $Q=Q_{n}\equiv n^{-1}\sum_{i=1}^{n}X{*}_{i}x_{i}$.

We now state a crucial result in an informal way and refer to *SI Appendix* for precise statements. Formally, the overlap concentrates around its mean (for all t∈[0,1]), a behavior called “replica-symmetric” in statistical physics. To make this statement mathematically rigorous, one has to slightly modify the interpolating model by adding a “side channel” that brings vanishingly small additional information about X* without affecting the asymptotic free entropy density. This perturbation forces the overlap to concentrate. Effectively, one can use the following formal formula (see *SI Appendix*, section 4.3, *Lemma 2* for a precise statement):

$${Var}_{t}\left(Q\right)=E\langle {\left(Q-E{\left\langle Q\right\rangle }_{t}\right)}^{2}\rangle_{t}=O_{n}\left(1\right).$$ [18]

#### Canceling the remainder.

Note from **[15]** and **[4]** that the first two terms appearing in **[17]** are precisely the missing ones to obtain the expression of the potential on the r.h.s. of **[16]**. Thus, we want to “cancel” the Gibbs bracket in **[17]**. This term is called the remainder. To prove the replica formula, we have to show that this remainder vanishes, which was until now a difficult task. But due to the freedom of choice of the interpolation path allowed by the interpolating function q, we are able to do so by “adapting” the interpolation (thus the name of the method). Thus, we want to choose $q\left(t\right)=E{\left\langle Q\right\rangle }_{t}\approx Q$ because of **[18]**. However, $E{\left\langle Q\right\rangle }_{t}$ is a function of $\int_{0}^{t}q\left(v\right)dv$. The equation $q\left(t\right)=E{\left\langle Q\right\rangle }_{t}\in \left[0,\rho \right]$ is therefore an order 1 differential equation over $t\mapsto \int_{0}^{t}q\left(v\right)dv$. Assume for the moment that this equation has a solution over [0,1]. Once the solution $q_{n}^{\left(r\right)}$ is selected, the Cauchy–Schwarz inequality applied to the remainder allows us to show that its absolute value is upper bounded by $C\sqrt{{Var}_{t}\left(Q\right)}$ for some constant C>0 independent of n and t. Therefore from **[17]** and **[18]**, for 0≤t≤1 we get

$$f_{n}^{\prime }\left(t\right)=\frac{r\left(t\right)}{2}q_{n}^{\left(r\right)}\left(t\right)-\frac{r\left(t\right)\rho }{2}+O_{n}\left(1\right).$$

Finally combining this with **[15]** and **[16]** leads to

$$\begin{array}{l} f_{n}=\psi_{P_{0}}\left(\int_{0}^{1}r\left(t\right)dt\right)+\frac{m}{n}\Psi_{P_{out}}\left(\int_{0}^{1}q_{n}^{\left(r\right)}\left(t\right)dt;\rho \right)-\frac{1}{2}\int_{0}^{1}r\left(t\right)q_{n}^{\left(r\right)}\left(t\right)dt+O_{n}\left(1\right). \end{array}$$ [19]

This important equality is obtained due to the choice of the optimal interpolation path $q_{n}^{\left(r\right)}\left(t\right)$ permitted by the method.

#### Matching bounds and end of proof.

We now possess all of the necessary tools to finish the sketch of the proof of *Theorem 1*. We first prove that $\underset{n\to \infty }{\lim}f_{n}=\underset{r\geq 0}{\sup}\underset{q\in \left[0,\rho \right]}{\inf}f_{RS}\left(q,r\right)$. Then in *SI Appendix*, we show that (i) this is also equal to $\underset{q\in \left[0,\rho \right]}{\sup}\underset{r\geq 0}{\inf}f_{RS}\left(q,r\right)$, which gives the first equality of the theorem, and (ii) that this supinf is attained at the supremum of the state evolution fixed points, which gives the second equality.

##### Lower bound.

Choose *r(t)* = *r* the constant function. Identity **19** implies ${lim inf}_{n\to \infty }f_{n}\geq \underset{q\in \left[0,\rho \right]}{\inf}f_{RS}\left(q,r\right)$. This is true for all r≥0 and thus

$$\underset{n\to \infty }{lim inf}f_{n}\geq \underset{r\geq 0}{\sup}\underset{q\in \left[0,\rho \right]}{\inf}f_{RS}\left(q,r\right).$$ [20]

##### Upper bound.

We show in *SI Appendix* that $\Psi_{P_{out}}^{\prime }$ is nonnegative, continuous, and bounded and thus we can define $K\equiv 2\alpha \max_{q\in \left[0,\rho \right]}\Psi_{P_{out}}^{\prime }\left(q;\rho \right)\in R_{+}$. Consequently we can complete the general differential equation satisfied by *q(t)* by choosing *r(t)* as the solution of (see *SI Appendix* for more details)

$$r\left(t\right)=2\alpha \Psi_{P_{\text{out}}}^{\prime }\left(\int_{0}^{1}q_{n}^{\left(r\right)}\left(t\right)dt;\rho \right)\in \left[0,K\right]$$

In *SI Appendix* we show that a solution exists. Moreover from **[19]** and convexity of **[5]** and **[6]** we can assert

$$f_{n}\leq \int_{0}^{1}f_{RS}\left(q_{n}^{\left(r\right)}\left(t\right),r\left(t\right)\right)dt+O_{n}\left(1\right).$$

Finally note that if we denote $r{*}_{n}$ the solution of the ODE

$$f_{RS}\left(\int_{0}^{1}q_{n}^{\left(r{*}_{n}\right)}\left(t\right)dt,r{*}_{n}\right)=\underset{q\in \left[0,\rho \right]}{\inf}f_{RS}\left(q,r{*}_{n}\right).$$

Indeed, the function $g_{r{*}_{n}}:q\in \left[0,\rho \right]\mapsto f_{RS}\left(q,r{*}_{n}\right)$ is convex (*SI Appendix*) and its derivative is $g_{r{*}_{n}}^{\prime }\left(q\right)=\alpha \Psi_{P_{out}}^{\prime }\left(q\right)-r{*}_{n}/2$. Since $g_{r{*}_{n}}^{\prime }\left(\int_{0}^{1}q_{n}^{\left(r{*}_{n}\right)}\left(t\right)dt\right)=0$ by definition of $r{*}_{n}$, the minimum of $g_{r{*}_{n}}$ is necessarily achieved at $\int_{0}^{1}q_{n}^{\left(r{*}_{n}\right)}\left(t\right)dt$. We thus have

$$\underset{n\to \infty }{lim sup}f_{n}\leq \underset{r\geq 0}{\sup}\underset{q\in \left[0,\rho \right]}{\inf}f_{RS}\left(q,r\right)$$

which, when combined with **[20]**, allows us to deduce the result

$$\underset{n\to \infty }{\lim}f_{n}=\underset{r\geq 0}{\sup}\underset{q\in \left[0,\rho \right]}{\inf}f_{RS}\left(q,r\right).$$

## Supplementary Material

Supplementary File

## References

[1] Nelder J, Wedderburn R (1972). Generalized linear models. J R Stat Soc Ser A.

[2] McCullagh P (1984). Generalized linear models. Eur J Oper Res.

[3] Fienup JR (1982). Phase retrieval algorithms: A comparison. Appl Opt.

[4] Demanet L, Hand P (2014). Stable optimizationless recovery from phaseless linear measurements. J Fourier Anal Appl.

[5] Candes EJ, Strohmer T, Voroninski V (2013). Phaselift: Exact and stable signal recovery from magnitude measurements via convex programming. Commun Pure Appl Math.

[6] Boufounos PT, Baraniuk RG (2008). 1-bit compressive sensing. 42nd Annual Conference on Information Sciences and Systems (CISS).

[7] Bühlmann P, Van De Geer S (2011). Statistics for High-Dimensional Data: Methods, Theory and Applications.

[8] LeCun Y, Bengio Y, Hinton G (2015). Deep learning. Nature.

[9] Donoho DL, Tanner J (2005). Sparse nonnegative solution of underdetermined linear equations by linear programming. Proc Natl Acad Sci USA.

[10] Candes EJ, Tao T (2006). Near-optimal signal recovery from random projections: Universal encoding strategies?. IEEE Trans Inf Theory.

[11] Donoho DL, Maleki A, Montanari A (2009). Message-passing algorithms for compressed sensing. Proc Natl Acad Sci USA.

[12] Rangan S, Kuleshov A, Blinovsky VM, Ephremides A (2011). Generalized approximate message passing for estimation with random linear mixing. IEEE International Symposium on Information Theory Proceedings (ISIT).

[13] Zdeborová L, Krzakala F (2016). Statistical physics of inference: Thresholds and algorithms. Adv Phys.

[14] Kamilov U, Goyal VK, Rangan S (2011). Optimal quantization for compressive sensing under message passing reconstruction. IEEE International Symposium on Information Theory Proceedings (ISIT).

[15] Xu Y, Kabashima Y, Zdeborová L (2014). Bayesian signal reconstruction for 1-bit compressed sensing. J Stat Mech Theory Exp.

[16] Schniter P, Rangan S (2015). Compressive phase retrieval via generalized approximate message passing. IEEE Trans Signal Process.

[17] Bayati M, Montanari A (2012). The lasso risk for Gaussian matrices. IEEE Trans Inf Theory.

[18] El Karoui N, Bean D, Bickel PJ, Lim C, Yu B (2013). On robust regression with high-dimensional predictors. Proc Natl Acad Sci USA.

[19] Donoho D, Montanari A (2016). High dimensional robust m-estimation: Asymptotic variance via approximate message passing. Probab Theory Relat Fields.

[20] Gribonval R, Machart P (2013). https://papers.nips.cc/book/advances-in-neural-information-processing-systems-26-2013.

[21] Advani M, Ganguli S (2016). https://papers.nips.cc/book/advances-in-neural-information-processing-systems-29-2016.

[22] Gardner E, Derrida B (1989). Three unfinished works on the optimal storage capacity of networks. J Phys A Math Gen.

[23] Seung HS, Sompolinsky H, Tishby N (1992). Statistical mechanics of learning from examples. Phys Rev A.

[24] Watkin TLH, Rau A, Biehl M (1993). The statistical mechanics of learning a rule. Rev Mod Phys.

[25] Engel A, Van den Broeck C (2001). Statistical Mechanics of Learning.

[26] Engel A, Reimers L (1994). Reliability of replica symmetry for the generalization problem in a toy multilayer neural network. Europhys Lett.

[27] Bex GJ, Serneels R, den Broeck CV (1995). Storage capacity and generalization error for the reversed-wedge Ising perceptron. Phys Rev E.

[28] Hosaka T, Kabashima Y, Nishimori H (2002). Statistical mechanics of lossy data compression using a nonmonotonic perceptron. Phys Rev E.

[29] Baldassi C (2016). Unreasonable effectiveness of learning neural networks: From accessible states and robust ensembles to basic algorithmic schemes. Proc Natl Acad Sci USA.

[30] Martin CH, Mahoney MW (2017).

[31] Tishby N, Pereira FC, Bialek W (1999). The information bottleneck method. Proceedings of the 37th Annual Allerton Conference on Communication, Control and Computing.

[32] Shwartz-Ziv R, Tishby N (2017).

[33] Shannon CE (1948). A mathematical theory of communication, part i, part ii. Bell Syst Tech J.

[34] Tanaka T (2002). A statistical-mechanics approach to large-system analysis of CDMA multiuser detectors. IEEE Trans Inf Theory.

[35] Guo D, Verdú S (2005). Randomly spread CDMA: Asymptotics via statistical physics. IEEE Trans Inf Theory.

[36] Barron AR, Joseph A (2010). Toward fast reliable communication at rates near capacity with Gaussian noise. IEEE International Symposium on Information Theory (ISIT).

[37] Barbier J, Krzakala F (2017). Approximate message-passing decoder and capacity-achieving sparse superposition codes. IEEE Trans Inf Theory.

[38] Rush C, Greig A, Venkataramanan R (2017). Capacity-achieving sparse superposition codes via approximate message passing decoding. IEEE Trans Inf Theory.

[39] Barbier J, Dia M, Macris N (2016). Threshold saturation of spatially coupled sparse superposition codes for all memoryless channels. IEEE Information Theory Workshop (ITW).

[40] Barbier J, Dia M, Macris N (2017).

[41] Mézard M (1989). The space of interactions in neural networks: Gardner’s computation with the cavity method. J Phys A Math Gen.

[42] Bolthausen E (2014). An iterative construction of solutions of the TAP equations for the Sherrington–Kirkpatrick model. Commun Math Phys.

[43] Bayati M, Montanari A (2011). The dynamics of message passing on dense graphs, with applications to compressed sensing. IEEE Trans Inf Theory.

[44] Bayati M, Lelarge M, Montanari A (2015). Universality in polytope phase transitions and message passing algorithms. Ann Appl Probab.

[45] Javanmard A, Montanari A (2013). State evolution for general approximate message passing algorithms, with applications to spatial coupling. Inf Inference.

[46] Barbier J, Dia M, Macris N, Krzakala F, Do M, Hovakimyan N (2016). The mutual information in random linear estimation. 54th Annual Allerton Conference on Communication, Control, and Computing.

[47] Barbier J, Macris N, Dia M, Krzakala F (2017).

[48] Reeves G, Pfister HD (2016). The replica-symmetric prediction for compressed sensing with Gaussian matrices is exact. IEEE International Symposium on Information Theory (ISIT).

[49] Mézard M, Parisi G, Virasoro MA (1987). Spin Glass Theory and Beyond.

[50] Györgyi G (1990). First-order transition to perfect generalization in a neural network with binary synapses. Phys Rev A.

[51] Sompolinsky H, Tishby N, Seung HS (1990). Learning from examples in large neural networks. Phys Rev Lett.

[52] Barbier J, Macris N (2017).

[53] Coja-Oghlan A, Krzakala F, Perkins W, Zdeborova L, Hatami H, McKenzie P, King V (2017). Information-theoretic thresholds from the cavity method. Proceedings of the 49th Annual ACM SIGACT Symposium on Theory of Computing (STOC).

[54] Guo D, Shamai S, Verdú S (2005). Mutual information and minimum mean-square error in Gaussian channels. IEEE Trans Inf Theory.

[55] Opper M, Haussler D (1991). Generalization performance of Bayes optimal classification algorithm for learning a perceptron. Phys Rev Lett.

[56] Thouless DJ, Anderson PW, Palmer RG (1977). Solution of ‘solvable model of a spin glass’. Philos Mag.

[57] Kabashima Y (2008). Inference from correlated patterns: A unified theory for perceptron learning and linear vector channels. J Phys Conf Ser.

[58] Donoho DL, Javanmard A, Montanari A (2013). Information-theoretically optimal compressed sensing via spatial coupling and approximate message passing. IEEE Trans Inf Theory.

[59] Opper M, Winther O (1996). Mean field approach to Bayes learning in feed-forward neural networks. Phys Rev Lett.

[60] Opper M, Winther O (2001). Tractable approximations for probabilistic models: The adaptive Thouless-Anderson-Palmer mean field approach. Phys Rev Lett.

[61] Mondelli M, Montanari A (2017).

[62] Barbier J, Krzakala F, Macris N, Miolane L, Zdeborová L (2017). https://github.com/sphinxteam/GeneralizedLinearModel2017.

[63] Fletcher AK, Rangan S (2018). Iterative reconstruction of rank-one matrices in noise. Inf Inference.

[64] Hansel D, Mato G, Meunier C (1992). Memorization without generalization in a multilayered neural network. Europhys Lett.

[65] Pedregosa F (2011). Scikit-learn: Machine learning in Python. J Machine Learn Res.

[66] Chollet F (2015). https://github.com/fchollet/keras.

[67] Wu Y, Verdú S (2010). Rényi information dimension: Fundamental limits of almost lossless analog compression. IEEE Trans Inf Theory.

[68] Krzakala F, Mézard M, Sausset F, Sun Y, Zdeborová L (2012). Statistical-physics-based reconstruction in compressed sensing. Phys Rev X.

[69] Maleki A, Anitori L, Yang Z, Baraniuk RG (2013). Asymptotic analysis of complex lasso via complex approximate message passing (CAMP). IEEE Trans Inf Theory.

[70] Soltanolkotabi M (2017).

[71] Rosenblatt F (1957).

[72] Guerra F, Toninelli FL (2002). The thermodynamic limit in mean field spin glass models. Commun Math Phys.

[73] Talagrand M (2010). Mean Field Models for Spin Glasses: Volume I: Basic Examples.

