# Supplementary material for: Optimal errors and phase transitions in high-dimensional generalized linear models
Source: Proc Natl Acad Sci U S A. 2019 Mar 1;116(12):5451–60. doi: 10.1073/pnas.1802705116 (PMC6431156; doi:10.1073/pnas.1802705116)
Supplement: Supplementary File [file pnas.1802705116.sapp.pdf]

Optimal Errors and Phase Transitions in High-Dimensional Generalized Linear Models: Supplementary Informations

Jean Barbier[◊⋆⊗], Florent Krzakala[⋆], Nicolas Macris[†], Léo Miolane[∗⊗] and Lenka Zdeborová[∪]

Contents

1 Setting **2**
1.1 Generalized linear estimation: Problem statement 2
1.2 The teacher-student scenario 4
1.3 Two scalar inference channels 5

2 Main results **6**
2.1 Replica-symmetric formula and mutual information 6
2.2 Optimal reconstruction (or estimation) error 9
2.3 Optimal generalization (or prediction) error 10
2.4 Optimality of the generalized approximate message-passing algorithm 11
2.5 Optimal denoising error 12

3 Application to concrete situations **13**
3.1 Generic observations 13
3.2 Phase diagram of perfect learning 15
3.3 Examples of optimal generalization error 18

4 Proof of the replica formula by the adaptive interpolation method **21**
4.1 Interpolating estimation problem 22
4.2 Free entropy variation along the interpolation path 24
4.3 Overlap concentration and fundamental sum rule 25
4.4 Lower and upper matching bounds 26

5 Proofs of the limits of optimal errors **29**
5.1 Optimal generalization error: Proof of Theorem 4 29
5.2 Generalization error of GAMP: Proof of Proposition 2 35
5.3 Limit of the overlap: Proof of Theorem 2 36
5.4 Denoising error: Proof of Corollary 4 44

◊ Quantitative Life Sciences, International Center for Theoretical Physics, Trieste, Italy.
⋆ Laboratoire de Physique de l'Ecole Normale Supérieure, CNRS & Université Pierre et Marie Curie & École Normale Supérieure & PSL Université, Paris, France.
† Laboratoire de Théorie des Communications, Faculté Informatique et Communications, Ecole Polytechnique Fédérale de Lausanne, Suisse.
∗ Département d'Informatique de l'ENS, École Normale Supérieure & CNRS & PSL Research University & Inria, Paris, France.
∪ Institut de Physique Théorique, CNRS & CEA & Université Paris-Saclay, Saclay, France.
⊗ Corresponding authors: jbarbier@ictp.it, leo.miolane@gmail.com

Appendix A Some technicalities 45
A.1 The Nishimori identity . . . 45
A.2 Unicity of the optimizer q^* of the replica formula: Proof of Proposition 1 . . . 46
A.3 Continuity properties of the mutual information . . . 46
A.4 A simple consequence of hypotheses (h1)-(h2)-(h3)-(h4) . . . 49
A.5 Derivative of the interpolating free entropy: Proof of Proposition 3 . . . 50
A.6 Boundedness of an overlap fluctuation . . . 53
A.7 Proof of Proposition 6 . . . 54

Appendix B Some properties of the scalar channels 54
B.1 The additive Gaussian scalar channel . . . 54
B.2 The non-linear scalar channel . . . 55

Appendix C Approximation 61
C.1 Relaxing the hypotheses on P_0 and $\mathbf{\Phi}$ . . . 62
C.2 Relaxing the hypotheses on φ . . . 63
C.3 The case of discrete channels: Removing the Gaussian noise . . . 64

Appendix D Some sup-inf formulas 65

Appendix E Concentration of free entropy and overlaps 68
E.1 Concentration of the free entropy . . . 68
E.2 Concentration of the overlap . . . 72

Appendix F Details on numerics 76
F.1 General purpose algorithms . . . 77
F.2 Evaluating the replica formula . . . 77
F.3 Breaking the symmetry in GAMP . . . 78

1 Setting

1.1 Generalized linear estimation: Problem statement

We give a formal description of the observation model to which our results apply. The generalized linear *model* covers both the *estimation* (or inference) problem and the supervised *learning* problems (see Sec. 3).

Let $n, m \in \mathbb{N}^*$. Let P_0 be a probability distribution over $\mathbb{R}$ and let $(X_i^*)_{i=1}^n \overset{\text{iid}}{\sim} P_0$ be the components of a signal vector $\mathbf{X}^*$ (this is also denoted $\mathbf{X}^* \overset{\text{iid}}{\sim} P_0$). We fix a function $\varphi : \mathbb{R} \times \mathbb{R}^{k_A} \to \mathbb{R}$ and consider $(\mathbf{A}_\mu)_{\mu=1}^m \overset{\text{iid}}{\sim} P_A$, where P_A is a probability distribution over $\mathbb{R}^{k_A}$, $k_A \in \mathbb{N}$. We acquire m measurements through

$$Y_\mu = \varphi\Big(\frac{1}{\sqrt{n}}[\mathbf{\Phi}\mathbf{X}^*]_\mu, \mathbf{A}_\mu\Big) + \sqrt{\Delta} Z_\mu \,, \qquad 1 \le \mu \le m \,, \tag{1}$$

where $(Z_\mu)_{\mu=1}^m \overset{\text{iid}}{\sim} \mathcal{N}(0,1)$ is an additive Gaussian noise, $\Delta \ge 0$, and $\mathbf{\Phi}$ is a $m \times n$ measurement matrix with independent entries that have zero mean and unit variance. The estimation problem is to recover $\mathbf{X}^*$ from the knowledge of $\mathbf{Y} = (Y_\mu)_{\mu=1}^m$, φ, $\mathbf{\Phi}$, Δ, P_0 and P_A (the realization of the random stream $\mathbf{A}$ itself, if present in the model, is unknown). We use the notation $[\mathbf{\Phi}\mathbf{X}^*]_\mu = \sum_{i=1}^n \Phi_{\mu i} X_i^*$. When $\varphi(x, \mathbf{A}) = \varphi(x) = x$ we have a random linear estimation problem, whereas if, say, $\varphi(x) = \text{sgn}(x)$ we have a noisy single layer perceptron. Sec. 3 discusses various examples related to non-linear estimation and supervised learning.

Denote the prior over the signal as $dP_0(\mathbf{x}) = \prod_{i=1}^n dP_0(x_i)$, and similarly $dP_A(\mathbf{a}) = \prod_{\mu=1}^m dP_A(\mathbf{a}_\mu)$. It is also fruitful to think of the measurements as the outputs of a "channel",

$$Y_\mu \sim P_{\rm out}\Big(\cdot \,\Big|\frac{1}{\sqrt{n}}[\mathbf{\Phi}\mathbf{X}^*]_\mu\Big)\,. \tag{2}$$

When $\Delta > 0$ the transition kernel $P_{\rm out}$ admits a transition density with respect to (w.r.t.) Lebesgue's measure, given by

$$P_{\rm out}(y|x) = \frac{1}{\sqrt{2\pi\Delta}}\int dP_A(\mathbf{a}) e^{-\frac{1}{2\Delta}(y-\varphi(x,\mathbf{a}))^2}\,. \tag{3}$$

When $\Delta = 0$, we will only consider discrete channels where φ takes values in $\mathbb{N}$[1]. In that case $P_{\rm out}$ admits a transition density with respect the counting measure on $\mathbb{N}$ given by (here $\mathbf{1}(\cdot)$ is the indicator function)

$$P_{\rm out}(y|x) = \int dP_A(\mathbf{a})\mathbf{1}(y = \varphi(x,\mathbf{a}))\,. \tag{4}$$

Note that for deterministic models, $\mathbf{A}$ in (1) is absent and thus the associated $\int dP_A(\mathbf{a})$ integral in (3)-(4) simply disappears. In fact (1) is sometimes called a "random function representation" of a transition kernel $P_{\rm out}$. Our analysis uses both representations (1) and (2).

Throughout this paper we often adopt the language of statistical mechanics. In particular the random variables $\mathbf{Y}$ (and also $\mathbf{\Phi}$, $\mathbf{X}^*$, $\mathbf{A}$, $\mathbf{Z}$) are called *quenched* variables because once the measurements are acquired they have a "fixed realization". An expectation taken w.r.t. *all* quenched random variables appearing in an expression will simply be denoted by $\mathbb{E}$ *without* subscript. Subscripts are only used when the expectation carries over a subset of random variables appearing in an expression or when some confusion could arise.

A fundamental role is played by the posterior distribution of (the signal) $\mathbf{x}$ given the quenched measurements $\mathbf{Y}$ (recall that $\mathbf{X}^*$, $\mathbf{A}$ and $\mathbf{Z}$ are unknown). According to the Bayes formula this posterior is

$$dP(\mathbf{x}|\mathbf{Y},\mathbf{\Phi}) = \frac{1}{\mathcal{Z}(\mathbf{Y},\mathbf{\Phi})} dP_0(\mathbf{x})\prod_{\mu=1}^m P_{\rm out}\Big(Y_\mu\Big|\frac{1}{\sqrt{n}}[\mathbf{\Phi}\mathbf{x}]_\mu\Big) \tag{5}$$

$$= \frac{1}{\mathcal{Z}(\mathbf{Y},\mathbf{\Phi})} dP_0(\mathbf{x}) e^{-\mathcal{H}(\mathbf{x};\mathbf{Y},\mathbf{\Phi})} \tag{6}$$

where the *Hamiltonian* is defined as

$$\mathcal{H}(\mathbf{x};\mathbf{Y},\mathbf{\Phi}) := -\sum_{\mu=1}^m \ln P_{\rm out}\Big(Y_\mu\Big|\frac{1}{\sqrt{n}}[\mathbf{\Phi}\mathbf{x}]_\mu\Big) \tag{7}$$

and the *partition function* (the normalization factor) is defined as

$$\mathcal{Z}(\mathbf{Y},\mathbf{\Phi}) := \int dP_0(\mathbf{x}) e^{-\mathcal{H}(\mathbf{x};\mathbf{Y},\mathbf{\Phi})}\,. \tag{8}$$

From the point of view of statistical mechanics (6) is a Gibbs distribution and the integration over $dP_0(\mathbf{x})$ in the partition function is best thought as a "sum over annealed or fluctuating degrees of freedom". Let us introduce a standard statistical mechanics notation for the expectation w.r.t. the posterior (5), the so called

[1]Notice that this allows to study any channel whose outputs belong to a countable set S by applying a injection $u: S \to \mathbb{N}$ to the outputs.

Gibbs bracket $\langle - \rangle$ defined as

$$\langle g(\mathbf{x})\rangle := \mathbb{E}[g(\mathbf{X})|\mathbf{Y},\mathbf{\Phi}] = \int dP(\mathbf{x}|\mathbf{Y},\mathbf{\Phi})g(\mathbf{x}) \tag{9}$$

for any continuous bounded function g. The main quantity of interest here is the associated averaged *free entropy* (or minus the averaged *free energy*)

$$f_n := \frac{1}{n}\mathbb{E}\ln\mathcal{Z}(\mathbf{Y},\mathbf{\Phi})\,. \tag{10}$$

It is perhaps useful to stress that $\mathcal{Z}(\mathbf{Y},\mathbf{\Phi})$ is nothing else than the density of $\mathbf{Y}$ conditioned on $\mathbf{\Phi}$ so we have the explicit representation (used later on)

$$\begin{aligned} f_n &= \frac{1}{n}\mathbb{E}_{\mathbf{\Phi}}\int d\mathbf{Y}\mathcal{Z}(\mathbf{Y},\mathbf{\Phi})\ln\mathcal{Z}(\mathbf{Y},\mathbf{\Phi}) \\ &= \frac{1}{n}\mathbb{E}_{\mathbf{\Phi}}\int d\mathbf{Y}dP_0(\mathbf{X}^*)e^{-\mathcal{H}(\mathbf{X}^*;\mathbf{Y},\mathbf{\Phi})}\ln\int dP_0(\mathbf{x})\,e^{-\mathcal{H}(\mathbf{x};\mathbf{Y},\mathbf{\Phi})}\,, \end{aligned} \tag{11}$$

where $d\mathbf{Y} = \prod_{\mu=1}^m dY_\mu$. Thus f_n is minus the conditional entropy $-H(\mathbf{Y}|\mathbf{\Phi})/n$ of the measurements. One of the main contributions of this paper is the derivation, thanks to the adaptive interpolation method, of the thermodynamic limit $\lim_{n\to\infty} f_n$ in the "high-dimensional regime", namely when $n, m \to \infty$ while $m/n \to \alpha > 0$ (α is sometimes referred to as the "measurement rate" or "sampling rate").

1.2 The teacher-student scenario

We now describe an important conceptual setting, the *teacher-student scenario* (also called planted model), that allows to then define the optimal generalization error. We voluntarily employ terms coming from machine learning instead of the signal processing terminology used until here.

First the teacher randomly generates a *classifier* $\mathbf{X}^* \in \mathbb{R}^n$ (the signal in the estimation problem) with $\mathbf{X}^* \overset{\text{iid}}{\sim} P_0$ and an ensemble of m *patterns* (row-vectors) $\mathbf{\Phi}_\mu \in \mathbb{R}^n$ for $\mu = 1,\dots,m$ such that $\mathbf{\Phi}_\mu \overset{\text{iid}}{\sim} \mathcal{N}(\mathbf{0},\mathbf{I}_n)$. The teacher then chooses a model (φ, P_A, Δ) or equivalently P_{out}, which are linked through (3)-(4). The teacher then output *labels* $Y_\mu \in \mathbb{R}$ through (1) or (2) for $\mu = 1,\dots,m$.

The student is given the distribution P_0, the model (φ, P_A, Δ) or equivalently P_{out} and the training data composed of the pattern-label pairs $\{(Y_\mu;\mathbf{\Phi}_\mu)\}_{\mu=1}^m$ generated by the teacher. His (supervised) learning task is then to predict the labels associated with new, yet unseen, patterns from all this knowledge.

How does the teacher may evaluate the student's prediction capabilities? The teacher starts by randomly generating a new line of the matrix, or pattern, $\mathbf{\Phi}_{\text{new}}$. Then, still using the same $\mathbf{X}^*$, he generates the associated new label $Y_{\text{new}} \sim P_{\text{out}}(\cdot \mid \mathbf{\Phi}_{\text{new}} \cdot \mathbf{X}^*/\sqrt{n})$. He is now ready to evaluate the student generalization performance. For that purpose, an important quantity is the *generalization error* (or prediction error). If we denote $\widehat{Y}(\mathbf{\Phi}_{\text{new}},\mathbf{\Phi},\mathbf{Y})$ the estimator used by the student (which is thus a measurable function of the observations), the generalization error is defined as

$$\mathcal{E}_{\text{gen}}(\widehat{Y}) := \mathbb{E}\big[\big(Y_{\text{new}} - \widehat{Y}(\mathbf{\Phi}_{\text{new}},\mathbf{\Phi},\mathbf{Y})\big)^2\big]\,. \tag{12}$$

The optimal generalization error is then defined as the minimum of $\mathcal{E}_{\text{gen}}$ over all estimators $\widehat{Y}(\mathbf{\Phi}_{\text{new}},\mathbf{\Phi},\mathbf{Y})$:

$$\mathcal{E}_{\text{gen}}^{\text{opt}} := \min_{\widehat{Y}}\mathcal{E}_{\text{gen}}(\widehat{Y}) = \text{MMSE}(Y_{\text{new}}|\mathbf{\Phi}_{\text{new}},\mathbf{\Phi},\mathbf{Y}) = \mathbb{E}\big[\big(Y_{\text{new}} - \mathbb{E}[Y_{\text{new}}|\mathbf{\Phi}_{\text{new}},\mathbf{\Phi},\mathbf{Y}]\big)^2\big]\,. \tag{13}$$

Here, and for the rest of the paper, we define the minimum mean-square error (MMSE) function as follows:

Given two random variables $\mathbf{A}, \mathbf{B}$, the MMSE in estimating $\mathbf{A}$ given $\mathbf{B}$ is defined as

$$\mathrm{MMSE}(\mathbf{A}|\mathbf{B}) := \mathbb{E}\big[\|\mathbf{A} - \mathbb{E}[\mathbf{A}|\mathbf{B}]\|^2\big]\,, \tag{14}$$

where $\mathbb{E}[\mathbf{A}|\mathbf{B}]$ is the expectation of $\mathbf{A}$ with respect to its posterior given $\mathbf{B}$.

A word about notations: Let us emphasize on the link between the different notations that we use in the present supplementary material and in the main text. E.g., the expectation w.r.t. to the posterior of $Y_{\rm new}$ appearing in (13) can be written equivalently as:

$$\mathbb{E}[Y_{\rm new}|\mathbf{\Phi}_{\rm new}, \mathbf{\Phi}, \mathbf{Y}] = \mathbb{E}_{P_A(\mathbf{a})}\mathbb{E}_{P(\mathbf{x}|\mathbf{\Phi},\mathbf{Y})}\varphi\Big(\frac{\mathbf{\Phi}_{\rm new}\cdot\mathbf{x}}{\sqrt{n}}, \mathbf{a}\Big) = \mathbb{E}_{P_A(\mathbf{a})}\Big\langle\varphi\Big(\frac{\mathbf{\Phi}_{\rm new}\cdot\mathbf{x}}{\sqrt{n}}, \mathbf{a}\Big)\Big\rangle\,. \tag{15}$$

To see that, just write:

$$\begin{aligned}
\mathbb{E}[Y_{\rm new}|\mathbf{\Phi}_{\rm new}, \mathbf{\Phi}, \mathbf{Y}] &:= \int dY_{\rm new}\, Y_{\rm new} P(Y_{\rm new}|\mathbf{\Phi}_{\rm new}, \mathbf{\Phi}, \mathbf{Y}) \\
&= \int dY_{\rm new}\, Y_{\rm new} P_{\rm out}\Big(Y_{\rm new}\Big|\frac{1}{\sqrt{n}}\mathbf{\Phi}_{\rm new}\cdot\mathbf{x}\Big)\, dP(\mathbf{x}|\mathbf{Y}, \mathbf{\Phi}) \\
&= \Big\langle \int dY_{\rm new}\, Y_{\rm new} P_{\rm out}\Big(Y_{\rm new}\Big|\frac{1}{\sqrt{n}}\mathbf{\Phi}_{\rm new}\cdot\mathbf{x}\Big)\Big\rangle \\
&= \Big\langle \int dP_A(\mathbf{a}) dY_{\rm new}\, Y_{\rm new}\frac{1}{\sqrt{2\pi\Delta}} e^{-\frac{1}{2\Delta}\left\{Y_{\rm new}-\varphi\left(\frac{1}{\sqrt{n}}\mathbf{\Phi}_{\rm new}\cdot\mathbf{x},\mathbf{a}\right)\right\}^2}\Big\rangle \\
&= \mathbb{E}_{P_A(\mathbf{a})}\Big\langle\varphi\Big(\frac{1}{\sqrt{n}}\mathbf{\Phi}_{\rm new}\cdot\mathbf{x}, \mathbf{a}\Big)\Big\rangle\,.
\end{aligned} \tag{16}$$

Here we used definition (3) for the transition kernel, but using instead (4) would lead to the same identity.

1.3 Two scalar inference channels

An important role in our proof of the asymptotic expression of the free entropy is played by simple *scalar* inference channels. As we will see, the free entropy is expressed in terms of the free entropy of these channels. This "decoupling property" results from the mean-field approach in statistical physics, used through in the replica method to perform a formal calculation of the free entropy of the model [1, 2]. Let us now introduce these two scalar denoising models.

The first one is an additive Gaussian channel. Let $r \geq 0$, which plays the role of a signal-to-noise ratio (snr). Suppose that $X_0 \sim P_0$ and that we observe

$$Y_0 = \sqrt{r}\, X_0 + Z_0\,, \tag{17}$$

where $Z_0 \sim \mathcal{N}(0, 1)$ independently of X_0. Consider the inference problem consisting of retrieving X_0 from the observations Y_0. The associated posterior distribution is

$$dP(x|Y_0) = \frac{dP_0(x)e^{\sqrt{r}\,Y_0 x - rx^2/2}}{\int dP_0(x)e^{\sqrt{r}\,Y_0 x - rx^2/2}}\,. \tag{18}$$

In this expression all the x-independent terms have been simplified between the numerator and the normalization. The free entropy associated with this channel is just the expectation of the logarithm of the normalization factor

$$\psi_{P_0}(r) := \mathbb{E}\ln\int dP_0(x)e^{\sqrt{r}\,Y_0 x - rx^2/2}\,. \tag{19}$$

The basic properties of ψ_{P_0} are presented in Appendix B.1 .

The second scalar channel that appears naturally in the problem is linked to the transition kernel $P_{\rm out}$ through the following inference model. Suppose that $V, W^* \overset{\rm iid}{\sim} \mathcal{N}(0,1)$ where V is *known* while the inference problem is to recover the unknown W^* from the following observation

$$\widetilde{Y}_0 \sim P_{\rm out}\big(\,\cdot\mid \sqrt{q}\,V + \sqrt{\rho - q}\,W^*\big)\,, \tag{20}$$

where $\rho > 0$, $q \in [0, \rho]$. Notice that the channel (20) is equivalent to $\widetilde{Y}_0 = \varphi(\sqrt{q}\,V + \sqrt{\rho - q}\,W^*, \mathbf{A}) + \sqrt{\Delta}Z$ with $\Delta \geq 0$ and where $(\mathbf{A}, Z) \sim P_A \otimes \mathcal{N}(0,1)$, independently of V, W^*. The free entropy for this model, again related to the normalization of the posterior $dP(w|\widetilde{Y}_0, V)$, is

$$\Psi_{P_{\rm out}}(q;\rho) = \Psi_{P_{\rm out}}(q) := \mathbb{E}\ln \int \mathcal{D}w P_{\rm out}\big(\widetilde{Y}_0|\sqrt{q}\,V + \sqrt{\rho - q}\,w\big)\,, \tag{21}$$

where $\mathcal{D}w := dw(2\pi)^{-1/2}e^{-w^2/2}$ is the standard Gaussian measure. In (21) above, $P_{\rm out}$ denotes either the transition density with respect to Lebesgue's measure (given by (3)) in the case $\Delta > 0$, or the density with respect to the counting measure over $\mathbb{N}$ (given by (4)), in the case of a "discrete" channel (φ takes values in $\mathbb{N}$ and $\Delta = 0$). We prove in Appendix B.2 that this function is convex, differentiable and non-decreasing w.r.t. its first argument.

2 Main results

2.1 Replica-symmetric formula and mutual information

Let us now introduce our first main result, a single-letter *replica-symmetric formula* for the asymptotic free entropy of model (1), (2). The result holds under the following rather general hypotheses. We will consider two cases, that is when there is some Gaussian noise ($\Delta > 0$, see (h5.a) below) and the case without Gaussian noise ($\Delta = 0$, see (h5.b) below):

(h1) The prior distribution P_0 admits a finite third moment and has at least two points in its support.
(h2) There exists $\gamma > 0$ such that the sequence $(\mathbb{E}[|\varphi(\frac{1}{\sqrt{n}}[\mathbf{\Phi X}^*]_1, \mathbf{A}_1)|^{2+\gamma}])_{n\geq 1}$ is bounded.
(h3) The random variables $(\Phi_{\mu i})$ are independent with zero mean, unit variance and finite third moment that is bounded with n.
(h4) For almost-all values of $\mathbf{a} \in \mathbb{R}^{k_A}$ (w.r.t. P_A), the function $x \mapsto \varphi(x, \mathbf{a})$ is continuous almost everywhere.

We will also assume that one of the two following hypotheses hold:

(h5.a) $\Delta > 0$.
(h5.b) $\Delta = 0$ and φ takes values in $\mathbb{N}$.

Remark 1. *The above hypotheses are here stated using the "random function" representation of* (1)*. In many cases, it can be useful to state them using the "transition kernel" representation of* (2)*. The hypotheses (h2) and (h4) are respectively equivalent*[2] *to:*

(h2') There exists $\gamma > 0$ such that $\mathbb{E}[|Y_1|^{2+\gamma}]$ remains bounded with n.
(h4') $x \in \mathbb{R} \mapsto P_{\rm out}(\cdot|x)$ is continuous almost everywhere for the weak convergence.

Under the above hypothesis (h5.a) (respectively (h5.b)), the transition kernel $P_{\rm out}$ admits a density with respect to Lebesgue's measure on $\mathbb{R}$ (resp. the counting measure on $\mathbb{N}$) that will be denoted by $P_{\rm out}(\cdot|x)$.

[2]The implications (h2) $\Leftrightarrow$ (h2') and (h4) $\Rightarrow$ (h4') are obvious. If (h4') holds one can show, by inverting cumulative distribution functions, that there exists a function $\varphi : \mathbb{R} \times [0,1] \to \mathbb{R}$ such that (1) holds for $A_\mu \overset{\rm iid}{\sim} P_A = {\rm Unif}([0,1])$ and that (h4) is verified.

We will call the kernel $P_{\rm out}$ *informative* if there exists $y \in \mathbb{R}$ (resp. $y \in \mathbb{N}$) such that $P_{\rm out}(y \,|\, \cdot)$ is not equal almost everywhere to a constant. If $P_{\rm out}$ is not informative, it is not difficult to show that estimation is then impossible.

Let us define the *replica-symmetric potential* (or just potential). Call $\rho := \mathbb{E}[(X^*)^2]$ where $X^* \sim P_0$. Then the potential is

$$f_{\rm RS}(q, r; \rho) = f_{\rm RS}(q, r) := \psi_{P_0}(r) + \alpha \Psi_{P_{\rm out}}(q; \rho) - \frac{rq}{2} \,. \tag{22}$$

We define also $f_{\rm RS}(\rho, +\infty) = \lim_{r\to\infty} f_{\rm RS}(\rho, r)$. From now on denote $\psi'_{P_0}(r)$ and $\Psi'_{P_{\rm out}}(q) = \Psi'_{P_{\rm out}}(q;\rho)$ the derivatives of $\psi_{P_0}(r)$ and $\Psi_{P_{\rm out}}(q;\rho)$ w.r.t. their first argument. We need also to define the set of the critical points of $f_{\rm RS}$:

$$\Gamma := \left\{ (q, r) \in [0, \rho] \times (\mathbb{R}_+ \cup \{+\infty\}) \;\middle|\; \begin{array}{lcl} q & = & 2\psi'_{P_0}(r) \\ r & = & 2\alpha \Psi'_{P_{\rm out}}(q;\rho) \end{array} \right\} , \tag{23}$$

where, with a slight abuse of notation, we define $\psi'_{P_0}(+\infty) = \lim_{r\to\infty} \psi'_{P_0}(r)$ and $\Psi'_{P_{\rm out}}(\rho) = \lim_{q\to\rho} \Psi'_{P_{\rm out}}(q)$. These limits are well defined by convexity of ψ_{P_0} and $\Psi_{P_{\rm out}}$. The elements of Γ are called "fixed points of the state evolution". Our first main result is

Theorem 1 (Replica-symmetric formula for the free entropy)**.** *Suppose that hypotheses (h1)-(h2)-(h3)-(h4) hold. Suppose that either hypothesis (h5.a) or (h5.b) holds. Then, for the generalized linear estimation model* (1), (2) *the thermodynamic limit of the free entropy* (10) *verifies*

$$f_\infty := \lim_{n\to\infty} f_n = \sup_{q\in[0,\rho]} \inf_{r\geq 0} f_{\rm RS}(q, r) = \sup_{(q,r)\in\Gamma} f_{\rm RS}(q, r) \,. \tag{24}$$

Moreover, if $P_{\rm out}$ is informative, then the "$\sup\inf$*" and the supremum over Γ in* (24) *are achieved over the same couples (q, r).*

An immediate corollary of Theorem 1 is the limiting expression of the mutual information between the signal and the observations. To state the result, we need to introduce two mutual informations associated to the two scalar channels presented in Sec. 1.3, namely

$$I_{P_0}(r) := I(X_0; \sqrt{r}\, X_0 + Z_0) = \frac{r\rho}{2} - \psi_{P_0}(r) \tag{25}$$

for the channel (17) and

$$\mathcal{I}_{P_{\rm out}}(q) := I(W^*; \widetilde{Y}_0 | V) = \Psi_{P_{\rm out}}(\rho) - \Psi_{P_{\rm out}}(q) \tag{26}$$

for the channel (20).

Corollary 1 (Single-letter formula for the mutual information)**.** *The thermodynamic limit of the mutual information for model* (1), (2) *between the observations and the hidden variables verifies*

$$i_\infty := \lim_{n\to\infty} \frac{1}{n} I(\boldsymbol{X}^*; \mathbf{Y} | \mathbf{\Phi}) = \inf_{q\in[0,\rho]} \sup_{r\geq 0} i_{\rm RS}(q, r) = \inf_{(q,r)\in\Gamma} i_{\rm RS}(q, r) \,, \tag{27}$$

where

$$i_{\rm RS}(q, r) := I_{P_0}(r) + \alpha \mathcal{I}_{P_{\rm out}}(q) - \frac{r}{2}(\rho - q) \,. \tag{28}$$

Proof. This follows from a simple calculation:

$$\frac{1}{n}I(\mathbf{X}^*;\mathbf{Y}|\mathbf{\Phi}) = \frac{1}{n}H(\mathbf{Y}|\mathbf{\Phi}) - \frac{1}{n}H(\mathbf{Y}|\mathbf{X}^*,\mathbf{\Phi}) = -f_n + \frac{1}{n}\mathbb{E}\ln P(\mathbf{Y}|\mathbf{X}^*,\mathbf{\Phi})$$
$$= -f_n + \frac{m}{n}\mathbb{E}\ln P_{\text{out}}(Y_1\,|\,\mathbf{\Phi}_1\cdot\mathbf{X}^*/\sqrt{n})\,. \tag{29}$$

By the central limit theorem (that we can apply under hypotheses (h1)-(h3)) we have

$$S_n := \frac{1}{\sqrt{n}}\mathbf{\Phi}_1\cdot\mathbf{X}^* = \frac{1}{\sqrt{n}}\sum_{i=1}^{n}\Phi_{1,i}X_i^* \xrightarrow[n\to\infty]{(d)} \mathcal{N}(0,\rho)\,.$$

Now, under the hypotheses (h2)-(h4) and either (h5.a) or (h5.b) it is not difficult to verify that

$$\mathbb{E}\ln P_{\text{out}}(Y_1\,|\,\mathbf{\Phi}_1\cdot\mathbf{X}^*/\sqrt{n}) = \mathbb{E}\int dY P_{\text{out}}(Y|S_n)\ln P_{\text{out}}(Y|S_n)$$
$$\xrightarrow[n\to\infty]{} \mathbb{E}\int dY P_{\text{out}}(Y|\sqrt{\rho}\,V)\ln P_{\text{out}}(Y|\sqrt{\rho}\,V) = \Psi_{P_{\text{out}}}(\rho)$$

where $V\sim\mathcal{N}(0,1)$. We conclude, using (29):

$$\frac{1}{n}I(\mathbf{X}^*;\mathbf{Y}|\mathbf{\Phi}) = -f_n + \alpha\Psi_{P_{\text{out}}}(\rho) + o_n(1) \tag{30}$$

where $\lim_{n\to\infty} o_n(1) = 0$. □

The next proposition, proved in Appendix A.2, states that for almost every $\alpha > 0$ there is one unique optimizer q^* in (24) (or equivalently in (27)):

Proposition 1. *Assume that the assumptions of Theorem 1 hold and that P_{out} is informative. Define*

$$D^* := \Big\{\alpha > 0 \,\Big|\, \text{(24) (or equivalently (27)) admits a unique optimizer } q^*(\alpha)\Big\}\,. \tag{31}$$

the set D^* is equal to $\mathbb{R}_+^*$ minus some countable set. Moreover $\alpha\mapsto q^*(\alpha)$ is continuous on D^*.

As an application of Theorem 1 we can compute the free entropy of the "planted perceptron" on the hypercube and the sphere. This perceptron model has already been studied in physics [3] and more recently in statistics, where it is known as "one-bit compressed sensing" [4,5]. The limit of the free entropy follows from an application of Theorem 1 with $\varphi(x) = \text{sgn}(x)$ and $P_0 = \frac{1}{2}\delta_{-1} + \frac{1}{2}\delta_1$ (for the hypercube) or $P_0 = \mathcal{N}(0,1)$ (for the sphere). For $\mu\in\{1,\dots,m\}$ we define

$$S_\mu := \Big\{\mathbf{x}\in\mathbb{R}^n \,\Big|\, \text{sgn}(\mathbf{x}\cdot\mathbf{\Phi}_\mu) = \text{sgn}(\mathbf{X}^*\cdot\mathbf{\Phi}_\mu)\Big\}\,. \tag{32}$$

We will use the notation $\mathcal{N}(x) = \mathbb{P}(Z\le x)$ for $Z\sim\mathcal{N}(0,1)$. Let $\mathbb{S}_n$ be the unit sphere in $\mathbb{R}^n$ and μ_n the uniform probability measure on $\mathbb{S}_n$.

Corollary 2 (Free entropy of the planted perceptron). *Let* $Z, V \overset{\text{iid}}{\sim} \mathcal{N}(0,1)$. *We have*

$$
\begin{aligned}
&\frac{1}{n}\mathbb{E}\ln\Big(\#\bigcap_{\mu=1}^{m} S_\mu \cap \{-1,1\}^n\Big) \\
&\xrightarrow[n\to\infty]{} \ln(2) + \sup_{q\in[0,1)}\inf_{r\geq 0}\Big\{\mathbb{E}\ln\cosh(\sqrt{r}Z+r) + 2\alpha\mathbb{E}\Big[\mathcal{N}\Big(\frac{\sqrt{q}\,V}{\sqrt{1-q}}\Big)\ln\mathcal{N}\Big(\frac{\sqrt{q}\,V}{\sqrt{1-q}}\Big)\Big] - \frac{r(q+1)}{2}\Big\}, \quad (33)\\
&\frac{1}{n}\mathbb{E}\ln\mu_n\Big(\bigcap_{\mu=1}^{m} S_\mu \cap \mathbb{S}_n\Big) \\
&\xrightarrow[n\to\infty]{} \sup_{q\in[0,1)}\Big\{\frac{1}{2}\ln(1-q) + 2\alpha\mathbb{E}\Big[\mathcal{N}\Big(\frac{\sqrt{q}\,V}{\sqrt{1-q}}\Big)\ln\mathcal{N}\Big(\frac{\sqrt{q}\,V}{\sqrt{1-q}}\Big)\Big] + \frac{q}{2}\Big\}. \quad (34)
\end{aligned}
$$

2.2 Optimal reconstruction (or estimation) error

We first consider the problem of estimating $\mathbf{X}^*$ given $\mathbf{Y}$ and $\mathbf{\Phi}$. The following theorem states that the optimizer $q^*(\alpha)$ of the replica-symmetric formula (24) gives the asymptotic correlation between the planted solution $\mathbf{X}^*$ and a typical sample from the posterior distribution $P(\cdot\,|\,\mathbf{Y},\mathbf{\Phi})$:

Theorem 2 (Limit of the overlap). *Assume that all the moments of* P_0 *are finite and that* P_{out} *is informative. Assume that (h1)-(h2)-(h3)-(h4) hold and that either (h5.a) or (h5.b) holds. Then for all* $\alpha \in D^*$,

$$\frac{1}{n}\big|\boldsymbol{x}\cdot\boldsymbol{X}^*\big| = \frac{1}{n}\Big|\sum_{i=1}^{n} x_i X_i^*\Big| \xrightarrow[n\to\infty]{} q^*(\alpha)\,, \qquad \textit{in probability}, \tag{35}$$

where $\boldsymbol{x} = (x_1,\dots,x_n)$ *is sampled from the posterior distribution of the signal* $P(\cdot\,|\,\mathbf{Y},\mathbf{\Phi})$ *given by (5), independently of everything else.*

Theorem 2 is proved in Sec. 5.3. Notice that in all generality it is only possible to estimate $\mathbf{X}^*$ up to its sign (think for instance to $\mathbf{Y} = |\mathbf{\Phi}\mathbf{X}^*|/\sqrt{n} + \sqrt{\Delta}\mathbf{Z}$), this is why the absolute values in (35) are needed. For this reason, the usual MSE on $\mathbf{X}^*$

$$\mathrm{mse}(\widehat{\mathbf{X}}) := \frac{1}{n}\mathbb{E}\Big[\big\|\mathbf{X}^* - \widehat{\mathbf{X}}(\mathbf{Y},\mathbf{\Phi})\big\|^2\Big]$$

is not (in all generality) an appropriate error metric. Indeed, in the case where $\mathbf{Y} = |\mathbf{\Phi}\mathbf{X}^*|/\sqrt{n} + \sqrt{\Delta}\mathbf{Z}$, where $\mathbf{\Phi},\mathbf{X}^*,\mathbf{Z}$ have all independent $\mathcal{N}(0,1)$ entries, then $\mathbb{E}[\mathbf{X}^*|\mathbf{Y},\mathbf{\Phi}] = 0$ and $\min_{\widehat{\mathbf{X}}}\mathrm{mse}(\widehat{\mathbf{X}}) = 1$. This means that the minimum mean-square error is always equal to the variance and thus, in this sense, it is never possible to estimate the signal better than trivial estimators. For this reason, the appropriate error metric for the reconstruction problem is the MSE on $\mathbf{X}^*\mathbf{X}^{*\intercal}$. From Theorem 2 one deduces the limit of the MMSE in estimating $\mathbf{X}^*\mathbf{X}^{*\intercal}$:

Corollary 3 (Matrix minimum mean-square error). *Under the same conditions as in Theorem 2, for all* $\alpha \in D^*$ *we have*

$$\mathrm{MMSE}_n := \frac{1}{n^2}\mathbb{E}\Big[\big\|\boldsymbol{X}^*\boldsymbol{X}^{*\intercal} - \mathbb{E}[\boldsymbol{X}^*\boldsymbol{X}^{*\intercal}|\mathbf{Y},\mathbf{\Phi}]\big\|_{\mathrm{F}}^2\Big] \xrightarrow[n\to\infty]{} \rho^2 - q^*(\alpha)^2\,, \tag{36}$$

where $\|-\|_{\mathrm{F}}$ *denotes the Frobenius norm.*

2.3 Optimal generalization (or prediction) error

In order to express the optimal generalization error we introduce the following function (recall that $\widetilde{Y}_0$ is drawn from the channel (20)):

$$\mathcal{E}(q) := \mathrm{MMSE}(\widetilde{Y}_0|V) = \mathbb{E}\big[\big(\widetilde{Y}_0 - \mathbb{E}[\widetilde{Y}_0|V]\big)^2\big] \tag{37}$$

$$= \mathbb{E}_V \int dY\, Y^2 P_{\mathrm{out}}(Y|\sqrt{\rho}\, V) - \mathbb{E}_V\Big[\mathbb{E}_W\Big[\int dY\, Y P_{\mathrm{out}}(Y|\sqrt{q}\, V + \sqrt{\rho - q}\, W)\Big]^2\Big] \tag{38}$$

$$= \mathbb{E}\big[\varphi(\sqrt{\rho}\, V, \mathbf{A})^2\big] - \mathbb{E}_V\big[\mathbb{E}_{W,\mathbf{A}}\big[\varphi(\sqrt{q}\, V + \sqrt{\rho - q}\, W, \mathbf{A})\big]^2\big] + \Delta \tag{39}$$

where $V, W \overset{\text{iid}}{\sim} \mathcal{N}(0,1)$, $\mathbf{A} \sim P_A$ are independent random variables, $\mathbb{E}_{W,\mathbf{A}}$ denotes the expectation w.r.t. W and $\mathbf{A}$ only and $\mathbb{E}_W[-]^2 = (\mathbb{E}_W[-])^2$. We recall $\rho := \mathbb{E}[(X^*)^2]$ with $X^* \sim P_0$. With a slight abuse of notation $\int dY$ denotes in (38) either the integration w.r.t. Lebesgue's measure on $\mathbb{R}$ in the case $\Delta > 0$ or the integration w.r.t. the counting measure on $\mathbb{N}$ (in the case $\Delta = 0$).

Recall the teacher-student setting of Sec. 1.2: The generalization error is related to the estimation of a new output $Y_{\mathrm{new}} \sim P_{\mathrm{out}}(\cdot \,|\, \mathbf{\Phi}_{\mathrm{new}} \cdot \mathbf{X}^*/\sqrt{n})$ where $\mathbf{\Phi}_{\mathrm{new}}$ is a new row of the matrix, and is defined by (13).

Theorem 3 (Optimal generalization error)**.** *Assume that P_{out} is informative, that (h1)-(h2)-(h3)-(h4) hold and that either (h5.a) or (h5.b) hold. Then for all $\alpha \in D^*$ we have*

$$\mathcal{E}_{\mathrm{gen}}^{\mathrm{opt}}(\alpha) \xrightarrow[n\to\infty]{} \mathcal{E}(q^*(\alpha)) \tag{40}$$

where $q^*(\alpha)$ is the optimizer of the replica-symmetric formula (24), see Proposition 1.

Theorem 3 follows from a more general result, that we state now. Let $f : \mathbb{R} \to \mathbb{R}$ and consider the generalized optimal generalization error

$$\mathcal{E}_{f,n}(\alpha) := \mathrm{MMSE}(f(Y_{\mathrm{new}})|\mathbf{\Phi}_{\mathrm{new}}, \mathbf{Y}, \mathbf{\Phi}) = \mathbb{E}\big[\big(f(Y_{\mathrm{new}}) - \mathbb{E}[f(Y_{\mathrm{new}})|\mathbf{\Phi}_{\mathrm{new}}, \mathbf{Y}, \mathbf{\Phi}]\big)^2\big] \tag{41}$$

which is the minimum mean-square error on $f(Y_{\mathrm{new}})$. In particular $\mathcal{E}_{\mathrm{gen}}^{\mathrm{opt}}(\alpha) = \mathcal{E}_{f,n}(\alpha)$ for $f : x \mapsto x$. We define also

$$\mathcal{E}_f(q) := \mathrm{MMSE}(f(\widetilde{Y}_0)|V) = \mathbb{E}\big[\big(f(\widetilde{Y}_0) - \mathbb{E}[f(\widetilde{Y}_0)|V]\big)^2\big] \tag{42}$$

$$= \mathbb{E}\big[f\big(\varphi(\sqrt{\rho}\, V, \mathbf{A}) + \sqrt{\Delta} Z\big)^2\big] - \mathbb{E}_V\big[\mathbb{E}_{W,Z,\mathbf{A}}\big[f\big(\varphi(\sqrt{q}\, V + \sqrt{\rho - q}\, W, \mathbf{A}) + \sqrt{\Delta} Z\big)\big]^2\big], \tag{43}$$

where $\widetilde{Y}_0$ is the output of the second scalar channel (20).

Theorem 4 (Generalized optimal generalization error)**.** *Let $f : \mathbb{R} \to \mathbb{R}$ be a measurable function such that $\mathbb{E}[|f(Y_1)|^{2+\gamma}]$ remains bounded as n grows, for some $\gamma > 0$. Assume that P_{out} is informative and that (h1)-(h2)-(h3)-(h4) hold and that either (h5.a) or (h5.b) holds. Then for all $\alpha \in D^*$ we have*

$$\mathcal{E}_{f,n}(\alpha) \xrightarrow[n\to\infty]{} \mathcal{E}_f(q^*(\alpha)) \tag{44}$$

where $q^*(\alpha)$ is the optimizer of the replica-symmetric formula (24), see Proposition 1.

Theorem 4 is proved in Sec. 5.1.

2.4 Optimality of the generalized approximate message-passing algorithm

2.4.1 The generalized approximate message-passing algorithm

While the main results presented until now are information-theoretic, our next one concerns the performance of a popular algorithm to solve random instances of generalized linear problems, called generalized approximate message-passing (GAMP). We shall not re-derive its properties here, and instead refer to the original papers for details. This approach has a long history, especially in statistical physics [6–9], error correcting codes [10], and graphical models [11]. For a modern derivation in the context of linear models, see [12–14]. The case of generalized linear models was discussed by Rangan in [15], and has been used for classification purpose in [16].

We first need to define two so-called threshold functions that are associated to the two scalar channels (17) and (20). The first one is the posterior mean of the signal in channel (17) with signal-to-noise ratio r:

$$g_{P_0}(y,r) := \mathbb{E}[X_0|Y_0 = y]\,. \tag{45}$$

The second one is the posterior mean of W^* in channel (20) with "noise level" $\eta = \rho - q$:

$$g_{P_{\rm out}}(\widetilde{y}, v, \eta) := \mathbb{E}[W^*|\widetilde{Y}_0 = \widetilde{y}, \sqrt{q}\,V = v]\,. \tag{46}$$

These functions act componentwise when applied to vectors.

Given initial estimates $(\widehat{\mathbf{x}}^0, \mathbf{v}^0)$ for the means and variances of the elements of the signal vector $\mathbf{X}^*$, GAMP takes as input the observation vector $\mathbf{Y}$ and then iterates the following equations with initialization $g^0_\mu = 0$ for all $\mu = 1, \dots, m$ (we denote by $\overline{\mathbf{u}}$ the average over all the components of the vector $\mathbf{u}$ and $\mathbf{\Phi}^\intercal$ is the transpose of the matrix $\mathbf{\Phi}$): From $t = 1$ until convergence,

$$\begin{cases} V^t &= \overline{\mathbf{v}^{t-1}} \\ \boldsymbol{\omega}^t &= \mathbf{\Phi}\widehat{\mathbf{x}}^{t-1}/\sqrt{n} - V^t\mathbf{g}^{t-1} \\ g^t_\mu &= g_{P_{\rm out}}(Y_\mu, \omega^t_\mu, V^t) \qquad \forall\, \mu = 1,\dots m \\ \lambda^t &= \alpha\, \overline{g^2_{P_{\rm out}}(\mathbf{Y}, \boldsymbol{\omega}^t, V^t)} \\ \mathbf{R}^t &= \widehat{\mathbf{x}}^{t-1} + (\lambda^t)^{-1}\mathbf{\Phi}^\intercal\mathbf{g}^t/\sqrt{n} \\ \widehat{x}^t_i &= g_{P_0}(R^t_i, \lambda^t) \qquad \forall\, i = 1,\dots n \\ \mathrm{v}^t_i &= (\lambda^t)^{-1}\,\partial_R g_{P_0}(R,\lambda^t)|_{R=R^t_i} \qquad \forall\, i = 1,\dots n \end{cases} \tag{47}$$

One of the strongest asset of GAMP is that its performance can be tracked rigorously in the limit $n, m \to \infty$ while $m/n \to \alpha$ via a procedure known as state evolution (SE), see [17, 18] for the linear case, and [15, 19] for the generalized one. In our notations, state evolution tracks the asymptotic value of the overlap between the true hidden value $\mathbf{X}^*$ and its estimate by GAMP $\widehat{\mathbf{x}}^t$ defined as $q^t := \lim_{n\to\infty} \mathbf{X}^* \cdot \widehat{\mathbf{x}}^t/n$ (that is related to the asymptotic mean-square error (MSE) E^t between $\mathbf{X}^*$ and its estimate $\widehat{\mathbf{x}}^t$ by $E^t = \rho - q^t$, where recall that $\rho := \mathbb{E}[(X^*)^2]$ with $X^* \sim P_0$) via:

$$\begin{cases} q^{t+1} &= 2\psi'_{P_0}(r^t)\,, \\ r^t &= 2\alpha\Psi'_{P_{\rm out}}(q^t;\rho)\,. \end{cases} \tag{48}$$

From Theorem 1 we realize that the fixed points of these equations correspond to the critical points of the asymptotic free entropy in (24). In fact, in the replica heuristic, the optimizer q^* of the potential is conjectured to give the optimal value of the overlap, a fact that was proven for the linear channel [20–22]. We will see in Sec. 3 that $q^t \xrightarrow[t\to\infty]{} q^*$ for a large set of parameters.

2.4.2 Estimation and generalization error of GAMP

Perhaps more surprisingly, one can use GAMP in the teacher-student scenario described in Sec. 1.2 in order to provide an estimation of a new output $Y_{\text{new}} \sim P_{\text{out}}(\cdot \,|\, \mathbf{\Phi}_{\text{new}} \cdot \mathbf{X}^* / \sqrt{n})$ where $\mathbf{\Phi}_{\text{new}}$ is a new row of the matrix. As $\widehat{\mathbf{x}}^t$ is the GAMP estimate of the posterior expectation of $\mathbf{X}^*$, with estimated variance $\mathbf{v}^t$, the natural heuristic is to consider for the posterior probability distribution of the random variable $\mathbf{\Phi}_{\text{new}} \cdot \mathbf{X}^* / \sqrt{n}$ a Gaussian with mean $\mathbf{\Phi}_{\text{new}} \cdot \widehat{\mathbf{x}}^{t-1} / \sqrt{n}$ and variance $V^t = E^t = \rho - q^t$ (the fact that the variance and MSE are equal follows from the Nishimori identity of Proposition 12 but applied to GAMP instead of the Gibbs measure, see e.g. [23] where this is shown). This allows to estimate the posterior mean of the output, which leads to the GAMP prediction (recall the P_{out} definition (3)-(4)):

$$\widehat{Y}^{\text{GAMP},t} := \int y \, P_{\text{out}}\Big(y \,\Big|\, \frac{1}{\sqrt{n}} \mathbf{\Phi}_{\text{new}} \cdot \widehat{\mathbf{x}}^{t-1} + \sqrt{\rho - q^t}\, w\Big) \mathcal{D}w dy \,, \tag{49}$$

where $\mathcal{D}w$ denotes the standard Gaussian measure. The following claim, from [15], gives the precise estimation error of GAMP. It is stated there as a claim because some steps of the proof are missing. The paper [19] affirms in its abstract to prove the claim of [15], but without further details. For these reasons, we believe that the claim holds, however we prefer to state it here as a claim (instead of a theorem).

Claim 1 (GAMP estimation error, [15])**.** *We have almost surely for all $t \in \mathbb{N}$,*

$$\lim_{n\to\infty} \frac{1}{n} \widehat{\boldsymbol{x}}^t \cdot \boldsymbol{X}^* = \lim_{n\to\infty} \frac{1}{n} \|\widehat{\boldsymbol{x}}^t\|^2 = q^t \,, \tag{50}$$

as well as

$$\lim_{n\to\infty} \frac{1}{n^2} \mathbb{E}\Big[\big\|\boldsymbol{X}^*\boldsymbol{X}^{*\intercal} - \widehat{\boldsymbol{x}}^t(\widehat{\boldsymbol{x}}^t)^\intercal\big\|^2\Big] = \rho^2 - (q^t)^2 \,. \tag{51}$$

Compairing (51) with the MMSE given by Corollary 4, we see that if $\lim_{t\to\infty} q^t = q^*(\alpha)$, then GAMP achieves the MMSE. Provided that Claim 1 holds we can deduce the generalization error of GAMP:

Proposition 2 (GAMP generalization error)**.** *Suppose that hypotheses (h1)-(h2)-(h4) hold. Moreover suppose that either (h5.a) or (h5.b) holds. Assume that $(\Phi_{\mu i}) \overset{iid}{\sim} \mathcal{N}(0,1)$, and that $x \mapsto P_{\text{out}}(\cdot|x)$ is continuous almost everywhere for the Wasserstein distance of order 2. Let $t \in \mathbb{N}$. Assume that the limit (50) holds in probability and that there exists $\eta > 0$ such that $\mathbb{E}[|\widehat{Y}^{\text{GAMP},t}|^{2+\eta}]$ remains bounded (as n grows). Then we have*

$$\lim_{n\to\infty} \mathcal{E}_{\text{gen}}^{\text{GAMP},t} := \lim_{n\to\infty} \mathbb{E}\big[\big(Y_{\text{new}} - \widehat{Y}^{\text{GAMP},t}\big)^2\big] = \mathcal{E}(q^t) \,. \tag{52}$$

Remark 2. *If we modify slightly the GAMP estimator of (49) by changing the first y into $f(y)$, it is not difficult to show (following the steps of Proposition 2) that this new estimator achieves an asymptotic error of $\mathcal{E}_f(q^t)$, given by (42), for estimating $f(Y_{\text{new}})$.*

Proposition 2 is proved in Sec. 5.2. We see that this formula matches the one for the Bayes-optimal generalization error, see Theorem 3, up to the fact that instead of $q^*(\alpha)$ (the optimizer of the replica formula (24)) appearing in the optimal error formula, here it is q^t which appears. Thus clearly, when q^t converges to $q^*(\alpha)$ (we shall see that this is the case in many situations in the examples of Sec. 3) this yields a very interesting and non trivial result: *GAMP achieves the Bayes-optimal generalization error* in a plethora of models (a task again often believed to be intractable) and this for large sets of parameters.

2.5 Optimal denoising error

Another interesting error measure to study is the following "denoising error". Assume that the observations are noisy, i.e. $\Delta > 0$ in (1). The goal here is to denoise the observations Y_μ and estimate the signal which in this case is $\varphi\big(\frac{1}{\sqrt{n}}[\mathbf{\Phi}\mathbf{X}^*]_\mu, \mathbf{A}_\mu\big)$.

The minimum denoising error (in L^2 sense) is actually a simple corollary from the replica-symmetric formula of Theorem 1 and follows from a so-called "I-MMSE relation", see Proposition 13. We will need the joint posterior distribution of $(W^*, \mathbf{A})$ given $(V, \widetilde{Y}_0)$ for the scalar channel (20). So we define the Gibbs bracket for the scalar channel by (here $\mathbf{a} \in \mathbb{R}^{k_A}$):

$$\langle g(w, \mathbf{a}) \rangle_{\rm sc} := \mathbb{E}[g(W^*, \mathbf{A}) | \widetilde{Y}_0, V] = \frac{\int \mathcal{D}w dP_A(\mathbf{a}) g(w, \mathbf{a}) e^{-\frac{1}{2\Delta}\left\{\widetilde{Y}_0 - \varphi(\sqrt{q}\, V + \sqrt{\rho - q}\, w, \mathbf{a})\right\}^2}}{\int \mathcal{D}w dP_A(\mathbf{a}) e^{-\frac{1}{2\Delta}\left\{\widetilde{Y}_0 - \varphi(\sqrt{q}\, V + \sqrt{\rho - q}\, w, \mathbf{a})\right\}^2}}\,, \tag{53}$$

for any continuous bounded function g. When the function depends only on w it may be re-written as

$$\langle g(w) \rangle_{\rm sc} = \frac{\int \mathcal{D}w g(w) P_{\rm out}\big(\widetilde{Y}_0 \big| \sqrt{q}\, V + \sqrt{\rho - q}\, w\big)}{\int \mathcal{D}w P_{\rm out}\big(\widetilde{Y}_0 \big| \sqrt{q}\, V + \sqrt{\rho - q}\, w\big)}\,. \tag{54}$$

Corollary 4 (Optimal denoising error). *Suppose that hypotheses (h1)-(h2)-(h3)-(h4) hold. Suppose that either hypothesis (h5.a) or (h5.b) holds. Then for almost every $\Delta > 0$, for any optimal couple (q^*, r^*) of (24),*

$$\begin{aligned}\lim_{n\to\infty} \frac{1}{m} \mathrm{MMSE}\Big(\varphi\Big(\frac{1}{\sqrt{n}} \boldsymbol{\Phi} \boldsymbol{X}^*, \boldsymbol{A}\Big) \Big| \boldsymbol{\Phi}, \boldsymbol{Y}\Big) &= \mathrm{MMSE}\big(\varphi(\sqrt{q^*}\, V + \sqrt{\rho - q^*}\, W^*, \mathbf{A}) \big| \widetilde{Y}_0, V\big) \\ &= \mathbb{E}\big[\varphi(\sqrt{\rho}\, V, \boldsymbol{A})^2\big] - \mathbb{E}\big[\langle \varphi(\sqrt{q^*}\, V + \sqrt{\rho - q^*}\, w, \boldsymbol{a}) \rangle_{\rm sc}^2\big]\,, \end{aligned} \tag{55}$$

where $\langle - \rangle_{\rm sc}$ acts jointly on $(w, \boldsymbol{a})$ and is defined by (53), and $V, W^* \overset{\rm iid}{\sim} \mathcal{N}(0,1)$.

Note that the joint posterior over both the signal $\mathbf{X}^*$ and the random stream $\mathbf{A}$ is simply expressed as

$$dP(\mathbf{x}, \mathbf{a} | \mathbf{Y}, \boldsymbol{\Phi}) \propto dP_0(\mathbf{x}) dP_A(\mathbf{a}) \prod_{\mu=1}^{m} e^{-\frac{1}{2\Delta}\left\{Y_\mu - \varphi\left(\frac{1}{\sqrt{n}}[\boldsymbol{\Phi}\mathbf{x}]_\mu, \mathbf{a}_\mu\right)\right\}^2}\,. \tag{56}$$

The proof of Corollary 4 is presented in Sec. 5.4.

3 Application to concrete situations

In this section, we show how our main results can be applied to several models of interest in fields ranging from machine learning to signal processing, and unveil several interesting new phenomena in learning of generalized linear models. For various specific cases of prior P_0 and output $P_{\rm out}$, we evaluate numerically the free entropy potential (22), its stationary points Γ and identify which of them gives the information-theoretic results, i.e. is the optimizer in (24). We also identify which of the stationary points corresponds to the result obtained asymptotically by the GAMP algorithm, i.e. the fixed point of the state evolution (48). Finally we compute the corresponding generalization error (52). We stress that in this section the results are based on numerical investigation of the resulting formulas: We do not aim at rigor that would involve precise bounds and more detailed analytical control for the corresponding integrals.

3.1 Generic observations

Using the functions $g_{P_{\rm out}}$ and g_{P_0} introduced in Sec. 2.4 we can rewrite the fixed point equations (23) as

$$q = 2\psi'_{P_0}(r) = \mathbb{E}[g_{P_0}(Y_0, r)^2]\,, \tag{57}$$

$$r = 2\alpha \Psi'_{P_{\rm out}}(q) = \frac{\alpha}{\rho - q} \mathbb{E}[g_{P_{\rm out}}(\widetilde{Y}_0, \sqrt{q}\, V, \rho - q)^2]\,, \tag{58}$$

where the expectation in (57) corresponds to the scalar channel (17) and the expectation in (58) corresponds to the second scalar channel (20).

Non-informative fixed point and its stability: It is interesting to analyze under what conditions $q^* = 0$ is the optimizer of (24). Notice that $q^* = 0$ corresponds to the error on the recovery of the signal as large as it would be if we had no observations at our disposition. Theorem 1 gives that any optimal couple (q^*, r^*) of (24) should be a fixed point of the state evolution equations (57)–(58). A sufficient condition for $(q, r) = (0, 0)$ to be a fixed point of (57)–(58) is that:

(a) The transition density $P_{\rm out}(y|z)$ is even in the argument z.

(b) The prior P_0 has zero mean.

In order to see this, notice that if $P_{\rm out}(y|z)$ is even in z then from the definition (46) of the function $g_{P_{\rm out}}$ we have $g_{P_{\rm out}}(y, 0, \rho) = 0$ and consequently from (58) we have $\Psi'_{P_{\rm out}}(0) = 0$. From the second point, notice that we have $\psi'_{P_0}(0) = \frac{1}{2}\mathbb{E}_{P_0}[X_0]^2 = 0$.

We assume now that the transition density $P_{\rm out}(y|z)$ is even in the argument z and that the prior P_0 has zero mean. In order for $q = 0$ to be the global maximizer q^* of (24) or to be a relevant fixed point of the state evolution (48) (relevant in the sense that GAMP might indeed converge to it in a practical setting) we need $q = 0$ to be a stable fixed point of the above equations (57)–(58). We therefore need to expand (57)–(58) around $q = 0$, and doing so, we obtain that $q = 0$ is stable if

$$2\alpha\Psi''_{P_{\rm out}}(0) \times 2\psi''_{P_0}(0) = \alpha\mathbb{E}\big[\big(\langle w^2\rangle_{\rm sc} - \langle w\rangle_{\rm sc}^2 - 1\big)^2\big] < 1\,, \qquad (59)$$

where the expectation corresponds to the scalar channel (20) with $q = 0$ and the Gibbs bracket $\langle - \rangle_{\rm sc}$ is given by (53). The expectation quantifies how the observation of $\widetilde{Y}_0$ in the scalar channel (20) modifies the variance of W^* (which is 1 without any observation). Rewriting this condition more explicitly into a form that is convenient for numerical evaluation we get (recalling (54), $q = 0$ and condition (a))

$$\alpha \int dy \frac{\big(\int \mathcal{D}z(z^2-1)P_{\rm out}(y|\sqrt{\rho}z)\big)^2}{\int \mathcal{D}zP_{\rm out}(y|\sqrt{\rho}z)} < 1\,, \qquad (60)$$

where recall that $\mathcal{D}z$ is a standard Gaussian measure. We conjecture that the condition (60) delimits precisely the region where polynomial-time algorithms do not perform better than “random guessing” (see the discussion below, where we will make this stability condition explicit for several examples of symmetric output channels). Note that the condition (60) also appears in a recent work [24] as a barrier for performance of spectral algorithms.

Exact recovery fixed point: Another particular fixed point of (57)–(58) that we observe is the one corresponding to exact recovery $q^* = \rho$. A sufficient and necessary condition for this to be a fixed point is that $\lim_{q\to\rho}\Psi'_{P_{\rm out}}(q) = +\infty$. Heuristically, this means that the integral of the Fisher information of the output channel should diverge:

$$\int dyd\omega \frac{e^{-\frac{\omega^2}{2\rho}}}{\sqrt{2\pi\rho}} \frac{P'_{\rm out}(y|\omega)^2}{P_{\rm out}(y|\omega)} = +\infty\,, \qquad (61)$$

where $P'_{\rm out}(y|\omega)$ denotes the partial derivative w.r.t. ω. This typically means that the channel should be noiseless. For the Gaussian channel with noise variance Δ, the above expression equals $1/\Delta$. For the probit channel where $P_{\rm out}(y|z) = {\rm erfc}(-yz/\sqrt{2\Delta})/2$ the above expression at small Δ is proportional to $1/\sqrt{\Delta}$.

Stability of the exact recovery fixed point was also investigated, but we did not obtain any unified expression. The stability depends non-trivially on both the properties of the output channel, but also on the

properties of the prior. Below we give several examples where exact recovery either is or is not possible, or where there is a phase transition between the two regimes.

3.2 Phase diagram of perfect learning

In this section we consider *deterministic (noiseless) output channels* and ask: How many measurements are needed in order to perfectly recover the signal?

Our crucial point is to compare with the well explored phase diagram of Bayesian (noiseless) compressed sensing in the case of the linear channel [13, 25]. As the number of samples (measurements) varies we encounter five different regimes of parameters:

- The *tractable recovery* phase: This is the region in the parameter space where GAMP achieves perfect reconstruction.
- The *non-informative* phase: Region where perfect reconstruction is information-theoretically impossible and moreover even the Bayes-optimal estimator is as bad as a random guess based on the prior information and on the knowledge of the output function.
- The *no recovery* phase: Region where perfect reconstruction is information-theoretically impossible, but an estimator positively correlated with the ground truth exists.
- The *hard* phase: Region where the perfect reconstruction is information-theoretically possible, but where GAMP is unable to achieve it. At the same time, in this region GAMP leads to a better generalization error than the one corresponding to the non-informative fixed point. It remains a challenging open question whether polynomial-time algorithms can achieve perfect reconstruction in this regime.
- The *hard non-informative* phase: This phase corresponds to the region where perfect reconstruction is information-theoretically possible but where GAMP only achieves an error as bad as randomly guessing, given by the trivial fixed point. In this phase as well, the existence of polynomial-time exact recovery algorithms is an open question. This phase does not exist for the linear channel.

Some of the codes used in this section can be consulted online on the github repository [26].

3.2.1 The linear channel

The case of exact recovery of a sparse signal after it passed trough a noiseless linear channel, i.e. $\varphi(x) = x$, is studied in the literature in great details, especially in the context of compressed sensing [12, 27]. For a signal with a fraction ρ of non-zero entries it is found that as soon as $\alpha > \rho$, perfect reconstruction is theoretically possible, although it may remain computationally difficult. The whole field of compressed sensing builds on the realization that, using the ℓ_1 norm minimization technique, one can efficiently recover the signal for larger α values than the so-called Donoho-Tanner transition [12, 28].

In the context of the present paper, when the empirical distribution of the signal is known, one can fairly easily beat the ℓ_1 transition and reconstruct the signal up to lower values of α using the Bayesian GAMP algorithm [12, 13, 15, 25]. In this case, three different phases are present [13, 25]: $i)$ For $\alpha < \rho$, perfect reconstruction is impossible; $ii)$ for $\rho < \alpha < \alpha_s$ reconstruction is possible, but not with any known polynomial-complexity algorithm; $iii)$ for $\alpha > \alpha_s$, the so-called *spinodal transition* computed with state evolution, GAMP provides a polynomial-complexity algorithm able to reach perfect reconstruction. The line $\alpha_s(\rho)$ depends on the distribution of the signal. For a Gauss-Bernoulli signal with a fraction ρ of non-zero (Gaussian) values we compare the GAMP performance to the optimal one in Fig. 1 (left and right). This is the same figure as in the main text. We copy it here so that the SI is self-contained.

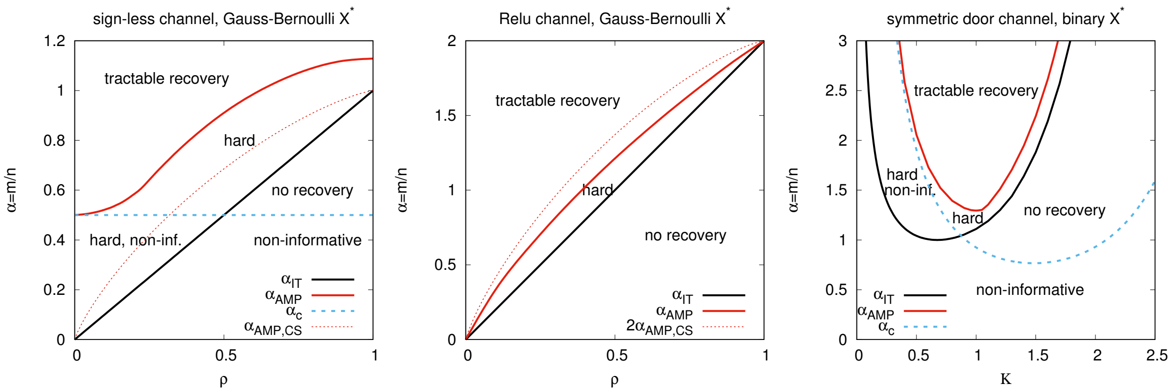

Figure 1: Phase diagrams showing boundaries of the region where exact recovery is possible (in absence of noise). **Left:** The case of sign-less sparse recovery, $\varphi(x) = |x|$ with a Gauss-Bernoulli signal, as a function of the ratio between number of samples/measurements and the dimension $\alpha = m/n$, and the fraction of non-zero components ρ. Evaluating the free entropy for this case, we find that a recovery of the signal is information-theoretically impossible for $\alpha < \alpha_{\rm IT} = \rho$. Recovery becomes possible starting from $\alpha > \rho$, just as in the canonical compressed sensing. Algorithmically the sign-less case is much harder. Evaluating (60) we conclude that GAMP is not able to perform better than a random guess as long as $\alpha < \alpha_c = 1/2$. For larger values of α, the inference using GAMP leads to better results than a purely random guess. GAMP can exactly recover the signal and generalize perfectly only for values of α larger than $\alpha_{\rm AMP}$ (full red line). The dotted red line shows for comparison the algorithmic phase transition of the canonical compressed sensing. **Center:** Analogous to the left panel, for the ReLU output function, $\varphi(x) = \max(0, x)$. Here it is always possible to perform better than random guessing using GAMP. The dotted red line shows the algorithmic phase transition when using information only about the non-zero observations. **Right:** Phase diagram for the symmetric door output function $\varphi(z) = {\rm sign}(|z| - K)$ for a Rademacher signal, as a function of α and K. The stability line α_c is depicted in dashed blue, the information-theoretic phase transition to exact recovery $\alpha_{\rm IT}$ in black, and the algorithmic one $\alpha_{\rm AMP}$ in red.

3.2.2 The rectified linear unit (ReLU) channel

Let us start by discussing the case of a generalized linear model with the ReLU output channel, i.e. $\varphi(x) = \max(0, x)$, with a signal coming from a Gauss-Bernoulli distribution $P_0 = \rho\mathcal{N}(0,1) + (1-\rho)\delta_0$ with a fraction ρ of non-zero (Gaussian) values. We are motivated by the omnipresent use of the ReLU activation function in deep learning, and explore its properties for GLMs that can be seen as a simple single layer neural network.

Our analysis shows that a perfect generalization (and thus a perfect reconstruction of the signal as well) is possible whenever the number of samples per dimension (measurement rate) $\alpha > 2\rho$, and impossible when $\alpha < 2\rho$. This is very intuitive, since half of the measurements (those non-zero) are giving as much information as in the linear case, thus the factor 2.

How hard is it to actually solve the problem with an efficient algorithm? The answer is given by applying the state evolution analysis to GAMP, which tells us that only for even larger values of α, beyond the spinodal transition, does GAMP reach a perfect recovery. Notice, however, that this spinodal transition occurs at a significantly lower measurement rate α than one would reach just keeping the non-zero measurements. This shows that, actually, these zero measurements contain a useful information for the algorithm. The situation is shown in the center panel of Fig. 1: The zero measurements do not help information-theoretically but they, however, do help algorithmically.

3.2.3 The sign-less channel

We now discuss the sign-less channel where only the absolute value of the linear mixture is observed, i.e. $\varphi(x) = |x|$. This case can be seen as the real-valued analog of the famous phase retrieval problem. We again

consider the signal to come from a Gauss-Bernoulli distribution with a fraction ρ of non-zero (Gaussian) values.

Sparse phase retrieval has been well explored in the literature in the regime where the number s of non-zeros is sub-leading in the dimension, $s = o(n)$. This case is known to present a large algorithmic gap. While analogously to compressed sensing exact recovery is information-theoretically possible for a number of measurement $\Omega(s\ln(n/s))$, best known algorithms achieve it only with $\Omega(s^2/\ln n)$ measurements [29], see also [30] and references therein for a good discussion of other related literature. This is sometimes referred to as the s^2 barrier. We are not aware of a study where, as in our setting, the sparsity is $s = \rho n$ and the number of measurements is αn with $\alpha = \Omega(1)$. Our analysis in this regime hence sheds a new light on the hardness of the problem of recovering a sparse signal from sign-less measurements.

Our analysis of the mutual information shows that a perfect reconstruction is information-theoretically possible as soon as $\alpha > \rho$: In other words, the problem is –information-theoretically– as easy, or as hard as the compressed sensing one. This is maybe less surprising when one thinks of the following algorithm: Try all 2^m choices of the possible signs for the m outputs, and solve a compressed sensing problem for each of them. Clearly, this should yields a perfect solution only in the case of the actual combination of signs.

Algorithmically, however, the problem is much harder than for the linear output channel. As shown in the left side of Fig. 1, for small ρ one requires a much larger fraction α of measurements in order for GAMP to recover the signal. For the linear channel the algorithmic transition $\alpha_s(\rho) \to 0$ as $\rho \to 0$, while for the sign-less channel we get $\alpha_s(\rho) \to 1/2$ as $\rho \to 0$. In other words if one looses the signs one cannot perform recovery in compressed sensing with less than $n/2$ measurements.

What we observe in this example for $\alpha < 1/2$ is in the statistical physics literature on neural networks known as retarded learning [31]. This appears in problems where the $\varphi(x)$ function is symmetric, as seen at the beginning of this section: There is always a critical point of the mutual information with an overlap value $q = 0$. For this problem, this critical point is actually "stable" (meaning that it is actually a local minimum in q in the mutual information (27)) for all $\alpha < 1/2$ independently of ρ. To see that, we have to go back to (59). For the absolute value channel, the posterior distribution of W^* given $\widetilde{Y}_0 = \sqrt{\rho}\,|W^*|$ in the second scalar channel (20) is a mean of two Dirac masses on $-W^*$ and W^*. Thus, the posterior variance is $\langle w^2\rangle_{\rm sc} - \langle w\rangle_{\rm sc}^2 = (W^*)^2$. Consequently, (59) leads to the stability condition $\alpha < 1/2$.

This has the two following implications: $i)$ In the non-informative phase, when $\alpha < 1/2$ and $\rho > \alpha$, the minimum at $q = 0$ is actually the global one. In this case, the MMSE on $\mathbf{X}^*$ and the generalization error are the ones given by using 0 as a guess for each element of $\mathbf{X}^*$; in other words, there is no useful information that one can exploit and no algorithmic approach can be better than a random guess. $ii)$ In the hard non-informative phase when $\alpha < 1/2$, GAMP initialized at random, i.e. close to the $q = 0$ fixed point, will remain there. This suggests that in this region, even if a perfect reconstruction is information-theoretically possible, it will still be very hard to beat a random guess with a tractable algorithm.

3.2.4 The symmetric door channel

The third output channel we study in detail is the symmetric door channel, where $\varphi(x) = \mathrm{sgn}(|x| - K)$. In case of channels with discrete set of outputs exact recovery is only possible when the prior is also discrete. In the present case we consider the signal to be Rademacher, where each element is chosen at random between 1 and -1, i.e. $P_0 = \frac{1}{2}\delta_{+1} + \frac{1}{2}\delta_{-1}$. This channel was studied previously using the replica method in the context of optimal data compression [32].

This output channel is in the class of symmetric channels for which overlap $q = 0$ is a fixed point. This fixed point is stable for $\alpha < \alpha_c(K)$. Exact recovery is information-theoretically possible above $\alpha_{\rm IT}(K)$ and tractable with the GAMP algorithm above the spinodal transition $\alpha_s(K)$. The values of these three transition lines are depicted in the right panel of Fig. 1.

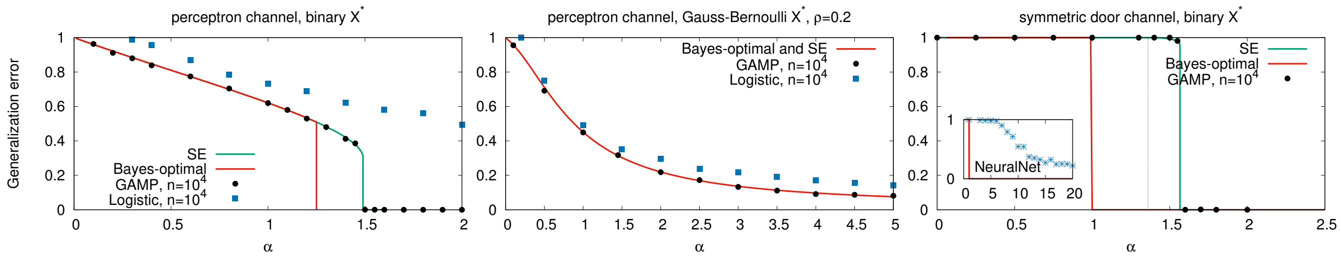

Figure 2: Generalization error in three classification problems as a function of the number of data-samples per dimension α. The red line is the Bayes-optimal generalization error, while the green one shows the (asymptotic) performances of GAMP as predicted by the state evolution (SE), when different. For comparison, we also show the result of GAMP (black dots) and, in blue, the performance of a standard out-of-the-box solver, both tested on a single randomly generated instance. **Left:** Perceptron, with $\varphi(x) = \mathrm{sgn}(x)$ and a Rademacher (± 1) signal. While a perfect generalization is information-theoretically possible starting from $\alpha = 1.249(1)$, the state evolution predicts that GAMP will allow such perfect prediction only from $\alpha = 1.493(1)$. The results of a logistic regression with fine-tuned ridge penalty with the software scikit-learn [33] are shown for comparison. **Middle:** Perceptron with Gauss-Bernoulli coefficients for the signal. No phase transition is observed in this case, but a smooth decrease of the error with α. The results of a logistic regression with fine-tuned ℓ_1 sparsity-enhancing penalty (again with [33]) are very close to optimal. **Right:** The symmetric door activation rule with parameter $K = 0.67449$ chosen in order to observe the same number of occurrence of the two classes. In this case there is a sharp phase transition at $\alpha = 1$ from a situation where it is impossible to learn the rule, so that the generalization is not better than a random guess, to a situation where the optimal generalization error drops to zero. However, GAMP identifies the rule perfectly only starting from $\alpha_s = 1.566(1)$ (GAMP error stays 1 up to $\alpha_{\mathrm{stab}} = 1.36$, see the black dashed curve). Interestingly, this non-linear rule seems very hard to learn for other existing algorithms. Using Keras [34], a neural network with two hidden layers was able to learn approximately the rule, but only for much larger training set sizes (shown in inset, the Keras/tensorflow code for this particular run can be found on the github repository [26]).

We note that $\alpha_{\mathrm{IT}} \geq 1$ is a generic bound on exact recovery for every K, required by a simple counting argument. While a-priori it is not clear whether this bound is saturated for some K, we observe that it is for $K = 0.67449$ such that half of the observed measurements are negative and the rest positive. This is, however, not efficiently achievable with GAMP. The saturation of the $\alpha_{\mathrm{IT}} \geq 1$ bound was remarked previously in the context of the work [32] on optimal data compression. Our work predicts that this information-theoretic result will not be achievable with known efficient algorithms.

3.3 Examples of optimal generalization error

Besides the formula for the mutual information, the main result of this paper is the Theorem 3 for the optimal generalization error, and formula (52) for the generalization error achieved by the GAMP algorithm. In this section we evaluate both these generalization errors for several cases of priors and output functions. We study both regression problems, where the output is real-valued, and classification problems, where the output is discrete.

While in realistic regression and classification problems the matrix $\mathbf{\Phi}$ corresponds to the data, and is thus not i.i.d. random, we view the practical interest of our theory as a benchmark for state-of-the art algorithms. Our work provides an exact asymptotic analysis of optimal generalization error and sample complexity for a range of simple rules where a teacher uses random data to generate labels. The challenge for state-of-the-art multi-purpose algorithms is to try to match as closely as possible the performance that can be obtained with GAMP that is fine-tuned to the specific form of the output and prior.

3.3.1 Threshold output: The perceptron

The example of non-linear output that is the most widely explored in the literature is the threshold output, where the deterministic output (or "activation") function is $\varphi(x) = \mathrm{sgn}(x)$. This output in the teacher-student setting of the present paper is known as the perceptron problem [3], or equivalently, the one-bit compressed sensing in signal processing [4]. Its solution has been discussed in details within the replica formalism (see for instance [5, 8, 35, 36]) and we confirm all of these heuristic computations within our approach. Let $V \sim \mathcal{N}(0,1)$. The formula (38), (44) for the generalization error then reduces to (recall $q^* = q^*(\alpha)$ is an optimizer of (24))

$$\lim_{n\to\infty} \mathcal{E}_{\rm gen}^{\rm opt} = 1 - \int \mathcal{D}V \Big(\frac{2}{\sqrt{\pi}} \int_0^{V\sqrt{\frac{q^*}{2(\rho-q^*)}}} dt\, e^{-t^2/2}\Big)^2 = 1 - \mathbb{E}\Big[\mathrm{erf}\Big(V\sqrt{\frac{q^*}{2(\rho-q^*)}}\Big)^2\Big]\,. \tag{62}$$

In Fig. 2 (left) we plot the optimal generalization error of the perceptron with a Rademacher signal, the state evolution prediction of the generalization error of the GAMP algorithm, together with the error actually achieved by GAMP on one randomly generated instance of the problem. We also compare these to the performance of a standard logistic regression. As expected from existing literature [3, 37] we confirm that in this case the information-theoretic transition appears at a number of samples per dimension $\alpha_{\rm IT} = 1.249(1)$, while the algorithmic transition is at $\alpha_s = 1.493(1)$. Logistic regression does not seem to be able to match the performance on GAMP in this case.

In Fig. 2 (center) we plot the generalization error for a Gauss-Bernoulli signal with density $\rho = 0.2$. Cases as this one were studied in detail in the context of one-bit compressed sensing [5] and GAMP was found to match the optimal generalization performance with no phase transitions observed, which is confirmed by our analysis. In this case the logistic regression is rather close to the performance of GAMP.

3.3.2 Symmetric Door

The next classification problem, i.e. discrete output rule, we study is the symmetric door function $\varphi(x) = \mathrm{sgn}(|x| - K)$. In this case the generalization error (38) becomes (here again $V \sim \mathcal{N}(0,1)$)

$$\lim_{n\to\infty} \mathcal{E}_{\rm gen}^{\rm opt} = 1 - \mathbb{E}_V\Big[\Big\{\mathrm{erf}\Big(\frac{K-\sqrt{q^*}\,V}{\sqrt{2(\rho-q^*)}}\Big) - \mathrm{erf}\Big(-\frac{K+\sqrt{q^*}\,V}{\sqrt{2(\rho-q^*)}}\Big) - 1\Big\}^2\Big]\,. \tag{63}$$

In Fig. 2 (right) we plot the generalization error for $K = 0,67449$ such that $1/2$ of the outputs are 1 and $1/2$ are -1. The symmetric door output is an example of function for which the optimal generalization error for $\alpha < \alpha_{\rm IT} = 1$ (for that specific value of K, see phase diagram in the right panel of Fig. 1) is as bad as if we were guessing randomly. The GAMP algorithm still achieves such a bad generalization until $\alpha_{\rm stab} = 1.36$, and achieves perfect generalization only for $\alpha > \alpha_s = 1.566(1)$.

Interestingly, labels created from this very simple symmetric door rule seem to be very challenging to learn for general purpose algorithms. We tried to optimize parameters of a two-layers neural network and only managed to get the performances shown in the inset of Fig. 2 (right). It is an interesting theoretical challenge whether a deeper neural network can learn this simple rule from fewer samples.

3.3.3 Linear regression

The additive white Gaussian noise (AWGN) channel, or linear regression, is defined by $\varphi(x, A) = x + \sigma A$ with $A \sim \mathcal{N}(0,1)$. This models the (noisy) linear regression problem, as well as noisy random linear estimation

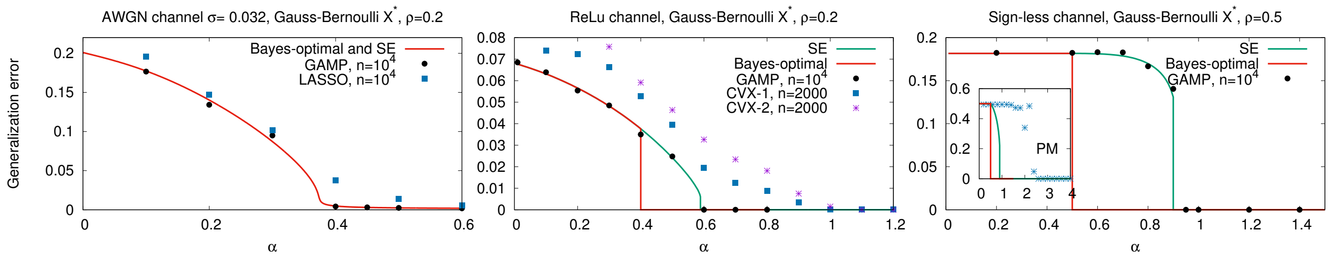

Figure 3: The generalization error for three regression problems is plotted as a function of the number of samples per dimension α. The red line is again the Bayes-optimal generalization error, while the green one shows the (asymptotic) performances of GAMP as predicted by the state evolution (SE), when different. Again, we also show the result of GAMP on a particular instance (black dots) and, in blue, the performance of an out-of-the-box solver. **Left:** White Gaussian noise output and a Gauss-Bernoulli signal. For this choice of noise, there is no sharp transition (as opposed to what happens at smaller noises). The results of a LASSO with fine-tuned ℓ_1 sparsity-enhancing penalty (with [33]) are very close to optimal. **Middle:** Here we analyze a ReLU output function $\varphi(x) = \max(0, x)$, still with a Gauss-Bernoulli signal. Now there is an information-theoretic phase transition at $\alpha = 2\rho = 0.4$, but GAMP requires $\alpha_s = 0.589(1)$ to reach perfect recovery. We show for comparison the results of maximum likelihood estimation performed with CVXPY —a powerful python-embedded language for convex optimization [38]— using two methods that are both amenable to convex optimization: In CVX-1 we use only the non-zero values of $\mathbf{Y}$, and perform a minimization of the ℓ_1 norm of $\mathbf{x}$ subject to $Y_\mu = \mathbf{\Phi}_\mu \cdot \mathbf{x}$ for $\mu \in \{1, \dots, m\}$ such that $Y_\mu \neq 0$, while in CVX-2, we use all the dataset, with the constraint that $Y_\mu = \mathbf{\Phi}_\mu \cdot \mathbf{x}$ for $\mu \in \{1, \dots, m\}$ such that $Y_\mu \neq 0$ (as before) and the additional restriction $\mathbf{\Phi}_\mu \cdot \mathbf{x} \leq 0$ for $\mu \in \{1, \dots, m\}$ such that $Y_\mu = 0$. In both case, a perfect generalization is obtained only for $\alpha \gtrapprox 1$. **Right:** The sign-less output function $\varphi(x) = |x|$. The information-theoretic perfect recovery starts at $\alpha = \rho = 0.5$, but the problem is again harder algorithmically for GAMP that succeeds only above $\alpha_s = 0.90(1)$. Again, the problem appears to be hard for other solvers. In inset, we show the performance for the estimation problem using PhaseMax [39], which is able to learn the rule only using about four times as many measurements than needed information-theoretically.

and compressed sensing. In this case (38) leads to

$$\lim_{n\to\infty} \mathcal{E}_{\rm gen}^{\rm opt} = \rho - q^* + \sigma^2 \,. \tag{64}$$

This result agrees with the generalization error analyzed heuristically in [37] in the limit $\sigma \to 0$. Fig. 3 (left) depicts the generalization error for this example. The performance of GAMP in this case is very close to the one of LASSO.

3.3.4 Rectified linear unit (ReLU)

In Fig. 3 (center) we analyze the generalization error for the ReLU output function, $\varphi(x) = \max(0, x)$. This channel models the behavior of a single neuron with the rectified linear unit activation [40] widely used in multilayer neural networks. In this case (38) becomes after simple algebra and Gaussian integration by parts (again $V \sim \mathcal{N}(0, 1)$),

$$\lim_{n\to\infty} \mathcal{E}_{\rm gen}^{\rm opt} = \frac{\rho}{2} - \frac{q^*}{4}\left(1 + \mathbb{E}_V\left[V^2 \mathrm{erf}\left(V\sqrt{\frac{q^*}{2(\rho - q^*)}}\right)^2\right]\right) - \frac{(\rho - q^*)^{3/2}}{\sqrt{\rho + q^*}}\left(\frac{1}{2\pi} + \frac{q^*}{\rho\pi}\sqrt{\frac{\rho + q^*}{\rho - q^*}}\right). \tag{65}$$

For sparse Gauss-Bernoulli signals in Fig. 3 (center) we observe again the information-theoretic transition to perfect generalization to be distinct from the algorithmic one. At the same time our test with existing algorithms were not able to closely match the performance of GAMP. This hence also remains an interesting benchmark.

3.3.5 Sign-less channel

In Fig. 3 (right) we analyze the generalization error for the sign-less output function where $\varphi(x) = |x|$. This models a situation similar to compressed sensing, except that the sign of the output has been lost. This is a real-valued analog of the phase retrieval problem as discussed in Sec. 3.2.3. In this case the generalization error (38) becomes (again $V \sim \mathcal{N}(0,1)$)

$$\lim_{n\to\infty} \mathcal{E}_{\rm gen}^{\rm opt} = \rho - \mathbb{E}_V \big[b(V\sqrt{q^*}, \rho - q^*)^2\big] \,, \tag{66}$$

where

$$b(x,y) = \sqrt{\frac{2y}{\pi}} e^{-\frac{x^2}{2y}} + \frac{x}{2}{\rm erfc}\Big(-\frac{x}{\sqrt{2y}}\Big) - \frac{x}{2}\Big\{1 + {\rm erf}\Big(-\frac{x}{\sqrt{2y}}\Big)\Big\} \,. \tag{67}$$

Our comparison with the performance of a state-of-the-art algorithm PhaseMax [39] suggests that also for this simple benchmark there is room for improvement in term of matching the performance of GAMP.

3.3.6 Sigmoid, or logistic regression

Let us also consider an output function with auxiliary randomization. After having generated the classifier $\mathbf{X}^*$, the teacher randomly associates the label $+1$ to the pattern $\mathbf{\Phi}_\mu$ with probability $f_\lambda(n^{-1/2}\mathbf{\Phi}_\mu \cdot \mathbf{X}^*)$, where $f_\lambda(x) = (1 + \exp(-\lambda x))^{-1} \in [0,1]$ is the sigmoid of parameter $\lambda > 0$, and the label -1 with probability $1 - f_\lambda(n^{-1/2}\mathbf{\Phi}_\mu \cdot \mathbf{X}^*)$. One of the (many) possible ways for the teacher to do so is by selecting $\varphi(x, A) = \mathbf{1}(A \le f_\lambda(x)) - \mathbf{1}(A > f_\lambda(x))$, where $\mathbf{1}(E)$ is the indicator function of the event E. He then generates a stream of uniform random numbers $\mathbf{A} \overset{\rm iid}{\sim} \mathcal{U}_{[0,1]}$ and obtains the labels through (1) (with $\Delta = 0$). Let $V, w \overset{\rm iid}{\sim} \mathcal{N}(0,1)$. In this setting the error (38) becomes

$$\lim_{n\to\infty} \mathcal{E}_{\rm gen}^{\rm opt} = 2 - 4\,\mathbb{E}_V\big[\big\{\mathbb{E}_w f_\lambda(\sqrt{q^*}\,V + \sqrt{\rho - q^*}\,w)\big\}^2\big] \,. \tag{68}$$

This formula reduces to (62) when $\lambda \to \infty$ as it should.

4 Proof of the replica formula by the adaptive interpolation method

We now prove Theorem 1. Our main tool will be an interpolation method recently introduced in [41] and called "adaptive interpolation method". Here we formulate the method as a direct evolution of the Guerra and Toninelli interpolation method developed in the context of spin glasses [42]. In contrast with the discrete and more pedestrian version of the adaptive interpolation method presented in [41], here we employ a continuous approach which is more straightforward (see [41] for the links between the discrete and continuous versions of the method) and that has also been recently used in [43] for studying non-symmetric tensor estimation.

We will prove Theorem 1 under the following hypotheses:

(H1) The support of the prior distribution P_0 is included in $[-S, S]$, for some $S > 0$.
(H2) φ is a bounded $\mathcal{C}^2$ function with bounded first and second derivatives w.r.t. its first argument.
(H3) $(\Phi_{\mu i}) \overset{\rm iid}{\sim} \mathcal{N}(0,1)$.

These stronger assumptions will then be relaxed in Appendix C to the weaker assumptions (h1)-(h2)-(h3)-(h4)

and (h5.a) or (h5.b). Since the observations (1) are equivalent to the rescaled observations

$$\widetilde{Y}_\mu := \Delta^{-1/2} Y_\mu = \Delta^{-1/2} \varphi\Big(\frac{1}{\sqrt{n}}[\mathbf{\Phi X}^*]_\mu, \mathbf{A}_\mu\Big) + Z_\mu \,, \qquad 1 \le \mu \le m \,, \tag{69}$$

the variance Δ of the Gaussian noise can be "incorporated" inside the function φ. Thus, it suffices to prove Theorem 1 for $\Delta = 1$ and we suppose, for the rest of the proof, that we are in this equivalent case.

4.1 Interpolating estimation problem

We introduce an "interpolating estimation problem" that interpolates between the original problem (2) at $t = 0$, $t \in [0,1]$ being the interpolation parameter, and the two scalar problems described in Sec. 1.3 at $t = 1$ which are analytically tractable. For $t \in (0,1)$ the interpolating estimation problem is a mixture of the original and scalar problems. This interpolation scheme is inspired from the interpolation paths used by Talagrand to study the perceptron, see [44]. There are two major differences between the "non-planted perceptron" studied by Talagrand, and the "planted perceptron" that we are investigating:

- In the planted case, the presence of a planted solution forces (under small perturbations) the correlations to vanish for all values of the parameters, see [45, 46]. In the non-planted case, proving such decorrelation is much more involved, and is proved only in a limited region of the parameter space, see [44].
- However, in the planted case, there can be arbitrarily many solutions to the state evolution equations (23) (see Remark 21 in [47]), whereas in the region studied by [44], there is only one solution. For this reason, our interpolation method needs to be more sophisticated in order to interpolate with the "right fixed point".

We fix a sequence $(s_n)_{n\ge1} \in (0,1/2]^{\mathbb{N}}$ that converges to 0 as n goes to infinity (s_n will be chosen in Sec. 4.3 below to be equal to $\frac{1}{2}n^{-1/16}$). We define $\mathcal{B}_n := [s_n, 2s_n]^2$. For all $\epsilon = (\epsilon_1, \epsilon_2) \in \mathcal{B}_n$, we consider two continuous "interpolation functions" $q_\epsilon : [0,1] \to [0,\rho]$ and $r_\epsilon : [0,1] \to [0, r_{\max}]$, where $r_{\max} := 2\alpha \sup_{q\in[0,\rho]} \Psi'_{P_{\rm out}}(q;\rho) = 2\alpha \Psi'_{P_{\rm out}}(\rho;\rho)$ (recall that by Proposition 18, $\Psi'_{P_{\rm out}}$ is non-decreasing). We define also for all $t \in [0,1]$ and all $\epsilon \in \mathcal{B}_n$

$$R_1(t,\epsilon) := \epsilon_1 + \int_0^t r_\epsilon(v)dv \,, \qquad R_2(t,\epsilon) := \epsilon_2 + \int_0^t q_\epsilon(v)dv \,. \tag{70}$$

We will be mainly interested in functions r_ϵ, q_ϵ that satisfy some regularity properties. We will use the following definition:

Definition 1 (Regularity). *We say that the families of functions $(q_\epsilon)_{\epsilon\in\mathcal{B}_n}$ and $(r_\epsilon)_{\epsilon\in\mathcal{B}_n}$, taking values respectively in $[0,\rho]$ and $[0, r_{\max}]$, are regular if for all $t \in [0,1]$ the mapping*

$$R^t : \left| \begin{array}{ccc} (s_n, 2s_n)^2 & \to & R^t\big((s_n,2s_n)^2\big) \\ \epsilon & \mapsto & \big(R_1(t,\epsilon), R_2(t,\epsilon)\big) \end{array} \right. \tag{71}$$

is a $\mathcal{C}^1$ diffeomorphism, whose Jacobian is greater or equal to 1.

Define

$$S_{t,\mu} := \sqrt{\frac{1-t}{n}}\,[\mathbf{\Phi X}^*]_\mu + \sqrt{R_2(t,\epsilon)}\,V_\mu + \sqrt{\rho t - R_2(t,\epsilon) + 2s_n}\,W^*_\mu \tag{72}$$

where $V_\mu, W^*_\mu \overset{\text{iid}}{\sim} \mathcal{N}(0,1)$. Consider the following observation channels, with two types of observations obtained through

$$\begin{cases} Y_{t,\mu} & \sim & P_{\rm out}(\,\cdot\,|\,S_{t,\mu})\,, & 1 \le \mu \le m, \\ Y'_{t,i} & = & \sqrt{R_1(t,\epsilon)}\,X^*_i + Z'_i\,, & 1 \le i \le n, \end{cases} \tag{73}$$

where $(Z'_i)_{i=1}^n \overset{\text{iid}}{\sim} \mathcal{N}(0,1)$. We assume that $\mathbf{V} = (V_\mu)_{\mu=1}^m$ is *known*. Then the inference problem is to recover both unknowns $\mathbf{W}^* = (W^*_\mu)_{\mu=1}^m$ and $\mathbf{X}^* = (X^*_i)_{i=1}^n$ from the knowledge of $\mathbf{V}$, $\mathbf{\Phi}$ and the "time-dependent" observations $\mathbf{Y}_t = (Y_{t,\mu})_{\mu=1}^m$ and $\mathbf{Y}'_t = (Y'_{t,i})_{i=1}^n$.

We now understand that $R_1(t,\epsilon)$ appearing in the second set of measurements in (73), and the terms $1-t$, $R_2(t,\epsilon)$ and $\rho t - R_2(t,\epsilon) + 2s_n$ appearing in the first set all play the role of signal-to-noise ratios in the interpolating model, with t giving more and more "power" (or weight) to the scalar inference channels when increasing. Here is the first crucial and novel ingredient of our interpolation scheme. In the classical interpolation method, these signal intensities would all take a trivial form (i.e. would be linear in t) but here, the non-trivial (integral) dependency in t of the intensities through the use of the interpolation functions q and r allows for much more flexibility when choosing the interpolation path. This will allow us to actually choose the "optimal interpolation path" (this will become clear soon).

Define $u_y(x) := \ln P_{\rm out}(y|x)$ and, with a slight abuse of notations,

$$s_{t,\mu} = s_{t,\mu}(\mathbf{x}, w_\mu) := \sqrt{\frac{1-t}{n}}[\mathbf{\Phi}\mathbf{x}]_\mu + \sqrt{R_2(t,\epsilon)}\,V_\mu + \sqrt{\rho t - R_2(t,\epsilon) + 2s_n}\,w_\mu\,. \tag{74}$$

We introduce the *interpolating Hamiltonian*

$$\mathcal{H}_{t,\epsilon}(\mathbf{x},\mathbf{w};\mathbf{Y}_t,\mathbf{Y}'_t,\mathbf{\Phi},\mathbf{V}) := -\sum_{\mu=1}^m \ln P_{\rm out}(Y_{t,\mu}|s_{t,\mu}) + \frac{1}{2}\sum_{i=1}^n \big(Y'_{t,i} - \sqrt{R_1(t,\epsilon)}\,x_i\big)^2. \tag{75}$$

The dependence in $\mathbf{\Phi}$ and $\mathbf{V}$ of the Hamiltonian is through the $(s_{t,\mu})_{\mu=1}^m$. It becomes, when the observations are replaced by their expression (73),

$$\mathcal{H}_{t,\epsilon}(\mathbf{x},\mathbf{w};\mathbf{Y}_t,\mathbf{Y}'_t,\mathbf{\Phi},\mathbf{V}) = -\sum_{\mu=1}^m u_{Y_{t,\mu}}(s_{t,\mu}) + \frac{1}{2}\sum_{i=1}^n \big(\sqrt{R_1(t,\epsilon)}\,(X^*_i - x_i) + Z'_i\big)^2\,. \tag{76}$$

We also introduce the corresponding Gibbs bracket $\langle - \rangle_{n,t,\epsilon}$ which is the expectation operator w.r.t. the (t,ϵ)-dependent posterior distribution of $(\mathbf{X}^*,\mathbf{W}^*)$ given $(\mathbf{Y}_t,\mathbf{Y}'_t,\mathbf{\Phi},\mathbf{V})$. It is defined as

$$\langle g(\mathbf{x},\mathbf{w})\rangle_{n,t,\epsilon} := \frac{1}{\mathcal{Z}_{t,\epsilon}(\mathbf{Y}_t,\mathbf{Y}'_t,\mathbf{\Phi},\mathbf{V})}\int dP_0(\mathbf{x})\mathcal{D}\mathbf{w}\,g(\mathbf{x},\mathbf{w})\,e^{-\mathcal{H}_{t,\epsilon}(\mathbf{x},\mathbf{w};\mathbf{Y}_t,\mathbf{Y}'_t,\mathbf{\Phi},\mathbf{V})}\,, \tag{77}$$

for every continuous bounded function g on $\mathbb{R}^n \times \mathbb{R}^m$. In (77) $\mathcal{D}\mathbf{w} = (2\pi)^{-m/2}\prod_{\mu=1}^m dw_\mu e^{-w_\mu^2/2}$ is the m-dimensional standard Gaussian distribution and $\mathcal{Z}_{t,\epsilon}(\mathbf{Y}_t,\mathbf{Y}'_t,\mathbf{\Phi},\mathbf{V})$ is the appropriate normalization (or *partition function*):

$$\mathcal{Z}_{t,\epsilon}(\mathbf{Y}_t,\mathbf{Y}'_t,\mathbf{\Phi},\mathbf{V}) := \int dP_0(\mathbf{x})\mathcal{D}\mathbf{w}\,e^{-\mathcal{H}_{t,\epsilon}(\mathbf{x},\mathbf{w};\mathbf{Y}_t,\mathbf{Y}'_t,\mathbf{\Phi},\mathbf{V})}\,. \tag{78}$$

Finally the *interpolating free entropy* is

$$f_{n,\epsilon}(t) := \frac{1}{n}\mathbb{E}\ln \mathcal{Z}_{t,\epsilon}(\mathbf{Y}_t,\mathbf{Y}'_t,\mathbf{\Phi},\mathbf{V})\,. \tag{79}$$

Note that the presence of the *perturbation* $\epsilon = (\epsilon_1, \epsilon_2)$ induces only a small change in the free entropy, namely of the order of s_n:

Lemma 1 (Small free entropy variation under perturbation)**.** *For all* $\epsilon_1, \epsilon_2 \in [s_n, 2s_n]$*,*

$$|f_{n,\epsilon}(0) - f_{n,\epsilon=(0,0)}(0)| \le C s_n \tag{80}$$

for some constant C *that only depends on* S*,* α *and* φ*.*

Proof. Let us compute (or directly obtain by the I-MMSE theorem)

$$\Big|\frac{df_{n,\epsilon}(0)}{d\epsilon_1}\Big| = \frac{1}{2}|\mathbb{E}[(X_1^*)^2] - \mathbb{E}\langle Q\rangle_{n,0,\epsilon}| \le \frac{S^2}{2}, \tag{81}$$

by hypothesis (H1). Next we compute

$$\Big|\frac{df_{n,\epsilon}(0)}{d\epsilon_2}\Big| = \frac{1}{2n}\sum_{\mu=1}^{m}\big|\mathbb{E}\big[u'_{Y_{0,\mu}}(S_{0,\mu})\langle u'_{Y_{0,\mu}}(s_{0,\mu})\rangle_{n,0,\epsilon}\big]\big|\,. \tag{82}$$

This identity is obtained using very similar steps as in Sec. A.5 to which we refer. Under hypothesis (H2) this quantity is bounded by a constant that only depends on α and φ. Then by the mean value theorem we obtain $|f_{n,\epsilon}(0) - f_{n,(0,0)}(0)| \le C\|\epsilon\| \le 2\sqrt{2}s_n C$ for some constant C that only depends on S, α and φ. □

One verifies easily, using the Lemma 1, that for all $\epsilon \in \mathcal{B}_n$

$$\begin{cases} f_{n,\epsilon}(0) &= f_{n,(0,0)}(0) + \mathcal{O}(s_n) \\ &= f_n - \frac{1}{2} + \mathcal{O}(s_n)\,, \\ f_{n,\epsilon}(1) &= \psi_{P_0}(R_1(1,\epsilon)) - \frac{1}{2}(1+\rho R_1(1,\epsilon)) + \frac{m}{n}\Psi_{P_{\rm out}}(R_2(1,\epsilon);\rho+2s_n) \\ &= \psi_{P_0}(\int_0^1 r_\epsilon(t)dt) - \frac{1}{2}(1+\rho\int_0^1 r_\epsilon(t)dt) + \frac{m}{n}\Psi_{P_{\rm out}}(\int_0^1 q_\epsilon(t)dt;\rho) + \mathcal{O}(s_n)\,. \end{cases} \tag{83}$$

where f_n is given by (11) and where $\mathcal{O}(s_n)$ denotes a quantity that is bounded by Cs_n for some constant $C > 0$ that only depends on S, φ and α. For the last equality we used Proposition 17 in Appendix B.1 which says that ψ_{P_0} is $\frac{\rho}{2}$-Lipschitz and, similarly to Proposition 18, it is not difficult to verify that $(q_1, q_2) \mapsto \Psi_{P_{\rm out}}(q_1; q_2)$ is $\mathcal{C}^1$ on the compact set $\{(q_1, q_2)\,|\,0 \le q_1 \le q_2 \le \rho+1\}$ and is thus Lipschitz. We emphasize a crucial property of the interpolating model: It is such that at $t = 0$ we recover the original model and thus $f_{n,\epsilon}(0) \approx f_n - 1/2$ (the trivial constant comes from the purely noisy measurements of the second channel in (73)), while at $t = 1$ we have the two scalar inference channels and thus the associated terms ψ_{P_0} and $\Psi_{P_{\rm out}}$ discussed in Sec. 1.3 appear in $f_{n,\epsilon}(1)$. These are precisely the terms appearing in the potential (22).

4.2 Free entropy variation along the interpolation path

From the understanding of the previous section, it is at this stage very natural to evaluate the variation of free entropy along the interpolation path, which allows to "compare" the original and purely scalar models thanks to the identity

$$f_n = f_{n,\epsilon}(0) + \frac{1}{2} + \mathcal{O}(s_n) = f_{n,\epsilon}(1) - \int_0^1 \frac{df_{n,\epsilon}(t)}{dt}dt + \frac{1}{2} + \mathcal{O}(s_n)\,, \tag{84}$$

where the first equality follows from (83). As discussed above, part of the potential (22) appears in $f_{n,\epsilon}(1)$. If the interpolation is properly done, the missing terms required to obtain the potential on the r.h.s. of (84) should naturally appear. Then by choosing the optimal interpolation path thanks to the non-trivial snr dependencies

in t (i.e. by selecting the proper interpolating functions q and r), we will be able to show the equality between the replica formula and the free entropy $\lim_{n\to\infty} f_n$.

We thus now compute the t-derivative of the free entropy along the interpolation path (see Appendix A.5 for the proof). Let $u'_y(x)$ be the derivative (w.r.t. x) of $u_y(x)$. Then we have the following.

Proposition 3 (Free entropy variation). *The derivative of the free entropy* (79) *verifies, for all $\epsilon \in \mathcal{B}_n$ and all $t \in (0,1)$*

$$\frac{df_{n,\epsilon}(t)}{dt} = -\frac{1}{2}\mathbb{E}\Big\langle\Big(\frac{1}{n}\sum_{\mu=1}^{m} u'_{Y_{t,\mu}}(S_{t,\mu})u'_{Y_{t,\mu}}(s_{t,\mu}) - r_\epsilon(t)\Big)\big(Q - q_\epsilon(t)\big)\Big\rangle_{n,t,\epsilon} + \frac{r_\epsilon(t)}{2}(q_\epsilon(t)-\rho) + \mathcal{O}_n(1)\,, \tag{85}$$

where $\mathcal{O}_n(1)$ is a quantity that goes to 0 in the $n, m \to \infty$ limit, uniformly in $t \in (0,1)$, $\epsilon \in \mathcal{B}_n$ and uniformly in the choice of the functions q_ϵ and r_ϵ. The overlap is

$$Q_n = Q := \frac{1}{n}\boldsymbol{X}^* \cdot \boldsymbol{x} = \frac{1}{n}\sum_{i=1}^{n} X_i^* x_i \tag{86}$$

where $\boldsymbol{x}$ is a sample from the posterior of model (73) *associated with the Gibbs bracket $\langle - \rangle_{n,t,\epsilon}$, see* (77).

4.3 Overlap concentration and fundamental sum rule

The next lemma plays a key role in our proof. Essentially it states that the overlap concentrates around its mean, a behavior called "replica symmetric" in statistical physics. Similar results have been obtained in the context of the analysis of spin glasses [44, 48]. Here we use a formulation taylored to Bayesian inference problems as developed in the context of LDPC codes, random linear estimation [21] and Nishimori symmetric spin glasses [46, 49, 50].

Proposition 4 (Overlap concentration). *Assume that the interpolation functions (q_ϵ), (r_ϵ) are regular, see Definition 1. Let $s_n = \frac{1}{2}n^{-1/16}$ for all $n \geq 1$. Under assumptions (H1), (H2) and (H3) there exists a constant $C(\varphi, S, \alpha)$ that depends only on S, φ and α such that*

$$\frac{1}{s_n^2}\int_{\mathcal{B}_n} d\epsilon \int_0^1 dt\, \mathbb{E}\big\langle\big(Q - \mathbb{E}\langle Q\rangle_{n,t,\epsilon}\big)^2\big\rangle_{n,t,\epsilon} \leq \frac{C(\varphi, S, \alpha)}{n^{1/8}}\,. \tag{87}$$

Proposition 4 follows from Proposition 29 proved in Appendix E.2, combined with (221) and Fubini's theorem. Note from (83) and (22) that the second term appearing in (85) is precisely the missing one that is required in order to obtain the expression of the potential on the r.h.s. of (84). Thus in order to prove Theorem 1 we would like to "cancel" the Gibbs bracket in (85), which is the so called *remainder* (once integrated over t). This is made possible thanks to the adaptive interpolating functions.

One possible way to cancel the remainder is to choose $q_\epsilon(t) = \mathbb{E}\,\langle Q\rangle_{n,t,\epsilon}$, which is approximately equal to Q because it concentrates by Proposition 4. However, $\mathbb{E}\,\langle Q\rangle_{n,t,\epsilon}$ depends on $\int_0^t q_\epsilon(v)dv$ (and on t, $\int_0^t r_\epsilon(v)dv$ and ϵ too). The equation $q_\epsilon(t) = \mathbb{E}\,\langle Q\rangle_{n,t,\epsilon}$ is therefore a first order differential equation over $t \mapsto \int_0^t q_\epsilon(v)dv$. We will see in details in Sec. 4.4 that it possesses a solution, but for the moment we just assume it exists in order to derive the following *fundamental sum rule*, which is a core identity in the proof scheme:

Proposition 5 (Fundamental sum rule). *Assume that the interpolation functions (q_ϵ) and (r_ϵ) are regular (see Definition 1). Assume that for all $t \in [0,1]$ and $\epsilon \in \mathcal{B}_n$ we have $q_\epsilon(t) = \mathbb{E}\langle Q\rangle_{n,t,\epsilon}$. Then*

$$f_n = \frac{1}{s_n^2}\int_{\mathcal{B}_n}\Big\{\psi_{P_0}\big(\textstyle\int_0^1 r_\epsilon(t)dt\big) + \alpha\Psi_{P_{\rm out}}\big(\int_0^1 q_\epsilon(t)dt;\rho\big) - \frac{1}{2}\int_0^1 q_\epsilon(t)r_\epsilon(t)dt\Big\}d\epsilon + \mathcal{O}_n(1)\,, \tag{88}$$

where $o_n(1)$ denotes a quantity that goes to 0 as $n \to \infty$ uniformly w.r.t. the choice of the interpolation functions.

Proof. By the Cauchy-Schwarz inequality

$$\Big(\frac{1}{s_n^2}\int_{\mathcal{B}_n} d\epsilon \int_0^1 dt\, \mathbb{E}\Big\langle \Big(\frac{1}{n}\sum_{\mu=1}^m u'_{Y_{t,\mu}}(S_{t,\mu})u'_{Y_{t,\mu}}(s_{t,\mu}) - r_\epsilon(t)\Big)\big(Q - q_\epsilon(t)\big)\Big\rangle_{n,t,\epsilon}\Big)^2$$

$$\leq \frac{1}{s_n^2}\int_{\mathcal{B}_n} d\epsilon \int_0^1 dt\, \mathbb{E}\Big\langle \Big(\frac{1}{n}\sum_{\mu=1}^m u'_{Y_{t,\mu}}(S_{t,\mu})u'_{Y_{t,\mu}}(s_{t,\mu}) - r_\epsilon(t)\Big)^2\Big\rangle_{n,t,\epsilon} \times \frac{1}{s_n^2}\int_{\mathcal{B}_n} d\epsilon \int_0^1 dt\, \mathbb{E}\big\langle \big(Q - q_\epsilon(t)\big)^2\big\rangle_{n,t,\epsilon}.$$

The first term of this product is bounded by some constant $C(\varphi,\alpha)$ that only depend on φ and α, see Appendix A.6. The second term is bounded by $C(\varphi,S,\alpha)n^{-1/8}$ by Proposition 4, since we assumed that for all $\epsilon \in \mathcal{B}_n$ and all $t \in [0,1]$ we have $q_\epsilon(t) = \mathbb{E}\langle Q\rangle_{n,t,\epsilon}$. We have therefore

$$\Big|\frac{1}{s_n^2}\int_{\mathcal{B}_n} d\epsilon \int_0^1 dt\, \mathbb{E}\Big\langle \Big(\frac{1}{n}\sum_{\mu=1}^m u'_{Y_{t,\mu}}(S_{t,\mu})u'_{Y_{t,\mu}}(s_{t,\mu}) - r_\epsilon(t)\Big)\big(Q - q_\epsilon(t)\big)\Big\rangle_{n,t,\epsilon}\Big| \leq \frac{C(\varphi,S,\alpha)}{n^{1/16}}.$$

Therefore from (85)

$$\frac{1}{s_n^2}\int_{\mathcal{B}_n} d\epsilon \int_0^1 dt \frac{df_{n,\epsilon}(t)}{dt} = \frac{1}{2s_n^2}\int_{\mathcal{B}_n} d\epsilon \int_0^1 dt\big\{q_\epsilon(t)r_\epsilon(t) - r_\epsilon(t)\rho\big\} + o_n(1) + \mathcal{O}(n^{-1/16}). \tag{89}$$

Here the small terms are going to 0 both uniformly w.r.t. to the choice of q_ϵ and r_ϵ. When replacing (89) in (84) and combining it with (83) we reach the claimed identity (88), but up to the fact that $\Psi_{P_{\rm out}}(\int_0^1 q_\epsilon(t)dt;\rho)$ is multiplied by m/n instead of α. Recalling that $m/n \to \alpha$ as $m,n \to \infty$ allows to finish the argument (notice that $\Psi_{P_{\rm out}}$ is continuous and hence bounded on $[0,\rho]$, see Proposition 18). □

We are now ready to prove matching bounds.

4.4 Lower and upper matching bounds

We now possess all the necessary tools to prove Theorem 1 in three steps.

(i) We prove that, under assumptions (H1), (H2) and (H3), $\lim_{n\to\infty} f_n = \sup_{r\geq 0}\inf_{q\in[0,\rho]} f_{\rm RS}(q,r)$.

(ii) Under hypothesis (H2), the function $\Psi_{P_{\rm out}}$ is convex, Lipschitz and non-decreasing (Proposition 19). We thus apply Corollary 7 of Appendix D to get $\sup_{r\geq 0}\inf_{q\in[0,\rho]} f_{\rm RS}(q,r) = \sup_{q\in[0,\rho]}\inf_{r\geq 0} f_{\rm RS}(q,r)$. We then deduce from (i) that $\lim_{n\to\infty} f_n = \sup_{q\in[0,\rho]}\inf_{r\geq 0} f_{\rm RS}(q,r)$ under (H1)-(H2)-(H3).

(iii) Finally, the approximation arguments given in Appendix C permit to relax (H1)-(H2) to the weaker hypotheses (h1)-(h2) and allow to replace the Gaussian assumption (H3) on $\mathbf{\Phi}$ by (h1)-(h3)-(h4). The fact that for discrete channels the Gaussian noise can then be removed, allowing to replace (h5.a) (i.e. $\Delta > 0$ treated until here) to (h5.b) (i.e. $\Delta = 0$ and φ takes values in $\mathbb{N}$), is proven in Sec. C.3. This proves the first equality of Theorem 1. The last equality in (24) and the remaining part of Theorem 1 follow then from Lemma 23.

It thus remains to tackle (i), but before that we need a definition. For $t \in [0,1]$ and $\epsilon \in \mathcal{B}_n$, we write $R^t(\epsilon) = (R_1(t,\epsilon), R_2(t,\epsilon))$. The quantity $\mathbb{E}\langle Q\rangle_{n,t,\epsilon}$ is a function of $n, t, R^t(\epsilon)$ that we write $\mathbb{E}\langle Q\rangle_{n,t,\epsilon} = F_n\big(t, R^t(\epsilon)\big)$, where F_n is a function defined on

$$D_n := \Big\{(t,r_1,r_2) \in [0,1]\times\mathbb{R}_+\times\mathbb{R}_+ \,\Big|\, r_2 \leq \rho t + 2s_n\Big\}. \tag{90}$$

The following proposition, proven in Appendix A.7, will be useful.

Proposition 6. *F_n is a continuous function from D_n to $[0,\rho]$. Let D_n° denotes the interior of D_n. F_n admits partial derivatives with respect to its second and third argument on D_n°. These partial derivatives are both continuous and non-negative on D_n°.*

Let us now start with the lower bound.

4.4.1 Lower bound

Proposition 7 (Lower bound)**.** *The free entropy* (10) *verifies*

$$\liminf_{n\to\infty} f_n \geq \sup_{r\geq 0} \inf_{q\in[0,\rho]} f_{\rm RS}(q,r)\,. \tag{91}$$

Proof. We consider, for $(\epsilon_1,\epsilon_2) \in \mathcal{B}_n$ and a fixed value $r \in [0, r_{\max}]$, the following 1st order differential equation:

$$y(0) = (\epsilon_1,\epsilon_2) \qquad \text{and} \qquad \forall\, t\in[0,1], \quad y'(t) = \big(r, F_n(t,y(t))\big)\,. \tag{92}$$

By the Cauchy-Lipschitz Theorem (see for instance Theorem 3.1 in Chapter V from [51]) this equation admits a (unique) solution that we write $y(\cdot,\epsilon) = \big(y_1(\cdot,\epsilon), y_2(\cdot,\epsilon)\big)$. The hypotheses of the Cauchy-Lipschitz Theorem are verified, because of Proposition 6. We define then, for all $t\in[0,1]$,

$$r_\epsilon(t) = y_1'(t,\epsilon) = r \qquad \text{and} \qquad q_\epsilon(t) = y_2'(t,\epsilon) = F_n(t,y(t,\epsilon)) \in [0,\rho]\,.$$

We have therefore $R_1(t,\epsilon) = \epsilon_1 + \int_0^t y_1'(s,\epsilon)ds = y_1(t,\epsilon)$ and similarly $R_2(t,\epsilon) = y_2(t,\epsilon)$. We obtain that for all $t\in[0,1]$,

$$q_\epsilon(t) = F_n(t,y(t,\epsilon)) = F_n\big(t,(R_1(t,\epsilon),R_2(t,\epsilon))\big) = \mathbb{E}\langle Q\rangle_{n,t,\epsilon}\,.$$

Let us show now that the functions (q_ϵ) and (r_ϵ) are regular (see Definition 1). Let $t\in[0,1]$. The function $R^t : \epsilon \mapsto (R_1(t,\epsilon), R_2(t,\epsilon)) = y(t,\epsilon)$ is the flow of (92) and is thus injective (by unicity of the solution) and $\mathcal{C}^1$ because of the regularity properties (see Proposition 6) of F_n. The Jacobian of the flow is given by the Liouville formula (see Corollary 3.1 in Chapter V from [51]):

$$\det\Big(\frac{\partial R^t}{\partial \epsilon}(\epsilon)\Big) = \exp\Big(\int_0^t dv \frac{\partial F_n}{\partial y_2}(v,y(v,\epsilon))\Big) \geq 1,$$

because by Proposition 6 we have $\partial_{y_2} F_n \geq 0$. We obtain (by the local inversion Theorem) that R^t is a $\mathcal{C}^1$ diffeomorphism, and since its Jacobian is greater or equal to 1 the functions (q_ϵ) and (r_ϵ) are regular.

We have seen that for all $\epsilon\in\mathcal{B}_n$ and all $t\in[0,1]$, $q_\epsilon(t) = \mathbb{E}\langle Q\rangle_{t,n,\epsilon}$, so we can apply Proposition 5 to get

$$\begin{aligned} f_n &= \frac{1}{s_n^2}\int_{\mathcal{B}_n} \Big\{\psi_{P_0}(r) + \alpha\Psi_{P_{\rm out}}\big(\textstyle\int_0^1 q_\epsilon(t)dt;\rho\big) - \frac{r}{2}\int_0^1 q_\epsilon(t)dt\Big\} d\epsilon + \mathcal{O}_n(1) \\ &= \frac{1}{s_n^2}\int_{\mathcal{B}_n} f_{\rm RS}\big(\textstyle\int_0^1 q_\epsilon(t)dt, r\big)d\epsilon + \mathcal{O}_n(1) \\ &\geq \inf_{q\in[0,\rho]} f_{\rm RS}(q,r) + \mathcal{O}_n(1) \end{aligned}$$

and thus $\liminf_{n\to\infty} f_n \geq \inf_{q\in[0,\rho]} f_{\rm RS}(q,r)$. This is true for all $r\in[0,r_{\max}]$ so we get

$$\liminf_{n\to\infty} f_n \geq \sup_{r\in[0,r_{\max}]} \inf_{q\in[0,\rho]} f_{\rm RS}(q,r)\,. \tag{93}$$

Let $r \geq r_{\max}$. We have for all $q\in[0,\rho]$, $\partial_q f_{\rm RS}(q,r) = \alpha\Psi'_{P_{\rm out}}(q) - \frac{r}{2} \leq 0$, because $r\geq r_{\max} \geq 2\alpha\Psi'_{P_{\rm out}}(q)$.

Therefore for all $r \geq r_{\max}$, $\inf_{q\in[0,\rho]} f_{\rm RS}(q,r) = f_{\rm RS}(\rho,r)$ and

$$\frac{\partial}{\partial r}\inf_{q\in[0,\rho]} f_{\rm RS}(q,r) = \frac{\partial}{\partial r} f_{\rm RS}(\rho,r) = \psi_{P_0}'(r) - \frac{\rho}{2} \leq 0,$$

because by Proposition 17, ψ_{P_0} is $\frac{\rho}{2}$-Lipschitz. The function $r \mapsto \inf_{q\in[0,\rho]} f_{\rm RS}(q,r)$ is therefore non-increasing on $[r_{\max},+\infty)$. Going back to (93), we conclude

$$\liminf_{n\to\infty} f_n \geq \sup_{r\in[0,r_{\max}]}\inf_{q\in[0,\rho]} f_{\rm RS}(q,r) = \sup_{r\geq 0}\inf_{q\in[0,\rho]} f_{\rm RS}(q,r)\,. \tag{94}$$

□

4.4.2 Upper bound

Proposition 8 (Upper bound)**.** *The free entropy* (10) *verifies*

$$\limsup_{n\to\infty} f_n \leq \sup_{r\geq 0}\inf_{q\in[0,\rho]} f_{\rm RS}(q,r)\,. \tag{95}$$

Proof. We consider, for $(\epsilon_1,\epsilon_2)\in\mathcal{B}_n$, the following order-1 system of differential equations:

$$y(0) = (\epsilon_1,\epsilon_2) \qquad \text{and} \qquad \forall\, t\in[0,1], \quad y'(t) = \begin{pmatrix} 2\alpha\Psi_{P_{\rm out}}'\big(F_n(t,y(t))\big) \\ F_n(t,y(t)) \end{pmatrix}. \tag{96}$$

By Proposition 18 the function $\Psi_{P_{\rm out}}'$ is $\mathcal{C}^1$ and takes values in $[0,r_{\max}]$. By Proposition 6, the function F_n is continuous, bounded and admits partial derivatives w.r.t. its second and third arguments, that are continuous. We can therefore apply the Cauchy-Lipschitz Theorem as in the proof of Proposition 7: The equation (96) admits a (unique) solution that we write $y(\cdot,\epsilon) = \big(y_1(\cdot,\epsilon),y_2(\cdot,\epsilon)\big)$. We define then, for all $t\in[0,1]$,

$$r_\epsilon(t) = y_1'(t,\epsilon) = 2\alpha\Psi_{P_{\rm out}}'\big(F_n(t,y(t,\epsilon))\big) \in [0,r_{\max}] \qquad \text{and} \qquad q_\epsilon(t) = y_2'(t,\epsilon) = F_n(t,y(t,\epsilon)) \in [0,\rho]\,.$$

We have therefore $R_1(t,\epsilon) = \epsilon_1 + \int_0^t y_1'(s,\epsilon)ds = y_1(t,\epsilon)$ and similarly $R_2(t,\epsilon) = y_2(t,\epsilon)$. We obtain that for all $t\in[0,1]$,

$$q_\epsilon(t) = F_n(t,y(t,\epsilon)) = F_n\big(t,(R_1(t,\epsilon),R_2(t,\epsilon))\big) = \mathbb{E}\langle Q\rangle_{n,t,\epsilon}\,.$$

Let us show now that the functions (q_ϵ) and (r_ϵ) are regular (see Definition 1). Let $t\in[0,1]$. The function $R^t : \epsilon \mapsto (R_1(t,\epsilon),R_2(t,\epsilon)) = y(t,\epsilon)$ is the flow of (96) and is thus injective and $\mathcal{C}^1$ because of the regularity properties (see Proposition 6) of F_n. The Jacobian of the flow is again given by the Liouville formula:

$$\det\Big(\frac{\partial R^t}{\partial\epsilon}(\epsilon)\Big) = \exp\Big(\int_0^t dv 2\alpha\frac{\partial F_n}{\partial y_1}(v,y(v,\epsilon))\Psi_{P_{\rm out}}''\big(F_n(v,y(v,\epsilon))\big) + \int_0^t dv\frac{\partial F_n}{\partial y_2}(v,y(v,\epsilon))\Big) \geq 1,$$

because by Proposition 6, $\frac{\partial F_n}{\partial y_1}$ and $\frac{\partial F_n}{\partial y_2}$ are both non negative and since $\Psi_{P_{\rm out}}$ is convex (see Proposition 18), we have also $\Psi_{P_{\rm out}}'' \geq 0$. We obtain (by the local inversion Theorem) that R^t is a $\mathcal{C}^1$ diffeomorphism. Its Jacobian is greater or equal to 1, and the functions (q_ϵ) and (r_ϵ) are therefore regular.

We have seen that for all $\epsilon\in\mathcal{B}_n$ and all $t\in[0,1]$, $q_\epsilon(t) = \mathbb{E}\langle Q\rangle_{t,n,\epsilon}$, so we can apply Proposition 5 to get

$$\begin{aligned} f_n &= \frac{1}{s_n^2}\int_{\mathcal{B}_n}\Big\{\psi_{P_0}\big(\textstyle\int_0^1 r_\epsilon(t)dt\big) + \alpha\Psi_{P_{\rm out}}\big(\int_0^1 q_\epsilon(t)dt;\rho\big) - \frac{1}{2}\int_0^1 q_\epsilon(t)r_\epsilon(t)dt\Big\}d\epsilon + \mathcal{O}_n(1) \\ &\leq \frac{1}{s_n^2}\int_{\mathcal{B}_n}\int_0^1\Big\{\psi_{P_0}(r_\epsilon(t)) + \alpha\Psi_{P_{\rm out}}(q_\epsilon(t);\rho) - \frac{1}{2}q_\epsilon(t)r_\epsilon(t)\Big\}dtd\epsilon + \mathcal{O}_n(1) \end{aligned} \tag{97}$$

by Jensen's inequality, because by Propositions 17 and 18 the functions ψ_{P_0} and $\Psi_{P_{\rm out}}$ are convex.

Let us fix $\epsilon \in \mathcal{B}_n$ and $t \in [0,1]$. By definition of r_ϵ and q_ϵ, we have

$$r_\epsilon(t) = 2\alpha\Psi'_{P_{\rm out}}\big(F_n(t, y(t,\epsilon))\big) = 2\alpha\Psi'_{P_{\rm out}}\big(q_\epsilon(t)\big). \tag{98}$$

The function $g : q \in [0,\rho] \mapsto 2\alpha\Psi_{P_{\rm out}}(q;\rho) - r_\epsilon(t)q$ is convex by Proposition 18. By equation (98) above, we see that $g'(q_\epsilon(t)) = 0$ and therefore:

$$\alpha\Psi_{P_{\rm out}}(q_\epsilon(t);\rho) - \frac{1}{2}q_\epsilon(t)r_\epsilon(t) = \inf_{q\in[0,\rho]}\big\{\alpha\Psi_{P_{\rm out}}(q;\rho) - \frac{1}{2}q\,r_\epsilon(t)\big\}.$$

This holds for all $\epsilon \in \mathcal{B}_n$ and all $t \in [0,1]$. Plugging this back in (97), we get:

$$\begin{aligned} f_n &\le \frac{1}{s_n^2}\int_{\mathcal{B}_n}\int_0^1 \inf_{q\in[0,\rho]}\Big\{\psi_{P_0}(r_\epsilon(t)) + \alpha\Psi_{P_{\rm out}}(q;\rho) - \frac{1}{2}q\,r_\epsilon(t)\Big\}dtd\epsilon + \mathcal{O}_n(1) \\ &\le \sup_{r\ge 0}\inf_{q\in[0,\rho]}\Big\{\psi_{P_0}(r) + \alpha\Psi_{P_{\rm out}}(q;\rho) - \frac{1}{2}q\,r\Big\} + \mathcal{O}_n(1)\,. \end{aligned}$$

This proves Proposition 8. □

From the arguments given at the beginning of the section, this ends the proof of Theorem 1.

5 Proofs of the limits of optimal errors

5.1 Optimal generalization error: Proof of Theorem 4

5.1.1 Formal derivation and proof idea: A teacher-student scenario with side information

Before proving Theorem 4 rigorously, we find useful to provide a conceptual framework allowing to formally derive the generalization error (a framework that will actually serve as a basis for the rigorous derivation presented in the next section). In order to obtain the (generalized) optimal generalization error, we need first to assume that the new "test labels" are also observed by the student in the teacher-student scenario of Sec. 1.2 but with a very low signal-to-noise ratio. The presence of this side information will allow us to use the I-MMSE relation (Proposition 13) to obtain the generalization error when small, but non-zero, information about the test labels is known by the student. Then, by formally taking the limit of vanishing side information on the resulting expression (and *assuming* that the large n and vanishing side information limits commute), we will recover the generalization error. We thus now introduce the following "train-test" observation model.

The set of patterns and labels are divided into two sets by the teacher: The *training set* $\mathcal{S}^{\rm tr}$ of size m that will be used as the main source of information by the student in order to then generalize, and the *test set* $\mathcal{S}^{\rm te}$ of size $m' = \epsilon n$ that will be used by the teacher in order to evaluate the performance of the student, but also by the student as small additional side information. Let us be more precise: The teacher gives to the student both the patterns and associated labels of the training set, namely $\mathcal{S}^{\rm tr} := \{(Y_\mu;\mathbf{\Phi}_\mu)\}_{\mu=1}^m$ (recall the labels are given by (1), (2)). For the test set, the test patterns to classify are given to the student but the associated labels are (almost) not: Let $\epsilon,\lambda \ge 0$. Instead of the test labels $\{\widetilde{Y}_\mu\}_{\mu=1}^{m'}$ (that should be totally unknown to the student in the ideal setting), what is given to the student is

$$U_\mu = \sqrt{\lambda}\,Y'_\mu + Z'_\mu\,, \qquad \text{for} \qquad 1 \le \mu \le m' = \epsilon n\,, \tag{99}$$

where $Z'_\mu \overset{\text{iid}}{\sim} \mathcal{N}(0,1)$, and Y'_μ is given by

$$Y'_\mu = f(\widetilde{Y}_\mu)\,, \qquad \widetilde{Y}_\mu \sim P_{\rm out}\Big(\cdot \,\Big|\, \frac{\mathbf{\Phi}'_\mu \cdot \mathbf{X}^*}{\sqrt{n}}\Big)\,, \tag{100}$$

where $f : \mathbb{R} \to \mathbb{R}$ is a continuous bounded function and $\mathbf{\Phi}'_\mu \overset{\text{iid}}{\sim} \mathcal{N}(0, \mathbf{I}_n)$ independently of everything else. We will first prove Theorem 4 for continuous bounded functions f, and then relax this at the end of the proof. The test set given to the student, in addition of the training set, is $\mathcal{S}^{\rm te} = \mathcal{S}^{\rm te}(\lambda,\epsilon) := \{(U_\mu = \sqrt{\lambda}\, Y'_\mu + Z'_\mu; \mathbf{\Phi}'_\mu)\}_{\mu=1}^{\epsilon n}$ where λ *is typically very small.* Indeed, we are particularly interested in the case $\lambda, \epsilon \to 0$ when the student has *no* information about the test labels, which is the ideal setting we want to study. But in order to employ the I-MMSE relation we consider instead very small $\lambda > 0$.

The learning of the classifier $\mathbf{X}^*$ given $\mathcal{S}^{\rm tr}$ *and* $\mathcal{S}^{\rm te}$ is a slight extension of model (1). Define $\mathbf{Y}' = (Y'_\mu)_{\mu=1}^{m'}$ as the vector of test labels (before they are corrupted by additional noise through (99)). Then the (generalized) optimal generalization error *with side information* (i.e. at $\lambda, \epsilon > 0$) in this "train-test" observation model is

$$\mathcal{E}^{\rm side}_{f,n}(\lambda,\epsilon) := \min_{\widehat{\mathbf{Y}}'} \frac{1}{\epsilon n}\mathbb{E}\big[\big\|\mathbf{Y}' - \widehat{\mathbf{Y}}'(\mathcal{S}^{\rm te},\mathcal{S}^{\rm tr})\big\|^2\big] = \frac{1}{\epsilon n}\mathbb{E}\big[\big\|\mathbf{Y}' - \mathbb{E}\big[\mathbf{Y}'\big|\mathcal{S}^{\rm te},\mathcal{S}^{\rm tr}\big]\big\|^2\big]\,. \tag{101}$$

The "true" generalization error (41) is recovered by defining instead $\mathcal{S}^{\rm te} = \mathbf{\Phi}'$ or equivalently letting $\lambda, \epsilon \to 0$, i.e. when only $\mathcal{S}^{\rm tr}$ and the test patterns are given to the student: $\lim_{\lambda,\epsilon\to 0} \mathcal{E}^{\rm side}_{f,n}(\lambda,\epsilon) = \mathcal{E}_{f,n}$. Note that the f function only plays a role in the test set, while the labels of the training data are generated through the "pure model" (1), (2).

From there one can use the I-MMSE relation of Proposition 13 in order to formally compute the limiting $n \to \infty$ expression of (101). Indeed,

$$\frac{\partial}{\partial\lambda}\frac{1}{n} I(\mathbf{Y}'; \sqrt{\lambda}\mathbf{Y}' + \mathbf{Z}'|\mathbf{Y}, \mathbf{\Phi}, \mathbf{\Phi}') = \frac{\epsilon}{2}\mathcal{E}^{\rm side}_{f,n}(\lambda,\epsilon)\,. \tag{102}$$

Fortunately, by a straightforward extension of the interpolation method presented in Sec. 4 one can generalize Theorem 1 to take into account this additional side information and access this mutual information (see the end of the section for the proof):

Lemma 2. *For all $\epsilon, \lambda \geq 0$ we have*

$$\frac{1}{n} I(\mathbf{Y}'; \sqrt{\lambda}\mathbf{Y}' + \mathbf{Z}'|\mathbf{Y}, \mathbf{\Phi}, \mathbf{\Phi}') \xrightarrow[n\to\infty]{} \inf_{q\in[0,\rho]} \sup_{r\geq 0} \tilde{i}_{\rm RS}(q,r,\lambda) - i_\infty\,, \tag{103}$$

where i_∞ is given by Corollary 1 and

$$\tilde{i}_{\rm RS}(q,r,\lambda) := i_{\rm RS}(q,r) + \epsilon I(f(Y^{(q)}); \sqrt{\lambda}\, f(Y^{(q)}) + Z'|V) \tag{104}$$

$$= I_{P_0}(r) + \alpha \mathcal{I}_{P_{\rm out}}(q;\rho) + \epsilon I(f(Y^{(q)}); \sqrt{\lambda}\, f(Y^{(q)}) + Z'|V) - \frac{r}{2}(\rho - q)\,. \tag{105}$$

Recall that $Y^{(q)}$ is sampled from the "second scalar channel" (20): $Y^{(q)} \sim P_{\rm out}(\cdot\,|\,\sqrt{q}\,V + \sqrt{\rho-q}\,W^*)$, where $V, W^* \overset{\text{iid}}{\sim} \mathcal{N}(0,1)$.

Define the following MMSE function:

$$M_f : (\lambda, q) \mapsto \mathrm{MMSE}\big(f(Y^{(q)})\big|\sqrt{\lambda}\, f(Y^{(q)}) + Z', V\big)\,. \tag{106}$$

By concavity arguments detailed in the next section, we have almost everywhere

$$\lim_{n\to\infty}\frac{\partial}{\partial\lambda}\frac{1}{n}I(\mathbf{Y}';\sqrt{\lambda}\mathbf{Y}'+\mathbf{Z}'|\mathbf{Y},\mathbf{\Phi},\mathbf{\Phi}')=\frac{\partial}{\partial\lambda}\lim_{n\to\infty}\frac{1}{n}I(\mathbf{Y}';\sqrt{\lambda}\mathbf{Y}'+\mathbf{Z}'|\mathbf{Y},\mathbf{\Phi},\mathbf{\Phi}')=\frac{\partial}{\partial\lambda}\inf_{q\in[0,\rho]}\sup_{r\geq 0}\tilde{i}_{\rm RS}(q,r,\lambda)$$

using Lemma 2 for the last equality. Assuming $\partial_\lambda \inf_{q\in[0,\rho]}\sup_{r\geq0}\tilde{i}_{\rm RS}(q,r,\lambda)=\partial_\lambda\tilde{i}_{\rm RS}(q,r,\lambda)|_{(q_\lambda^*,r_\lambda^*)}$, where $(q_\lambda^*,r_\lambda^*)$ is an optimal couple, (102) and the last identity combined lead to

$$\frac{\epsilon}{2}\lim_{n\to\infty}\mathcal{E}_{f,n}^{\rm side}(\lambda,\epsilon)=\epsilon\frac{\partial}{\partial\lambda}I\big(f(Y^{(q)});\sqrt{\lambda}f(Y^{(q)})+Z'\big|V\big)\Big|_{q_\lambda^*}=\frac{\epsilon}{2}M_f(\lambda,q_\lambda^*)\,, \tag{107}$$

using again the I-MMSE relation for the last equality. Thus $\lim_{n\to\infty}\mathcal{E}_{f,n}^{\rm side}(\lambda,\epsilon)=M_f(\lambda,q_\lambda^*)$.

A formal calculation of the vanishing side information limit of $M_f(\lambda,q_\lambda^*)$ gives back $\mathcal{E}_f(q^*(\alpha))$ (recall (42) and $q^*(\alpha)$ is the optimizer of the replica-symmetric formula (24)), so that $\lim_{\lambda,\epsilon\to0}\lim_{n\to\infty}\mathcal{E}_{f,n}^{\rm side}(\lambda,\epsilon)=\mathcal{E}_f(q^*(\alpha))$. It is very natural to believe that the vanishing side information limit of $\lim_{n\to\infty}\mathcal{E}_{f,n}^{\rm side}(\lambda,\epsilon)$ should give back the true asymptotic generalization error. So if one could justify the commutation of limits

$$\lim_{\lambda,\epsilon\to0}\lim_{n\to\infty}\mathcal{E}_{f,n}^{\rm side}(\lambda,\epsilon)=\lim_{n\to\infty}\lim_{\lambda,\epsilon\to0}\mathcal{E}_{f,n}^{\rm side}(\lambda,\epsilon)=\lim_{n\to\infty}\mathcal{E}_{f,n}$$

this would end the proof. We prove this point in the next section.

Proof of Lemma 2: Extending the interpolation method presented in Sec. 4, one can generalize Theorem 1 to take into account this additional side information. This gives directly

$$\frac{1}{n}I(\mathbf{X}^*;\mathbf{Y},\sqrt{\lambda}\mathbf{Y}'+\mathbf{Z}'|\mathbf{\Phi},\mathbf{\Phi}')\xrightarrow[n\to\infty]{}\tilde{I}_\infty(\alpha,\epsilon,\lambda):=\inf_{q\in[0,\rho]}\sup_{r\geq0}\tilde{I}_{\rm RS}(q,r,\lambda) \tag{108}$$

where $\tilde{I}_{\rm RS}(q,r,\lambda)$ is given by

$$\tilde{I}_{\rm RS}(q,r,\lambda):=I_{P_0}(r)+\alpha\mathcal{I}_{P_{\rm out}}(q;\rho)+\epsilon I(W^*;\sqrt{\lambda}f(Y^{(q)})+Z'|V)-\frac{r}{2}(\rho-q)\,. \tag{109}$$

Conditionally on $(V,f(Y^{(q)}))$, the random variables W^* and $\sqrt{\lambda}f(Y^{(q)})+Z'$ are independent, therefore

$$I\big(f(Y^{(q)});\sqrt{\lambda}f(Y^{(q)})+Z'\big|V\big)=I\big(W^*,f(Y^{(q)});\sqrt{\lambda}f(Y^{(q)})+Z'\big|V\big)\,.$$

Now, by the chain rule of the mutual information we have

$$I\big(W^*,f(Y^{(q)});\sqrt{\lambda}f(Y^{(q)})+Z'\big|V\big)=I\big(W^*;\sqrt{\lambda}f(Y^{(q)})+Z'\big|V\big)+I\big(f(Y^{(q)});\sqrt{\lambda}f(Y^{(q)})+Z'\big|V,W^*\big)\,.$$

We obtain that

$$I\big(W^*;\sqrt{\lambda}f(Y^{(q)})+Z'\big|V\big)=I\big(f(Y^{(q)});\sqrt{\lambda}f(Y^{(q)})+Z'\big|V\big)-I\big(f(Y^{(q)});\sqrt{\lambda}f(Y^{(q)})+Z'\big|V,W^*\big)\,. \tag{110}$$

Notice that the last mutual information in the above equation does not depend on q nor r. Therefore we have:

$$\inf_{q\in[0,\rho]}\sup_{r\geq0}\tilde{I}_{\rm RS}(q,r,\lambda)=-\epsilon I\big(f(Y^{(q)});\sqrt{\lambda}f(Y^{(q)})+Z'\big|V,W^*\big)+\inf_{q\in[0,\rho]}\sup_{r\geq0}\tilde{i}_{\rm RS}(q,r,\lambda)\,. \tag{111}$$

Now, by the chain rule, we have

$$\frac{1}{n}I(\mathbf{X}^*;\mathbf{Y},\sqrt{\lambda}\mathbf{Y}'+\mathbf{Z}'|\mathbf{\Phi},\mathbf{\Phi}') = \frac{1}{n}I(\mathbf{X}^*;\mathbf{Y}|\mathbf{\Phi}) + \frac{1}{n}I(\mathbf{X}^*;\sqrt{\lambda}\mathbf{Y}'+\mathbf{Z}'|\mathbf{Y},\mathbf{\Phi},\mathbf{\Phi}')\,. \tag{112}$$

The limit of the left-hand side is given by (108). By Corollary 1, we have $\lim_{n\to\infty} I(\mathbf{X}^*;\mathbf{Y}|\mathbf{\Phi})/n = i_\infty$. It remains to investigate the last term of the equation above. By the arguments used to prove (110), we have

$$\begin{aligned} I(\mathbf{X}^*;\sqrt{\lambda}\mathbf{Y}'+\mathbf{Z}'|\mathbf{Y},\mathbf{\Phi},\mathbf{\Phi}') &= I(\mathbf{Y}';\sqrt{\lambda}\mathbf{Y}'+\mathbf{Z}'|\mathbf{Y},\mathbf{\Phi},\mathbf{\Phi}') - I(\mathbf{Y}';\sqrt{\lambda}\mathbf{Y}'+\mathbf{Z}'|\mathbf{Y},\mathbf{\Phi},\mathbf{X}^*,\mathbf{\Phi}') \\ &= I(\mathbf{Y}';\sqrt{\lambda}\mathbf{Y}'+\mathbf{Z}'|\mathbf{Y},\mathbf{\Phi},\mathbf{\Phi}') - I(\mathbf{Y}';\sqrt{\lambda}\mathbf{Y}'+\mathbf{Z}'|\mathbf{X}^*,\mathbf{\Phi}')\,. \end{aligned} \tag{113}$$

We have $I(\mathbf{Y}';\sqrt{\lambda}\mathbf{Y}'+\mathbf{Z}'|\mathbf{X}^*,\mathbf{\Phi}')/n = \epsilon I(Y_1';\sqrt{\lambda}Y_1'+Z_1'|\mathbf{X}^*,\mathbf{\Phi}_1')$ and it is not difficult to show, using similar computations as in the proof of Corollary 1, that

$$I(Y_1';\sqrt{\lambda}Y_1'+Z_1'|\mathbf{X}^*,\mathbf{\Phi}_1') \xrightarrow[n\to\infty]{} I\big(f(Y^{(q)});\sqrt{\lambda}f(Y^{(q)})+Z'\big|V,W^*\big)\,,$$

(recall that the right-hand side does not depend on q). Combining this with (113), (112), (108), Corollary 1 and (111), we obtain the desired result. □

5.1.2 Proof of Theorem 4

In order to compute the limit of the (generalized) generalization error, we work in the teacher-student scenario with side-information discussed in the previous section.

Lemma 3. *For all $\alpha,\lambda>0$ the set*

$$D_{\alpha,\lambda} := \big\{\epsilon\geq 0\,\big|\,\text{the infimum in (103) is achieved at a unique } q^*_{\alpha,\epsilon,\lambda}\big\} \tag{114}$$

is equal to $[0,+\infty)$ minus some countable set. Moreover, $\epsilon\mapsto q^*_{\alpha,\lambda,\epsilon}$ is continuous on $D_{\alpha,\epsilon}$.

Proof. This follows from the same arguments than the proof of Proposition 1. □

Lemma 4. *For all $\alpha,\lambda>0$, we have for all $\epsilon\in D_{\alpha,\lambda}\setminus\{0\}$*

$$\lim_{n\to\infty}\mathrm{MMSE}(Y_1'|\mathbf{Y},\boldsymbol{U},\mathbf{\Phi},\mathbf{\Phi}') = M_f(\lambda,q^*_{\alpha,\epsilon,\lambda})\,,$$

where $q^*_{\alpha,\epsilon,\lambda}$ is the unique minimizer of (103).

Proof. Let us fix $\alpha,\epsilon>0$. Consider the function

$$h_{\alpha,\epsilon}:\lambda\mapsto \inf_{q\in[0,\rho]}\sup_{r\geq 0}\tilde{i}_{\mathrm{RS}}(q,r,\lambda)\,. \tag{115}$$

Corollary 4 from [52] gives that $h_{\alpha,\epsilon}$ is differentiable at λ if and only if

$$\Big\{\epsilon\frac{\partial}{\partial\lambda}I\big(f(Y^{(q)});\sqrt{\lambda}f(Y^{(q)})+Z'\big|V\big) = \frac{\epsilon}{2}M_f(\lambda,q)\,\Big|\,q \text{ minimizer of (103) (or equivalently of (115))}\Big\}$$

is a singleton (the equality comes from the I-MMSE relation from Proposition 13). In such case, Corollary 4 from [52] also gives that

$$h'_{\alpha,\epsilon}(\lambda) = \frac{\epsilon}{2}M_f(\lambda,q)\,, \tag{116}$$

for all q minimizer of (115). So if now $\epsilon\in D_{\alpha,\lambda}\setminus\{0\}$, then the minimizer is unique and thus $h_{\alpha,\epsilon}$ is differentiable at λ, with derivative $h'_{\alpha,\lambda}(\lambda)=\epsilon M_f(\lambda,q^*_{\alpha,\epsilon,\lambda})/2$. However, by (103) in Lemma 2, $h_{\alpha,\epsilon}$ is the pointwise

limit on $\mathbb{R}_+$ of the sequence of concave functions

$$(h_n)_{n\geq 1} = \left(\lambda \mapsto \frac{1}{n} I\big(\mathbf{Y}'; \sqrt{\lambda}\mathbf{Y}' + \mathbf{Z}' \big| \mathbf{Y}, \mathbf{\Phi}, \mathbf{\Phi}'\big) + i_\infty\right)_{n\geq 1}.$$

Consequently, a standard convex analysis result gives that $h_n'(\lambda) \xrightarrow[n\to\infty]{} h'_{\alpha,\epsilon}(\lambda)$. By the I-MMSE relation (Proposition 13) we have $h_n'(\lambda) = \epsilon \mathrm{MMSE}(Y_1'|\mathbf{Y}, \mathbf{U}, \mathbf{\Phi}, \mathbf{\Phi}')/2$ and we conclude using the fact that $\epsilon \neq 0$. $\square$

Lemma 5. *For all $\alpha \in D^*$ given by (31),*

$$\lim_{\lambda\to 0}\lim_{\epsilon\to 0} M_f(\lambda, q^*_{\alpha,\epsilon,\lambda}) = \mathcal{E}_f(q^*(\alpha)).$$

Proof. Let $\alpha \in D^*$ and $\lambda > 0$. We have by definition of $D_{\alpha,\lambda}$, of D^* and using the link between $\tilde{i}_{\mathrm{RS}}$ and i_{RS} given by (104), that $0 \in D_{\alpha,\lambda}$. By Lemma 3 above, we have

$$q^*_{\alpha,\epsilon,\lambda} \xrightarrow[\epsilon\to 0,\ \epsilon\in D_{\alpha,\lambda}]{} q^*_{\alpha,0,\lambda} = q^*(\alpha).$$

Analogously to Proposition 22, $M_f(\lambda,\cdot)$ is continuous on $[0,\rho]$, thus $\lim_{\epsilon\to 0} M_f(\lambda, q^*_{\alpha,\epsilon,\lambda}) = M_f(\lambda, q^*(\alpha))$. And we obtain the result by taking $\lim_{\lambda\to 0} M_f(\lambda, q^*(\alpha)) = \mathcal{E}_f(q^*(\alpha))$, using that $M_f(\cdot, q)$ is continuous for $q \in [0,\rho]$ fixed (by Proposition 13) and by comparing (106) and (42). $\square$

In order to simplify the proof, we assume that $m = \alpha n$. By definition of the generalization error (41) and of the labels $\mathbf{Y}'$ given by (100),

$$\mathcal{E}_{f,n}(\alpha) := \mathrm{MMSE}(Y_1'|\mathbf{Y}, \mathbf{\Phi}, \mathbf{\Phi}').$$

Lemma 6 (Lower bound on the generalization error). *For all $\alpha \in D^*$,*

$$\liminf_{n\to\infty} \mathcal{E}_{f,n}(\alpha) \geq \mathcal{E}_f(q^*(\alpha)).$$

Proof. Let $\alpha \in D^*$, $\lambda > 0$ and $\epsilon \in D_{\alpha,\lambda} \setminus \{0\}$. Obviously,

$$\mathcal{E}_{f,n}(\alpha) \geq \mathrm{MMSE}(Y_1'|\mathbf{Y}, \mathbf{U}, \mathbf{\Phi}, \mathbf{\Phi}') \xrightarrow[n\to\infty]{} M_f(\lambda, q^*_{\alpha,\epsilon,\lambda}),$$

where we used Lemma 4. Consequently $\liminf_{n\to\infty} \mathcal{E}_{f,n}(\alpha) \geq M_f(\lambda, q^*_{\alpha,\epsilon,\lambda})$ and we obtain the lower bound by letting $\epsilon, \lambda \to 0$ and using Lemma 5. $\square$

Let us now prove the converse upper bound.

Lemma 7. *There exists a constant $C > 0$ (that only depend on f) such that for all $\alpha, \lambda > 0$ and all $\epsilon \in D_{\alpha,\lambda}\setminus\{0\}$*

$$\limsup_{n\to\infty} \mathcal{E}_{f,n}(\alpha+\epsilon) \leq M_f(\lambda, q^*_{\alpha,\epsilon,\lambda}) + C\lambda.$$

Proof. We will let the signal-to-noise ratio (snr) of the observation of Y_1' go to zero. Let us denote by λ_1 this snr: $U_1 = \sqrt{\lambda_1}\, Y_1' + Z_1'$. We will let λ_1 go from λ to 0 while the other snr for the observations of U_μ for $\mu = 2, \dots, \epsilon n$ will remain equal to λ. Recall that we denote $\mathbf{U} = (U_\mu)_{\mu=1}^{\epsilon n}$. Using Proposition 9 from [53],

$$\left|\frac{\partial}{\partial \lambda_1}\mathrm{MMSE}(Y_1'|\mathbf{Y}, \mathbf{U}, \mathbf{\Phi}, \mathbf{\Phi}')\right| = \mathbb{E}\big[\mathrm{Var}(Y_1'|\mathbf{Y}, \mathbf{U}, \mathbf{\Phi}, \mathbf{\Phi}')^2\big] \leq \mathbb{E}\big[(Y_1')^4\big] \leq \|f\|_\infty^4.$$

We define $C := \|f\|_\infty^4$. Consequently, by the mean value theorem,

$$\big|\mathrm{MMSE}(Y_1'|\mathbf{Y}, \mathbf{U}, \mathbf{\Phi}, \mathbf{\Phi}') - \mathrm{MMSE}(Y_1'|\mathbf{Y}, (U_\mu)_{\mu=2}^{\epsilon n}, \mathbf{\Phi}, \mathbf{\Phi}')\big| \leq C\lambda. \tag{117}$$

Since $(U_\mu)_{\mu=2}^{\epsilon n}$ contains less information than $(\widetilde{Y}_\mu)_{\mu=2}^{\epsilon n}$ because of the additional Gaussian noise and the application of the function f, we have

$$\mathrm{MMSE}(Y_1'|\mathbf{Y},(U_\mu)_{\mu=2}^{\epsilon n},\mathbf{\Phi},\mathbf{\Phi}') \geq \mathrm{MMSE}(Y_1'|\mathbf{Y},(\widetilde{Y}_\mu)_{\mu=2}^{\epsilon n},\mathbf{\Phi},\mathbf{\Phi}') = \mathcal{E}_{f,n}(\alpha+\epsilon-1/n) \geq \mathcal{E}_{f,n}(\alpha+\epsilon)\,. \quad (118)$$

The last identity combined with (117) leads to

$$\mathrm{MMSE}(Y_1'|\mathbf{Y},\mathbf{U},\mathbf{\Phi},\mathbf{\Phi}') + C\lambda \geq \mathcal{E}_{f,n}(\alpha+\epsilon)\,. \quad (119)$$

By Lemma 4 we know that $\lim_{n\to\infty}\mathrm{MMSE}(Y_1'|\mathbf{Y},\mathbf{U},\mathbf{\Phi},\mathbf{\Phi}') = M_f(\lambda, q^*_{\alpha,\epsilon,\lambda})$. Thus we conclude by taking the limsup in the inequality above. □

Corollary 5 (Upper bound on the generalization error). *For all $\alpha\in D^*$,*

$$\limsup_{n\to\infty}\mathcal{E}_{f,n}(\alpha) \leq \mathcal{E}_f(q^*(\alpha))\,.$$

Proof. Let $\alpha\in D^*$, $\lambda>0$ and $\epsilon_1>0$ such that $\alpha-\epsilon_1\in D^*$. Since by Lemma 3 the set $D_{\alpha-\epsilon_1,\lambda}$ is dense in $\mathbb{R}_+$, we can find $\epsilon_2\in D_{\alpha-\epsilon_1,\lambda}$ such that $0<\epsilon_2\leq\epsilon_1$. Using Lemma 7 above, we have

$$\limsup_{n\to\infty}\mathcal{E}_{f,n}(\alpha-\epsilon_1+\epsilon_2) \leq M_f(\lambda, q^*_{\alpha-\epsilon_1,\epsilon_2,\lambda}) + C\lambda\,.$$

Now, using the fact that $\epsilon_2\leq\epsilon_1$ we have

$$\limsup_{n\to\infty}\mathcal{E}_{f,n}(\alpha) \leq \limsup_{n\to\infty}\mathcal{E}_{f,n}(\alpha-\epsilon_1+\epsilon_2) \leq M_f(\lambda, q^*_{\alpha-\epsilon_1,\epsilon_2,\lambda}) + C\lambda\,.$$

Now, by Lemma 5 we have

$$\lim_{\lambda\to 0}\lim_{\epsilon_2\to 0} M_f(\lambda, q^*_{\alpha-\epsilon_1,\epsilon_2,\lambda}) + C\lambda = \mathcal{E}_f(q^*(\alpha-\epsilon_1))$$

which leads to $\limsup_{n\to\infty}\mathcal{E}_{f,n}(\alpha)\leq\mathcal{E}_f(q^*(\alpha-\epsilon_1))$. We conclude by letting $\epsilon_1\to 0$ (recall that by Proposition 1 D^* is dense in $\mathbb{R}_+$ so it is possible to find $\epsilon_1>0$ arbitrary small such that $\alpha-\epsilon_1\in D^*$), using the continuity of $\mathcal{E}_f$ (by Proposition 22) and the continuity of q^* (by Proposition 1). □

Proof of Theorem 4: For the moment we have proven Theorem 4 when f is continuous and bounded. We are going to relax this assumption by approximation. Let $f:\mathbb{R}\to\mathbb{R}$ such that $\mathbb{E}[|f(Y_{\mathrm{new}})|^{2+\gamma}]$ remains bounded as n goes to infinity, for some $\gamma>0$. Let $\epsilon>0$. By density of the continuous and bounded functions in the space $L^2(\mathbb{R})$ equipped with the law of $Y^{(q)}\sim P_{\mathrm{out}}(\cdot\,|\,\sqrt{q}\,V+\sqrt{\rho-q}\,W)$ ($V,W\overset{\mathrm{iid}}{\sim}\mathcal{N}(0,1)$), we can find a continuous bounded function $\widetilde{f}:\mathbb{R}\to\mathbb{R}$ such that $\mathbb{E}[(f(Y^{(q)})-\widetilde{f}(Y^{(q)}))^2]\leq\epsilon$.

Lemma 8. *For all $q\in[0,\rho]$ (because the law of $Y^{(q)}$ does not depend on q), we have*

$$f(Y_{\mathrm{new}})-\widetilde{f}(Y_{\mathrm{new}}) \xrightarrow[n\to\infty]{(d)} f(Y^{(q)})-\widetilde{f}(Y^{(q)})\,. \quad (120)$$

Proof. Let $(\mathbf{A}_{\mathrm{new}},Z_{\mathrm{new}})\sim P_A\otimes\mathcal{N}(0,1)$ such that $Y_{\mathrm{new}}=\varphi(\mathbf{\Phi}_{\mathrm{new}}\cdot\mathbf{X}^*/\sqrt{n},\mathbf{A}_{\mathrm{new}})+\sqrt{\Delta}Z_{\mathrm{new}}$. By the central limit theorem (that we apply under (h1)-(h3) and using (h4))

$$\varphi\Big(\frac{\mathbf{\Phi}_{\mathrm{new}}\cdot\mathbf{X}^*}{\sqrt{n}},\mathbf{A}_{\mathrm{new}}\Big) \xrightarrow[n\to\infty]{(d)} \varphi(\sqrt{\rho}Z,\mathbf{A}_{\mathrm{new}})\,, \quad (121)$$

where $Z\sim\mathcal{N}(0,1)$ is independent from $\mathbf{A}_{\mathrm{new}}$. Under (h5.b) this proves Lemma 8, because in that case

$Y_{\rm new} = \varphi(\mathbf{\Phi}_{\rm new}\cdot \mathbf{X}^*/\sqrt{n}, \mathbf{A}_{\rm new})$ takes values in $\mathbb{N}$. Under (h5.a) we let $g:\mathbb{R}\to\mathbb{R}$ be a continuous bounded function and we write $h := f - \widetilde{f}$. Then

$$\mathbb{E}\big[g\circ h(Y_{\rm new})\big] = \mathbb{E}\Big[g\circ h\Big(\varphi\Big(\frac{\mathbf{\Phi}_{\rm new}\cdot \mathbf{X}^*}{\sqrt{n}}, \mathbf{A}_{\rm new}\Big) + \sqrt{\Delta}Z_{\rm new}\Big)\Big]$$
$$= \mathbb{E}\Big[\frac{1}{\sqrt{2\pi\Delta}}\int g\circ h(z)\exp\Big\{-\frac{1}{2\Delta}\Big(z-\varphi\Big(\frac{\mathbf{\Phi}_{\rm new}\cdot \mathbf{X}^*}{\sqrt{n}}, \mathbf{A}_{\rm new}\Big)\Big)^2\Big\}dz\Big]\,.$$

The function $x\mapsto \int g\circ h(z)\frac{e^{-\frac{1}{2\Delta}(z-x)^2}}{\sqrt{2\pi\Delta}}dz$ is continuous and bounded: (121) then gives that

$$\mathbb{E}\big[g\circ h(Y_{\rm new})\big] \xrightarrow[n\to\infty]{} \mathbb{E}\Big[\frac{1}{\sqrt{2\pi\Delta}}\int g\circ h(z)\exp\Big\{-\frac{1}{2\Delta}\Big(z-\varphi\big(\sqrt{\rho}Z, \mathbf{A}_{\rm new}\big)\Big)^2\Big\}dz\Big] = \mathbb{E}\big[g\circ h(Y^{(q)})\big],$$

which concludes the proof by the Portemanteau Theorem. □

The sequence $\big((f(Y_{\rm new})-\tilde{f}(Y_{\rm new}))^2\big)_{n\geq 0}$ is uniformly integrable because bounded in $L^{1+\gamma}$ with $\gamma>0$. Consequently, Lemma 8 above implies

$$\mathbb{E}\big[(f(Y_{\rm new})-\widetilde{f}(Y_{\rm new}))^2\big] \xrightarrow[n\to\infty]{} \big\|f(Y^{(q)})-\widetilde{f}(Y^{(q)})\big\|_{L^2}^2 = \mathbb{E}\big[(f(Y^{(q)})-\widetilde{f}(Y^{(q)}))^2\big] \leq \epsilon\,.$$

Therefore, we can find $n_0\in\mathbb{N}$ such that for all $n\geq n_0$, $\|f(Y_{\rm new})-\widetilde{f}(Y_{\rm new})\|_{L^2}^2 = \mathbb{E}[(f(Y_{\rm new})-\widetilde{f}(Y_{\rm new}))^2] \leq 2\epsilon$. If we now apply Theorem 4 for $\widetilde{f}$, we can find $n_1\geq n_0$ such that for all $n\geq n_1$, $|\mathcal{E}_{\widetilde{f},n}^{1/2} - \mathcal{E}_{\widetilde{f}}(q^*(\alpha))^{1/2}| \leq \sqrt{\epsilon}$. Let $n\geq 1$, and compute

$$\Big|\mathcal{E}_{f,n}^{1/2} - \mathcal{E}_{\widetilde{f},n}^{1/2}\Big| = \Big|\big\|f(Y_{\rm new}) - \mathbb{E}[f(Y_{\rm new})|\mathbf{Y},\mathbf{\Phi},\mathbf{\Phi}_{\rm new}]\big\|_{L^2} - \big\|\widetilde{f}(Y_{\rm new}) - \mathbb{E}[\widetilde{f}(Y_{\rm new})|\mathbf{Y},\mathbf{\Phi},\mathbf{\Phi}_{\rm new}]\big\|_{L^2}\Big|$$
$$\leq \big\|f(Y_{\rm new})-\widetilde{f}(Y_{\rm new})\big\|_{L^2} + \big\|\mathbb{E}[f(Y_{\rm new})-\widetilde{f}(Y_{\rm new})|\mathbf{Y},\mathbf{\Phi},\mathbf{\Phi}_{\rm new}]\big\|_{L^2}$$
$$\leq 2\big\|f(Y_{\rm new})-\widetilde{f}(Y_{\rm new})\big\|_{L^2} \leq 2\sqrt{2\epsilon}\,,$$

where we successively used the triangular inequality twice for the first inequality ($||a-b|-|x-y|| \leq |a-x+y-b| \leq |a-x|+|y-b|$) and Jensen's inequality for the second. By the same arguments we have also $|\mathcal{E}_f(q)^{1/2} - \mathcal{E}_{\widetilde{f}}(q)^{1/2}| \leq 2\sqrt{\epsilon}$ for all $q\in[0,\rho]$. We conclude that for all $n\geq n_1$,

$$|\mathcal{E}_{f,n}^{1/2} - \mathcal{E}_f(q^*(\alpha))^{1/2}| \leq \big|\mathcal{E}_{f,n}^{1/2} - \mathcal{E}_{\widetilde{f},n}^{1/2}\big| + \big|\mathcal{E}_{\widetilde{f}}(q^*(\alpha))^{1/2} - \mathcal{E}_f(q^*(\alpha))^{1/2}\big| + \big|\mathcal{E}_{\widetilde{f},n}^{1/2} - \mathcal{E}_{\widetilde{f}}(q^*(\alpha))^{1/2}\big|$$
$$\leq (2\sqrt{2}+3)\sqrt{\epsilon}\,, \tag{122}$$

which proves Theorem 4. □

5.2 Generalization error of GAMP: Proof of Proposition 2

Let us decompose:

$$\mathcal{E}_{\rm gen}^{\rm GAMP,\mathit{t}} := \mathbb{E}\big[\big(Y_{\rm new} - \widehat{Y}^{\rm GAMP,\mathit{t}}\big)^2\big] = \mathbb{E}\big[Y_{\rm new}^2\big] + \mathbb{E}\big[\big(\widehat{Y}^{\rm GAMP,\mathit{t}}\big)^2\big] - 2\mathbb{E}\big[Y_{\rm new}\widehat{Y}^{\rm GAMP,\mathit{t}}\big]\,. \tag{123}$$

Lemma 9. *We have*

$$\mathbb{E}\big[Y_{\rm new}\widehat{Y}^{\rm GAMP,\mathit{t}}\big] \xrightarrow[n\to\infty]{} \mathbb{E}_V\Big[\mathbb{E}_W\Big[\int dY\,Y P_{\rm out}(Y|\sqrt{q^t}\,V + \sqrt{\rho-q^t}\,W)\Big]^2\Big]\,. \tag{124}$$

Proof. Start by writing

$$\mathbb{E}\big[Y_{\text{new}}\widehat{Y}^{\text{GAMP},t}\big] = \mathbb{E}\int y\, y'\, P_{\text{out}}\Big(y\Big|\frac{\mathbf{\Phi}_{\text{new}}\cdot\mathbf{X}^*}{\sqrt{n}}\Big) P_{\text{out}}\Big(y'\Big|\frac{\mathbf{\Phi}_{\text{new}}\cdot\widehat{\mathbf{x}}^t}{\sqrt{n}}+\sqrt{\rho-q^t}\,W\Big)dydy'$$

where $W\sim\mathcal{N}(0,1)$ is independent of everything else. $\mathbf{\Phi}_{\text{new}}\sim\mathcal{N}(0,\mathbf{I}_n)$ is independent of $\mathbf{X}^*$ and $\widehat{\mathbf{x}}^t$, so, conditionally on $\mathbf{X}^*,\widehat{\mathbf{x}}^t$ we have

$$\Big(\frac{\mathbf{\Phi}_{\text{new}}\cdot\mathbf{X}^*}{\sqrt{n}},\frac{\mathbf{\Phi}_{\text{new}}\cdot\widehat{\mathbf{x}}^t}{\sqrt{n}}\Big)\sim\mathcal{N}\Big(0,\frac{1}{n}\begin{pmatrix}\|\mathbf{X}^*\|^2 & \widehat{\mathbf{x}}^t\cdot\mathbf{X}^*\\ \widehat{\mathbf{x}}^t\cdot\mathbf{X}^* & \|\widehat{\mathbf{x}}^t\|^2\end{pmatrix}\Big).$$

We assumed that (50) holds, i.e. $\mathbf{X}^*\cdot\widehat{\mathbf{x}}^t/n\to q^t$ and $\|\widehat{\mathbf{x}}^t\|^2/n\to q^t$, in probability. By the law of large numbers $\|\mathbf{X}^*\|^2/n\to\rho$ in probability. Consequently,

$$\Big(\frac{\mathbf{\Phi}_{\text{new}}\cdot\mathbf{X}^*}{\sqrt{n}},\frac{\mathbf{\Phi}_{\text{new}}\cdot\widehat{\mathbf{x}}^t}{\sqrt{n}}\Big)\xrightarrow[n\to\infty]{(d)}\mathcal{N}\Big(0,\begin{pmatrix}\rho & q^t\\ q^t & q^t\end{pmatrix}\Big).$$

Since $x\mapsto P_{\text{out}}(\cdot|x)$ is continuous almost everywhere for the Wasserstein distance of order 2, the function $h:(a,b)\mapsto\mathbb{E}_W\int yy'P_{\text{out}}(y|a)P_{\text{out}}(y'|b+\sqrt{\rho-q^t}\,W)dydy'$ with $W\sim\mathcal{N}(0,1)$ is continuous almost everywhere. Therefore

$$H_n := h\Big(\frac{\mathbf{\Phi}_{\text{new}}\cdot\mathbf{X}^*}{\sqrt{n}},\frac{\mathbf{\Phi}_{\text{new}}\cdot\widehat{\mathbf{x}}^t}{\sqrt{n}}\Big)\xrightarrow[n\to\infty]{(d)}h(\sqrt{q^t}\,Z_0+\sqrt{\rho-q^t}\,Z_1,\sqrt{q^t}\,Z_0)\,, \tag{125}$$

where $Z_0,Z_1\overset{\text{iid}}{\sim}\mathcal{N}(0,1)$. We have by Jensen's inequality

$$\begin{aligned}\mathbb{E}\big[|H_n|^{1+\eta}\big]&\le\mathbb{E}\big[\big|Y_{\text{new}}\widehat{Y}^{\text{GAMP},t}\big|^{1+\eta}\big]\le\mathbb{E}\Big[\Big(\frac{1}{2}Y_{\text{new}}^2+\frac{1}{2}(\widehat{Y}^{\text{GAMP},t})^2\Big)^{1+\eta}\Big]\\&\le\frac{1}{2}\mathbb{E}|Y_1|^{2+2\eta}+\frac{1}{2}\mathbb{E}\big|\widehat{Y}^{\text{GAMP},t}\big|^{2+2\eta}.\end{aligned}$$

By assumption, there exists $\eta>0$ such that the two last terms above remain bounded with n: H_n is therefore bounded in $L^{1+\eta}$ and is therefore uniformly integrable. From (125) we thus get

$$\begin{aligned}\mathbb{E}\big[Y_{\text{new}}\widehat{Y}^{\text{GAMP},t}\big]=\mathbb{E}[H_n]&\xrightarrow[n\to\infty]{}\mathbb{E}\big[h(\sqrt{q^t}\,Z_0+\sqrt{\rho-q^t}\,Z_1,\sqrt{q^t}\,Z_0)\big]\\&=\mathbb{E}_V\Big[\mathbb{E}_W\Big[\int dY\,Y P_{\text{out}}(Y|\sqrt{q^t}\,V+\sqrt{\rho-q^t}\,W)\Big]^2\Big].\end{aligned}$$

□

Following the arguments of Lemma 9 one can also show that

$$\begin{aligned}\mathbb{E}\big[(\widehat{Y}^{\text{GAMP},t})^2\big]&\xrightarrow[n\to\infty]{}\mathbb{E}_V\Big[\mathbb{E}_W\Big[\int dY\,Y P_{\text{out}}(Y|\sqrt{q^t}\,V+\sqrt{\rho-q^t}\,W)\Big]^2\Big],\\\mathbb{E}\big[Y_{\text{new}}^2\big]&\xrightarrow[n\to\infty]{}\mathbb{E}_V\int dY\,Y^2P_{\text{out}}(Y|\sqrt{\rho}\,V).\end{aligned}$$

This proves (together with (123) and Lemma 9) Proposition 2.

5.3 Limit of the overlap: Proof of Theorem 2

Recall the definition of the overlap (86): $Q_n := \mathbf{X}^*\cdot\mathbf{x}/n$, where $\mathbf{x}=(x_1,\dots,x_n)$ is a sample from the posterior distribution $P(\mathbf{X}^*|\,\mathbf{Y},\mathbf{\Phi})$, independently of everything else. In this section we will show that $|Q_n|$

converges in probability to $q^*(\alpha)$, when $\alpha \in D^*$ given by (31). We will first show an upper-bound in Sec. 5.3.1 below, before proving the converse lower-bound in Sec. 5.3.2.

5.3.1 Upper bound on the overlap

Proposition 9 (Upper bound on the overlap). *For all $\alpha \in D^*$ and for all $\epsilon > 0$,*

$$\mathbb{P}\big(|Q_n| \geq q^*(\alpha) + \epsilon\big) \xrightarrow[n\to\infty]{} 0\,.$$

Let us fix $\alpha \in D^*$ and let $p \geq 1$. In order to obtain an upper bound on the overlap, we consider an observation model with some (small) extra information (that takes the form of a tensor of order $2p$) in addition of the original model (2), i.e. we observe

$$\begin{cases} \mathbf{Y} \sim P_{\rm out}(\cdot \,|\, \mathbf{\Phi}\mathbf{X}^*/\sqrt{n})\,, \\ \mathbf{Y}' = \sqrt{\frac{\lambda}{n^{2p-1}}}(\mathbf{X}^*)^{\otimes 2p} + \mathbf{Z}'\,, \end{cases} \tag{126}$$

where $\lambda \geq 0$, $\mathbf{Z}' = (Z'_{i_1\dots i_{2p}})_{1\leq i_1,\dots,i_{2p}\leq n} \overset{\rm iid}{\sim} \mathcal{N}(0,1)$ and $(\mathbf{X}^*)^{\otimes 2p} = (X_{i_1}\dots X_{i_{2p}})_{1\leq i_1,\dots,i_{2p}\leq n}$. In order to prove Proposition 9 we need the two results below, which are proven after the proof of Proposition 9.

Proposition 10 (Mutual information of the perturbed model). *For all $\lambda \geq 0$, the mutual information for model (126) verifies*

$$\lim_{n\to\infty} \frac{1}{n} I\big(\mathbf{X}^*; \mathbf{Y}, \mathbf{Y}'\big|\mathbf{\Phi}\big) = I(\lambda)\,, \tag{127}$$

where the right-hand-side is

$$I(\lambda) := \inf_{q\in[0,\rho]} \sup_{r\geq 0} \Big\{ I_{P_0}(r + 2p\lambda q^{2p-1}) + \alpha \mathcal{I}_{P_{\rm out}}(q) - \frac{r}{2}(\rho - q) + \frac{2p-1}{2}\lambda q^{2p} - \rho p \lambda q^{2p-1} + \frac{\lambda}{2}\rho^{2p}\Big\}. \tag{128}$$

Lemma 10. *The function I defined above by (128) is concave on $\mathbb{R}_+$. Its left- and right-derivatives are given by*

$$I'(\lambda^+) = \min\left\{\frac{1}{2}\big(\rho^{2p} - q_*(\lambda)^{2p}\big) \,\Big|\, q_*(\lambda) \text{ achieves the infimum in (128)}\right\},$$
$$I'(\lambda^-) = \max\left\{\frac{1}{2}\big(\rho^{2p} - q_*(\lambda)^{2p}\big) \,\Big|\, q_*(\lambda) \text{ achieves the infimum in (128)}\right\}.$$

We are now in position to prove Proposition 9.

Proof of Proposition 9: By the I-MMSE relation of Proposition 13,

$$\frac{1}{n}\frac{\partial}{\partial\lambda} I\big(\mathbf{X}^*; \mathbf{Y}, \mathbf{Y}'\big|\mathbf{\Phi}\big) = \frac{1}{n}\frac{\partial}{\partial\lambda} I\big((\mathbf{X}^*)^{\otimes 2p}; \mathbf{Y}, \mathbf{Y}'\big|\mathbf{\Phi}\big) = \frac{1}{2n^{2p}} \mathrm{MMSE}\big((\mathbf{X}^*)^{\otimes 2p}\big|\mathbf{Y}, \mathbf{Y}', \mathbf{\Phi}\big)\,.$$

Using Proposition 10 and Lemma 10 above we obtain by concavity that

$$\frac{1}{2n^{2p}} \mathrm{MMSE}\big((\mathbf{X}^*)^{\otimes 2p}\big|\mathbf{Y}, \mathbf{Y}', \mathbf{\Phi}\big) = \frac{1}{n}\frac{\partial}{\partial\lambda} I\big(\mathbf{X}^*; \mathbf{Y}, \mathbf{Y}'\big|\mathbf{\Phi}\big) \xrightarrow[n\to\infty]{} I'(\lambda) = \frac{1}{2}\big(\rho^{2p} - q_*(\lambda)^{2p}\big)\,,$$

for all $\lambda > 0$ for which the infimum of (128) is achieved at a unique $q_*(\lambda)$. Consequently,

$$\liminf_{n\to\infty} \frac{1}{n^{2p}} \mathrm{MMSE}\big((\mathbf{X}^*)^{\otimes 2p}\big|\mathbf{Y}, \mathbf{\Phi}\big) \geq \liminf_{n\to\infty} \frac{1}{n^{2p}} \mathrm{MMSE}\big((\mathbf{X}^*)^{\otimes 2p}\big|\mathbf{Y}, \mathbf{Y}', \mathbf{\Phi}\big) = \rho^{2p} - q_*(\lambda)^{2p}\,. \tag{129}$$

Let us now suppose that $\alpha \in D^*$. In that case, there exists a unique $q_*(\lambda = 0) = q^*(\alpha)$ that achieves the infimum in (128). Consequently, $I'(0^+) = \frac{1}{2}(\rho^{2p} - q^*(\alpha)^{2p})$. By concavity, $I'(\lambda) \to I'(0^+)$ as $\lambda \to 0$, which gives $q_*(\lambda) \to q^*(\alpha)$. By taking the $\lambda \to 0$ limit in (129) above we get

$$\liminf_{n\to\infty} \frac{1}{n^{2p}} \mathrm{MMSE}\big((\mathbf{X}^*)^{\otimes 2p} \big| \mathbf{Y}, \mathbf{\Phi}\big) \geq \rho^{2p} - q^*(\alpha)^{2p} \,.$$

One verifies easily that

$$\frac{1}{n^{2p}} \mathrm{MMSE}\big((\mathbf{X}^*)^{\otimes 2p} \big| \mathbf{Y}, \mathbf{\Phi}\big) = \rho^{2p} - \mathbb{E}\big[Q_n^{2p}\big] + o_n(1) \,, \tag{130}$$

so we deduce that

$$\limsup_{n\to\infty} \mathbb{E}\big[Q_n^{2p}\big] \leq q^*(\alpha)^{2p} \,.$$

Let $\epsilon > 0$. By Markov's inequality we have

$$\mathbb{P}\big(|Q_n| \geq q_*(\alpha) + \epsilon\big) \leq \frac{\mathbb{E}\big[Q_n^{2p}\big]}{(q_*(\alpha) + \epsilon)^{2p}} \,.$$

By taking the $\limsup$ in n on both sides we obtain

$$\limsup_{n\to\infty} \mathbb{P}\big(|Q_n| \geq q_*(\alpha) + \epsilon\big) \leq \frac{q^*(\alpha)^{2p}}{(q_*(\alpha) + \epsilon)^{2p}} \,,$$

and Proposition 9 follows by taking the $p \to \infty$ limit in the inequality above. □

We now prove the two preliminary results used in the proof of Proposition 9.

Proof of Proposition 10: The proof is very similar to the one of Theorem 1 (and Corollary 1), by the adaptive interpolation method (see Sec. 4), so we provide only the main arguments and omit to write the small perturbation (i.e. the ϵ_1, ϵ_2 present in Sec. 4) for simplicity.

In order to tackle model (126) we need first to study a simpler one, namely when we have access to the simultaneous observations $\mathbf{Y} \sim P_{\mathrm{out}}(\cdot \mid \mathbf{\Phi}\mathbf{X}^*/\sqrt{n})$ and $\mathbf{Y}'' = \sqrt{\gamma}\, \mathbf{X}^* + \mathbf{Z}''$. Define, for $\gamma \geq 0$, the free entropy (expected log-partition function) of this model:

$$F_n(\gamma) := \frac{1}{n} \mathbb{E} \ln \int dP_0(\mathbf{x}) \exp\Big(\sum_{i=1}^n \sqrt{\gamma} Z''_i x_i + \gamma x_i X_i^* - \frac{\gamma}{2} x_i^2\Big) \prod_{\mu=1}^m P_{\mathrm{out}}\Big(Y_\mu \Big| \frac{1}{\sqrt{n}} \mathbf{\Phi}_\mu \cdot \mathbf{x}\Big) \,, \tag{131}$$

where $Z''_i \overset{\text{iid}}{\sim} \mathcal{N}(0,1)$ are independent of everything else. Let us define

$$F_{\mathrm{RS}}(\gamma) := \sup_{q \in [0,\rho]} \inf_{r \geq 0} \Big\{ \psi_{P_0}(r + \gamma) + \alpha \Psi_{P_{\mathrm{out}}}(q) - \frac{rq}{2} \Big\} \,. \tag{132}$$

A slight and easy modification of the Theorem 1 gives that for all $\gamma \geq 0$

$$F_n(\gamma) \xrightarrow[n\to\infty]{} F_{\mathrm{RS}}(\gamma) \,. \tag{133}$$

F_n is a convex function of γ (this can be checked by relating it to the mutual information like in Corollary 1 and then using the I-MMSE relation of Proposition 13), thus F_{RS} is too. The function F_{RS} is therefore continuous on $\mathbb{R}_+$. F_n is also a non-decreasing function of γ (this is again checked using the I-MMSE relation). By Dini's second theorem we obtain that the convergence of (133) is uniform over all compact subsets of $\mathbb{R}_+$.

Now that we have studied this simpler model, we come back to the analysis of (126). We proceed by interpolation as in Sec. 4.1. Let $q : [0,1] \to [0,\rho]$ be a continuous interpolating function. For $t \in [0,1]$, consider the following "interpolating estimation model":

$$\begin{cases} Y_\mu & \sim \quad P_{\rm out}(\,\cdot\,|\,\mathbf{\Phi}\mathbf{X}^*/\sqrt{n})\,, & 1 \le \mu \le m\,, \\ \mathbf{Y}'_t & = \quad \sqrt{\frac{\lambda(1-t)}{n^{2p-1}}}(\mathbf{X}^*)^{\otimes 2p} + \mathbf{Z}'\,, & \\ Y''_{t,i} & = \quad \sqrt{2p\lambda\int_0^t q(v)^{2p-1}dv}\, X_i^* + Z''_i\,, & 1 \le i \le n\,. \end{cases} \tag{134}$$

Define the corresponding interpolating free entropy:

$$f_n(t) := \frac{1}{n}\mathbb{E}\ln \int dP_0(\mathbf{x}) e^{H_{n,t}(\mathbf{x})} \prod_{\mu=1}^m P_{\rm out}\Big(Y_\mu\Big|\frac{1}{\sqrt{n}}\mathbf{\Phi}_\mu\cdot\mathbf{x}\Big)\,,$$

where the Hamiltonian of the model is

$$\begin{aligned} H_{n,t}(\mathbf{x}) = \sum_{i=1}^n \Big\{\sqrt{2p\lambda\int_0^t q(v)^{2p-1}dv}\, Z''_i x_i + 2p\lambda\int_0^t q(v)^{2p-1}dv\, x_i X_i^* - p\lambda\int_0^t q(v)^{2p-1}dv\, x_i^2\Big\} \\ + \sum_{i_1,\dots,i_{2p}} \Big\{\sqrt{\frac{\lambda(1-t)}{n^{2p-1}}} Z'_{i_1\dots i_{2p}} x_{i_1}\dots x_{i_{2p}} + \frac{\lambda(1-t)}{n^{2p-1}} x_{i_1}\dots x_{i_{2p}} X^*_{i_1}\dots X^*_{i_{2p}} - \frac{\lambda(1-t)}{2n^{2p-1}} x^2_{i_1}\dots x^2_{i_{2p}}\Big\}\,. \end{aligned}$$

We aim at computing $f_n := f_n(0)$. We have $f_n(1) = F_n(2p\lambda\int_0^1 q(t)^{2p-1}dt)$. Similarly to Proposition 3 one can compute (see [41] where this computation is done):

$$f'_n(t) = -\frac{\lambda}{2}\mathbb{E}\big\langle Q_t^{2p} - 2pq(t)^{2p-1}Q_t\big\rangle_t \tag{135}$$

where $Q_t = \sum_{i=1}^n X_i^* x_i/n$ is the overlap between the planted solution $\mathbf{X}^*$ and $\mathbf{x} = (x_1,\dots,x_n)$, a sample from the posterior distribution $P(\mathbf{X}^*|\mathbf{Y},\mathbf{Y}'_t,\mathbf{Y}''_t)$. Similarly as in Sec. 4.1 the Gibbs bracket $\langle - \rangle_t$ denotes the expectation w.r.t. this t-dependent posterior acting on $\mathbf{x}$, $\mathbb{E}$ is w.r.t. the quenched variables $\mathbf{Y},\mathbf{Y}'_t,\mathbf{Y}''_t$. By convexity of the function $x \mapsto x^{2p}$, we have for all $a,b \in \mathbb{R}$, $a^{2p} - 2pab^{2p-1} \ge (1-2p)b^{2p}$. Consequently, if we choose q to be a constant function, i.e. $q(t) = q$ for all $t \in [0,1]$, we have

$$f'_n(t) \le \frac{\lambda}{2}(2p-1)q^{2p}\,.$$

This gives

$$f_n = f_n(0) = f_n(1) - \int_0^1 f'_n(t)dt \ge F_n(2p\lambda q^{2p-1}) - \frac{\lambda}{2}(2p-1)q^{2p}\,.$$

By taking the $\liminf$ in n on both sides, we obtain $\liminf_{n\to\infty} f_n \ge F_{\rm RS}(2p\lambda q^{2p-1}) - \frac{\lambda}{2}(2p-1)q^{2p}$ using (133) and since this holds for all $q \in [0,\rho]$ we get

$$\liminf_{n\to\infty} f_n \ge \sup_{q\in[0,\rho]}\Big\{F_{\rm RS}(2p\lambda q^{2p-1}) - \frac{\lambda}{2}(2p-1)q^{2p}\Big\}\,.$$

Let us now prove the converse upper-bound. One can show as in Sec. 4.3 that the overlap Q_t concentrates around its expectation: Proposition 4 applies. This perturbation does not change the free entropy in the limit $n \to \infty$ nor the following derivation, so we do not track it explicitely for the sake of simplicity. Let us go back to (135). Therefore, using this concentration and then choosing $q(t) = \tilde{q}(t) = \mathbb{E}\langle Q_t\rangle_t$ as done in Sec. 4.4, we

obtain that

$$f_n'(t) = \frac{\lambda}{2}(2p-1)\tilde{q}(t)^{2p} + o_n(1)\,.$$

Consequently,

$$\begin{aligned} f_n &= f_n(1) - \int_0^1 f_n'(t)dt = F_n\Big(2p\lambda \int_0^1 \tilde{q}(t)^{2p-1}dt\Big) - \frac{\lambda}{2}(2p-1)\int_0^1 \tilde{q}(t)^{2p}dt + o_n(1) \\ &\leq F_n\Big(2p\lambda \int_0^1 \tilde{q}(t)^{2p-1}dt\Big) - \frac{\lambda}{2}(2p-1)\Big(\int_0^1 \tilde{q}(t)^{2p-1}dt\Big)^{\frac{2p}{2p-1}} + o_n(1) \\ &\leq \sup_{q\in[0,\rho]} \Big\{F_n(2p\lambda q^{2p-1}) - \frac{\lambda}{2}(2p-1)q^{2p}\Big\} + o_n(1)\,. \end{aligned}$$

We use now the fact that the convergence in (133) is uniform over all compact sets to get the upper-bound: $\limsup_{n\to\infty} f_n \leq \sup_{q\in[0,\rho]} \{F_{\mathrm{RS}}(2p\lambda q^{2p-1}) - \frac{\lambda}{2}(2p-1)q^{2p}\}$. We conclude that

$$\lim_{n\to\infty} f_n = \sup_{q\in[0,\rho]} \Big\{F_{\mathrm{RS}}(2p\lambda q^{2p-1}) - \frac{\lambda}{2}(2p-1)q^{2p}\Big\}\,. \tag{136}$$

We are now going to simplify the right-hand side of the above equation.

Lemma 11. *F_{RS} is a convex function on $\mathbb{R}_+$, whose left- and right-derivatives at $\gamma \geq 0$ are:*

$$\begin{aligned} F_{\mathrm{RS}}'(\gamma^+) &= \max\Big\{\frac{1}{2}q_*(\gamma)\,\Big|\, q_*(\gamma) \text{ achieves the supremum in } (132)\Big\}\,, \\ F_{\mathrm{RS}}'(\gamma^-) &= \min\Big\{\frac{1}{2}q_*(\gamma)\,\Big|\, q_*(\gamma) \text{ achieves the supremum in } (132)\Big\}\,. \end{aligned}$$

In particular, F_{RS} is differentiable at $\gamma \geq 0$ if and only if the supremum in (132) is achieved at a unique $q_*(\gamma)$.

Proof. We already know that F_{RS} is convex (as a limit of convex functions, see (133)). We have

$$F_{\mathrm{RS}}(\gamma) = \sup_{q\in[0,\rho]} \inf_{r\geq 0}\Big\{\psi_{P_0}(r+\gamma) + \alpha\Psi_{P_{\mathrm{out}}}(q) - \frac{rq}{2}\Big\} = \sup_{q\in[0,\rho]} \Big\{\alpha\Psi_{P_{\mathrm{out}}}(q) - g(\gamma, q/2)\Big\} \tag{137}$$

where $g(\gamma,x) = \sup_{r\geq 0}\{xr - \psi_{P_0}(\gamma+r)\}$ is the Legendre transform of $r \mapsto \psi_{P_0}(\gamma+r)$. Let us now compute $\frac{\partial g}{\partial \gamma}(\gamma,x)$. If $x \leq \psi_{P_0}'(\gamma)$, then the supremum in r is achieved at $r=0$, $g(x,\gamma) = -\psi_{P_0}(\gamma)$. If now $x > \psi_{P_0}'(\gamma)$ then

$$g(\gamma,x) = \sup_{r\geq -\gamma}\{xr - \psi_{P_0}(\gamma+r)\} = -\gamma x + \sup_{r\geq 0}\{xr - \psi_{P_0}(r)\}\,.$$

The first equality comes from the fact that the supremum can not be achieved on $[-\gamma,0]$ because for all $r \in [-\gamma,0]$, $x > \psi_{P_0}'(\gamma) \geq \psi_{P_0}'(\gamma+r)$. We obtain

$$g(\gamma,x) = \begin{cases} -\psi_{P_0}(\gamma) & \text{if } x \leq \psi_{P_0}'(\gamma)\,, \\ -x\gamma + g(0,x) & \text{if } x > \psi_{P_0}'(\gamma)\,. \end{cases}$$

From there, we conclude that $\frac{\partial g}{\partial \gamma}(\gamma,x) = -\max\big(\psi_{P_0}'(\gamma), x\big)$. By Lemma 23, every optimal couple $(q_*(\gamma), r_*(\gamma))$ satisfy $q_*(\gamma) = 2\psi_{P_0}'(\gamma + r_*(\gamma))$. This implies (by convexity of ψ_{P_0}) that $q_*(\gamma)/2 \geq \psi_{P_0}'(\gamma)$. Using Corollary 4 from [52] F_{RS} we get that

$$F_{\mathrm{RS}}'(\gamma^+) = \max\Big\{-\frac{\partial g}{\partial \gamma}(\gamma, q_*(\gamma))\,\Big|\, q_*(\gamma) \text{ maximizer of } (132)\Big\} = \max\Big\{\frac{1}{2}q_*(\gamma)\,\Big|\, q_*(\gamma) \text{ maximizer of } (132)\Big\}$$

and analogously for $F'_{\mathrm{RS}}(\gamma^-)$. □

Lemma 12. *We have*

$$\sup_{\tilde{q}\in[0,\rho]} \left\{F_{\mathrm{RS}}(2p\lambda\tilde{q}^{2p-1})-(2p-1)\frac{\lambda}{2}\tilde{q}^{2p}\right\} = \sup_{q\in[0,\rho]}\inf_{r\geq 0}\left\{\psi_{P_0}(r+2p\lambda q^{2p-1})+\alpha\Psi_{P_{\mathrm{out}}}(q)-\frac{rq}{2}-(2p-1)\frac{\lambda}{2}q^{2p}\right\}.$$

Proof. Consider the equality above. The inequality l.h.s $\geq$ r.h.s. is obvious because it suffices to restrict the supremum over $(q,\tilde{q})\in[0,\rho]^2$ to the supremum over the couples (q,q) for $q\in[0,\rho]$.

Let us prove now the converse inequality. Let us do the change of variable $x=\tilde{q}^{2p-1}$ and define $H(x):=F_{\mathrm{RS}}(2p\lambda x)-(2p-1)\frac{\lambda}{2}x^{2p/(2p-1)}$. F_{RS} is left- and right-differentiable everywhere, so is H. We have

$$H'(x)=2p\lambda F'_{\mathrm{RS}}(2p\lambda x)-\frac{\lambda}{2}2px^{1/(2p-1)}=p\lambda\big(2F'_{\mathrm{RS}}(2p\lambda x)-x^{1/(2p-1)}\big) \tag{138}$$

at the points at which H is differentiable, and analogously for the left- and right-derivatives of H. Let $x\in[0,\rho^{2p-1}]$ be a point at which H achieves its supremum over $[0,\rho^{2p-1}]$. Let us distinguish 3 cases:

- Case 1: $x=0$. In that case, we have $H'(0^+)\leq 0$ and thus $F'_{\mathrm{RS}}(0^+)\leq 0$. Using Lemma 11, we obtain that the only $q\in[0,\rho]$ that achieves the supremum in (132) is $q=0=x^{1/(2p-1)}$.
- Case 2: $0<x<\rho^{2p-1}$. We have then $H'(x^-)\geq 0$ and $H'(x^+)\leq 0$. Using (138), we deduce that

  $$2F'_{\mathrm{RS}}\big((2p\lambda x)^+\big)\leq x^{1/(2p-1)}\leq 2F'_{\mathrm{RS}}\big((2p\lambda x)^-\big).$$

  F_{RS} is convex, so the above inequalities collapses into equalities and we get that F_{RS} is differentiable at $2p\lambda x$ with derivative given by $F'_{\mathrm{RS}}(2p\lambda x)=x^{1/(2p-1)}/2$. Lemma 11 above gives then that the supremum in (132) is achieved uniquely at $q=x^{1/(2p-1)}$.
- Case 3: $x=\rho^{2p-1}$. Using the same arguments than in Case 1, we obtain also $q=x^{1/(2p-1)}$.

Conclusion: In all 3 cases above, $q=x^{1/(2p-1)}$ achieves the supremum in (132). Recall that we used the change of variable $x=\tilde{q}^{2p-1}$. Consequently, if $\tilde{q}\in[0,\rho]$ achieves the supremum of $\tilde{q}\mapsto F_{\mathrm{RS}}(2p\lambda\tilde{q}^{2p-1})-(2p-1)\lambda\tilde{q}^{2p}$, then $\tilde{q}$ achieves also the supremum in (132). This proves the converse bound. □

By Lemma 12 and (136) above, we get that

$$f_n\xrightarrow[n\to\infty]{}\sup_{q\in[0,\rho]}\inf_{r\geq 0}\left\{\psi_{P_0}(r+2p\lambda q^{2p-1})+\alpha\Psi_{P_{\mathrm{out}}}(q)-\frac{rq}{2}-(2p-1)\frac{\lambda}{2}q^{2p}\right\}.$$

Proposition 10 follows then by rewriting the above limit in terms of mutual information, as we did to deduce Corollary 1 from Theorem 1. □

Proof of Lemma 10: The proof follows exactly the same steps than the one of Lemma 11, so we omit it for the sake brevity. □

5.3.2 Lower bound using the generalization error

Let us fix $\alpha\in D^*$. The sequence of the overlaps $\big(Q_n\big)_{n\geq 1}$ is tight (because bounded in L^1). By Prokhorov's Theorem we know that the sequence of the laws of $\big(Q_n\big)_{n\geq 1}$ is relatively compact. We can thus consider a subsequence along which it converges in law, to some random variable Q. In order to simplify the notations (and because working with an extraction does not change the proof) we will assume in the sequel that

$$Q_n\xrightarrow[n\to\infty]{(d)}Q,$$

for some random variable Q. We aim now at showing that $|Q| = q^*(\alpha)$ almost-surely.

Lemma 13 (Upper bound on the overlap). *$|Q| \leq q^*(\alpha)$ almost-surely.*

Proof. Let $\epsilon > 0$. The set $[0, q^*(\alpha)+\epsilon]$ is closed, so by Portemanteau's Theorem

$$\mathbb{P}\big(|Q| \leq q^*(\alpha)+\epsilon\big) \geq \limsup_{n\to\infty} \mathbb{P}\big(|Q_n| \leq q^*(\alpha)+\epsilon\big) = 1\,,$$

by Proposition 9. So $\mathbb{P}\big(|Q| \leq q^*(\alpha)+\epsilon\big) = 1$ for all $\epsilon > 0$ which gives $\mathbb{P}\big(|Q| \leq q^*(\alpha)\big) = 1$. □

We are going to prove the converse lower bound using Theorem 4. Let $f : \mathbb{R} \to \mathbb{R}$ be a continuous bounded function. Theorem 4 gives $\mathcal{E}_{f,n}(\alpha) \xrightarrow[n\to\infty]{} \mathcal{E}_f(q^*(\alpha))$. The function $\mathcal{E}_f$ can be written as

$$\mathcal{E}_f(q) = \frac{1}{2}\mathbb{E}\Big[h_f\big(\sqrt{q}Z_0 + \sqrt{\rho-q}Z_1, \sqrt{q}Z_0 + \sqrt{\rho-q}Z_1'\big)\Big]$$

where $Z_0, Z_1, Z_1' \overset{\text{iid}}{\sim} \mathcal{N}(0,1)$ and $h_f : (a,b) \in \mathbb{R}^2 \mapsto \int (f(y_1)-f(y_2))^2 P_{\text{out}}(y_1|a)P_{\text{out}}(y_2|b)dy_1dy_2$. By a central limit argument, we have:

Lemma 14.
$$\Big(\frac{\mathbf{x}\cdot\mathbf{\Phi}_{\text{new}}}{\sqrt{n}}, \frac{\mathbf{X}^*\cdot\mathbf{\Phi}_{\text{new}}}{\sqrt{n}}\Big) \xrightarrow[n\to\infty]{(d)} (Z_1, Z_2)\,,$$

where (Z_1, Z_2) is sampled, conditionally on Q, from $\mathcal{N}\Big(0, \left(\begin{smallmatrix}\rho & Q\\ Q & \rho\end{smallmatrix}\right)\Big)$.

Proof. Notice that $\mathbf{x}$ and $\mathbf{X}$ are independent of $\mathbf{\Phi}_{\text{new}}$. If $(\Phi_{\text{new},1},\dots,\Phi_{\text{new},n}) \overset{\text{iid}}{\sim} \mathcal{N}(0,1)$, then Lemma 14 is obvious because in that case

$$\Big(\frac{\mathbf{x}\cdot\mathbf{\Phi}_{\text{new}}}{\sqrt{n}}, \frac{\mathbf{X}^*\cdot\mathbf{\Phi}_{\text{new}}}{\sqrt{n}}\Big) \sim \mathcal{N}\bigg(0, \frac{1}{n}\begin{pmatrix}\|\mathbf{x}\|^2 & \mathbf{x}\cdot\mathbf{X}^*\\ \mathbf{x}\cdot\mathbf{X}^* & \|\mathbf{X}^*\|^2\end{pmatrix}\bigg) \quad \text{and} \quad \frac{1}{n}\begin{pmatrix}\|\mathbf{x}\|^2 & \mathbf{x}\cdot\mathbf{X}^*\\ \mathbf{x}\cdot\mathbf{X}^* & \|\mathbf{X}^*\|^2\end{pmatrix} \xrightarrow[n\to\infty]{(d)} \begin{pmatrix}\rho & Q\\ Q & \rho\end{pmatrix}.$$

Let us now suppose that the entries of $\mathbf{\Phi}_{\text{new}}$ are not i.i.d. standard Gaussian (but still verify hypothesis (h3)). Let $g_1,\dots,g_n \overset{\text{iid}}{\sim} \mathcal{N}(0,1)$. Let $L : \mathbb{R}^2 \to \mathbb{R}$ be a bounded $\mathcal{C}^3$ function, with bounded partial derivatives. We have to show that

$$\mathbb{E}\Big[L\Big(\frac{\mathbf{x}\cdot\mathbf{\Phi}_{\text{new}}}{\sqrt{n}}, \frac{\mathbf{X}^*\cdot\mathbf{\Phi}_{\text{new}}}{\sqrt{n}}\Big)\Big] \xrightarrow[n\to\infty]{} \mathbb{E}\big[L(Z_1,Z_2)\big]\,. \tag{139}$$

We have seen above that $\mathbb{E}\big[L\big(\frac{\mathbf{x}\cdot\mathbf{g}}{\sqrt{n}}, \frac{\mathbf{X}^*\cdot\mathbf{g}}{\sqrt{n}}\big)\big] \xrightarrow[n\to\infty]{} \mathbb{E}[L(Z_1,Z_2)]$. We now apply Theorem 5 (Theorem 2 from [54]) conditionally on $\mathbf{x}, \mathbf{X}^*$ to obtain

$$\mathbb{E}\Big[L\Big(\frac{\mathbf{x}\cdot\mathbf{\Phi}_{\text{new}}}{\sqrt{n}}, \frac{\mathbf{X}^*\cdot\mathbf{\Phi}_{\text{new}}}{\sqrt{n}}\Big)\Big] = \mathbb{E}\Big[L\Big(\frac{\mathbf{x}\cdot\mathbf{g}}{\sqrt{n}}, \frac{\mathbf{X}^*\cdot\mathbf{g}}{\sqrt{n}}\Big)\Big] + \mathcal{O}_n(n^{-1/2})\,,$$

which proves (139) and therefore Lemma 14. □

Proposition 11. *We have*

$$\mathcal{E}_{f,n}(\alpha) \xrightarrow[n\to\infty]{} \frac{1}{2}\mathbb{E}\big[h_f(Z_1,Z_2)\big]\,,$$

where (Z_1, Z_2) is defined in Lemma 14 above.

Proof. We have

$$\mathcal{E}_{f,n} = \mathbb{E}\Big[\Big(f(Y_{\text{new}}) - \mathbb{E}\big[f(Y_{\text{new}})\big|\boldsymbol{\Phi}_{\text{new}}, \boldsymbol{\Phi}, \mathbf{Y}\big]\Big)^2\Big]$$
$$= \frac{1}{2}\mathbb{E}\left[\int (f(y_{\text{new}}) - f(y))^2 P_{\text{out}}\big(y_{\text{new}}\big|\boldsymbol{\Phi}_{\text{new}} \cdot \mathbf{X}^*/\sqrt{n}\big) P_{\text{out}}\big(y\big|\boldsymbol{\Phi}_{\text{new}} \cdot \mathbf{x}/\sqrt{n}\big) dy_{\text{new}} dy\right]$$
$$= \frac{1}{2}\mathbb{E}\left[h_f\Big(\frac{\mathbf{x}\cdot\boldsymbol{\Phi}_{\text{new}}}{\sqrt{n}}, \frac{\mathbf{X}^*\cdot\boldsymbol{\Phi}_{\text{new}}}{\sqrt{n}}\Big)\right].$$

By Lemma 14 above, we have $\big(\frac{\mathbf{x}\cdot\boldsymbol{\Phi}_{\text{new}}}{\sqrt{n}}, \frac{\mathbf{X}^*\cdot\boldsymbol{\Phi}_{\text{new}}}{\sqrt{n}}\big) \xrightarrow[n\to\infty]{(d)} (Z_1, Z_2)$. Using (h4) (and the fact that either (h5.a) or (h5.b) hold), we can find a Borel set $S \subset \mathbb{R}$ of full Lebesgue's measure such that $x \mapsto P_{\text{out}}(y|x)$ is continuous on S, for all $y \in \mathbb{R}$. By dominated convergence (recall that f is assumed to be bounded), we obtain that h_f is continuous on $S \times S$. The set of discontinuity points of h_f has thus zero measure for the law of (Z_1, Z_2). Indeed if we condition on Q:

- if $|Q| < \rho$, then (Z_1, Z_2) has a density over $\mathbb{R}^2$.
- if $Q = \rho$, then $Z_1 = Z_2$ almost surely, but h_f is continuous on $\big\{(s,s)\,\big|\,s \in S\big\}$ that has full Lebesgue's measure on the diagonal $\big\{(x,x)\,\big|\,x \in \mathbb{R}\big\}$.
- if $Q = -\rho$, then $Z_1 = -Z_2$ almost surely and we use then similar arguments as for the previous point.

We have therefore:
$$h_f\Big(\frac{\mathbf{x}\cdot\boldsymbol{\Phi}_{\text{new}}}{\sqrt{n}}, \frac{\mathbf{X}^*\cdot\boldsymbol{\Phi}_{\text{new}}}{\sqrt{n}}\Big) \xrightarrow[n\to\infty]{(d)} h_f(Z_1, Z_2),$$
and Lemma 11 follows from the fact that h_f is bounded. □

Let us now define:
$$H_f : \left|\begin{array}{ccc} [-\rho,\rho] & \to & \mathbb{R} \\ q & \mapsto & \frac{1}{2}\mathbb{E}\big[h_f(G^{(q)})\big] \end{array}\right. \tag{140}$$
where $G^{(q)} \sim \mathcal{N}\big(0, \big(\begin{smallmatrix}\rho & q\\ q & \rho\end{smallmatrix}\big)\big)$. Notice that H_f is equal to the function $\mathcal{E}_f$ on $[0,\rho]$. By Proposition 11 above and Theorem 4, we have:
$$H_f(q^*(\alpha)) = \lim_{n\to\infty} \mathcal{E}_{f,n}(\alpha) = \mathbb{E}\big[H_f(Q)\big]. \tag{141}$$

Lemma 15. *For all $q \in [-\rho,\rho]$, $H_f(q) \geq H_f(|q|)$.*

Proof. Let $q \in [0,\rho]$ and $Z_0, Z_1, Z_1' \overset{\text{iid}}{\sim} \mathcal{N}(0,1)$.
$$H_f(-q) = \frac{1}{2}\mathbb{E}\Big[h_f\big(\sqrt{q}Z_0 + \sqrt{\rho-q}Z_1, -\sqrt{q}Z_0 + \sqrt{\rho-q}Z_1'\big)\Big].$$

Let us denote by $\mathbb{E}_{Z_1}$ and $\mathbb{E}_{Z_1'}$ the expectations with respect to Z_1 and Z_1'. By replacing h_f by its expression, we have
$$H_f(-q) = \frac{1}{2}\mathbb{E}\int (f(y) - f(y'))^2 P_{\text{out}}(y|\sqrt{q}Z_0 + \sqrt{\rho-q}Z_1) P_{\text{out}}(y'|-\sqrt{q}Z_0 + \sqrt{\rho-q}Z_1') dy dy'$$
$$= \frac{1}{2}\mathbb{E}\int (f(y) - f(y'))^2 \mathbb{E}_{Z_1} P_{\text{out}}(y|\sqrt{q}Z_0 + \sqrt{\rho-q}Z_1) \mathbb{E}_{Z_1'} P_{\text{out}}(y'|-\sqrt{q}Z_0 + \sqrt{\rho-q}Z_1') dy dy'$$
$$= \frac{1}{2}\mathbb{E}\int (f(y) - f(y'))^2 \widetilde{P}_{\text{out}}(y|Z_0) \widetilde{P}_{\text{out}}(y'|-Z_0) dy dy',$$

where $\widetilde{P}_{\text{out}}(y|z) = \mathbb{E}_{Z_1} P_{\text{out}}(y|\sqrt{q}z + \sqrt{\rho-q}Z_1)$. Let now Y and Y' be two random variables that are independent conditionally on Z_0 and distributed as
$$Y \sim \widetilde{P}_{\text{out}}(\cdot|Z_0) \qquad \text{and} \qquad Y' \sim \widetilde{P}_{\text{out}}(\cdot|-Z_0).$$

Then we have

$$
\begin{aligned}
H_f(-q) &= \frac{1}{2}\mathbb{E}\Big[\big(f(Y)-f(Y')\big)^2\Big] = \frac{1}{2}\mathbb{E}\Big[\big(f(Y)-\mathbb{E}[f(Y)|Z_0]+\mathbb{E}[f(Y)|Z_0]-f(Y')\big)^2\Big] \\
&= \frac{1}{2}\mathbb{E}\Big[\big(f(Y)-\mathbb{E}[f(Y)|Z_0]\big)^2\Big] + \frac{1}{2}\mathbb{E}\Big[\big(\mathbb{E}[f(Y)|Z_0]-f(Y')\big)^2\Big]\,,
\end{aligned}
$$

because Y and Y' are independent conditionally on Z_0. The conditional expectation $\mathbb{E}[f(Y)|Z_0]$ is Z_0-measurable, therefore $\mathbb{E}\big[(\mathbb{E}[f(Y)|Z_0]-f(Y'))^2\big] \geq \mathbb{E}\big[(\mathbb{E}[f(Y')|Z_0]-f(Y'))^2\big] = \mathbb{E}\big[(\mathbb{E}[f(Y)|Z_0]-f(Y))^2\big]$. We conclude

$$
H_f(-q) \geq \mathbb{E}\big[(\mathbb{E}[f(Y)|Z_0]-f(Y))^2\big] = H_f(q)\,.
$$

□

We have now all the tools needed to prove Theorem 2. Using Lemma 15 and (141) above, we get that $\mathbb{E}H_f(|Q|) \leq \mathbb{E}H_f(Q) = H_f(q^*(\alpha))$. Since H_f is equal to $\mathcal{E}_f$ on $[0,\rho]$ this gives

$$
\mathbb{E}\big[\mathcal{E}_f(|Q|)\big] \leq \mathcal{E}_f(q^*(\alpha))\,. \tag{142}
$$

If $q^*(\alpha) = 0$, then Theorem 2 follows simply from Proposition 9. We suppose now that $q^*(\alpha) > 0$ and consider $\epsilon \in (0, q^*(\alpha))$. We define $p(\epsilon) = \mathbb{P}\big(|Q| \leq q^*(\alpha)-\epsilon\big)$. We are going to show that $p(\epsilon) = 0$. We assumed that $P_{\rm out}$ is informative, so by Proposition 23 and Proposition 24 in Appendix B.2, there exists a continuous bounded function $f : \mathbb{R} \mapsto \mathbb{R}$ such that $\mathcal{E}_f$ is strictly decreasing on $[0,\rho]$. In the following, f is assumed to be such a function. We have

$$
\begin{aligned}
\mathbb{E}\big[\mathcal{E}_f(|Q|)\big] &= \mathbb{E}\Big[\mathbf{1}\big(|Q| \leq q^*(\alpha)-\epsilon\big)\mathcal{E}_f(|Q|) + \mathbf{1}\big(|Q| > q^*(\alpha)-\epsilon\big)\mathcal{E}_f(|Q|)\Big] \\
&\geq p(\epsilon)\mathcal{E}_f(q^*(\alpha)-\epsilon) + (1-p(\epsilon))\mathcal{E}_f(q^*(\alpha))\,.
\end{aligned}
$$

because $\mathcal{E}_f$ is non-increasing and because $|Q| \leq q^*(\alpha)$ almost-surely (Lemma 13). Combining this with (142) leads to

$$
p(\epsilon)\mathcal{E}_f(q^*(\alpha)) \geq p(\epsilon)\mathcal{E}_f(q^*(\alpha)-\epsilon)\,.
$$

Since $\mathcal{E}_f$ is strictly decreasing: $\mathcal{E}_f(q^*(\alpha)) < \mathcal{E}_f(q^*(\alpha)-\epsilon)$, which implies $p(\epsilon) = 0$. This is true for all $\epsilon > 0$, consequently $|Q| \geq q^*(\alpha)$ almost-surely. We get (using Lemma 13) that

$$
|Q| = q^*(\alpha)\,, \quad \text{almost-surely.}
$$

We conclude that the only possible limit in law of the tight sequence $\big(|Q_n|\big)_{n\geq 1}$ is $q^*(\alpha)$. Therefore $|Q_n| \to q^*(\alpha)$ in law and in probability because $q^*(\alpha)$ is a constant.

5.4 Denoising error: Proof of Corollary 4

Start by noticing that the denoising error, i.e. the right hand side of (55), is obtained through the I-MMSE theorem, see Proposition 13, applied to $\widetilde{i}_n := I(\mathbf{X}^*, \mathbf{A}; \mathbf{Y}\,|\boldsymbol{\Phi})/n$:

$$
\frac{\partial \widetilde{i}_n}{\partial \Delta^{-1}} = \frac{1}{2n}\mathrm{MMSE}\Big(\varphi\Big(\frac{1}{\sqrt{n}}\boldsymbol{\Phi}\mathbf{X}^*, \mathbf{A}\Big)\Big|\boldsymbol{\Phi}, \mathbf{Y}\Big)\,. \tag{143}
$$

This mutual information is simply computed using our main theorem. Indeed,

$$
\widetilde{i}_\infty := \lim_{n\to\infty} \widetilde{i}_n = \lim_{n\to\infty} \frac{1}{n}H(\mathbf{Y}|\boldsymbol{\Phi}) - \lim_{n\to\infty} \frac{1}{n}H(\mathbf{Y}|\boldsymbol{\Phi}, \mathbf{X}^*, \mathbf{A}) = -f_\infty - \lim_{n\to\infty} \frac{1}{n}H(\mathbf{Y}|\boldsymbol{\Phi}, \mathbf{X}^*, \mathbf{A})\,.
$$

One can simply check that $\lim_{n\to\infty} H(\mathbf{Y}|\mathbf{\Phi},\mathbf{X}^*,\mathbf{A})/n = \alpha\ln(2\pi\Delta e)/2$ by similar computations as in the proof of Corollary 1. Therefore, defining

$$\widetilde{i}_{\rm RS}(q,\Delta) := -\frac{\alpha}{2}\ln(2\pi\Delta e) - \alpha\Psi_{P_{\rm out}}(q) - \inf_{r\geq 0}\Big\{\psi_{P_0}(r) - \frac{qr}{2}\Big\},$$

we have $\widetilde{i}_\infty = \sup_{q\in[0,\rho]}\widetilde{i}_{\rm RS}(q,\Delta)$ from Theorem 1.

One can verify easily that $\widetilde{i}_n$ is a concave differentiable function of Δ^{-1} (this is again related to the I-MMSE theorem). Thus its limit $\widetilde{i}_\infty$ is also a concave function of Δ^{-1}. Therefore, a standard analysis lemma gives that the derivative of $\widetilde{i}_n$ w.r.t. Δ^{-1} converges to the derivative of $\widetilde{i}_\infty$ at every point at which $\widetilde{i}_\infty$ is differentiable (i.e. almost every points, by concavity): $\lim_{n\to\infty}\partial_{\Delta^{-1}}\widetilde{i}_n = \partial_{\Delta^{-1}}\widetilde{i}_\infty = \partial_{\Delta^{-1}}\sup_{q\in[0,\rho]}\widetilde{i}_{\rm RS}(q,\Delta)$. The first limit is given by the limit of the right hand side of (143). It thus remains to compute $\partial_{\Delta^{-1}}\sup_{q\in[0,\rho]}\widetilde{i}_{\rm RS}(q,\Delta)$.

Assume for a moment that the $\partial_{\Delta^{-1}}$ and $\sup_{q\in[0,\rho]}$ operations commute. Then we need to compute $\partial_{\Delta^{-1}}\Psi_{P_{\rm out}}(q)$; this follows from the I-MMSE theorem. Indeed, if we denote $S = \varphi(\sqrt{q}\,V + \sqrt{\rho-q}\,W^*,\mathbf{A})$, notice that $\Psi_{P_{\rm out}}(q) = -I(S;S+\sqrt{\Delta}Z\,|\,V) - \ln(2\pi e\Delta)/2$, because $\Psi_{P_{\rm out}}(q) = -H(S+\sqrt{\Delta}Z\,|\,V)$ and $I(S+\sqrt{\Delta}Z;S\,|\,V) = H(S+\sqrt{\Delta}Z\,|\,V) - H(S+\sqrt{\Delta}Z\,|\,V,S) = -\Psi_{P_{\rm out}}(q) - \ln(2\pi e\Delta)/2$. Therefore

$$\begin{aligned}\frac{\partial\Psi_{P_{\rm out}}(q)}{\partial\Delta^{-1}} &= \frac{\Delta}{2} - \frac{\partial}{\partial\Delta^{-1}}I(S+\sqrt{\Delta}Z;S\,|\,V) = \frac{\Delta}{2} - \frac{1}{2}\mathrm{MMSE}(S\,|\,V,S+\sqrt{\Delta}Z)\\ &= \frac{\Delta}{2} - \frac{1}{2}\Big(\mathbb{E}\big[\varphi(\sqrt{\rho}\,V,\mathbf{A})^2\big] - \mathbb{E}\big[\langle\varphi(\sqrt{q}\,V+\sqrt{\rho-q}\,w,\mathbf{a})\rangle_{\rm sc}^2\big]\Big)\,.\end{aligned}$$

Consequently,

$$\frac{\partial\widetilde{i}_{\rm RS}(q,\Delta)}{\partial\Delta^{-1}} = \frac{\alpha}{2}\Big(\mathbb{E}\big[\varphi(\sqrt{\rho}\,V,\mathbf{A})^2\big] - \mathbb{E}\big[\langle\varphi(\sqrt{q}\,V+\sqrt{\rho-q}\,w,\mathbf{a})\rangle_{\rm sc}^2\big]\Big)\,.$$

Now, Theorem 1 from [52] gives that at every Δ^{-1} at which i_∞ is differentiable

$$\frac{\partial\widetilde{i}_\infty}{\partial\Delta^{-1}} = \frac{\partial}{\partial\Delta^{-1}}\sup_{q\in[0,\rho]}\widetilde{i}_{\rm RS}(q,\Delta) = \frac{\alpha}{2}\Big(\mathbb{E}\big[\varphi(\sqrt{\rho}\,V,\mathbf{A})^2\big] - \mathbb{E}\big[\langle\varphi(\sqrt{q^*}\,V+\sqrt{\rho-q^*}\,w,\mathbf{a})\rangle_{\rm sc}^2\big]\Big)$$

where $q^*\in[0,\rho]$ is a point where the supremum above is achieved, and thus corresponds to an optimal couple in (24). As explained above, $\lim_{n\to\infty}\partial_{\Delta^{-1}}\widetilde{i}_n = \partial_{\Delta^{-1}}\widetilde{i}_\infty$ at every Δ^{-1} at which $\widetilde{i}_\infty$ is differentiable, which concludes the proof.

Appendix A: Some technicalities

A.1 The Nishimori identity

Proposition 12 (Nishimori identity)**.** *Let $(\boldsymbol{X},\boldsymbol{Y})\in\mathbb{R}^{n_1}\times\mathbb{R}^{n_2}$ be a couple of random variables. Let $k\geq 1$ and let $\boldsymbol{X}^{(1)},\dots,\boldsymbol{X}^{(k)}$ be k i.i.d. samples (given $\boldsymbol{Y}$) from the conditional distribution $P(\boldsymbol{X}=\cdot\,|\boldsymbol{Y})$, independently of every other random variables. Let us denote $\langle-\rangle$ the expectation operator w.r.t. $P(\boldsymbol{X}=\cdot\,|\boldsymbol{Y})$ and $\mathbb{E}$ the expectation w.r.t. $(\boldsymbol{X},\boldsymbol{Y})$. Then, for all continuous bounded function g we have*

$$\mathbb{E}\langle g(\boldsymbol{Y},\boldsymbol{X}^{(1)},\dots,\boldsymbol{X}^{(k)})\rangle = \mathbb{E}\langle g(\boldsymbol{Y},\boldsymbol{X}^{(1)},\dots,\boldsymbol{X}^{(k-1)},\boldsymbol{X})\rangle\,. \tag{144}$$

Proof. This is a simple consequence of Bayes formula. It is equivalent to sample the couple $(\mathbf{X},\mathbf{Y})$ according to its joint distribution or to sample first $\mathbf{Y}$ according to its marginal distribution and then to sample $\mathbf{X}$ conditionally to $\mathbf{Y}$ from its conditional distribution $P(\mathbf{X}=\cdot\,|\mathbf{Y})$. Thus the $(k+1)$-tuple $(\mathbf{Y},\mathbf{X}^{(1)},\dots,\mathbf{X}^{(k)})$ is equal

in law to $(\mathbf{Y}, \mathbf{X}^{(1)}, \dots, \mathbf{X}^{(k-1)}, \mathbf{X})$. □

A.2 Unicity of the optimizer q^* of the replica formula: Proof of Proposition 1

The function

$$h : \alpha \mapsto \inf_{q \in [0,\rho]} \Big\{ \alpha \mathcal{I}_{P_{\text{out}}}(q) + \sup_{r \geq 0} \big\{ I_{P_0}(r) - \frac{r}{2}(\rho - q) \big\} \Big\} \tag{145}$$

is concave (as an infimum of linear functions). An "envelope" theorem (Corollary 4 from [52]) gives that h is differentiable at α if and only if

$$\Big\{ \mathcal{I}_{P_{\text{out}}}(q) \,\Big|\, q \text{ minimizer of } (145) \Big\}$$

is a singleton. We assumed that P_{out} is informative, so Proposition 21 gives that $\mathcal{I}_{P_{\text{out}}}$ is strictly decreasing. We obtain thus that the set of points at which h is differentiable is exactly D^*. Since h is concave, D^* is equal to $\mathbb{R}_+^*$ minus a countable set. Corollary 4 from [52] gives also that $h'(\alpha) = \mathcal{I}_{P_{\text{out}}}(q^*(\alpha))$, for all $\alpha \in D^*$. The function h is concave, so its derivative h' is non-increasing. Since $\mathcal{I}_{P_{\text{out}}}$ is strictly decreasing, we obtain that $\alpha \in D^* \mapsto q^*(\alpha)$ is non-decreasing.

Let now $\alpha_0 \in D^*$. By concavity of h, $h'(\alpha) \to h'(\alpha_0)$ when $\alpha \in D^* \to \alpha_0$. Therefore:

$$\mathcal{I}_{P_{\text{out}}}(q^*(\alpha)) \xrightarrow[\alpha \in D \to \alpha_0]{} \mathcal{I}_{P_{\text{out}}}(q^*(\alpha_0))$$

which implies $q^*(\alpha) \to q^*(\alpha_0)$ by strict monotonicity of $\mathcal{I}_{P_{\text{out}}}$.

A.3 Continuity properties of the mutual information

We establish in this section two continuity properties of the mutual information, namely Proposition 14 and Corollary 6. Recall definition (14) of the MMSE function. The following proposition comes from [55] and will be repeatedly used in the sequel.

Proposition 13 (I-MMSE theorem, [53, 55])**.** *Let P_X be a probability distribution over $\mathbb{R}^n$ that admits a finite second moment. Let $\boldsymbol{X} \sim P_X$ and $\boldsymbol{Z} \sim \mathcal{N}(0, \mathbf{I}_n)$ be independent random variables. Then the function*

$$I_{P_X} : \left| \begin{array}{ccc} \mathbb{R}_+ & \to & \mathbb{R} \\ \lambda & \mapsto & I(\boldsymbol{X}; \sqrt{\lambda}\boldsymbol{X} + \boldsymbol{Z}) \end{array} \right.$$

is concave, continuously differentiable over $\mathbb{R}_+$, with derivative given by

$$I'_{P_X}(\lambda) = \frac{1}{2}\mathrm{MMSE}(\boldsymbol{X} | \sqrt{\lambda}\boldsymbol{X} + \boldsymbol{Z}) = \frac{1}{2}\mathbb{E}\Big[\big\| \boldsymbol{X} - \mathbb{E}[\boldsymbol{X} | \sqrt{\lambda}\boldsymbol{X} + \boldsymbol{Z}] \big\|^2 \Big].$$

Remark: We will often apply Proposition 13 in a "conditional fashion". Let $\mathbf{U}$ be some random variable independent from $\mathbf{Z}$, then

$$\frac{\partial}{\partial \lambda} I(\mathbf{X}; \sqrt{\lambda}\mathbf{X} + \mathbf{Z} | \mathbf{U}) = \frac{1}{2}\mathrm{MMSE}(\mathbf{X} | \sqrt{\lambda}\mathbf{X} + \mathbf{Z}, \mathbf{U}) = \frac{1}{2}\mathbb{E}\Big[\big\| \mathbf{X} - \mathbb{E}[\mathbf{X} | \sqrt{\lambda}\mathbf{X} + \mathbf{Z}, \mathbf{U}] \big\|^2 \Big].$$

Proposition 14. *Let P_1 and P_2 be two probability distributions on $\mathbb{R}^n$, that admits a finite second moment. We denote by $W_2(P_1, P_2)$ the Wasserstein distance of order 2 between P_1 and P_2.*

$$\big| I(\boldsymbol{X}_1; \boldsymbol{X}_1 + \boldsymbol{Z}) - I(\boldsymbol{X}_2; \boldsymbol{X}_2 + \boldsymbol{Z}) \big| \leq \big(\sqrt{\mathbb{E}\|\boldsymbol{X}_1\|^2} + \sqrt{\mathbb{E}\|\boldsymbol{X}_2\|^2} \big) W_2(P_1, P_2).$$

A similar result was proved in [56] but with a weaker bound for the W_2 distance.

Proof. Let $\epsilon > 0$. Let us fix a coupling of $\mathbf{X}_1 \sim P_1$ and $\mathbf{X}_2 \sim P_2$ such that

$$\big(\mathbb{E}\|\mathbf{X}_1 - \mathbf{X}_2\|^2\big)^{1/2} \leq W_2(P_1, P_2) + \epsilon\,.$$

Let us consider for $t_1, t_2 \in [0,1]$ the observation model

$$\begin{cases} \mathbf{Y}_1^{(t_1)} &= \sqrt{t_1}\mathbf{X}_1 + \mathbf{Z}_1\,, \\ \mathbf{Y}_2^{(t_2)} &= \sqrt{1-t_2}\mathbf{X}_2 + \mathbf{Z}_2\,, \end{cases}$$

where $\mathbf{Z}_1, \mathbf{Z}_2 \overset{\text{iid}}{\sim} \mathcal{N}(0, \mathbf{I}_n)$ are independent from $(\mathbf{X}_1, \mathbf{X}_2)$. Define $J(t_1,t_2) = I(\mathbf{X}_1, \mathbf{X}_2; \mathbf{Y}_1^{(t_1)}, \mathbf{Y}_2^{(t_2)})$ and $I(t) = J(t,t)$. Let us now differentiate J with respect to t_1. Using the chain rule for the mutual information,

$$\begin{aligned} J(t_1,t_2) &= I(\mathbf{X}_1,\mathbf{X}_2;\mathbf{Y}_2^{(t_2)}) + I(\mathbf{X}_1,\mathbf{X}_2;\mathbf{Y}_1^{(t_1)}|\mathbf{Y}_2^{(t_2)}) = I(\mathbf{X}_1,\mathbf{X}_2;\mathbf{Y}_2^{(t_2)}) + I(\mathbf{X}_1;\mathbf{Y}_1^{(t_1)}|\mathbf{Y}_2^{(t_2)}) + I(\mathbf{X}_2;\mathbf{Y}_1^{(t_1)}|\mathbf{X}_1,\mathbf{Y}_2^{(t_2)}) \\ &= I(\mathbf{X}_1,\mathbf{X}_2;\mathbf{Y}_2^{(t_2)}) + I(\mathbf{X}_1;\mathbf{Y}_1^{(t_1)}|\mathbf{Y}_2^{(t_2)}) \end{aligned}$$

because, conditionally on $\mathbf{X}_1$, $\mathbf{X}_2$ and $\mathbf{Y}_1^{(t_1)}$ are independent. The quantity $I(\mathbf{X}_1,\mathbf{X}_2;\mathbf{Y}_2^{(t_2)})$ does not depend on t_1, therefore by the "I-MMSE relation" from Proposition 13:

$$\frac{\partial J}{\partial t_1}(t_1,t_2) = \frac{1}{2}\mathrm{MMSE}(\mathbf{X}_1|\mathbf{Y}_1^{(t_1)},\mathbf{Y}_2^{(t_2)})$$

and similarly

$$\frac{\partial J}{\partial t_2}(t_1,t_2) = -\frac{1}{2}\mathrm{MMSE}(\mathbf{X}_2|\mathbf{Y}_1^{(t_1)},\mathbf{Y}_2^{(t_2)})\,.$$

Let us write $\mathbf{E}_i = \mathbb{E}[\mathbf{X}_i|\mathbf{Y}_1^{(t)},\mathbf{Y}_2^{(t)}]$ for $i=1,2$, then

$$I'(t) = \frac{1}{2}\mathrm{MMSE}(\mathbf{X}_1|\mathbf{Y}_1^{(t)},\mathbf{Y}_2^{(t)}) - \frac{1}{2}\mathrm{MMSE}(\mathbf{X}_2|\mathbf{Y}_1^{(t)},\mathbf{Y}_2^{(t)}) = \frac{1}{2}\mathbb{E}\left[\big\|\mathbf{X}_1-\mathbf{E}_1\big\|^2 - \big\|\mathbf{X}_2-\mathbf{E}_2\big\|^2\right]$$

so that

$$\begin{aligned} |I'(t)| &= \frac{1}{2}\mathbb{E}\left[\big(\|\mathbf{X}_1-\mathbf{E}_1\| + \|\mathbf{X}_2-\mathbf{E}_2\|\big)\big(\|\mathbf{X}_1-\mathbf{E}_1\| - \|\mathbf{X}_2-\mathbf{E}_2\|\big)\right] \\ &\leq \frac{1}{2}\mathbb{E}\left[\big\|\mathbf{X}_1\big\|^2 + \big\|\mathbf{X}_2\big\|^2\right]^{1/2}\mathbb{E}\left[\big(\|\mathbf{X}_1-\mathbf{E}_1\| - \|\mathbf{X}_2-\mathbf{E}_2\|\big)^2\right]^{1/2} \\ &\leq \frac{1}{2}\mathbb{E}\left[\big\|\mathbf{X}_1\big\|^2 + \big\|\mathbf{X}_2\big\|^2\right]^{1/2}\mathbb{E}\left[\big\|\mathbf{X}_1-\mathbf{X}_2+\mathbf{E}_2-\mathbf{E}_1\big\|^2\right]^{1/2} \\ &\leq \frac{1}{2}\big(\sqrt{\mathbb{E}\|\mathbf{X}_1\|^2} + \sqrt{\mathbb{E}\|\mathbf{X}_2\|^2}\big)\mathbb{E}\left[2\|\mathbf{X}_1-\mathbf{X}_2\|^2 + 2\|\mathbf{E}_2-\mathbf{E}_1\|^2\right]^{1/2} \\ &\leq \big(\sqrt{\mathbb{E}\|\mathbf{X}_1\|^2} + \sqrt{\mathbb{E}\|\mathbf{X}_2\|^2}\big)\big(W_2(P_1,P_2)+\epsilon\big)\,. \end{aligned}$$

We obtain the result by letting $\epsilon \to 0$. □

Proposition 15. *Let P_U be a probability distribution over $\mathbb{N}^m$ that admits a finite second moment. Let $\boldsymbol{U} \sim P_U$ and $\boldsymbol{Z} \sim \mathcal{N}(0, \mathbf{I}_m)$ be two independent random variables. Then $H(\boldsymbol{U}) = -\sum_{\mathbf{n}\in\mathbb{N}^m} P_U(\mathbf{n})\ln P_U(\mathbf{n})$ is finite and for all $\Delta \in (0,1]$,*

$$\big|I(\boldsymbol{U};\boldsymbol{U}+\sqrt{\Delta}\boldsymbol{Z}) - H(\boldsymbol{U})\big| \leq 48me^{-1/(16\Delta)}\,.$$

Proof. Let us define for $\Delta > 0$, $h(\Delta) = I(\mathbf{U};\mathbf{U}+\sqrt{\Delta}\mathbf{Z}) = I_{P_U}(\Delta^{-1})$. By Proposition 13 we have for all $\Delta > 0$,

$$h'(\Delta) = -\frac{1}{2\Delta^2}\mathrm{MMSE}(\mathbf{U}\,|\,\mathbf{U}+\sqrt{\Delta}\mathbf{Z})\,. \tag{146}$$

We are now going to upper bound $\mathrm{MMSE}(\mathbf{U} \,|\, \mathbf{U}+\sqrt{\Delta}\mathbf{Z})$ by considering the following estimator:

$$\widehat{\theta}_i = \arg\min_{u\in\mathbb{N}} |u - U_i + \sqrt{\Delta} Z_i|,$$

for all $i \in \{1,\dots,m\}$. Note that $\widehat{\theta}_i$ is well-defined almost-surely since there is a.s. a unique minimizer above. We have

$$\mathbb{P}(\widehat{\theta}_i \neq U_i) \leq \mathbb{P}\big(\sqrt{\Delta}|Z_i| \geq 1/2\big) = 2\mathbb{P}\Big(\mathcal{N}(0,1) \geq \frac{1}{2\sqrt{\Delta}}\Big) \leq 2\frac{1}{\sqrt{2\pi}} 2\sqrt{\Delta} e^{-1/(8\Delta)} \leq 2\sqrt{\Delta} e^{-1/(8\Delta)}\,,$$

by usual bounds on the Gaussian cumulative distribution function. We have then

$$\begin{aligned}
\mathrm{MMSE}(\mathbf{U} \,|\, \mathbf{U}+\sqrt{\Delta}\mathbf{Z}) &\leq \mathbb{E}\|\mathbf{U}-\widehat{\theta}\|^2 = \sum_{i=1}^m \mathbb{E}(U_i - \widehat{\theta}_i)^2 = \sum_{i=1}^m \mathbb{E}\Big[\mathbf{1}(\widehat{\theta}_i \neq U_i)(U_i-\widehat{\theta}_i)^2\Big] \\
&\leq \sum_{i=1}^m 2\mathbb{E}\Big[\mathbf{1}(\widehat{\theta}_i \neq U_i)(U_i - (U_i+\sqrt{\Delta}Z_i))^2\Big] + 2\mathbb{E}\Big[\mathbf{1}(\widehat{\theta}_i \neq U_i)(U_i+\sqrt{\Delta}Z_i-\widehat{\theta}_i)^2\Big] \\
&\leq \sum_{i=1}^m 2\mathbb{E}\Big[\mathbf{1}(\widehat{\theta}_i \neq U_i)\Delta Z_i^2\Big] + \frac{1}{2}\mathbb{E}\Big[\mathbf{1}(\widehat{\theta}_i \neq U_i)\Big] \\
&\leq \sum_{i=1}^m 2\Delta \mathbb{P}(\widehat{\theta}_i \neq U_i)^{1/2}\mathbb{E}[Z_i^4]^{1/2} + \frac{1}{2}\mathbb{P}(\widehat{\theta}_i \neq U_i) \\
&\leq m e^{-1/(16\Delta)}\Big(2\sqrt{6}\Delta^{5/4} + \sqrt{\Delta}\Big) \leq 6 m e^{-1/(16\Delta)}
\end{aligned}$$

for $\Delta \leq 1$. Plugging this inequality in (146), we obtain for all $\Delta \in (0,1]$,

$$|h'(\Delta)| \leq \frac{3m}{\Delta^2} e^{-1/(16\Delta)}\,. \tag{147}$$

Since $h(1)$ is finite and $\int_0^1 \frac{e^{-1/(16\Delta)}}{\Delta^2} d\Delta < +\infty$ we obtain that

$$\sup_{\Delta\in(0,1]} |h(\Delta)| < +\infty\,. \tag{148}$$

By definition of h:

$$h(\Delta) = I(\mathbf{U};\mathbf{U}+\sqrt{\Delta}\mathbf{Z}) = -\frac{m}{2} - \mathbb{E}\ln \sum_{\mathbf{u}\in\mathbb{N}^m} P_U(\mathbf{u}) \exp\Big(-\frac{1}{2\Delta}\|\mathbf{U}+\sqrt{\Delta}\mathbf{Z}-\mathbf{u}\|^2\Big)\,. \tag{149}$$

By the previous equality and (148), the family of (non-negative) random variables

$$\left(-\ln \sum_{\mathbf{u}\in\mathbb{N}^m} P_U(\mathbf{u}) \exp\Big(-\frac{1}{2\Delta}\|\mathbf{U}+\sqrt{\Delta}\mathbf{Z}-\mathbf{u}\|^2\Big)\right)_{\Delta\in(0,1]}$$

is bounded in L^1. Notice that (by dominated convergence)

$$-\ln \sum_{\mathbf{u}\in\mathbb{N}^m} P_U(\mathbf{u}) \exp\Big(-\frac{1}{2\Delta}\|\mathbf{U}+\sqrt{\Delta}\mathbf{Z}-\mathbf{u}\|^2\Big) \xrightarrow[\Delta\to 0]{} -\ln\Big(P_U(\mathbf{U})e^{-\frac{1}{2}\|\mathbf{Z}\|^2}\Big) = \frac{1}{2}\|\mathbf{Z}\|^2 - \ln P_U(\mathbf{U})$$

almost-surely. This gives (by Fatou's Lemma) that this almost-sure limit is integrable and thus that $H(\mathbf{U}) =$

$-\mathbb{E}\ln P_U(\mathbf{U})$ is finite. Let us now show that $h(\Delta) \xrightarrow[\Delta\to 0]{} H(\mathbf{U})$. We have almost-surely

$$\ln\Big(P_U(\mathbf{U})e^{-\frac{1}{2}\|\mathbf{Z}\|^2}\Big) \leq \ln\sum_{\mathbf{u}\in\mathbb{N}^m} P_U(\mathbf{u})\exp\Big(-\frac{1}{2\Delta}\|\mathbf{U}+\sqrt{\Delta}\mathbf{Z}-\mathbf{u}\|^2\Big) \leq 0\,.$$

Since we now know that the left-hand side is integrable (because $H(\mathbf{U})$ is finite), we can apply the dominated convergence theorem to obtain that

$$\mathbb{E}\ln\sum_{\mathbf{u}\in\mathbb{N}^m} P_U(\mathbf{u})\exp\Big(-\frac{1}{2\Delta}\|\mathbf{U}+\sqrt{\Delta}\mathbf{Z}-\mathbf{u}\|^2\Big) \xrightarrow[\Delta\to 0]{} \mathbb{E}\ln\Big(P_U(\mathbf{U})e^{-\frac{1}{2}\|\mathbf{Z}\|^2}\Big) = H(\mathbf{U}) - \frac{m}{2}\,,$$

which combined with (149) gives $h(\Delta) \xrightarrow[\Delta\to 0]{} H(\mathbf{U})$. Now, using the bound on the derivative of h (147) we conclude that for all $\Delta \in (0,1]$,

$$|h(\Delta) - H(\mathbf{U})| \leq 3m\int_0^\Delta \frac{e^{-1/(16t)}}{t^2}dt = 3m\Big[16e^{-1/(16t)}\Big]_0^\Delta = 48me^{-1/(16\Delta)}\,.$$

□

Corollary 6. *Let $\mathbf{U}$ be a random variable over $\mathbb{N}^m$ with finite second moment, let $\mathbf{X}$ be a random variable over $\mathbb{R}^n$ and let $\mathbf{Z}\sim\mathcal{N}(0,\mathbf{I}_m)$. We assume $(\mathbf{U},\mathbf{X})$ to be independent from $\mathbf{Z}$. Then, for all $\Delta\in(0,1]$,*

$$\big|I(\mathbf{X};\mathbf{U}+\sqrt{\Delta}\mathbf{Z}) - I(\mathbf{X};\mathbf{U})\big| \leq 100me^{-1/(16\Delta)}\,.$$

Proof. We have by the chain rule of the mutual information:

$$I(\mathbf{U};\mathbf{U}+\sqrt{\Delta}\mathbf{Z}) = I(\mathbf{U},\mathbf{X};\mathbf{U}+\sqrt{\Delta}\mathbf{Z}) = I(\mathbf{X};\mathbf{U}+\sqrt{\Delta}\mathbf{Z}) + I(\mathbf{U};\mathbf{U}+\sqrt{\Delta}\mathbf{Z}|\mathbf{X})\,.$$

By applying Proposition 15 twice, we get

$$|I(\mathbf{U};\mathbf{U}+\sqrt{\Delta}\mathbf{Z}) - H(\mathbf{U})|,\ |I(\mathbf{U};\mathbf{U}+\sqrt{\Delta}\mathbf{Z}|\mathbf{X}) - H(\mathbf{U}|\mathbf{X})| \leq 48me^{-1/(16\Delta)}\,.$$

Since $I(\mathbf{X};\mathbf{U}) = H(\mathbf{U}) - H(\mathbf{U}|\mathbf{X})$ we obtain the desired inequality. □

A.4 A simple consequence of hypotheses (h1)-(h2)-(h3)-(h4)

Proposition 16. *Assume that hypotheses (h1)-(h2)-(h3)-(h4) hold. Then there exists $\eta > 0$ such that*

$$\mathbb{E}\big[\varphi(\sqrt{\rho}Z,\mathbf{A})^{2+\eta}\big] < \infty\,,$$

where the expectation above is with respect to $(Z,\mathbf{A})\sim\mathcal{N}(0,1)\otimes P_A$.

Proof. By the Central Limit Theorem (using the fact that the third moments of $(X_i^*\Phi_{1,i})$ are bounded with n, because of hypotheses (h1) and (h3)) we have $\big([\mathbf{\Phi X}^*]_1/\sqrt{n},\mathbf{A}_1\big) \xrightarrow[n\to\infty]{(d)} (\sqrt{\rho}G,\mathbf{A}_1)$. This implies that

$$\varphi\left(\frac{[\mathbf{\Phi X}^*]_1}{\sqrt{n}},\mathbf{A}_1\right) \xrightarrow[n\to\infty]{(d)} \varphi(\sqrt{\rho}G,\mathbf{A}_1)\,, \tag{150}$$

because $\varphi(\cdot,\mathbf{A}_1)$ is almost-surely continuous almost-everywhere, by assumption (h4). The sequence of random variables $\big(\varphi([\mathbf{\Phi X}^*]_1/\sqrt{n},\mathbf{A}_1)\big)_n$ is by assumption (h2) bounded in $L^{2+\eta}$ for some $\eta > 0$. By (150) we conclude that $\mathbb{E}[\varphi(\sqrt{\rho}G,\mathbf{A}_1)^{2+\eta}] < \infty$. □

A.5 Derivative of the interpolating free entropy: Proof of Proposition 3

Recall $u'_y(x)$ is the x-derivative of $u_y(x) = \ln P_{\rm out}(y|x)$. Moreover denote $P'_{\rm out}(y|x)$ and $P''_{\rm out}(y|x)$ the first and second x-derivatives, respectively, of $P_{\rm out}(y|x)$. We will first prove that for all $t \in (0,1)$

$$\frac{df_{n,\epsilon}(t)}{dt} = -\frac{1}{2}\mathbb{E}\Big\langle\Big(\frac{1}{n}\sum_{\mu=1}^{m} u'_{Y_{t,\mu}}(S_{t,\mu})u'_{Y_{t,\mu}}(s_{t,\mu}) - r(t)\Big)\big(Q - q(t)\big)\Big\rangle_{n,t,\epsilon} + \frac{r(t)}{2}(q(t)-\rho) - \frac{A_n}{2}\,, \tag{151}$$

where recall $Q := \sum_{i=1}^n X_i^* x_i/n$ and

$$A_{n,\epsilon} := \mathbb{E}\Big[\frac{1}{\sqrt{n}}\sum_{\mu=1}^{m}\frac{P''_{\rm out}(Y_{t,\mu}|S_{t,\mu})}{P_{\rm out}(Y_{t,\mu}|S_{t,\mu})}\Big(\frac{1}{\sqrt{n}}\sum_{i=1}^{n}\big((X_i^*)^2-\rho\big)\Big)\frac{1}{n}\ln\mathcal{Z}_{t,\epsilon}\Big]\,. \tag{152}$$

Once this is done, we will prove that $A_{n,\epsilon}$ goes to 0 as $n\to\infty$ uniformly in $t\in[0,1]$, in order to obtain Proposition 3.

A.5.1 Proof of (151)

Recall definition (79) which becomes, when written as a function of the interpolating Hamiltonian (76),

$$f_{n,\epsilon}(t) = \frac{1}{n}\mathbb{E}_{\mathbf{\Phi},\mathbf{V}}\int d\mathbf{Y}_t d\mathbf{Y}'_t dP_0(\mathbf{X}^*)\mathcal{D}\mathbf{W}^* e^{-\mathcal{H}_{t,\epsilon}(\mathbf{X}^*,\mathbf{W}^*;\mathbf{Y}_t,\mathbf{Y}'_t,\mathbf{\Phi},\mathbf{V})}\ln\int dP_0(\mathbf{x})\mathcal{D}\mathbf{w}\, e^{-\mathcal{H}_{t,\epsilon}(\mathbf{x},\mathbf{w};\mathbf{Y}_t,\mathbf{Y}'_t,\mathbf{\Phi},\mathbf{V})}\,. \tag{153}$$

We will need the Hamiltonian t-derivative $\mathcal{H}'_{t,\epsilon}$ given by

$$\mathcal{H}'_{t,\epsilon}(\mathbf{X}^*,\mathbf{W}^*;\mathbf{Y}_t,\mathbf{Y}'_t,\mathbf{\Phi},\mathbf{V}) = -\sum_{\mu=1}^{m}\frac{dS_{t,\mu}}{dt}u'_{Y_{t,\mu}}(S_{t,\mu}) - \frac{r(t)}{2\sqrt{R_1(t)}}\sum_{i=1}^{n}X_i^*(Y'_{t,i}-\sqrt{R_1(t)}X_i^*)\,. \tag{154}$$

The derivative of the interpolating free entropy thus reads, for $0<t<1$,

$$\frac{df_{n,\epsilon}(t)}{dt} = \underbrace{-\frac{1}{n}\mathbb{E}\big[\mathcal{H}'_{t,\epsilon}(\mathbf{X}^*,\mathbf{W}^*;\mathbf{Y}_t,\mathbf{Y}'_t,\mathbf{\Phi},\mathbf{V})\ln\mathcal{Z}_{t,\epsilon}\big]}_{T_1} \underbrace{-\frac{1}{n}\mathbb{E}\big\langle\mathcal{H}'_{t,\epsilon}(\mathbf{x},\mathbf{w};\mathbf{Y}_t,\mathbf{Y}'_t,\mathbf{\Phi},\mathbf{V})\big\rangle_{n,t,\epsilon}}_{T_2} \tag{155}$$

where recall the definition of $\mathcal{Z}_{t,\epsilon} = \mathcal{Z}_{t,\epsilon}(\mathbf{Y}_t,\mathbf{Y}'_t,\mathbf{\Phi},\mathbf{V})$ given by (78).

Let us compute T_1. Let $1\le\mu\le m$. Let us start with the following term

$$\mathbb{E}\Big[\frac{dS_{t,\mu}}{dt}u'_{Y_{t,\mu}}(S_{t,\mu})\ln\mathcal{Z}_{t,\epsilon}\Big] = \frac{1}{2}\mathbb{E}\Big[\Big(-\frac{[\mathbf{\Phi}\mathbf{X}^*]_\mu}{\sqrt{n(1-t)}} + \frac{q(t)}{\sqrt{R_2(t)}}V_\mu + \frac{\rho-q(t)}{\sqrt{\rho t - R_2(t) + 2s_n}}W_\mu^*\Big)u'_{Y_{t,\mu}}(S_{t,\mu})\ln\mathcal{Z}_{t,\epsilon}\Big]\,. \tag{156}$$

Let us compute the first term of the right-hand side of the last identity. By Gaussian integration by parts w.r.t

$\Phi_{\mu i}$ we obtain

$$\begin{aligned}
&\frac{1}{\sqrt{n(1-t)}}\mathbb{E}\big[[\mathbf{\Phi X}^*]_\mu u'_{Y_{t,\mu}}(S_{t,\mu})\ln \mathcal{Z}_{t,\epsilon}\big]\\
&=\frac{1}{\sqrt{n(1-t)}}\sum_{i=1}^n \mathbb{E}\Big[\int d\mathbf{Y}_t d\mathbf{Y}'_t e^{-\mathcal{H}_{t,\epsilon}(\mathbf{X}^*,\mathbf{W}^*;\mathbf{Y}_t,\mathbf{Y}'_t,\mathbf{\Phi},\mathbf{V})}\Phi_{\mu i}X_i^* u'_{Y_{t,\mu}}(S_{t,\mu})\ln \mathcal{Z}_{t,\epsilon}\Big]\\
&=\frac{1}{n}\sum_{i=1}^n\Big(\mathbb{E}\big[(X_i^*)^2\big(u''_{Y_{t,\mu}}(S_{t,\mu})+u'_{Y_{t,\mu}}(S_{t,\mu})^2\big)\ln \mathcal{Z}_{t,\epsilon}\big]+\mathbb{E}\big\langle X_i^* x_i u'_{Y_{t,\mu}}(S_{t,\mu})u'_{Y_{t,\mu}}(s_{t,\mu})\big\rangle_{n,t,\epsilon}\Big)\\
&=\mathbb{E}\Big[\frac{1}{n}\sum_{i=1}^n (X_i^*)^2\frac{P''_{\rm out}(Y_{t,\mu}|S_{t,\mu})}{P_{\rm out}(Y_{t,\mu}|S_{t,\mu})}\ln \mathcal{Z}_{t,\epsilon}\Big]+\mathbb{E}\Big\langle\frac{1}{n}\sum_{i=1}^n X_i^* x_i u'_{Y_{t,\mu}}(S_{t,\mu})u'_{Y_{t,\mu}}(s_{t,\mu})\Big\rangle_{n,t,\epsilon}, \qquad (157)
\end{aligned}$$

where we used the identity

$$u''_{Y_{t,\mu}}(x)+u'_{Y_{t,\mu}}(x)^2=\frac{P''_{\rm out}(Y_{t,\mu}|x)}{P_{\rm out}(Y_{t,\mu}|x)}\,. \qquad (158)$$

We now compute the second term of the right hand side of (156). Using again Gaussian integrations by parts but this time w.r.t $V_\mu, W_\mu^* \overset{\rm iid}{\sim} \mathcal{N}(0,1)$ as well as the previous formula, we obtain similarly

$$\begin{aligned}
&\mathbb{E}\Big[\Big(\frac{q(t)}{\sqrt{R_2(t)}}V_\mu+\frac{\rho-q(t)}{\sqrt{\rho t-R_2(t)+2s_n}}W_\mu^*\Big)u'_{Y_{t,\mu}}(S_{t,\mu})\ln \mathcal{Z}_{t,\epsilon}\Big]\\
&=\mathbb{E}\Big[\int d\mathbf{Y}_t d\mathbf{Y}'_t e^{-\mathcal{H}_{t,\epsilon}(\mathbf{X}^*,\mathbf{W}^*;\mathbf{Y}_t,\mathbf{Y}'_t,\mathbf{\Phi},\mathbf{V})}\Big(\frac{q(t)}{\sqrt{R_2(t)}}V_\mu+\frac{\rho-q(t)}{\sqrt{\rho t-R_2(t)+2s_n}}W_\mu^*\Big)u'_{Y_{t,\mu}}(S_{t,\mu})\ln \mathcal{Z}_{t,\epsilon}\Big]\\
&=\mathbb{E}\Big[\rho\frac{P''_{\rm out}(Y_{t,\mu}|S_{t,\mu})}{P_{\rm out}(Y_{t,\mu}|S_{t,\mu})}\ln \mathcal{Z}_{t,\epsilon}\Big]+\mathbb{E}\big\langle q(t)u'_{Y_{t,\mu}}(S_{t,\mu})u'_{Y_{t,\mu}}(s_{t,\mu})\big\rangle_{n,t,\epsilon}\,. \qquad (159)
\end{aligned}$$

Combining equations (156), (157) and (159) together, we have

$$\begin{aligned}
&-\mathbb{E}\Big[\frac{dS_{t,\mu}}{dt}u'_{Y_{t,\mu}}(S_{t,\mu})\ln \mathcal{Z}_{t,\epsilon}\Big]\\
&=\frac{1}{2}\mathbb{E}\Big[\frac{P''_{\rm out}(Y_{t,\mu}|S_{t,\mu})}{P_{\rm out}(Y_{t,\mu}|S_{t,\mu})}\Big(\frac{1}{n}\sum_{i=1}^n (X_i^*)^2-\rho\Big)\ln \mathcal{Z}_{t,\epsilon}\Big]+\frac{1}{2}\mathbb{E}\Big\langle\Big(\frac{1}{n}\sum_{i=1}^n X_i^* x_i-q(t)\Big)u'_{Y_{t,\mu}}(S_{t,\mu})u'_{Y_{t,\mu}}(s_{t,\mu})\Big\rangle_{n,t,\epsilon}.
\end{aligned}$$

As seen from (154), (155) it remains to compute $\mathbb{E}[X_j^*(Y'_{t,j}-\sqrt{R_1(t)}X_j^*)\ln \mathcal{Z}_{t,\epsilon}]$. Recalling that for $1\le j\le n$, $Y'_{t,j}-\sqrt{R_1(t)}X_j^*=Z'_j$ and then using again a Gaussian integration by parts w.r.t $Z'_j\sim\mathcal{N}(0,1)$ we obtain

$$\begin{aligned}
\mathbb{E}\big[X_j^*(Y'_{t,j}-\sqrt{R_1(t)}X_j^*)\ln \mathcal{Z}_{t,\epsilon}\big]&=\mathbb{E}\big[X_j^* Z'_j\ln \mathcal{Z}_{t,\epsilon}\big]=\mathbb{E}\Big[X_j^* Z'_j\ln\int dP_0(\mathbf{x})\mathcal{D}\mathbf{w}\,e^{-\mathcal{H}_{t,\epsilon}(\mathbf{x},\mathbf{w};\mathbf{Y}_t,\mathbf{Y}'_t,\mathbf{\Phi},\mathbf{V})}\Big]\\
&=\mathbb{E}\Big[X_j^* Z'_j\ln\int dP_0(\mathbf{x})\mathcal{D}\mathbf{w}\,\exp\Big\{\sum_{\mu=1}^m u_{Y_{t,\mu}}(s_{t,\mu})-\frac{1}{2}\sum_{i=1}^n\big(\sqrt{R_1(t)}X_i^*+Z'_i-\sqrt{R_1(t)}\,x_i\big)^2\Big\}\Big]\\
&=-\mathbb{E}\big[X_j^*\big\langle\sqrt{R_1(t)}(X_j^*-x_j)+Z'_j\big\rangle_{n,t,\epsilon}\big]\\
&=-\sqrt{R_1(t)}\big(\rho-\mathbb{E}\langle X_j^* x_j\rangle_{n,t,\epsilon}\big)\,. \qquad (160)
\end{aligned}$$

Thus, by taking the sum,

$$-\frac{r(t)}{2\sqrt{R_1(t)}}\mathbb{E}\Big[\frac{1}{n}\sum_{i=1}^n X_i^*(Y'_{t,i}-\sqrt{R_1(t)}X_i^*)\ln \mathcal{Z}_{t,\epsilon}\Big]=\frac{r(t)\rho}{2}-\frac{r(t)}{2}\mathbb{E}\Big\langle\frac{1}{n}\sum_{i=1}^n X_i^* x_i\Big\rangle_{n,t,\epsilon}. \qquad (161)$$

Therefore, for all $t \in (0,1)$,

$$T_1 = \frac{1}{2}\mathbb{E}\Big[\frac{1}{\sqrt{n}}\sum_{\mu=1}^{m}\frac{P''_{\rm out}(Y_{t,\mu}|S_{t,\mu})}{P_{\rm out}(Y_{t,\mu}|S_{t,\mu})}\Big(\frac{1}{\sqrt{n}}\sum_{i=1}^{n}((X_i^*)^2-\rho)\Big)\frac{1}{n}\ln\mathcal{Z}_{t,\epsilon}\Big] + \frac{r(t)\rho}{2} - \frac{r(t)q(t)}{2}$$
$$+\frac{1}{2}\mathbb{E}\Big\langle\Big(\frac{1}{n}\sum_{\mu=1}^{m}u'_{Y_{t,\mu}}(S_{t,\mu})u'_{Y_{t,\mu}}(s_{t,\mu})-r(t)\Big)\Big(\frac{1}{n}\sum_{i=1}^{n}X_i^*x_i-q(t)\Big)\Big\rangle_{n,t,\epsilon}. \tag{162}$$

To obtain (151), it remains to show that $T_2 = 0$. This is a direct consequence of the Nishimori identity (see Appendix A.1):

$$T_2 = \frac{1}{n}\mathbb{E}\big\langle\mathcal{H}'_{t,\epsilon}(\mathbf{x},\mathbf{w};\mathbf{Y}_t,\mathbf{Y}'_t,\mathbf{\Phi})\big\rangle_{n,t,\epsilon} = \frac{1}{n}\mathbb{E}\,\mathcal{H}'_{t,\epsilon}(\mathbf{X}^*,\mathbf{W}^*;\mathbf{Y}_t,\mathbf{Y}'_t,\mathbf{\Phi}) = 0\,. \tag{163}$$

For obtaining the Lemma, it remains to show that $A_{n,\epsilon}$ goes to 0 uniformly in $t \in [0,1]$.

A.5.2 Proof that $A_{n,\epsilon}$ vanishes as $n \to \infty$

We now consider the final step, that is showing that $A_{n,\epsilon}$ given by (152) vanishes in the $n \to \infty$ limit uniformly in $t \in [0,1]$ under conditions (H1)-(H2)-(H3). First we show that

$$\mathbb{E}\Big[\frac{1}{\sqrt{n}}\sum_{\mu=1}^{m}\frac{P''_{\rm out}(Y_{t,\mu}|S_{t,\mu})}{P_{\rm out}(Y_{t,\mu}|S_{t,\mu})}\Big(\frac{1}{\sqrt{n}}\sum_{i=1}^{n}\big((X_i^*)^2-\rho\big)\Big)\Big] = 0\,. \tag{164}$$

Once this is done, we use the fact that $\frac{1}{n}\ln\mathcal{Z}_{t,\epsilon}$ concentrates around $f_{n,\epsilon}(t)$ to prove that $A_{n,\epsilon}$ converges to 0 as $n \to \infty$. We start by noticing the simple fact that for all $s \in \mathbb{R}$, $\int P''_{\rm out}(y|s)dy = 0$. Consequently, for $\mu \in \{1,\dots,m\}$,

$$\mathbb{E}\Big[\frac{P''_{\rm out}(Y_{t,\mu}|S_{t,\mu})}{P_{\rm out}(Y_{t,\mu}|S_{t,\mu})}\,\Big|\,\mathbf{X}^*,\mathbf{S}_t\Big] = \int dY_{t,\mu}P''_{\rm out}(Y_{t,\mu}|S_{t,\mu}) = 0\,. \tag{165}$$

Thus, using the "tower property" of the conditionnal expectation:

$$\mathbb{E}\Big[\Big(\sum_{i=1}^{n}((X_i^*)^2-\rho)\Big)\sum_{\mu=1}^{m}\frac{P''_{\rm out}(Y_{t,\mu}|S_{t,\mu})}{P_{\rm out}(Y_{t,\mu}|S_{t,\mu})}\Big] = \mathbb{E}\Big[\Big(\sum_{i=1}^{n}\big((X_i^*)^2-\rho\big)\Big)\mathbb{E}\Big[\sum_{\mu=1}^{m}\frac{P''_{\rm out}(Y_{t,\mu}|S_{t,\mu})}{P_{\rm out}(Y_{t,\mu}|S_{t,\mu})}\,\Big|\,\mathbf{X}^*,\mathbf{S}_t\Big]\Big] = 0$$

which gives (164). We now show that $A_{n,\epsilon}$ goes to 0 uniformly in $t \in [0,1]$ as $n \to \infty$. Using successively (164) and the Cauchy-Schwarz inequality, we have

$$|A_{n,\epsilon}| = \Big|\mathbb{E}\Big[\frac{1}{\sqrt{n}}\sum_{\mu=1}^{m}\frac{P''_{\rm out}(Y_{t,\mu}|S_{t,\mu})}{P_{\rm out}(Y_{t,\mu}|S_{t,\mu})}\Big(\frac{1}{\sqrt{n}}\sum_{i=1}^{n}\big((X_i^*)^2-\rho\big)\Big)\Big(\frac{1}{n}\ln\mathcal{Z}_{t,\epsilon}-f_{n,\epsilon}(t)\Big)\Big]\Big|$$
$$\le \mathbb{E}\Big[\Big(\frac{1}{\sqrt{n}}\sum_{\mu=1}^{m}\frac{P''_{\rm out}(Y_{t,\mu}|S_{t,\mu})}{P_{\rm out}(Y_{t,\mu}|S_{t,\mu})}\Big)^2\Big(\frac{1}{\sqrt{n}}\sum_{i=1}^{n}\big((X_i^*)^2-\rho\big)\Big)^2\Big]^{1/2}\mathbb{E}\Big[\Big(\frac{1}{n}\ln\mathcal{Z}_{t,\epsilon}-f_{n,\epsilon}(t)\Big)^2\Big]^{1/2}. \tag{166}$$

Using again the "tower property" of conditional expectations

$$\mathbb{E}\Big[\Big(\sum_{\mu=1}^{m}\frac{P''_{\rm out}(Y_{t,\mu}|S_{t,\mu})}{P_{\rm out}(Y_{t,\mu}|S_{t,\mu})}\Big)^2\Big(\sum_{i=1}^{n}((X_i^*)^2-\rho)\Big)^2\Big]$$
$$=\mathbb{E}\Big[\Big(\sum_{i=1}^{n}((X_i^*)^2-\rho)\Big)^2\mathbb{E}\Big[\Big(\sum_{\mu=1}^{m}\frac{P''_{\rm out}(Y_{t,\mu}|S_{t,\mu})}{P_{\rm out}(Y_{t,\mu}|S_{t,\mu})}\Big)^2\,\Big|\,\mathbf{X}^*,\mathbf{S}_t\Big]\Big]\,. \tag{167}$$

Now, using the fact that conditionally on $\mathbf{S}_t$, the random variables $\big(\frac{P''_{\rm out}(Y_{t,\mu}|S_{t,\mu})}{P_{\rm out}(Y_{t,\mu}|S_{t,\mu})}\big)_{1\le\mu\le m}$ are i.i.d. and centered, we have

$$\mathbb{E}\Big[\Big(\sum_{\mu=1}^{m}\frac{P''_{\rm out}(Y_{t,\mu}|S_{t,\mu})}{P_{\rm out}(Y_{t,\mu}|S_{t,\mu})}\Big)^2\,\Big|\,\mathbf{X}^*,\mathbf{S}_t\Big]=\mathbb{E}\Big[\Big(\sum_{\mu=1}^{m}\frac{P''_{\rm out}(Y_{t,\mu}|S_{t,\mu})}{P_{\rm out}(Y_{t,\mu}|S_{t,\mu})}\Big)^2\,\Big|\,\mathbf{S}_t\Big]=m\mathbb{E}\Big[\Big(\frac{P''_{\rm out}(Y_1|S_{t,1})}{P_{\rm out}(Y_1|S_{t,1})}\Big)^2\,\Big|\,\mathbf{S}_t\Big]\,. \tag{168}$$

Under condition (H2), it is not difficult to show that there exists a constant $C>0$ such that

$$\mathbb{E}\Big[\Big(\frac{P''_{\rm out}(Y_{t,1}|S_{t,1})}{P_{\rm out}(Y_{t,1}|S_{t,1})}\Big)^2\,\Big|\,\mathbf{S}_t\Big]\le C\,. \tag{169}$$

Combining now (169), (168) and (167) we obtain that

$$\mathbb{E}\Big[\Big(\sum_{\mu=1}^{m}\frac{P''_{\rm out}(Y_{t,\mu}|S_{t,\mu})}{P_{\rm out}(Y_{t,\mu}|S_{t,\mu})}\Big)^2\Big(\sum_{i=1}^{n}((X_i^*)^2-\rho)\Big)^2\Big]\le mC\,\mathbb{E}\Big[\Big(\sum_{i=1}^{n}((X_i^*)^2-\rho)\Big)^2\Big]=mnC\,\mathrm{Var}\big((X_1^*)^2\big)\,.$$

Going back to (166), therefore there exists a constant $C'>0$ such that

$$|A_{n,\epsilon}|\le C'\,\mathbb{E}\Big[\Big(\frac{1}{n}\ln\mathcal{Z}_{t,\epsilon}-f_{n,\epsilon}(t)\Big)^2\Big]^{1/2}\,. \tag{170}$$

By Theorem 6 we have $\mathbb{E}[(n^{-1}\ln\mathcal{Z}_{t,\epsilon}-f_{n,\epsilon}(t))^2]\to 0$ as $n\to\infty$ uniformly in $t\in[0,1]$. Thus $A_{n,\epsilon}$ goes to 0 as $n\to\infty$ uniformly in $t\in[0,1]$, ϵ and w.r.t. the choice of the interpolation functions. This ends the proof of Proposition 3.

A.6 Boundedness of an overlap fluctuation

In this appendix we show that the "overlap fluctuation"

$$\mathbb{E}\Big\langle\Big(\frac{1}{n}\sum_{\mu=1}^{m}u'_{Y_{t,\mu}}(S_{t,\mu})u'_{Y_{t,\mu}}(s_{t,\mu})-r_\epsilon(t)\Big)^2\Big\rangle_{n,t,\epsilon}\le 2r_{\rm max}^2+2\mathbb{E}\Big\langle\Big(\frac{1}{n}\sum_{\mu=1}^{m}u'_{Y_{t,\mu}}(S_{t,\mu})u'_{Y_{t,\mu}}(s_{t,\mu})\Big)^2\Big\rangle_{n,t,\epsilon} \tag{171}$$

is bounded uniformly in t under hypothesis (H2) on φ. From the representation (3) (recall we consider $\Delta=1$ in Sec. 4)

$$u_{Y_{t,\mu}}(s)=\ln P_{\rm out}(Y_{t,\mu}|s)=\ln\int dP_A(\mathbf{a}_\mu)\frac{1}{\sqrt{2\pi}}e^{-\frac{1}{2}(Y_{t,\mu}-\varphi(s,\mathbf{a}_\mu))^2} \tag{172}$$

and thus

$$u'_{Y_{t,\mu}}(s)=\frac{\int dP_A(\mathbf{a}_\mu)(Y_{t,\mu}-\varphi(s,\mathbf{a}_\mu))\varphi'(s,\mathbf{a}_\mu)e^{-\frac{1}{2}(Y_{t,\mu}-\varphi(s,\mathbf{a}_\mu))^2}}{\int dP_A(\mathbf{a}_\mu)e^{-\frac{1}{2}(Y_{t,\mu}-\varphi(s,\mathbf{a}_\mu))^2}} \tag{173}$$

where φ' is the derivative w.r.t. the first argument. From (1) at $\Delta = 1$ we get $|Y_{t,\mu}| \le \sup|\varphi| + |Z_\mu|$, where the supremum is taken over both arguments of φ, and thus immediately obtain for all $s \in \mathbb{R}$

$$|u'_{Y_{t,\mu}}(s)| \le (2\sup|\varphi| + |Z_\mu|)\sup|\varphi'|\,. \tag{174}$$

From (174) and (171) we see that it suffices to check that

$$\frac{m^2}{n^2}\mathbb{E}\big[\big((2\sup|\varphi| + |Z_\mu|)^2(\sup|\varphi'|)^2\big)^2\big] \le C(\varphi,\alpha)$$

where $C(\varphi,\alpha)$ is a constant depending only on φ and α. This is easily seen by expanding all squares and using that m/n is bounded.

A.7 Proof of Proposition 6

The continuity and differentiability properties of F_n follow from the standard theorems of continuity and derivation under the integral sign. The domination hypotheses are easily verified because we are working under hypotheses (H1)-(H2).

The overlap $\mathbb{E}\langle Q\rangle_{n,t,\epsilon}$ is related to the minimum mean-square error by

$$\frac{1}{n}\mathrm{MMSE}(\mathbf{X}^*|\mathbf{Y}_t,\mathbf{Y}'_t,\mathbf{V},\mathbf{\Phi}) = \frac{1}{n}\mathbb{E}\Big[\big\|\mathbf{X}^* - \mathbb{E}[\mathbf{X}^*|\mathbf{Y}_t,\mathbf{Y}'_t,\mathbf{V},\mathbf{\Phi}]\big\|^2\Big] = \frac{1}{n}\mathbb{E}\Big[\big\|\mathbf{X}^* - \langle\mathbf{x}\rangle_{n,t,\epsilon}\big\|^2\Big] = \rho - \mathbb{E}\langle Q\rangle_{n,t,\epsilon}.$$

Since the left hand side belongs to $[0,\rho]$, we obtain that $\mathbb{E}\langle Q\rangle_{n,t,\epsilon} \in [0,\rho]$.

It remains therefore to prove that $\mathrm{MMSE}(\mathbf{X}^*|\mathbf{Y}_t,\mathbf{Y}'_t,\mathbf{V},\mathbf{\Phi})$ is separately non-increasing in R_1 and R_2. R_1 only appears in the definition of $\mathbf{Y}'_t$. Recall that $\mathbf{Y}'_t = \sqrt{R_1}\,\mathbf{X}^* + \mathbf{Z}'$, where $\mathbf{Z}'$ is a standard Gaussian vector, so $\mathrm{MMSE}(\mathbf{X}^*|\mathbf{Y}_t,\mathbf{Y}'_t,\mathbf{V},\mathbf{\Phi})$ is obviously a non-increasing function of R_1.

R_2 only plays a role in $\mathbf{Y}_t \sim P_{\rm out}(\cdot\,|\,\sqrt{(1-t)/n}\,\mathbf{\Phi}\mathbf{X}^* + \sqrt{R_2}\,\mathbf{V} + \sqrt{\rho t - R_2 + 2s_n}\,\mathbf{W}^*)$. Let $0 < r_2 \le r'_2 < \rho t$. Let $\mathbf{V}' \sim \mathcal{N}(0,\mathbf{I}_m)$, independently of everything else. Define

$$\widetilde{\mathbf{Y}}_t \sim P_{\rm out}\Big(\cdot\,\Big|\,\sqrt{\frac{1-t}{n}}\,\mathbf{\Phi}\mathbf{X}^* + \sqrt{r_2}\,\mathbf{V} + \sqrt{r'_2 - r_2}\,\mathbf{V}' + \sqrt{\rho t - r'_2 + 2s_n}\,\mathbf{W}^*\Big),$$

independently of everything else. Now notice that

$$\begin{aligned}\mathrm{MMSE}(\mathbf{X}^*|\mathbf{Y}_t,\mathbf{Y}'_t,\mathbf{V},\mathbf{\Phi})|_{R_2=r_2} &= \mathrm{MMSE}(\mathbf{X}^*|\widetilde{\mathbf{Y}}_t,\mathbf{Y}'_t,\mathbf{V},\mathbf{\Phi}),\\ \mathrm{MMSE}(\mathbf{X}^*|\mathbf{Y}_t,\,\mathbf{Y}'_t,\mathbf{V},\mathbf{\Phi})|_{R_2=r'_2} &= \mathrm{MMSE}(\mathbf{X}^*|\widetilde{\mathbf{Y}}_t,\mathbf{Y}'_t,\mathbf{V},\mathbf{V}',\mathbf{\Phi}),\end{aligned}$$

which implies of course that $\mathrm{MMSE}(\mathbf{X}^*|\mathbf{Y}_t,\mathbf{Y}'_t,\mathbf{V},\mathbf{\Phi})|_{R_2=r_2} \ge \mathrm{MMSE}(\mathbf{X}^*|\mathbf{Y}_t,\mathbf{Y}'_t,\mathbf{V},\mathbf{\Phi})|_{R_2=r'_2}$. We have proved that $\mathrm{MMSE}(\mathbf{X}^*|\mathbf{Y}_t,\mathbf{Y}'_t,\mathbf{V},\mathbf{\Phi})$ is a non-increasing function of R_2.

Appendix B: Some properties of the scalar channels

B.1 The additive Gaussian scalar channel

We recall some properties (see [55] and [57] for proofs) of the free entropy of the first scalar channel (17).

Proposition 17. *Let $X_0 \sim P_0$ be a real random variable with finite second moment. Let $r \ge 0$ and $Y_0 = \sqrt{r}X_0 + Z_0$, where $Z_0 \sim \mathcal{N}(0,1)$ is independent from X_0. Then the function*

$$\psi_{P_0} : r \mapsto \mathbb{E}\ln\int dP_0(x)e^{\sqrt{r}\,Y_0x - rx^2/2}$$

is convex, differentiable, non-decreasing and $\frac{1}{2}\mathbb{E}[X_0^2]$*-Lipschitz on* $\mathbb{R}_+$*. Moreover,* ψ_{P_0} *is strictly convex, if* P_0 *is not a Dirac measure.*

B.2 The non-linear scalar channel

We prove here some properties of the free entropy of the second scalar channel (20), where $V, W^* \overset{\text{iid}}{\sim} \mathcal{N}(0,1)$ and

$$Y^{(q)} \sim P_{\text{out}}\big(\,\cdot\,\big|\sqrt{q}\,V + \sqrt{\rho-q}\,W^*\big)\,. \tag{175}$$

In this channel, the statistician observes V and $Y^{(q)}$ and wants to recover W^*. Recall that by definition $\mathcal{I}_{P_{\text{out}}}(q) = I(W^*;Y^{(q)}|V) = \Psi_{P_{\text{out}}}(\rho) - \Psi_{P_{\text{out}}}(q)$ so the properties we will prove on $\Psi_{P_{\text{out}}}$ can be directly translated for $\mathcal{I}_{P_{\text{out}}}$, and vice-versa.

Proposition 18. *Suppose that for all* $x \in \mathbb{R}$, $P_{\text{out}}(\cdot\,|\,x)$ *is the law of* $\varphi(x,A) + \sqrt{\Delta}Z$ *where* $\Delta > 0$, $\varphi : \mathbb{R}\times\mathbb{R}^{k_A} \to \mathbb{R}$ *is a measurable function and* $(Z,A) \sim \mathcal{N}(0,1)\otimes P_A$*, for some probability distribution* P_A *over* $\mathbb{R}^{k_A}$*. In that case* P_{out} *admits a density given by*

$$P_{\text{out}}(y|x) = \frac{1}{\sqrt{2\pi\Delta}}\int dP_A(\boldsymbol{a})e^{-\frac{1}{2\Delta}(y-\varphi(x,\boldsymbol{a}))^2}\,.$$

Assume that φ *is bounded and* $\mathcal{C}^2$ *with respect to its first coordinate, with bounded first and second derivatives. Then* $q \mapsto \Psi_{P_{\text{out}}}(q)$ *is convex,* $\mathcal{C}^2$ *and non-decreasing on* $[0,\rho]$*.*

Proof. Let $V, W^* \overset{\text{iid}}{\sim} \mathcal{N}(0,1)$ and $Y^{(q)}$ be the output of the scalar channel given by (175). Then for all $q \in [0,\rho]$,

$$\Psi_{P_{\text{out}}}(q) = \mathbb{E}\ln\int dw\,\frac{e^{-\frac{w^2}{2}}}{\sqrt{2\pi}}P_{\text{out}}\big(Y^{(q)}|\sqrt{q}\,V + \sqrt{\rho-q}\,w\big)$$

Under the hypotheses we made on φ, we will be able to use continuity and differentiation under the expectation, because all the domination hypotheses will be easily verified. It is thus easy to check that $\Psi_{P_{\text{out}}}$ is continuous on $[0,\rho]$.

We compute now the first derivative. Recall that $\langle - \rangle_{\text{sc}}$, defined in (53), denotes the posterior distribution of W^* given $Y^{(q)}$. We will use the notation $u_y(x) = \ln P_{\text{out}}(y|x)$. For $q \in (0,\rho)$ we have

$$\begin{aligned}\Psi'_{P_{\text{out}}}(q) &= \frac{1}{2}\mathbb{E}\Big\langle u'_{Y^{(q)}}(\sqrt{q}\,V + \sqrt{\rho-q}\,w)u'_{Y^{(q)}}(\sqrt{q}\,V + \sqrt{\rho-q}\,W^*)\Big\rangle_{\text{sc}} \\ &= \frac{1}{2}\mathbb{E}\Big\langle u'_{Y^{(q)}}(\sqrt{q}\,V + \sqrt{\rho-q}\,w)\Big\rangle_{\text{sc}}^2 \geq 0\,,\end{aligned}$$

where $w \sim \langle - \rangle_{\text{sc}}$, independently of everything else. $\Psi_{P_{\text{out}}}$ is therefore non-decreasing. Using the boundedness assumption on φ and its derivatives, it is not difficult to check that $\Psi'_{P_{\text{out}}}$ is indeed bounded.

We will now compute $\Psi''_{P_{\text{out}}}$. To lighten the notations, we write $u'(w)$ for $u'_{Y^{(q)}}(\sqrt{q}\,V + \sqrt{\rho-q}\,w)$. We compute

$$\begin{aligned}\partial_q\mathbb{E}\Big\langle u'(w)u'(W^*)\Big\rangle_{\text{sc}} = \mathbb{E}\Big[\Big(\frac{1}{2\sqrt{q}}V - \frac{1}{2\sqrt{\rho-q}}W^*\Big)u'(W^*)\Big\langle u'(w)u'(W^*)\Big\rangle_{\text{sc}}\Big] &\qquad (A)\\ +2\mathbb{E}\Big\langle\Big(\frac{1}{2\sqrt{q}}V - \frac{1}{2\sqrt{\rho-q}}W^*\Big)u''(W^*)u'(w)\Big\rangle_{\text{sc}} &\qquad (B)\\ +\mathbb{E}\Big\langle\Big(\frac{1}{2\sqrt{q}}V - \frac{1}{2\sqrt{\rho-q}}W^*\Big)u'(W^*)^2u'(w)\Big\rangle_{\text{sc}} &\qquad (C)\\ -\mathbb{E}\Big\langle u'(W^*)u'(w)\Big\rangle_{\text{sc}}\Big\langle\Big(\frac{1}{2\sqrt{q}}V - \frac{1}{2\sqrt{\rho-q}}w\Big)u'(w)\Big\rangle_{\text{sc}} &\qquad (D)\end{aligned} \tag{176}$$

Notice that $(A) = (C)$. We compute, using Gaussian integration by parts and the Nishimori identity (Proposition 12)

$$
\begin{aligned}
(A) &= \frac{1}{2}\mathbb{E}\Big[u'(W^*)\Big\langle u'(W^*)u''(w)\Big\rangle_{\rm sc}\Big] + \frac{1}{2}\mathbb{E}\Big[u'(W^*)\Big\langle u'(W^*)u'(w)^2\Big\rangle_{\rm sc}\Big] \\
&\quad - \frac{1}{2}\mathbb{E}\Big[u'(W^*)\Big\langle u'(W^*)u'(w)\Big\rangle_{\rm sc}\Big\langle u'(w)\Big\rangle_{\rm sc}\Big] \qquad (177)
\end{aligned}
$$

$$
(B) = \mathbb{E}\Big\langle u''(W^*)u''(w)\Big\rangle_{\rm sc} + \mathbb{E}\Big\langle u''(W^*)u'(w)^2\Big\rangle_{\rm sc} - \mathbb{E}\Big\langle u''(W^*)u'(w)\Big\rangle_{\rm sc}\Big\langle u'(w)\Big\rangle_{\rm sc} \qquad (178)
$$

$$
\begin{aligned}
(D) &= -\mathbb{E}\Big\langle\Big(\frac{1}{2\sqrt{q}}V - \frac{1}{2\sqrt{\rho-q}}W^*\Big)u'(W^*)u'(w^{(1)})u'(w^{(2)})\Big\rangle_{\rm sc} \\
&= -\mathbb{E}\langle u'(W^*)u''(w^{(1)})u'(w^{(2)})\rangle_{\rm sc} - \mathbb{E}\langle u'(W^*)u'(w^{(1)})^2u'(w^{(2)})\rangle_{\rm sc} \\
&\quad + \mathbb{E}\langle u'(W^*)u'(w^{(1)})u'(w^{(2)})\rangle_{\rm sc}\langle u'(w)\rangle_{\rm sc} \qquad (179)
\end{aligned}
$$

We now replace (177), (178) and (179) in (176):

$$
\begin{aligned}
2\Psi''_{P_{\rm out}}(q) &= \mathbb{E}\Big\langle u'(W^*)^2u''(w)\Big\rangle_{\rm sc} + \mathbb{E}\Big\langle u'(W^*)^2u'(w)^2\Big\rangle_{\rm sc} - \mathbb{E}\Big\langle u'(W^*)^2u'(w^{(1)})u'(w^{(2)})\Big\rangle_{\rm sc} \\
&\quad + \mathbb{E}\Big\langle u''(W^*)u''(w)\Big\rangle_{\rm sc} + \mathbb{E}\Big\langle u''(W^*)u'(w)^2\Big\rangle_{\rm sc} - \mathbb{E}\Big\langle u''(W^*)u'(w^{(1)})u'(w^{(2)})\Big\rangle_{\rm sc} \\
&\quad - \mathbb{E}\langle u'(W^*)u''(w^{(1)})u'(w^{(2)})\rangle_{\rm sc} - \mathbb{E}\langle u'(W^*)u'(w^{(1)})^2u'(w^{(2)})\rangle_{\rm sc} + \mathbb{E}\langle u'(w)\rangle_{\rm sc}^4 \,.
\end{aligned}
$$

Using the identity $u_Y''(x) + u_Y'(x)^2 = \frac{P''_{\rm out}(Y|x)}{P_{\rm out}(Y|x)}$, this factorizes and gives

$$
\Psi''_{P_{\rm out}}(q) = \frac{1}{2}\mathbb{E}\Big[\Big(\Big\langle\frac{P''_{\rm out}(Y|\sqrt{q}\,V+\sqrt{\rho-q}\,w)}{P_{\rm out}(Y|\sqrt{q}\,V+\sqrt{\rho-q}w)}\Big\rangle_{\rm sc} - \Big\langle u'_{Y^{(q)}}(\sqrt{q}\,V+\sqrt{\rho-q}\,w)\Big\rangle_{\rm sc}^2\Big)^2\Big] \geq 0\,. \qquad (180)
$$

$\Psi_{P_{\rm out}}$ is thus convex on $[0,\rho]$. It is not difficult to verify (by standard arguments of continuity under the integral) that $\Psi''_{P_{\rm out}}$ is continuous on $[0,\rho]$, which gives that $\Psi_{P_{\rm out}}$ is $\mathcal{C}^2$ on its domain. □

Proposition 19. *Suppose that for all $x\in\mathbb{R}$, $P_{\rm out}(\cdot\,|\,x)$ is the law of $\varphi(x,A)+\sqrt{\Delta}Z$ where $\varphi:\mathbb{R}\times\mathbb{R}^{k_A}\to\mathbb{R}$ is a measurable function and $(Z,A)\sim\mathcal{N}(0,1)\otimes P_A$, for some probability distribution P_A over $\mathbb{R}^{k_A}$. Assume also that*

$$
\mathbb{E}[\varphi(\sqrt{\rho}Z,A)^2] < \infty\,, \qquad (181)
$$

and that we are in one of the following cases:

(i) $\Delta > 0$.

(ii) $\Delta = 0$ *and φ takes values in $\mathbb{N}$.*

Then $q\mapsto\Psi_{P_{\rm out}}(q)$ is continuous, convex and non-decreasing over $[0,\rho]$.

Notice that (181) is for instance verified under hypotheses (h1)-(h2)-(h3)-(h4), see Proposition 16.

Proof. We deduce Proposition 19 from Proposition 18 above by an approximation procedure. Since $\Psi_{P_{\rm out}} = \Psi_{P_{\rm out}}(\rho) - \mathcal{I}_{P_{\rm out}}$, we will work with the mutual information $\mathcal{I}_{P_{\rm out}}$. Let us define $U^{(q)} = \varphi\big(\sqrt{q}\,V+\sqrt{\rho-q}\,W^*, A\big)$ and $Y^{(q)} = U^{(q)} + \sqrt{\Delta}Z$.

We start by proving Proposition 19 under the assumption (i). Let $\epsilon>0$. By density of the $\mathcal{C}^\infty$ functions with compact support in L^2 (see for instance Corollary 4.2.2 from [58]), one can find a $\mathcal{C}^\infty$ function $\widehat{\varphi}$ with compact support, such that

$$
\mathbb{E}\Big[\big(\varphi(\sqrt{\rho}\,Z,A) - \widehat{\varphi}(\sqrt{\rho}\,Z,A)\big)^2\Big] \leq \epsilon^2\,.
$$

Let us write $\widehat{U}^{(q)} = \widehat{\varphi}(\sqrt{q}\,V + \sqrt{\rho - q}\,W^*, A)$ and $\widehat{Y}^{(q)} = \widehat{U} + \sqrt{\Delta}Z$. We have by the chain rule for the mutual information

$$\begin{aligned} I(U^{(q)}; Y^{(q)}|V) &= I(W^*, U^{(q)}; Y^{(q)}|V) \\ &= I(U^{(q)}; Y^{(q)}|V, W^*) + I(W^*; Y^{(q)}|V) = I(U^{(q)}; Y^{(q)}|V, W^*) + \mathcal{I}_{P_{\rm out}}(q) \end{aligned} \tag{182}$$

and similarly, $\mathcal{I}_{\widehat{P}_{\rm out}}(q) = I(\widehat{U}^{(q)}; \widehat{Y}^{(q)}|V) - I(\widehat{U}^{(q)}; \widehat{Y}^{(q)}|V, W^*)$. By Proposition 14, there exists a constant $C > 0$ such that

$$|I(\widehat{U}^{(q)}; \widehat{Y}^{(q)}|V) - I(U^{(q)}; Y^{(q)}|V)| \leq C\epsilon \quad \text{and} \quad |I(\widehat{U}^{(q)}; \widehat{Y}^{(q)}|V, W^*) - I(U^{(q)}; Y^{(q)}|V, W^*)| \leq C\epsilon\,.$$

We get that for all $q \in [0, \rho]$, $|\mathcal{I}_{P_{\rm out}}(q) - \mathcal{I}_{\widehat{P}_{\rm out}}(q)| \leq C\epsilon$. The function $\mathcal{I}_{P_{\rm out}}$ can therefore be uniformly approximated by continuous, concave, non-increasing functions on $[0, \rho]$: $\mathcal{I}_{P_{\rm out}}$ is therefore continuous, concave and non-increasing.

Let us now prove Proposition 19 under the assumption (ii). Under this assumption we have $\mathcal{I}_{P_{\rm out}}(q) = I(W^*; U^{(q)}|V)$ and by the case (i) we know that the function $i_\Delta(q) = I(W^*; U^{(q)} + \sqrt{\Delta}Z|V)$ is concave and non-increasing for all $\Delta > 0$. By Corollary 6 we obtain that for all $q \in [0, \rho]$ and all $\Delta \in (0, 1]$ we have

$$\left|\mathcal{I}_{P_{\rm out}}(q) - i_\Delta(q)\right| \leq 100 e^{-1/(16\Delta)}\,,$$

which proves (by uniform approximation) that $\mathcal{I}_{P_{\rm out}}$ is continuous, concave and non-increasing. □

Proposition 20. *Under the same hypotheses than Proposition 19 above, $\Psi_{\rm out}$ is differentiable over $[0, \rho)$ and for all $q \in [0, \rho)$*

$$\Psi'_{P_{\rm out}}(q) = \frac{1}{2(\rho - q)} \mathbb{E}\langle w \rangle_{\rm sc}^2\,,$$

where we recall that $\langle - \rangle_{\rm sc}$ is defined by (53).

Proof. The fact that $\Psi_{P_{\rm out}}$ is differentiable on $[0, \rho)$ follows from differentiation under the expectation sign. In order to see it, we define $X = \sqrt{q}\,V + \sqrt{\rho - q}\,W^*$. Then, for all $q \in [0, \rho)$:

$$\Psi_{P_{\rm out}}(q) = \mathbb{E} \int dX \frac{1}{\sqrt{2\pi(\rho - q)}} e^{-\frac{(X - \sqrt{q}\,V)^2}{2(\rho - q)}} \int dY P_{\rm out}(Y|X) \ln \int dx \frac{1}{\sqrt{2\pi(\rho - q)}} e^{-\frac{(x - \sqrt{q}\,V)^2}{2(\rho - q)}} P_{\rm out}(Y|x)\,. \tag{183}$$

We are now in a good setting to differentiate under the expectation sign. We have for all $q \in (0, \rho)$,

$$\frac{\partial}{\partial q} \left[\frac{1}{\sqrt{\rho - q}} e^{-\frac{(X - \sqrt{q}\,V)^2}{2(\rho - q)}}\right] = \frac{1}{2\sqrt{\rho - q}} \Big(\frac{1}{\rho - q} - \frac{(X - \sqrt{q}V)^2}{(\rho - q)^2} + \frac{V(X - \sqrt{q}V)}{\sqrt{q}(\rho - q)}\Big) e^{-\frac{(X - \sqrt{q}\,V)^2}{2(\rho - q)}}\,. \tag{184}$$

Thus

$$\begin{aligned} \Psi'_{P_{\rm out}}(q) &= \frac{1}{2}\mathbb{E}\left[\Big(\frac{1}{\rho - q} - \frac{(X - \sqrt{q}\,V)^2}{(\rho - q)^2} + \frac{V(X - \sqrt{q}\,V)}{\sqrt{q}\,(\rho - q)}\Big) \ln \int dx \frac{1}{\sqrt{2\pi(\rho - q)}} e^{-\frac{(x - \sqrt{q}\,V)^2}{2(\rho - q)}} P_{\rm out}(Y|x)\right] \\ &\quad + \frac{1}{2}\mathbb{E}\left\langle \frac{1}{\rho - q} - \frac{(x - \sqrt{q}\,V)^2}{(\rho - q)^2} + \frac{V(x - \sqrt{q}\,V)}{\sqrt{q}\,(\rho - q)} \right\rangle_{\rm sc} \end{aligned}$$

where the Gibbs brackets $\langle - \rangle_{\rm sc}$ denotes the expectation with respect to $x \sim P(X|Y^{(q)}, V)$. The second term

of the sum above is equal to zero. Indeed by the Nishimori identity (Proposition 12):

$$\mathbb{E}\left\langle \frac{1}{\rho-q} - \frac{(x-\sqrt{q}\,V)^2}{(\rho-q)^2} + \frac{V(x-\sqrt{q}\,V)}{\sqrt{q}\,(\rho-q)} \right\rangle_{\rm sc} = \mathbb{E}\left[\frac{1}{\rho-q} - \frac{(X-\sqrt{q}\,V)^2}{(\rho-q)^2} + \frac{V(X-\sqrt{q}\,V)}{\sqrt{q}\,(\rho-q)}\right] = \frac{1}{\rho-q}\mathbb{E}\left[1-(W^*)^2\right] = 0\,.$$

We now compute, by Gaussian integration by parts with respect to $V \sim \mathcal{N}(0,1)$:

$$\mathbb{E}\left[\frac{V(X-\sqrt{q}\,V)}{\sqrt{q}\,(\rho-q)} \ln \int dx \frac{1}{\sqrt{2\pi(\rho-q)}} e^{-\frac{(x-\sqrt{q}\,V)^2}{2(\rho-q)}} P_{\rm out}(Y^{(q)}|x)\right]$$
$$= \mathbb{E}\left[\frac{-1}{\rho-q}\ln\int dx \frac{e^{-\frac{(x-\sqrt{q}\,V)^2}{2(\rho-q)}}}{\sqrt{2\pi(\rho-q)}} P_{\rm out}(Y^{(q)}|x)\right] + \mathbb{E}\left[\frac{(X-\sqrt{q}\,V)^2}{(\rho-q)^2}\ln\int dx \frac{e^{-\frac{(x-\sqrt{q}\,V)^2}{2(\rho-q)}}}{\sqrt{2\pi(\rho-q)}} P_{\rm out}(Y^{(q)}|x)\right]$$
$$+ \mathbb{E}\left\langle \frac{(X-\sqrt{q}\,V)(x-\sqrt{q}\,V)}{(\rho-q)^2}\right\rangle_{\rm sc}.$$

Bringing all together, we conclude:

$$\Psi'_{P_{\rm out}}(q) = \frac{1}{2}\mathbb{E}\left\langle \frac{(X-\sqrt{q}\,V)(x-\sqrt{q}\,V)}{(\rho-q)^2}\right\rangle_{\rm sc} = \frac{1}{2(\rho-q)}\mathbb{E}\langle w\rangle_{\rm sc}^2\,.$$

This derivative is continuous at $q=0$ thus $\Psi_{P_{\rm out}}$ is differentiable at $q=0$ with derivative given by the same expression. □

Proposition 21. *Assume that the hypotheses of Proposition 19 hold and suppose also that the kernel $P_{\rm out}$ is informative. Then $\Psi_{P_{\rm out}}$ is strictly increasing on $[0,\rho]$.*

Proof. Let us suppose that $\Psi_{P_{\rm out}}$ is not strictly increasing on $[0,\rho]$. There exists thus $q \in (0,\rho)$ such that $\Psi'_{P_{\rm out}}(q) = 0$. This means that $\langle w\rangle_{\rm sc} = 0$ almost surely and therefore that

$$\int_{\mathbb{R}} P_{\rm out}(Y^{(q)}\,|\,\sqrt{q}\,V + \sqrt{\rho-q}\,w) w e^{-w^2/2} dw = 0$$

almost-surely. Let us write $\sigma = \sqrt{\rho-q}$. Consequently,

$$\int_{\mathbb{R}} P_{\rm out}(y\,|\,v+\sigma w) w e^{-w^2/2} dw = 0 \tag{185}$$

for almost all y in $\mathbb{R}$ (if we are under assumption (i)) or all $y \in \mathbb{N}$ (under assumption (ii)) and almost all $v \in \mathbb{R}$. We will now use the following lemma:

Lemma 16. *Let $Z \sim \mathcal{N}(0,1)$ and let $f : \mathbb{R} \to \mathbb{R}$ be a bounded function. Suppose that for almost all $v \in \mathbb{R}$,*

$$\mathbb{E}[Zf(v+Z)] = 0\,.$$

Then, there exists a constant $C \in \mathbb{R}$ such that $f(v) = C$ for almost every v.

Proof. Let us define the function

$$h : t \mapsto \mathbb{E}[f(Z-t)] = \frac{1}{\sqrt{2\pi}}\int f(x) e^{-(x+t)^2/2} dx\,.$$

We have $h'(t) = \frac{-1}{\sqrt{2\pi}} \int f(x)(x+t)e^{-(x+t)^2/2}dx = -\mathbb{E}[Zf(Z-t)] = 0$ and therefore h is equal to some constant $C \in \mathbb{R}$. We are going to show that $f = C$ almost everywhere. Without loss of generality we can assume that $C = 0$, otherwise it suffices to consider the function $\tilde{f} = f - C$. Now we have for all $n \geq 0$, $t \in \mathbb{R}$

$$0 = h^{(n)}(t) = \frac{1}{\sqrt{2\pi}} \int f(x) \frac{\partial}{\partial t} e^{-(x+t)^2/2} dx = \frac{1}{\sqrt{2\pi}} \int f(x)(-1)^n H_n(x+t) e^{-(x+t)^2/2} dx \,,$$

where H_n is n^{th} Hermite polynomial, defined as $H_n(x) = (-1)^n e^{x^2/2} \frac{d^n}{dx^n} e^{-x^2/2}$. Therefore, for all $n \geq 0$,

$$\int f(x) H_n(x) e^{-x^2/2} dx = 0 \,,$$

which implies that $f = 0$ almost everywhere since the Hermite functions form an orthonormal basis of $L^2(\mathbb{R})$. □

We apply now Lemma 16 to (185) where the function f is given by $f(x) = P_{\text{out}}(y \,|\, \sigma x)$. We thus obtain that for almost every y, $P_{\text{out}}(y \,|\, \cdot)$ is almost everywhere equal to a constant. Under assumption (ii), we get that for all $y \in \mathbb{N}$, $P_{\text{out}}(y \,|\, \cdot)$ is almost everywhere equal to a constant: this contradicts the hypothesis that P_{out} is informative.

If now assumption (i) holds, then by (3) the density function $P_{\text{out}}(\cdot \,|\, x)$ is continuous on $\mathbb{R}$ for all $x \in \mathbb{R}$. Let us fix $y \in \mathbb{R}$. We are going to show that $P_{\text{out}}(y \,|\, \cdot)$ is almost everywhere equal to a constant C_y. Given what we just showed, we can construct a sequence $(y_n)_n \in \mathbb{R}^{\mathbb{N}}$ that converges to y such that for all $n \geq 0$, there exists $E_n \subset \mathbb{R}$ with full Lebesgue's measure and $C_n \in \mathbb{R}$ such that for all $x \in E_n$,

$$P_{\text{out}}(y_n|x) = C_n \,.$$

Let us define $E = \cap_{n \geq 0} E_n$. E has therefore full Lebesgue's measure. Let now $x_1, x_2 \in E$. By continuity of $P_{\text{out}}(\cdot|x_i)$, we get

$$P_{\text{out}}(y_n|x_i) \xrightarrow[n\to\infty]{} P_{\text{out}}(y|x_i), \qquad \text{for } i = 1, 2.$$

Since we know that for all $n \geq 0$ that $P_{\text{out}}(y_n|x_1) = C_n = P_{\text{out}}(y_n|x_2)$, we deduce that $P_{\text{out}}(y|x_1) = P_{\text{out}}(y|x_2)$. This proves that $P_{\text{out}}(y \,|\, \cdot)$ is almost everywhere equal to a constant C_y and contradicts the fact that P_{out} is informative. □

We turn now our attention to the study of the function:

$$\mathcal{E}_f : \left| \begin{array}{ccc} [0, \rho] & \to & \mathbb{R}_+ \\ q & \mapsto & \mathbb{E}\big[(f(Y^{(q)}) - \mathbb{E}[f(Y^{(q)})|V])^2\big] \end{array} \right. \tag{186}$$

where $f : \mathbb{R} \to \mathbb{R}$ is a continuous bounded function. We will prove that $\mathcal{E}_f$ is continuous (Proposition 22) and strictly decreasing (Proposition 23) under the following hypotheses.

(a) For all $x \in \mathbb{R}$, $P_{\text{out}}(\cdot \,|\, x)$ is the law of $\varphi(x, \mathbf{A}) + \sqrt{\Delta}\, Z$ where $\varphi : \mathbb{R} \times \mathbb{R}^{k_A} \to \mathbb{R}$ is a measurable function and $(Z, \mathbf{A}) \sim \mathcal{N}(0,1) \otimes P_A$, for some probability distribution P_A over $\mathbb{R}^{k_A}$.

(b) For almost all $a \in \mathbb{R}^{k_A}$ (w.r.t. P_A), $\varphi(\cdot, a)$ is continuous almost everywhere.

We suppose also that we are in one of the following cases:

(i) $\Delta > 0$.

(ii) $\Delta = 0$ and φ takes values in $\mathbb{N}$.

Proposition 22. *Under the hypotheses presented above, $\mathcal{E}_f$ is continuous on $[0, \rho]$.*

Proof. Consider expression (43): The first term does not depend on q and the second one is continuous by Lebesgue's convergence theorem. □

Proposition 23. *Assume that the hypotheses of Proposition 22 hold. Suppose that $x \mapsto \int f(y) P_{\text{out}}(y \mid x) dy$ is not almost-everywhere equal to a constant. Then $\mathcal{E}_f$ is strictly decreasing on $[0, \rho]$.*

Proof. $\mathcal{E}_f(q) = \mathbb{E}[f(Y^{(q)})^2] - \mathbb{E}\big[\mathbb{E}[f(Y^{(q)})|V]^2\big]$. Since the first term does not depend on q, it suffices to show that $H : q \mapsto \mathbb{E}\big[\mathbb{E}[f(Y^{(q)})|V]^2\big]$ is strictly increasing on $[0, \rho]$. We have for $q \in (0, \rho)$:

$$\mathbb{E}[f(Y^{(q)})|V] = \int\int f(y) \frac{e^{-w^2/2}}{\sqrt{2\pi}} P_{\text{out}}(y|\sqrt{q}\,V + \sqrt{\rho - q}\,w) dy dw = \int\int f(y) \frac{e^{-\frac{(x-\sqrt{q}V)^2}{2(\rho-q)}}}{\sqrt{2\pi(\rho - q)}} P_{\text{out}}(y|x) dy dx\,.$$

So we have, using (184):

$$\begin{aligned}
\frac{\partial}{\partial q}\mathbb{E}[f(Y^{(q)})|V] &= \int\int \frac{f(y)}{2}\Big(\frac{1}{\rho - q} - \frac{(x - \sqrt{q}\,V)^2}{(\rho - q)^2} + \frac{V(x - \sqrt{q}\,V)}{\sqrt{q}(\rho - q)}\Big) \frac{e^{-\frac{(x-\sqrt{q}\,V)^2}{2(\rho-q)}}}{\sqrt{2\pi(\rho - q)}} P_{\text{out}}(y|x) dy dw \\
&= \frac{1}{2(\rho - q)}\mathbb{E}\left[f(Y^{(q)})\Big(1 - W^{*2} + \frac{\sqrt{\rho - q}\,VW^*}{\sqrt{q}}\Big)\Big|V\right].
\end{aligned}$$

We obtain

$$H'(q) = \frac{1}{\rho - q}\mathbb{E}\left[\mathbb{E}[f(Y^{(q)})|V]\,\mathbb{E}\left[f(Y^{(q)})\Big(1 - W^{*2} + \frac{\sqrt{\rho - q}\,VW^*}{\sqrt{q}}\Big)\Big|V\right]\right]. \tag{187}$$

We compute by Gaussian integration by parts:

$$\begin{aligned}
&\mathbb{E}\left[\mathbb{E}[f(Y^{(q)})|V]\,\mathbb{E}\left[f(Y^{(q)})VW^*\Big|V\right]\right] = \mathbb{E}\left[V\mathbb{E}[f(Y^{(q)})|V]\,\mathbb{E}\left[f(Y^{(q)})W^*\Big|V\right]\right] \\
&= \mathbb{E}\left[\frac{\partial}{\partial V}\mathbb{E}[f(Y^{(q)})|V]\,\mathbb{E}\left[f(Y^{(q)})W^*\Big|V\right]\right] + \mathbb{E}\left[\mathbb{E}[f(Y^{(q)})|V]\,\frac{\partial}{\partial V}\mathbb{E}\left[f(Y^{(q)})W^*\Big|V\right]\right]. \qquad (188)
\end{aligned}$$

We compute successively

$$\begin{aligned}
\frac{\partial}{\partial V}\mathbb{E}[f(Y^{(q)})|V] &= \frac{\partial}{\partial V}\int\int f(y) \frac{e^{-\frac{(x-\sqrt{q}\,V)^2}{2(\rho-q)}}}{\sqrt{2\pi(\rho - q)}} P_{\text{out}}(y|x) dy dx \\
&= \int\int f(y) \frac{\sqrt{q}\,(x - \sqrt{q}\,V)}{\rho - q} \frac{e^{-\frac{(x-\sqrt{q}\,V)^2}{2(\rho-q)}}}{\sqrt{2\pi(\rho - q)}} P_{\text{out}}(y|x) dy dx = \frac{\sqrt{q}}{\sqrt{\rho - q}}\mathbb{E}\Big[f(Y^{(q)})W^*\Big|V\Big].
\end{aligned} \tag{189}$$

$$\begin{aligned}
\frac{\partial}{\partial V}\mathbb{E}[f(Y^{(q)})W^*|V] &= \frac{\partial}{\partial V}\int\int f(y) \frac{x - \sqrt{q}\,V}{\sqrt{\rho - q}} \frac{e^{-\frac{(x-\sqrt{q}\,V)^2}{2(\rho-q)}}}{\sqrt{2\pi(\rho - q)}} P_{\text{out}}(y|x) dy dx \\
&= \int\int f(y) \Big(\frac{-\sqrt{q}}{\sqrt{\rho - q}} + \frac{\sqrt{q}(x - \sqrt{q}\,V)^2}{(\rho - q)^{3/2}}\Big) \frac{e^{-\frac{(x-\sqrt{q}\,V)^2}{2(\rho-q)}}}{\sqrt{2\pi(\rho - q)}} P_{\text{out}}(y|x) dy dx \\
&= \frac{\sqrt{q}}{\sqrt{\rho - q}}\mathbb{E}\Big[f(Y^{(q)})\big(-1 + W^{*2}\big)\Big|V\Big].
\end{aligned} \tag{190}$$

By plugging (188)-(189)-(190) back in (187) we get:

$$H'(q) = \frac{1}{\rho - q}\mathbb{E}\Big[\mathbb{E}\big[f(Y^{(q)})W^*|V\big]^2\Big] \geq 0\,.$$

Let us suppose now that H is not strictly increasing on $[0,\rho]$. This means that we can find $q \in (0,\rho)$ such that $H'(q) = 0$ and therefore $\mathbb{E}[f(Y^{(q)})W^*|V] = 0$ almost-surely. This gives that for almost all $v \in \mathbb{R}$,

$$\mathbb{E}\Big[W \int f(y) P_{\rm out}(y|\sqrt{q}\, v + \sqrt{\rho - q}\, W) dy\Big] = 0\,,$$

where $\mathbb{E}$ is the expectation with respect to $W \sim \mathcal{N}(0,1)$. Lemma 16 gives then that the function $x \mapsto \int f(y) P_{\rm out}(y|x) dy$ is almost everywhere equal to a constant: we obtain a contradiction. We conclude that H is strictly increasing on $[0,\rho]$ and thus $\mathcal{E}_f$ is strictly decreasing on $[0,\rho]$. □

Proposition 24. *Assume that the hypotheses of Proposition 22 hold. If the channel $P_{\rm out}$ is informative, then there exists a continuous bounded function $f : \mathbb{R} \to \mathbb{R}$ such that $x \mapsto \int f(y) P_{\rm out}(y|x) dy$ is not almost everywhere equal to a constant.*

Proof. Let us suppose that for all continuous bounded function $f : \mathbb{R} \to \mathbb{R}$ we have

$$\int f(y) P_{\rm out}(y|x) dy = C_f$$

for almost all $x \in \mathbb{R}$, for some constant $C_f \in \mathbb{R}$. Let $X \sim \mathbb{N}(0,1)$ and $Y \sim P_{\rm out}(\cdot|X)$. We have then $\mathbb{E}[f(Y)|X] = C_f = \mathbb{E}[f(Y)]$ almost surely. Let $g : \mathbb{R} \to \mathbb{R}$ be another continuous bounded function and compute:

$$\mathbb{E}[g(X)f(Y)] = \mathbb{E}\big[g(X)\mathbb{E}[f(Y)|X]\big] = \mathbb{E}[g(X)]\mathbb{E}[f(Y)]\,.$$

It follows that X and Y are independent: The measures $P_{\rm out}(y|x)\frac{e^{-x^2/2}}{\sqrt{2\pi}}dydx$ and $\mathbb{E}[P_{\rm out}(y|X)]\frac{e^{-x^2/2}}{\sqrt{2\pi}}dydx$ are therefore equal. Consequently, for almost every x, y we have

$$P_{\rm out}(y|x) = \mathbb{E}[P_{\rm out}(y|X)]\,.$$

This gives that for almost every y, $P_{\rm out}(y|\cdot)$ is almost everywhere equal to a constant. We conclude by the arguments presented at the end of the proof of Proposition 21 that $P_{\rm out}$ is not informative, which is a contradiction. □

Appendix C: Approximation

Let us recall the various hypotheses considered in this paper, starting with the stronger set:

(H1) The prior distribution P_0 has a bounded support.
(H2) φ is a bounded $\mathcal{C}^2$ function with bounded first and second derivatives w.r.t. its first argument.
(H3) $(\Phi_{\mu i}) \overset{\rm iid}{\sim} \mathcal{N}(0,1)$.

The aim of this section is to relax them to the weaker ones:

(h1) The prior distribution P_0 admits a finite third moment and has at least two points in its support.
(h2) There exists $\gamma > 0$ such that the sequence $(\mathbb{E}[|\varphi(\frac{1}{\sqrt{n}}[\mathbf{\Phi X}^*]_1, \mathbf{A}_1)|^{2+\gamma}])_{n\geq 1}$ is bounded.
(h3) The random variables $(\Phi_{\mu i})$ are independent with zero mean, unit variance and finite third moment that is bounded with n.
(h4) For almost-all values of $\mathbf{a} \in \mathbb{R}^{k_A}$ (w.r.t. P_A), the function $x \mapsto \varphi(x, \mathbf{a})$ is continuous almost everywhere.

The hypotheses on the precence or not of the Gaussian noise in (1) are:

(h5.a) $\Delta > 0$.

(h5.b) $\Delta = 0$ and φ takes values in $\mathbb{N}$.

In this section, we suppose that Theorem 1 holds for channels of the form (1) (with $\Delta > 0$) under the hypotheses (H1), (H2) and (H3), as proven in Section 4.

We show in this section that this imply that Theorem 1 holds under the weaker hypotheses (h1)-(h2)-(h3)-(h4), and either (h5.a) or (h5.b). This section is organized as follows: We first prove Theorem 1 under (h1)-(h2)-(h3)-(h4) and (h5.a) (i.e. $\Delta > 0$). This is done by first relaxing the hypotheses on P_0 and $\mathbf{\Phi}$ (Sec. C.1) and then the hypotheses on φ (Sec. C.2). Finally, in Sec. C.3, we let $\Delta \to 0$ in order to prove Theorem 1 under (h1)-(h2)-(h3)-(h4) and (h5.b).

Note that the statement of Theorem 1 is equivalent to the statement of Corollary 1, which simply express the result in terms of mutual information. This formulation will be slightly more convenient to relax the hypotheses. We will therefore prove in this section that (27) holds under the hypotheses (h1)-(h2)-(h3)-(h4), and either (h5.a) or (h5.b). The statement of Theorem 1 can then be directly obtained by using the expressions of I_{P_0}, $\mathcal{I}_{P_{\rm out}}$ in terms of ψ_{P_0}, $\Psi_{P_{\rm out}}$, the relation (30) and Lemma 23.

C.1 Relaxing the hypotheses on P_0 and $\mathbf{\Phi}$

As explained at the beginning of Sec. 4, it suffices to consider the case $\Delta = 1$. We start by relaxing the hypothesis (H1).

Lemma 17 (Relaxing P_0)**.** *Suppose that (h1)-(H2)-(H3) and (h5.a) hold. Then Theorem 1 holds.*

Proof. The ideas are basically the same that in [59] (Sec. 6.2.2). We omit the details here for the sake of brevity. □

We now relax the Gaussian assumption on the "measurement matrix" $\mathbf{\Phi}$.

Lemma 18 (Relaxing $\mathbf{\Phi}$)**.** *Suppose that $\varphi : \mathbb{R} \times \mathbb{R}^{k_A} \to \mathbb{R}$ is $\mathcal{C}^\infty$ with compact support and that (h1)-(h3)-(h5.a) hold. Then Theorem 1 holds.*

Proof. The proof is based on the Lindeberg generalization theorem (Theorem 2 from [54]) which is a variant of the generalized "Lindeberg principle" from [60]:

Theorem 5 (Lindeberg generalization theorem)**.** *Let $(U_i)_{1\leq i \leq n}$ and $(V_i)_{1\leq i\leq n}$ be two collections of random variables with independent components and $f : \mathbb{R}^n \to \mathbb{R}$ a $\mathcal{C}^3$ function. Denote $a_i = |\mathbb{E}U_i - \mathbb{E}V_i|$ and $b_i = |\mathbb{E}[U_i^2] - \mathbb{E}[V_i^2]|$. Then*

$$|\mathbb{E}f(\mathbf{U}) - \mathbb{E}f(\mathbf{V})| \leq \sum_{i=1}^n \Big\{ a_i \mathbb{E}|\partial_i f(U_{1:i-1}, 0, V_{i+1:n})| + \frac{b_i}{2}\mathbb{E}|\partial_i^2 f(U_{1:i-1}, 0, V_{i+1:n})|$$
$$+ \frac{1}{2}\mathbb{E}\int_0^{U_i} |\partial_i^3 f(U_{1:i-1}, 0, V_{i+1:n})|(U_i - s)^2 ds + \frac{1}{2}\mathbb{E}\int_0^{V_i} |\partial_i^3 f(U_{1:i-1}, 0, V_{i+1:n})|(V_i - s)^2 ds \Big\}.$$

Let $(\Phi'_{\mu,i}) \overset{\rm iid}{\sim} \mathcal{N}(0,1)$ and let $(\Phi_{\mu,i})$ be a family of independent random variables, with zero mean and unit variance. Let f'_n be the free entropy (10) with design matrix $\mathbf{\Phi}'$ and f_n be the free entropy (10) with design matrix $\mathbf{\Phi}$.

We will apply Theorem 5 to the function

$$F : \mathbf{U} \in \mathbb{R}^{m\times n} \mapsto \frac{1}{n}\mathbb{E}\ln \int_{\mathbf{x},\mathbf{a}} dP_A(\mathbf{a})dP_0(\mathbf{x})\, e^{-\frac{1}{2}\sum\limits_{\mu=1}^m \Big(\varphi\big(\frac{1}{\sqrt{n}}[\mathbf{U}\mathbf{X}^*]_\mu, \mathbf{A}_\mu\big) - \varphi\big(\frac{1}{\sqrt{n}}[\mathbf{U}\mathbf{x}]_\mu, \mathbf{a}_\mu\big) + Z_\mu\Big)^2}$$

where the expectation $\mathbb{E}$ is taken w.r.t. $\mathbf{X}^*$, $\mathbf{A}$ and $\mathbf{Z}$. We have

$$f_n = \mathbb{E}F(\mathbf{\Phi}) \qquad \text{and} \qquad f_n' = \mathbb{E}F(\mathbf{\Phi}') \, .$$

It is not difficult to verify that F is a $\mathcal{C}^3$ function and that for all $1 \leq \mu \leq m$ and $1 \leq i \leq n$:

$$\left\| \frac{\partial^3 F}{\partial U_{\mu,i}^3} \right\|_\infty \leq \frac{C}{n^{5/2}} \, ,$$

for some constant C that only depends on φ and the first three moments of P_0. Thus, an application of Theorem 5 gives $|f_n - f_n'| \leq \frac{C}{\sqrt{n}}$. By Proposition 17, we know that Theorem 1 holds for f_n', thus it holds for f_n. □

C.2 Relaxing the hypotheses on φ

It remains to relax the hypotheses on φ. This section is dedicated to the proof of the following proposition, which is of course exactly the statement of Theorem 1.

Proposition 25 (Relaxing φ)**.** *Suppose that (h1)-(h2)-(h3)-(h4) and (h5.a) hold. Then, Theorem 1 holds for the output channel* (1).

To prove Proposition 25 we will approximate the function φ with a function $\widehat{\varphi}$ which is $\mathcal{C}^\infty$ with compact support. In the following, G is a standard Gaussian random variable, independent of everything else.

Proposition 26. *Suppose that (h1)-(h2)-(h3)-(h4) hold. Then, for all $\epsilon > 0$, there exist $\widehat{\varphi} \in \mathcal{C}^\infty(\mathbb{R} \times \mathbb{R}^{k_A})$ with compact support, such that*

$$\mathbb{E}\left[(\varphi(\sqrt{\rho}G, \boldsymbol{A}) - \widehat{\varphi}(\sqrt{\rho}G, \boldsymbol{A}))^2\right] \leq \epsilon \, ,$$

and for n large enough, we have

$$\mathbb{E}\left[\left(\varphi\left(\frac{1}{\sqrt{n}}[\mathbf{\Phi}\boldsymbol{X}^*]_1, \boldsymbol{A}_1\right) - \widehat{\varphi}\left(\frac{1}{\sqrt{n}}[\mathbf{\Phi}\boldsymbol{X}^*]_1, \boldsymbol{A}_1\right)\right)^2\right] \leq \epsilon \, .$$

Proof. By the Central Limit Theorem (using the fact that the third moments of $(X_i^* \Phi_{1,i})$ are bounded with n, because of hypotheses (h1) and (h3))

$$\left(\frac{[\mathbf{\Phi}\mathbf{X}^*]_1}{\sqrt{n}}, \mathbf{A}_1\right) \xrightarrow[n\to\infty]{(d)} (\sqrt{\rho}G, \mathbf{A}_1) \, . \tag{191}$$

This implies that

$$\varphi\left(\frac{[\mathbf{\Phi}\mathbf{X}^*]_1}{\sqrt{n}}, \mathbf{A}_1\right) \xrightarrow[n\to\infty]{(d)} \varphi(\sqrt{\rho}G, \mathbf{A}_1) \, , \tag{192}$$

because $\varphi(\cdot, \mathbf{A}_1)$ is almost-surely continuous almost-everywhere, by assumption (h4). The following sequence $(\varphi(\frac{[\mathbf{\Phi}\mathbf{X}^*]_1}{\sqrt{n}}, \mathbf{A}_1))_n$ is by assumption (h2) bounded in L^2, thus by (192) we have that $\mathbb{E}[\varphi(\sqrt{\rho}G, \mathbf{A}_1)^2] < \infty$. Let $\epsilon > 0$. We have just proved that $\varphi \in L^2(\mathbb{R} \times \mathbb{R}^{k_A})$ with the measure induced by $(\sqrt{\rho}G, \mathbf{A}_1)$. There exists (see for instance Corollary 4.2.2 in [58]) a $\mathcal{C}^\infty$ function with compact support $\widehat{\varphi}$ such that $\mathbb{E}\left[(\varphi(\sqrt{\rho}G, \mathbf{A}) - \widehat{\varphi}(\sqrt{\rho}G, \mathbf{A}))^2\right] \leq \epsilon$.

One deduce from (191) and (192) that

$$\left(\varphi\left(\frac{1}{\sqrt{n}}[\mathbf{\Phi}\mathbf{X}^*]_1, \mathbf{A}_1\right) - \widehat{\varphi}\left(\frac{1}{\sqrt{n}}[\mathbf{\Phi}\mathbf{X}^*]_1, \mathbf{A}_1\right)\right)^2 \xrightarrow[n\to\infty]{(d)} (\varphi(\sqrt{\rho}G, \mathbf{A}) - \widehat{\varphi}(\sqrt{\rho}G, \mathbf{A}))^2 \, .$$

Now, hypothesis (h2) gives that the sequence above is uniformly integrable. This gives that

$$\mathbb{E}\left(\varphi\left(\frac{1}{\sqrt{n}}[\mathbf{\Phi}\mathbf{X}^*]_1, \mathbf{A}_1\right) - \widehat{\varphi}\left(\frac{1}{\sqrt{n}}[\mathbf{\Phi}\mathbf{X}^*]_1, \mathbf{A}_1\right)\right)^2 \xrightarrow[n\to\infty]{} \mathbb{E}(\varphi(\sqrt{\rho}G, \mathbf{A}) - \widehat{\varphi}(\sqrt{\rho}G, \mathbf{A}))^2 \leq \epsilon\,.$$

Consequently, the left-hand side is smaller that 2ϵ for n large enough. This concludes the proof. □

In the remaining of this section, we prove Proposition 25. Let $\epsilon > 0$. Let φ and $\widehat{\varphi}$ as in Proposition 26. Let us define $\mathbf{Y} = \varphi(n^{-1/2}\mathbf{\Phi}\mathbf{X}^*, \mathbf{A}) + \sqrt{\Delta}Z$ and $\widehat{\mathbf{Y}} = \widehat{\varphi}(n^{-1/2}\mathbf{\Phi}\mathbf{X}^*, \mathbf{A}) + \sqrt{\Delta}Z$.

Lemma 19. *Suppose that (h1)-(h2)-(h3)-(h4) and (h5.a) hold. There exists a constant $C > 0$ such that for n large enough*

$$\left|\frac{1}{n}I(\mathbf{X}^*;\mathbf{Y}|\mathbf{\Phi}) - \frac{1}{n}I(\mathbf{X}^*;\widehat{\mathbf{Y}}|\mathbf{\Phi})\right| \leq C\sqrt{\epsilon}\,.$$

Proof. We have, for n large enough

$$\mathbb{E}\|\mathbf{Y} - \widehat{\mathbf{Y}}\|^2 = m\mathbb{E}\left[\left(\varphi\left(\frac{1}{\sqrt{n}}[\mathbf{\Phi}\mathbf{X}^*]_1, \mathbf{A}_1\right) - \widehat{\varphi}\left(\frac{1}{\sqrt{n}}[\mathbf{\Phi}\mathbf{X}^*]_1, \mathbf{A}_1\right)\right)^2\right] \leq m\epsilon\,.$$

By Proposition 14, we obtain that there exists a constant $C > 0$ (that depends only on Δ and φ) such that

$$\left|I(\mathbf{X}^*;\mathbf{Y}|\mathbf{\Phi}) - I(\mathbf{X}^*;\widehat{\mathbf{Y}}|\mathbf{\Phi})\right| \leq Cm\sqrt{\epsilon}\,,$$

which gives the result. □

Let $P_{\rm out}$ denote the transition kernel associated to φ and $\widehat{P}_{\rm out}$ the one associated to $\widehat{\varphi}$. Analogously to the previous Lemma, one can show:

Lemma 20. *There exists a constant $C' > 0$ such that for all $q \in [0,\rho]$, $|\mathcal{I}_{P_{\rm out}}(q) - \mathcal{I}_{\widehat{P}_{\rm out}}(q)| \leq C'\sqrt{\epsilon}$.*

From there we obtain that

$$\left|\inf_{q\in[0,\rho]}\sup_{r\geq 0} i_{\rm RS}(q,r) - \inf_{q\in[0,\rho]}\sup_{r\geq 0} \widehat{i}_{\rm RS}(q,r)\right| \leq C'\sqrt{\epsilon}\,. \tag{193}$$

Applying Theorem 1 for $\widehat{P}_{\rm out}$, we obtain that for n large enough $|\frac{1}{n}I(\mathbf{X}^*;\widehat{\mathbf{Y}}|\mathbf{\Phi}) - \inf_{q\in[0,\rho]}\sup_{r\geq 0}\widehat{i}_{\rm RS}(q,r)| \leq \sqrt{\epsilon}$. We now combine this with (193) and Lemma 19 we obtain that for n large enough

$$\left|\frac{1}{n}I(\mathbf{X}^*;\mathbf{Y}|\mathbf{\Phi}) - \inf_{q\in[0,\rho]}\sup_{r\geq 0} i_{\rm RS}(q,r)\right| \leq \left|\frac{1}{n}I(\mathbf{X}^*;\widehat{\mathbf{Y}}|\mathbf{\Phi}) - \inf_{q\in[0,\rho]}\sup_{r\geq 0}\widehat{i}_{\rm RS}(q,r)\right| + (C+C')\sqrt{\epsilon} \leq (C+C'+1)\sqrt{\epsilon}\,,$$

which concludes the proof of Proposition 25, because of (30) and the definition of the functions I_{P_0} and $\mathcal{I}_{P_{\rm out}}$ in Corollary 1.

C.3 The case of discrete channels: Removing the Gaussian noise

Now that we proved (Proposition 25) that Theorem 1 holds under hypotheses (h1)-(h2)-(h3)-(h4) and (h5.a), we are going to show that it holds under (h1)-(h2)-(h3)-(h4) and (h5.b) by letting $\Delta \to 0$. We suppose in this section that φ takes values in $\mathbb{N}$ and write $\mathbf{Y} = \varphi\big(\mathbf{\Phi}\mathbf{X}^*/\sqrt{n}, \mathbf{A}\big)$. By Proposition 25 we know that for all $\Delta > 0$,

$$\frac{1}{n}I(\mathbf{X}^*;\mathbf{Y}+\sqrt{\Delta}\mathbf{Z}|\mathbf{\Phi}) \xrightarrow[n\to\infty]{} \inf_{q\in[0,\rho]}\sup_{r\geq 0}\left\{I_{P_0}(r) + \alpha I(W^*;\varphi(\sqrt{q}V+\sqrt{\rho-q}W^*, A)+\sqrt{\Delta}Z|V) - \frac{r}{2}(\rho-q)\right\},$$

where $\mathbf{Z} \sim \mathcal{N}(0, \mathbf{I}_m)$ and $(V, W^*, Z, A) \sim \mathcal{N}(0,1)^{\otimes 3} \otimes P_A$. Since $\mathbf{Y}$ takes values in $\mathbb{N}^m$ and φ takes values in $\mathbb{N}$, we can apply Corollary 6 twice to obtain that for all $\Delta \in (0,1]$,

$$\Big| I(\mathbf{X}^*; \mathbf{Y} + \sqrt{\Delta}\mathbf{Z} | \mathbf{\Phi}) - I(\mathbf{X}^*; \mathbf{Y} | \mathbf{\Phi}) \Big| \leq 100 m e^{-1/(16\Delta)}$$

and (recall that by definition $\mathcal{I}_{P_{\rm out}}(q) = I\big(W^*; \varphi(\sqrt{q}V + \sqrt{\rho - q}W^*, A) \big| V\big)$):

$$|I\big(W^*; \varphi(\sqrt{q}V + \sqrt{\rho - q}W^*, A) + \sqrt{\Delta}Z \big| V\big) - \mathcal{I}_{P_{\rm out}}(q)| \leq 100 e^{-1/(16\Delta)} \,.$$

Since our control over Δ is uniform in n, we can permute the $n \to \infty$ limit with the $\Delta \to 0$ limit to get:

$$\frac{1}{n} I(\mathbf{X}^*; \mathbf{Y} | \mathbf{\Phi}) \xrightarrow[n\to\infty]{} \inf_{q \in [0,\rho]} \sup_{r \geq 0} \Big\{ I_{P_0}(r) + \alpha \mathcal{I}_{P_{\rm out}}(q) - \frac{r}{2}(\rho - q) \Big\} \,.$$

Appendix D: Some sup-inf formulas

This appendix gathers some useful lemmas for the manipulations of "sup-inf" formulas like (24).

Lemma 21. *Let $f, g : \mathbb{R}_+ \to \mathbb{R}$ be two non-decreasing convex functions. We have*

$$\sup_{x \geq 0} \inf_{y \geq 0} \big\{ f(x) + g(y) - xy \big\} = \sup_{y \geq 0} \inf_{x \geq 0} \big\{ f(x) + g(y) - xy \big\} \,.$$

Proof. Let us define the monotone conjugate (see the end of §12 of [61]) of f and g:

$$f^*(y) = \sup_{x \geq 0} \big\{ xy - f(x) \big\} \quad \text{and} \quad g^*(y) = \sup_{x \geq 0} \big\{ xy - g(x) \big\} \,.$$

These conjugates satisfy an analog of the Fenchel-Moreau Theorem: $f(x) = \sup_{y \geq 0}\{xy - f^*(y)\}$ and $g(y) = \sup_{x \geq 0}\{xy - g^*(x)\}$, see Theorem 12.4 from [61]. We have then

$$\begin{aligned}
\sup_{x \geq 0} \inf_{y \geq 0} \big\{ f(x) + g(y) - xy \big\} &= \sup_{x \geq 0} \big\{ f(x) - g^*(x) \big\} = \sup_{x \geq 0} \sup_{y \geq 0} \big\{ xy - f^*(y) - g^*(x) \big\} \\
&= \sup_{y \geq 0} \Big\{ -f^*(y) + \sup_{x \geq 0} \big\{ xy - g^*(x) \big\} \Big\} = \sup_{y \geq 0} \big\{ -f^*(y) + g(y) \big\} \\
&= \sup_{y \geq 0} \inf_{x \geq 0} \big\{ f(x) + g(y) - xy \big\}.
\end{aligned}$$

□

The next Lemma on the Legendre transform will be useful.

Lemma 22. *Let $V \subset \mathbb{R}$ be a non-empty, closed interval and let $g : V \to \mathbb{R}$ be a continuous convex function. Define*

$$g^* : x \in \mathbb{R} \mapsto \sup_{y \in V} \big\{ xy - g(y) \big\} \in \mathbb{R} \cup \{+\infty\} \,. \tag{194}$$

Let $\operatorname{dom} g^* = \{x \in \mathbb{R} \,|\, g^*(x) < \infty\}$. *Then g^* is a closed convex function and* $\operatorname{dom} g^*$ *is a non-empty interval. Moreover, for all $x \in \operatorname{dom} g^*$,*

$$\partial g^*(x) = \arg\max_{y \in V} \big\{ xy - g(y) \big\} \,. \tag{195}$$

In particular, if g is strictly convex then g^* is differentiable around every point in the interior of $\operatorname{dom} g^*$.

Proof. We first extend the function g on $\mathbb{R}$ by setting $g(x) = +\infty$ for all $x \notin V$. Notice that this does not change the definition of the function g^*. g is then a proper, closed convex function (see for instance [61] for

the definitions of these properties). By Theorem 12.2 in [61], g^* is also a proper closed convex function on $\mathbb{R}$, which gives that $\operatorname{dom} g^*$ is a non-empty interval. We now apply Corollary 23.5.1 from [61] to obtain

$$y \in \partial g^*(x) \iff x \in \partial g(y) \iff y \text{ maximizes } \big\{xy - g(y)\big\}\,,$$

for all $x \in \operatorname{dom} g^*$, which concludes the proof. □

Corollary 7. *Let $f : \mathbb{R}_+ \to \mathbb{R}$ be a convex, Lipschitz, non-decreasing function. Define $\rho = \sup_{x\geq 0} f'(x^+)$. Let $g : [0,\rho] \to \mathbb{R}$ be a convex, Lipschitz, non-decreasing function. For $q_1 \in \mathbb{R}_+$ and $q_2 \in [0,\rho]$ we define $\psi(q_1,q_2) = f(q_1) + g(q_2) - q_1q_2$. Then*

$$\sup_{q_1\geq 0}\inf_{q_2\in[0,\rho]} \psi(q_1,q_2) = \sup_{q_2\in[0,\rho]}\inf_{q_1\geq 0} \psi(q_1,q_2)\,.$$

Proof. In order to apply Lemma 21 we need to extend g on $\mathbb{R}_+$. We thus define for $x \geq 0$

$$g(x) = \begin{cases} g(x) & \text{if } x \leq \rho\,,\\ g(\rho) + (x-\rho)g'(\rho^-) & \text{if } x \geq \rho\,.\end{cases}$$

Obviously g is a convex, Lipschitz, non-decreasing function on $\mathbb{R}_+$. One can thus apply Lemma 21:

$$\sup_{q_1\geq 0}\inf_{q_2\geq 0}\big\{f(q_1)+g(q_2)-q_1q_2\big\} = \sup_{q_2\geq 0}\inf_{q_1\geq 0}\big\{f(q_1)+g(q_2)-q_1q_2\big\}\,. \tag{196}$$

We will show now that $\sup_{q_1\geq 0}\inf_{q_2\geq 0}\psi(q_1,q_2) = \sup_{q_1\geq 0}\inf_{q_2\in[0,\rho]}\psi(q_1,q_2)$. Let us define for $q_1 \geq 0$

$$g^*(q_1) = \sup_{q_2\in[0,\rho]}\{q_1q_2 - g(q_2)\} \quad\text{and}\quad h(q_1) = \inf_{q_2\in[0,\rho]}\big\{f(q_1)+g(q_2)-q_1q_2\big\} = f(q_1) - g^*(q_1)\,.$$

For $q_1 \geq g'(\rho^-)$ we have $g^*(q_1) = q_1\rho - g(\rho)$. The function h is therefore non-increasing on $[g'(\rho^-), +\infty)$, because f is ρ-Lipschitz. We get that $h = f - g^*$ achieves its supremum on $[0, g'(\rho^-)]$. Let q_1^* be the smallest point at which this supremum is achieved. Let us show that $\inf_{q_2\in[0,\rho]}\{g(q_2)-q_1^*q_2\} = \inf_{q_2\geq 0}\{g(q_2)-q_1^*q_2\}$.

- If $q_1^* = 0$, then the minimum over $[0,\rho]$ is achieved at $q_2 = 0$, because g is non-decreasing. By convexity, q_2 is also the minimizer over $\mathbb{R}_+$: both infimum are equal.
- If $q_1^* > 0$, the optimality condition of q_1^* gives $f'(q_1^{*-}) - (g^*)'(q_1^{*-}) \geq 0$. By (195) we obtain that there exists $q_2^* \in \arg\min_{q_2\in[0,\rho]}\{g(q_2)-q_1^*q_2\}$ such that $f'(q_1^{*-}) \geq q_2^*$. If $q_2^* < \rho$ we conclude, as above, that both infimum are equal. Suppose now that $q_2^* = \rho$ and define $q_1' = g'(\rho^-)$. By the optimality condition of $q_2^* = \rho$ we have $q_1' = g'(\rho^-) \leq q_1^*$. Compute

  $$h(q_1^*) - h(q_1') = f(q_1^*) - f(q_1') - \rho(q_1^* - q_1') \leq 0$$

  because f is ρ-Lipschitz. Since $q_1' \leq q_1^*$ and q_1^* is defined as the smallest maximizer of h, we get that $q_1^* = q_1'$. The left-hand derivative of $q_2 \mapsto g(q_2) - q_1^*q_2$ at $q = \rho$ is therefore equal to 0: ρ minimizes $q_2 \mapsto g(q_2) - q_1^*q_2$ over $\mathbb{R}_+$: both infimum are equal.

We have proved that $\inf_{q_2\in[0,\rho]}\{g(q_2)-q_1^*q_2\} = \inf_{q_2\geq 0}\{g(q_2)-q_1^*q_2\}$. Therefore

$$\sup_{q_1\geq 0}\inf_{q_2\in[0,\rho]}\psi(q_1,q_2) = \inf_{q_2\in[0,\rho]}\psi(q_1^*,q_2) = \inf_{q_2\geq 0}\psi(q_1^*,q_2) \leq \sup_{q_1\geq 0}\inf_{q_2\geq 0}\psi(q_1,q_2)\,.$$

We conclude that $\sup_{q_1\geq 0}\inf_{q_2\geq 0}\psi(q_1,q_2) = \sup_{q_1\geq 0}\inf_{q_2\in[0,\rho]}\psi(q_1,q_2)$ because the converse inequality is trivial. It remains to show now that $\sup_{q_2\geq 0}\inf_{q_1\geq 0}\psi(q_1,q_2) = \sup_{q_2\in[0,\rho]}\inf_{q_1\geq 0}\psi(q_1,q_2)$ to prove the

Lemma, because of (196). The inequality "$\geq$" is obvious and the inequality "$\leq$" follows from the fact that $\inf_{q_1 \geq 0} \psi(q_1, q_2) = -\infty$ if $q_2 > \rho$. $\square$

Lemma 23. *Let g be a strictly convex, differentiable, Lipschitz non-decreasing function on $\mathbb{R}_+$. Define $\rho = \sup_{x \geq 0} g'(x)$. Let f be a convex, continuous, strictly increasing function on $[0, \rho]$, differentiable on $[0, \rho)$. For $(q_1, q_2) \in [0, \rho] \times \mathbb{R}_+$ we define $\psi(q_1, q_2) = f(q_1) + g(q_2) - q_1 q_2$. Then*

$$\sup_{q_1 \in [0,\rho]} \inf_{q_2 \geq 0} \psi(q_1, q_2) = \sup_{(q_1,q_2) \in \Gamma} \psi(q_1, q_2)\,, \tag{197}$$

where

$$\Gamma = \left\{ (q_1, q_2) \in [0, \rho] \times (\mathbb{R}_+ \cup \{+\infty\}) \;\middle|\; \begin{array}{rcl} q_1 & = & g'(q_2) \\ q_2 & = & f'(q_1) \end{array} \right\},$$

where all the function are extended by there limits at the points at which they may not be defined (for instance $g'(+\infty) = \lim\limits_{q \to \infty} g'(q)$, $f'(\rho) = \lim\limits_{q \to \rho} f'(q)$). Moreover, the above extremas are achieved precisely on the same couples.

Proof. Let q_1^* be a maximizer of $f - g^*$ over $[0, \rho]$. q_1^* is well defined because f is continuous and g^* is continuous over $[0, \rho)$ and is either continuous at ρ or goes to $+\infty$ at ρ (this comes from the fact that g^* is a closed convex function, see Lemma 22).

Case 1: $0 < q_1^* < \rho$. By strict convexity of g, $\psi(q_1, \cdot)$ admits a unique minimizer q_2^* and $(g^*)'(q_1^*) = q_2^*$ by Lemma 22. Thus, the optimality condition at q_1^* gives

$$0 = f'(q_1^*) - (g^*)'(q_1^*) = f'(q_1^*) - q_2^*\,.$$

The optimality of q_2^* gives then $q_1^* \leq g'(q_2^*)$. Suppose that $q_1^* < g'(q_2^*)$. This is only possible when $q_2^* = 0$. Define $q_1' = g'(q_2^*) = g'(0)$. Remark that $g^*(q_1') = -g(0) = g^*(q_1^*)$. We supposed that $q_1' > q_1^*$ thus, by strict monotonicity of f, $f(q_1') - g^*(q_1') > f(q_1^*) - g^*(q_1^*)$ which contradict the optimality of q_1^*. We obtain therefore that $q_1^* = g'(q_2^*)$.

Case 2: $q_1^* = 0$. The optimality condition gives now

$$0 \leq f'(q_1^* = 0) \leq q_2^*\,, \tag{198}$$

where q_2^* is again the unique minimizer of $\psi(q_1^* = 0, \cdot) = f(0) + g$. g is strictly increasing, so $q_2^* = 0$. Therefore $q_2^* = 0 = f'(q_1^* = 0)$, by (198). As before we have necessarily, by optimality of q_2^* that $q_1^* = g'(q_2^*)$.

Case 3: $q_1^* = \rho$. In that case $\arg\min_{q_2 \geq 0} \{g(q_2) - q_1^* q_2\} = \emptyset$ because g is strictly convex and ρ-Lipschitz. Lemma 22 gives then that $\partial g^*(\rho) = \emptyset$ which implies (see Theorem 23.3 from [61]) that $(g^*)'(\rho^-) = +\infty$. Since $q_1^* = \rho$ maximizes $f - g^*$, we necessarily have then $f'(\rho^-) = +\infty$.

Using the slight abuse of notation explained in the Lemma, we have $f'(q_1^*) = +\infty = q_2^*$, where $q_2^* = +\infty$ is the unique "minimizer" of $\psi(q_1^*, \cdot)$, by strict convexity of g. By definition of ρ we have also $g'(q_2^*) = g'(+\infty) = \rho = q_1^*$.

We conclude from the tree cases above that the "sup-inf" in (197) is achieved, and that all the couples (q_1^*, q_2^*) that achieve this "sup-inf" belong to Γ. Thus

$$\sup_{q_1 \in [0,\rho]} \inf_{q_2 \geq 0} \psi(q_1, q_2) \leq \sup_{(q_1,q_2) \in \Gamma} \psi(q_1, q_2)\,.$$

Let now be $(q_1, q_2) \in \Gamma$. By convexity of g we see easily that $\psi(q_1, q_2) = \inf_{q_2'} \psi(q_1, q_2')$. Thus, $\psi(q_1, q_2) \leq \sup_{q_1'} \inf_{q_2'} \psi(q_1', q_2')$. Therefore

$$\sup_{(q_1,q_2)\in\Gamma} \psi(q_1, q_2) \leq \sup_{q_1\in[0,\rho]} \inf_{q_2\geq 0} \psi(q_1, q_2)\,.$$

This concludes the proof of (197). It remains to see that a couple $(q_1^*, q_2^*) \in \Gamma$ that achieves the supremum in (197) also achieves the "sup-inf". This simply follows from the fact that $\psi(q_1^*, q_2^*) = \inf_{q_2} \psi(q_1^*, q_2)$ and (197). $\square$

Appendix E: Concentration of free entropy and overlaps

E.1 Concentration of the free entropy

The goal of this appendix is to prove that the free entropy of the interpolating model studied in Sec. 4.1 concentrates around its expectation. To simplify the notations we use $C(\varphi, S, \alpha)$ for a generic non-negative constant depending *only* on φ, S and α (S is the supremum over the signal values). We will also use the notation $K = 1 + \max(\rho, r_{\max})$ for a constant (depending only on $\rho \leq S^2$ and φ) that upper bounds both R_1 and R_2 given by (70). It is also understood that n and m are large enough and $m/n \to \alpha$.

Theorem 6 (Free entropy concentration). *Under assumptions (H1), (H2) and (H3) there exists a non-negative constant $C(\varphi, S, \alpha)$ such that the partition function* (78) *concentrates as*

$$\mathrm{Var}\Big(\frac{1}{n}\ln \mathcal{Z}_{t,\epsilon}\Big) = \mathbb{E}\Big[\Big(\frac{1}{n}\ln \mathcal{Z}_{t,\epsilon} - \frac{1}{n}\mathbb{E}\ln \mathcal{Z}_{t,\epsilon}\Big)^2\Big] \leq \frac{C(\varphi, S, \alpha)}{n}\,. \tag{199}$$

The remaining of this appendix is dedicated to the proof of Theorem 6. We first recall some set-up and notation for the convenience of the reader. Recall that the interpolating Hamiltonian (76)–(74) is

$$\mathcal{H}_{t,\epsilon}(\mathbf{x}, \mathbf{w}; \mathbf{Y}_t, \mathbf{Y}_t', \mathbf{\Phi}, \mathbf{V}) = -\sum_{\mu=1}^{m} \ln P_{\mathrm{out}}(Y_{t,\mu}|s_{t,\mu}(\mathbf{x}, w_\mu)) + \frac{1}{2}\sum_{i=1}^{n}(Y_{t,i}' - \sqrt{R_1(t)}\,x_i)^2 \tag{200}$$

where

$$s_{t,\mu}(\mathbf{x}, w_\mu) := \sqrt{\frac{1-t}{n}}[\mathbf{\Phi}\mathbf{x}]_\mu + k_1(t)V_\mu + k_2(t)w_\mu\,, \quad k_1(t) := \sqrt{R_2(t)}\,, \quad k_2(t) := \sqrt{\rho t - R_2(t) + 2s_n}\,.$$

We find it convenient to use the random function representation (1) for the interpolating model, namely

$$\begin{cases} Y_{t,\mu} = \varphi\Big(\sqrt{\frac{1-t}{n}}[\mathbf{\Phi}\mathbf{X}^*]_\mu + k_1(t)V_\mu + k_2(t)W_\mu^*, \mathbf{A}_\mu\Big) + Z_\mu\,, \\ Y_{t,i}' = \sqrt{R_1(t)}\,X_i^* + Z_i'\,. \end{cases}$$

In this representation the random variables $(\mathbf{A}_\mu)_{1\leq\mu\leq m} \overset{\mathrm{iid}}{\sim} P_A$ are arbitrary, and $(Z_\mu)_{1\leq\mu\leq m} \overset{\mathrm{iid}}{\sim} \mathcal{N}(0,1)$, $(Z_i')_{1\leq i\leq n} \overset{\mathrm{iid}}{\sim} \mathcal{N}(0,1)$. We have

$$\begin{aligned} P_{\mathrm{out}}(Y_{t,\mu}|s_{t,\mu}(\mathbf{x}, w_\mu)) &= \int dP_A(\mathbf{a}_\mu)\, \frac{1}{\sqrt{2\pi}} \exp\Big\{-\frac{1}{2}\Big(Y_{t,\mu} - \varphi(s_{t,\mu}(\mathbf{x}, w_\mu), \mathbf{a}_\mu)\Big)^2\Big\} \\ &= \int dP_A(\mathbf{a}_\mu)\, \frac{1}{\sqrt{2\pi}} \exp\Big\{-\frac{1}{2}\Big(\Gamma_{t,\mu}(\mathbf{x}, w_\mu, \mathbf{a}_\mu) + Z_\mu\Big)^2\Big\} \end{aligned} \tag{201}$$

where, using the random function representation,

$$\Gamma_{t,\mu}(\mathbf{x},w_\mu,\mathbf{a}_\mu) \tag{202}$$
$$= \varphi\Big(\sqrt{\frac{1-t}{n}}[\mathbf{\Phi}\mathbf{X}^*]_\mu + k_1(t)V_\mu + k_2(t)W^*_\mu, \mathbf{A}_\mu\Big) - \varphi\Big(\sqrt{\frac{1-t}{n}}[\mathbf{\Phi}\mathbf{x}]_\mu + k_1(t)V_\mu + k_2(t)w_\mu, \mathbf{a}_\mu\Big)\,.$$

From (200), (201), (202) we can express the free entropy of the interpolating model as

$$\frac{1}{n}\ln \mathcal{Z}_{t,\epsilon} = \frac{1}{n}\ln \int dP_0(\mathbf{x})dP_A(\mathbf{a})\mathcal{D}\mathbf{w}\, e^{-\mathcal{H}_{t,\epsilon}(\mathbf{x},\mathbf{w},\mathbf{a})} - \frac{m}{2n}\ln(2\pi) \tag{203}$$

where $\mathcal{D}\mathbf{w}$ denote the standard m-dimensional Gaussian measure and where the Hamiltonian $\mathcal{H}_{t,\epsilon}$ is re-expressed as

$$\mathcal{H}_{t,\epsilon}(\mathbf{x},\mathbf{w},\mathbf{a}) = \frac{1}{2}\sum_{\mu=1}^{m}\big(\Gamma_{t,\mu}(\mathbf{x},w_\mu,\mathbf{a}_\mu) + Z_\mu\big)^2 + \frac{1}{2}\sum_{i=1}^{n}\big(\sqrt{R_1(t)}\,(X^*_i - x_i) + Z'_i\big)^2\,. \tag{204}$$

The interpretation here is that $\mathbf{x},\mathbf{w},\mathbf{a}$ are *annealed* variables and $\mathbf{\Phi},\mathbf{V},\mathbf{A},\mathbf{Y}_t,\mathbf{Y}'_t,\mathbf{X}^*,\mathbf{W}^*$, or equivalently $\mathbf{\Phi},\mathbf{V},\mathbf{A},\mathbf{Z},\mathbf{Z}',\mathbf{X}^*,\mathbf{W}^*$ are *quenched*. The inference problem is to recover $\mathbf{X}^*,\mathbf{W}^*$ given $\mathbf{\Phi},\mathbf{V},\mathbf{Y}_t,\mathbf{Y}'_t$. The free entropy can be further re-expressed as

$$\frac{1}{n}\ln \mathcal{Z}_{t,\epsilon} = \frac{1}{n}\ln \hat{\mathcal{Z}}_{t,\epsilon} - \frac{1}{2n}\sum_{\mu=1}^{m} Z_\mu^2 - \frac{1}{2n}\sum_{i=1}^{n} Z_i'^2 - \frac{m}{2n}\ln(2\pi) \tag{205}$$

where

$$\frac{1}{n}\ln \hat{\mathcal{Z}}_{t,\epsilon} = \frac{1}{n}\ln \int dP_0(\mathbf{x})dP_A(\mathbf{a})\mathcal{D}\mathbf{w}\, e^{-\hat{\mathcal{H}}_{t,\epsilon}(\mathbf{x},\mathbf{w},\mathbf{a})}\,, \tag{206}$$
$$\hat{\mathcal{H}}_{t,\epsilon}(\mathbf{x},\mathbf{w},\mathbf{a}) = \frac{1}{2}\sum_{\mu=1}^{m}\Big\{\Gamma_{t,\mu}(\mathbf{x},w_\mu,\mathbf{a}_\mu)^2 + 2Z_\mu\Gamma_{t,\mu}(\mathbf{x},w_\mu,\mathbf{a}_\mu)\Big\}$$
$$+\frac{1}{2}\sum_{i=1}^{n}\Big\{R_1(t)(X^*_i - x_i)^2 + 2Z'_i\sqrt{R_1(t)}(X^*_i - x_i)\Big\}\,. \tag{207}$$

In order to prove Theorem 6 it remains to show that there exists a constant $C(\varphi,S,\alpha) > 0$ such that $\mathrm{Var}(\ln \hat{\mathcal{Z}}_{t,\epsilon}/n) \le C(\varphi,S,\alpha)/n$. This concentration property together with (205) implies (199).

We will first show concentration w.r.t. all Gaussian variables $\mathbf{\Phi},\mathbf{V},\mathbf{Z},\mathbf{Z}',\mathbf{W}^*$ thanks to the classical Gaussian Poincaré inequality, then the concentration w.r.t. $\mathbf{A}$ and finally the one w.r.t. $\mathbf{X}^*$ using classical bounded differences arguments. The order in which we prove the concentrations matters. We recall here these two variances bounds. The reader can refer to [62] (Chapter 3) for detailed proofs of these statements.

Proposition 27 (Gaussian Poincaré inequality)**.** *Let $\boldsymbol{U} = (U_1,\dots,U_N)$ be a vector of N independent standard normal random variables. Let $g:\mathbb{R}^N \to \mathbb{R}$ be a continuously differentiable function. Then*

$$\mathrm{Var}(g(\boldsymbol{U})) \le \mathbb{E}\big[\|\nabla g(\boldsymbol{U})\|^2\big]\,. \tag{208}$$

Proposition 28 (Bounded difference)**.** *Let $\mathcal{U} \subset \mathbb{R}$. Let $g:\mathcal{U}^N \to \mathbb{R}$ a function that satisfies the bounded difference property, i.e., there exists some constants $c_1,\dots,c_N \ge 0$ such that*

$$\sup_{\substack{u_1,\dots,u_N\in\mathcal{U}^N\\ u'_i\in\mathcal{U}}} |g(u_1,\dots,u_i,\dots,u_N) - g(u_1,\dots,u'_i,\dots,u_N)| \le c_i \quad \textit{for all} \quad 1\le i\le N\,.$$

Let $\mathbf{U} = (U_1, \dots, U_N)$ *be a vector of* N *independent random variables that take values in* $\mathcal{U}$. *Then*

$$\mathrm{Var}(g(\boldsymbol{U})) \leq \frac{1}{4}\sum_{i=1}^{N} c_i^2 \,. \tag{209}$$

E.1.1 Concentration with respect to the Gaussian random variables Z, Z′, V, W*, Φ

Lemma 24. *Let* $\mathbb{E}_G$ *denotes the joint expectation w.r.t.* $\boldsymbol{Z}, \boldsymbol{Z}', \boldsymbol{V}, \boldsymbol{W}^*, \boldsymbol{\Phi}$ *only. There exists a constant* $C(\varphi, S, \alpha) > 0$ *such that*

$$\mathbb{E}\Big[\Big(\frac{1}{n}\ln \hat{\mathcal{Z}}_{t,\epsilon} - \frac{1}{n}\mathbb{E}_G \ln \hat{\mathcal{Z}}_{t,\epsilon}\Big)^2\Big] \leq \frac{C(\varphi, S, \alpha)}{n} \,. \tag{210}$$

Lemma 24 follows directly from Lemmas 25 and 26 below.

Lemma 25. *Let* $\mathbb{E}_{\mathbf{Z},\mathbf{Z}'}$ *denotes the expectation w.r.t.* $\boldsymbol{Z}, \boldsymbol{Z}'$ *only. There exists a constant* $C(\varphi, S, \alpha) > 0$ *such that*

$$\mathbb{E}\Big[\Big(\frac{1}{n}\ln \hat{\mathcal{Z}}_{t,\epsilon} - \frac{1}{n}\mathbb{E}_{\mathbf{Z},\mathbf{Z}'} \ln \hat{\mathcal{Z}}_{t,\epsilon}\Big)^2\Big] \leq \frac{C(\varphi, S, \alpha)}{n} \,. \tag{211}$$

Proof. We consider here $g = \ln \hat{\mathcal{Z}}_{t,\epsilon}/n$ only as a function of $\mathbf{Z}$ and $\mathbf{Z}'$ and work conditionally on all other random variables. We have

$$\|\nabla g\|^2 = \sum_{\mu=1}^{m} \Big|\frac{\partial g}{\partial Z_\mu}\Big|^2 + \sum_{i=1}^{n} \Big|\frac{\partial g}{\partial Z_i'}\Big|^2 . \tag{212}$$

Each of these partial derivatives are of the form $|\partial_u g| = |n^{-1}\langle \partial_u \hat{\mathcal{H}}_{t,\epsilon}\rangle_{\hat{\mathcal{H}}_{t,\epsilon}}|$ where the Gibbs bracket $\langle - \rangle_{\hat{\mathcal{H}}_{t,\epsilon}}$ pertains to the effective Hamiltonian (207). We find

$$\Big|\frac{\partial g}{\partial Z_\mu}\Big| = n^{-1}\big|\langle \Gamma_{t,\mu}\rangle_{\hat{\mathcal{H}}_{t,\epsilon}}\big| \leq 2n^{-1}\sup|\varphi| \,,$$
$$\Big|\frac{\partial g}{\partial Z_i'}\Big| = n^{-1}\sqrt{R_1(t)}\big|X_i^* - \langle x_i\rangle_{\hat{\mathcal{H}}_{t,\epsilon}}\big| \leq 2n^{-1}\sqrt{K}S \,,$$

and replacing in (212) we get $\|\nabla g\|^2 \leq 4n^{-1}(\frac{m}{n}(\sup|\varphi|)^2 + KS^2)$. Applying Proposition 27 we have

$$\mathbb{E}_{\mathbf{Z},\mathbf{Z}'}\Big[\Big(\frac{1}{n}\ln \hat{\mathcal{Z}}_{t,\epsilon} - \frac{1}{n}\mathbb{E}_{\mathbf{Z},\mathbf{Z}'} \ln \hat{\mathcal{Z}}_{t,\epsilon}\Big)^2\Big] \leq \frac{C(\varphi, S, \alpha)}{n} \,. \tag{213}$$

Taking the expectation in (213) gives the lemma. □

Lemma 26. *There exists a constant* $C(\varphi, S, \alpha) > 0$ *such that*

$$\mathbb{E}\Big[\Big(\frac{1}{n}\mathbb{E}_{\mathbf{Z},\mathbf{Z}'} \ln \hat{\mathcal{Z}}_{t,\epsilon} - \frac{1}{n}\mathbb{E}_G \ln \hat{\mathcal{Z}}_{t,\epsilon}\Big)^2\Big] \leq \frac{C(\varphi, S, \alpha)}{n} \,. \tag{214}$$

Proof. We consider here $g = \mathbb{E}_{\mathbf{Z},\mathbf{Z}'} \ln \hat{\mathcal{Z}}_{t,\epsilon}/n$ as a function of $\mathbf{V}$, $\mathbf{W}^*$, $\boldsymbol{\Phi}$ and we work conditionally on the other random variables. Let $\partial_x\varphi$ be the derivative of φ w.r.t. its first argument. We compute

$$\begin{aligned}\Big|\frac{\partial g}{\partial V_\mu}\Big| &= n^{-1}\Big|\mathbb{E}_{\mathbf{Z},\mathbf{Z}'}\Big\langle(\Gamma_{t,\mu} + Z_\mu)\frac{\partial \Gamma_{t,\mu}}{\partial V_\mu}\Big\rangle_{\hat{\mathcal{H}}_{t,\epsilon}}\Big| \\ &\leq n^{-1}\mathbb{E}_{\mathbf{Z},\mathbf{Z}'}\Big[(2\sup|\varphi| + |Z_\mu|)\,2\sqrt{K}\sup|\partial_x\varphi|\Big] = n^{-1}\Big(2\sup|\varphi| + \sqrt{\frac{2}{\pi}}\Big)\,2\sqrt{K}\sup|\partial_x\varphi| \,.\end{aligned}$$

The same inequality holds for $|\frac{\partial g}{\partial W_\mu^*}|$. To compute the derivative w.r.t. $\Phi_{\mu i}$ we first remark

$$\begin{aligned}\frac{\partial \Gamma_{t,\mu}}{\partial \Phi_{\mu i}} =&\sqrt{\frac{1-t}{n}}\Big\{X_i^*\,\partial_x\varphi\Big(\sqrt{\frac{1-t}{n}}[\mathbf{\Phi}\mathbf{X}^*]_\mu + k_1(t)V_\mu + k_2(t)W_\mu^*, \mathbf{A}_\mu\Big)\\ &\qquad - x_i\,\partial_x\varphi\Big(\sqrt{\frac{1-t}{n}}[\mathbf{\Phi}\mathbf{x}]_\mu + k_1(t)V_\mu + k_2(t)w_\mu, \mathbf{a}_\mu\Big)\Big\}.\end{aligned}$$

Therefore, as $t \in [0,1]$,

$$\begin{aligned}\Big|\frac{\partial g}{\partial \Phi_{\mu i}}\Big| &= n^{-1}\Big|\mathbb{E}_{\mathbf{Z},\mathbf{Z}'}\Big\langle(\Gamma_{t,\mu}+Z_\mu)\frac{\partial \Gamma_{t,\mu}}{\partial \Phi_{\mu i}}\Big\rangle_{\hat{\mathcal{H}}_{t,\epsilon}}\Big|\\ &\le n^{-3/2}\mathbb{E}_{\mathbf{Z},\mathbf{Z}'}\Big[(2\sup|\varphi| + |Z_\mu|)\,2S\sup|\partial_x\varphi|\Big] = n^{-3/2}\Big(2\sup|\varphi| + \sqrt{\frac{2}{\pi}}\Big)\,2S\sup|\partial_x\varphi|\,.\end{aligned}$$

Putting these inequalities together we find

$$\begin{aligned}\|\nabla g\|^2 &= \sum_{\mu=1}^m\Big|\frac{\partial g}{\partial V_\mu}\Big|^2 + \sum_{\mu=1}^m\Big|\frac{\partial g}{\partial W_\mu^*}\Big|^2 + \sum_{\mu=1}^m\sum_{i=1}^n\Big|\frac{\partial g}{\partial \Phi_{\mu i}}\Big|^2\\ &\le 2\frac{m}{n^2}\Big(2\sup|\varphi| + \sqrt{\frac{2}{\pi}}\Big)^2 4K(\sup|\partial_x\varphi|)^2 + \frac{mn}{n^3}\Big(2\sup|\varphi| + \sqrt{\frac{2}{\pi}}\Big)^2\, 4S^2(\sup|\partial_x\varphi|)^2\,.\end{aligned}$$

The lemma follows again from Proposition 27. □

E.1.2 Bounded difference with respect to A_μ

The next step is an application of the variance bound of Lemma 28 to show that $\mathbb{E}_G \ln\hat{\mathcal{Z}}_{t,\epsilon}/n$ concentrates w.r.t. $\mathbf{A}$ (we still keep $\mathbf{X}^*$ fixed for the moment).

Lemma 27. *Let $\mathbb{E}_{\mathbf{A}}$ denotes the expectation w.r.t. $\mathbf{A}$ only. There exists a constant $C(\varphi,\alpha) > 0$ such that*

$$\mathbb{E}\Big[\Big(\frac{1}{n}\mathbb{E}_G \ln\hat{\mathcal{Z}}_{t,\epsilon} - \frac{1}{n}\mathbb{E}_{G,\mathbf{A}}\ln\hat{\mathcal{Z}}_{t,\epsilon}\Big)^2\Big] \le \frac{C(\varphi,\alpha)}{n}\,. \tag{215}$$

Proof. Let us consider $g = \mathbb{E}_G \ln\hat{\mathcal{Z}}_{t,\epsilon}/n$ as a function of $\mathbf{A}$ only. Let $\nu \in \{1,\dots,m\}$. We must estimate variations $g(\mathbf{A}) - g(\mathbf{A}^{(\nu)})$ corresponding to two configurations $\mathbf{A}$ and $\mathbf{A}^{(\nu)}$ with $A_\mu^{(\nu)} = A_\mu$ for $\mu \neq \nu$ and $A_\nu^{(\nu)} = \tilde{A}_\nu$. We will use the notations $\hat{\mathcal{H}}_{t,\epsilon}^{(\nu)}$ and $\Gamma_{t,\mu}^{(\nu)}$ to denote respectively the quantities $\hat{\mathcal{H}}_{t,\epsilon}$ and $\Gamma_{t,\mu}$ where $\mathbf{A}$ is replaced by $\mathbf{A}^{(\nu)}$. By an application of Jensen's inequality one finds

$$\frac{1}{n}\mathbb{E}_G\langle\hat{\mathcal{H}}_{t,\epsilon}^{(\nu)} - \hat{\mathcal{H}}_{t,\epsilon}\rangle_{\hat{\mathcal{H}}_{t,\epsilon}^{(\nu)}} \le g(\mathbf{A}) - g(\mathbf{A}^{(\nu)}) \le \frac{1}{n}\mathbb{E}_G\langle\hat{\mathcal{H}}_{t,\epsilon}^{(\nu)} - \hat{\mathcal{H}}_{t,\epsilon}\rangle_{\hat{\mathcal{H}}_{t,\epsilon}} \tag{216}$$

where the Gibbs brackets pertain to the effective Hamiltonians (207). From (207) we obtain

$$\hat{\mathcal{H}}_{t,\epsilon}^{(\nu)} - \hat{\mathcal{H}}_{t,\epsilon} = \frac{1}{2}\sum_{\mu=1}^m\Big(\Gamma_{t,\mu}^{(\nu)2} - \Gamma_{t,\mu}^2 + 2Z_\mu(\Gamma_{t,\mu}^{(\nu)} - \Gamma_{t,\mu})\Big) = \frac{1}{2}\Big(\Gamma_{t,\nu}^{(\nu)2} - \Gamma_{t,\nu}^2 + 2Z_\nu(\Gamma_{t,\nu}^{(\nu)} - \Gamma_{t,\nu})\Big)\,.$$

Consequently

$$\begin{aligned}\frac{1}{2n}\mathbb{E}_G\Big\langle\Gamma_{t,\nu}^{(\nu)2} - \Gamma_{t,\nu}^2 + 2Z_\nu(\Gamma_{t,\nu}^{(\nu)} - \Gamma_{t,\nu})\Big\rangle_{\hat{\mathcal{H}}_{t,\epsilon}^{(\nu)}} &\le g(\mathbf{A}) - g(\mathbf{A}^{(\nu)})\\ &\le \frac{1}{2n}\mathbb{E}_G\Big\langle\Gamma_{t,\nu}^{(\nu)2} - \Gamma_{t,\nu}^2 + 2Z_\nu(\Gamma_{t,\nu}^{(\nu)} - \Gamma_{t,\nu})\Big\rangle_{\hat{\mathcal{H}}_{t,\epsilon}}.\end{aligned} \tag{217}$$

Notice that $\left|\Gamma_{t,\nu}^{(\nu)2} - \Gamma_{t,\nu}^2 + 2Z_\nu(\Gamma_{t,\nu}^{(\nu)} - \Gamma_{t,\nu})\right| \le 8(\sup|\varphi|)^2 + 8|Z_\nu|\sup|\varphi|$. Thus we conclude by (217) that g satisfies a bounded difference property:

$$|g(\mathbf{A}) - g(\mathbf{A}^{(\nu)})| \le \frac{4}{n}\sup|\varphi|\Big(\sup|\varphi| + \sqrt{\frac{2}{\pi}}\Big)\,. \tag{218}$$

Lemma 27 follows then by an application of Proposition 28. □

E.1.3 Bounded difference with respect to X_i^*

Let $\mathbb{E}_\Theta = \mathbb{E}_{\mathbf{A},G}$ denote the expectation w.r.t. all quenched variables except $\mathbf{X}^*$. It remains to bound the variance of $\mathbb{E}_\Theta \ln \hat{\mathcal{Z}}_{t,\epsilon}/n$ (which only depends on $\mathbf{X}^*$).

Lemma 28. *There exists a constant $C(\varphi,S,\alpha) > 0$ such that*

$$\mathbb{E}\Big[\Big(\frac{1}{n}\mathbb{E}_\Theta \ln \hat{\mathcal{Z}}_{t,\epsilon} - \frac{1}{n}\mathbb{E}\ln \hat{\mathcal{Z}}_{t,\epsilon}\Big)^2\Big] \le \frac{C(\varphi,S,\alpha)}{n}\,. \tag{219}$$

Proof. The lemma is proved using again a bounded difference argument. Let $g = \mathbb{E}_\Theta \ln \hat{\mathcal{Z}}_{t,\epsilon}/n$ a function of $\mathbf{X}^*$. Let $j \in \{1,\dots,n\}$. Let $\mathbf{X}^*, \mathbf{X}^{*(j)} \in [-S,S]^n$ be two input signals such that $X_i^{*(j)} = X_i^*$ for $i \neq j$.

We are going to interpolate between $g(\mathbf{X}^*)$ and $g(\mathbf{X}^{*(j)})$. For $s \in [0,1]$ we define

$$\psi(s) = g(s\mathbf{X}^* + (1-s)\mathbf{X}^{*(j)})\,.$$

Obviously $\psi(1) = g(\mathbf{X}^*)$ and $\psi(0) = g(\mathbf{X}^{*(j)})$. Using Gaussian integration by parts, it is not difficult to verify that for $s \in [0,1]$

$$|\psi'(s)| \le \frac{C(\varphi,S,\alpha)}{n}\,.$$

This implies the bounded difference property $|g(\mathbf{X}^*) - g(\mathbf{X}^{*(j)})| \le C(\varphi,S,\alpha)/n$ and using Proposition 28 we obtain the lemma. □

E.1.4 Proof of Theorem 6

From Lemmas 24, 27 and 28 above, we obtain directly that $\mathrm{Var}(\ln \hat{\mathcal{Z}}_{t,\epsilon}/n) \le C(\varphi,S,\alpha)/n$ for some constant $C(\varphi,S,\alpha) > 0$. As mentioned before this implies, thanks to (205), the Theorem 6.

E.2 Concentration of the overlap

In this appendix we provide the proof of Proposition 4. Recall the notation $\langle - \rangle_{n,t,\epsilon}$ for the Gibbs bracket associated to the Hamiltonian (76). It is crucial that it preserves the Nishimori identity of Appendix A.1, i.e. it must come from an inference problem with known parameters. Consider the corresponding average free entropy $f_{n,\epsilon}(t)$. In this section we think of it as a function of $R_1 = R_1(t,\epsilon)$ and $R_2 = R_2(t,\epsilon)$ given by (70), i.e. $(R_1,R_2) \mapsto f_{n,\epsilon}(t)$. Similarly the free entropy for a realization of the quenched variables is also viewed here as a function $(R_1,R_2) \mapsto F_{n,\epsilon}(t) := \ln \mathcal{Z}_{t,\epsilon}(\mathbf{Y}_t, \mathbf{Y}_t', \mathbf{\Phi}, \mathbf{V})/n$. For this section, we drop the indices in the Gibbs bracket $\langle - \rangle_{n,t,\epsilon}$ and simply write $\langle - \rangle$.

Let

$$\mathcal{L} := \frac{1}{n}\sum_{i=1}^n \Big(\frac{x_i^2}{2} - x_i X_i^* - \frac{x_i Z_i'}{2\sqrt{R_1}}\Big)\,.$$

The fluctuations of the overlap $Q := n^{-1}\sum_{i=1}^n X_i^* x_i$ and those of $\mathcal{L}$ are related through the remarkable identity

$$\mathbb{E}\big\langle(\mathcal{L}-\mathbb{E}\langle\mathcal{L}\rangle)^2\big\rangle = \frac{1}{4}\mathbb{E}\big\langle(Q-\mathbb{E}\langle Q\rangle)^2\big\rangle + \frac{1}{2}\mathbb{E}[\langle Q^2\rangle - \langle Q\rangle^2] + \frac{1}{4nR_1}\mathbb{E}[(X_1^*)^2]\,. \tag{220}$$

In particular

$$\mathbb{E}\big\langle(\mathcal{L}-\mathbb{E}\langle\mathcal{L}\rangle)^2\big\rangle \geq \frac{1}{4}\mathbb{E}\big\langle(Q-\mathbb{E}\langle Q\rangle)^2\big\rangle\,. \tag{221}$$

A detailed derivation of (220) involves only lengthy but straightforward algebra, using the Nishimori identity and integrations by parts w.r.t. the Gaussian noise Z_i', and can be found in Sec. 6 of [41]. Proposition 4 is then a direct consequence of the following:

Proposition 29 (Concentration of $\mathcal{L}$ on $\mathbb{E}\langle\mathcal{L}\rangle$)**.** *Let $\mathcal{B}_n := [s_n, 2s_n]^2$, where the sequence $(s_n) \in (0,1/2]^{\mathbb{N}}$. Assume that the interpolation functions (r_ϵ) and (q_ϵ) are regular (recall Definition 1). Under assumptions (H1), (H2) and (H3) there exists a constant $C(\varphi,S,\alpha)$ such that*

$$\int_{\mathcal{B}_n} d\epsilon\, \mathbb{E}\big\langle(\mathcal{L}-\mathbb{E}\langle\mathcal{L}\rangle_{n,t,\epsilon})^2\big\rangle_{n,t,\epsilon} \leq \frac{C(\varphi,S,\alpha)}{n^{1/4}}\,. \tag{222}$$

The proof of this proposition is broken in two parts. Notice that

$$\mathbb{E}\big\langle(\mathcal{L}-\mathbb{E}\langle\mathcal{L}\rangle)^2\big\rangle = \mathbb{E}\big\langle(\mathcal{L}-\langle\mathcal{L}\rangle)^2\big\rangle + \mathbb{E}\big[(\langle\mathcal{L}\rangle-\mathbb{E}\langle\mathcal{L}\rangle)^2\big]\,. \tag{223}$$

Thus it suffices to prove the two following lemmas (see the proofs below). The first lemma expresses concentration w.r.t. the posterior distribution (or "thermal fluctuations") and is an elementary consequence of concavity properties of the free entropy and the Nishimori identity.

Lemma 29 (Concentration of $\mathcal{L}$ on $\langle\mathcal{L}\rangle$)**.** *Under the same hypotheses as in Proposition 29 we have*

$$\int_{\mathcal{B}_n} d\epsilon\, \mathbb{E}\big\langle(\mathcal{L}-\langle\mathcal{L}\rangle_{n,t,\epsilon})^2\big\rangle_{n,t,\epsilon} \leq \frac{\rho(1+\rho)}{n}\,. \tag{224}$$

The second lemma expresses the concentration of the average overlap w.r.t. the realizations of quenched disorder variables and is a consequence of the concentration of the free entropy (more precisely Theorem 6 in Appendix E.1).

Lemma 30 (Concentration of $\langle\mathcal{L}\rangle$ on $\mathbb{E}\langle\mathcal{L}\rangle$)**.** *Under the same hypotheses as in Proposition 29 there exists a constant $C(\varphi,S,\alpha)$ such that*

$$\int_{\mathcal{B}_n} d\epsilon\, \mathbb{E}\big[(\langle\mathcal{L}\rangle_{n,t,\epsilon}-\mathbb{E}\langle\mathcal{L}\rangle_{n,t,\epsilon})^2\big] \leq \frac{C(\varphi,S,\alpha)}{n^{1/4}}\,. \tag{225}$$

We now turn to the proof of Lemmas 29 and 30. The main ingredient is a set of formulas for the first two derivatives of the free entropy w.r.t. $R_1 = R_1(t,\epsilon)$. For any given realisation of the quenched disorder,

$$\frac{dF_{n,\epsilon}(t)}{dR_1} = -\langle\mathcal{L}\rangle - \frac{1}{2n}\sum_{i=1}^n\Big((X_i^*)^2 + \frac{1}{\sqrt{R_1}}X_i^* Z_i'\Big)\,, \tag{226}$$

$$\frac{1}{n}\frac{d^2F_{n,\epsilon}(t)}{dR_1^2} = \langle\mathcal{L}^2\rangle - \langle\mathcal{L}\rangle^2 - \frac{1}{4n^2R_1^{3/2}}\sum_{i=1}^n\langle x_i\rangle Z_i'\,. \tag{227}$$

Averaging (226) and (227), using a Gaussian integration by parts w.r.t. Z_i' and the (Nishimori) identity $\mathbb{E}\langle x_i X_i^* \rangle = \mathbb{E}[\langle x_i \rangle^2]$ we find

$$\frac{df_{n,\epsilon}(t)}{dR_1} = -\mathbb{E}\langle \mathcal{L} \rangle - \frac{\rho}{2} = \frac{1}{2n}\sum_{i=1}^n \mathbb{E}[\langle x_i \rangle^2] - \frac{\rho}{2}\,, \tag{228}$$

$$\frac{1}{n}\frac{d^2 f_{n,\epsilon}(t)}{dR_1^2} = \mathbb{E}[\langle \mathcal{L}^2 \rangle - \langle \mathcal{L} \rangle^2] - \frac{1}{4n^2 R_1}\sum_{i=1}^n \mathbb{E}[\langle x_i^2 \rangle - \langle x_i \rangle^2]\,. \tag{229}$$

Proof of Lemma 29

From (229) we have

$$\begin{aligned}\mathbb{E}\big\langle (\mathcal{L} - \langle \mathcal{L} \rangle)^2 \big\rangle &= \frac{1}{n}\frac{d^2 f_{n,\epsilon}(t)}{dR_1^2} + \frac{1}{4n^2 R_1}\sum_{i=1}^n \mathbb{E}[\langle x_i^2 \rangle - \langle x_i \rangle^2] \\ &\le \frac{1}{n}\frac{d^2 f_{n,\epsilon}(t)}{dR_1^2} + \frac{\rho}{4n\epsilon_1}\,,\end{aligned} \tag{230}$$

where we used $\mathbb{E}\langle x_i^2 \rangle = \mathbb{E}_{P_0}[(X^*)^2] = \rho$ by the Nishimori identity, and $R_1 \ge \epsilon_1$. Recall $\mathcal{B}_n := [s_n, 2s_n]^2$. By assumption q and r are regular. Therefore $R^t : (\epsilon_1, \epsilon_2) \mapsto (R_1(t,\epsilon), R_2(t,\epsilon))$ is a diffeomorphism whose Jacobian $J(R^t)$ verifies $J(R^t)(\epsilon) \ge 1$ for all $\epsilon \in \mathcal{B}_n$. Integrating over $\epsilon \in \mathcal{B}_n$ we obtain

$$\begin{aligned}\int_{\mathcal{B}_n} d\epsilon\, \mathbb{E}\big\langle (\mathcal{L} - \langle \mathcal{L} \rangle)^2 \big\rangle &\le \frac{1}{n}\int_{R^t(\mathcal{B}_n)} \frac{dR_1 dR_2}{J(R^t)}\frac{d^2 f_{n,\epsilon}(t)}{dR_1^2} + \frac{\rho s_n}{4n}\int_{s_n}^{2s_n}\frac{d\epsilon_1}{\epsilon_1} \\ &\le \frac{1}{n}\int_{R^t(\mathcal{B}_n)} dR_1 dR_2 \frac{d^2 f_{n,\epsilon}(t)}{dR_1^2} + \frac{\rho s_n}{4n}\ln 2\,,\end{aligned} \tag{231}$$

where in the integral above $J(R^t)$ is a function of $(R^t)^{-1}(R_1, R_2)$. Note that from (70) we have $R^t(\mathcal{B}_n) \subset [s_n, 2s_n + r_{\max}] \times [s_n, 2s_n + \rho]$ and therefore

$$\begin{aligned}\int_{\mathcal{B}_n} d\epsilon\, \mathbb{E}\big\langle (\mathcal{L} - \langle \mathcal{L} \rangle)^2 \big\rangle &\le \frac{1}{n}\int_{s_n}^{2s_n+\rho} dR_2 \Big\{ \frac{df_{n,\epsilon}(t)}{dR_1}\Big|_{R_1 = s_n} - \frac{df_{n,\epsilon}(t)}{dR_1}\Big|_{R_1 = 2s_n + r_{\max}} \Big\} + \frac{\rho s_n}{4n}\ln 2 \\ &\le \frac{\rho(s_n + \rho)}{n} + \frac{\rho s_n}{4n}\ln 2\end{aligned} \tag{232}$$

using (228) combined with $\mathbb{E}\langle x_i^2 \rangle = \rho$ to assert that the derivative of the free entropy is bounded in absolute value by $\rho/2$. This concludes the proof of Lemma 29 using $s_n \le 1/2$ and $(\ln 2)/4 < 1$. □

Proof of Lemma 30

Consider the two functions

$$\widetilde{F}(R_1) := F_{n,\epsilon}(t) - \frac{\sqrt{R_1}}{n} S \sum_{i=1}^n |Z_i'|\,, \qquad \widetilde{f}(R_1) := \mathbb{E}\widetilde{F}(R_1) = f_{n,\epsilon}(t) - \frac{\sqrt{R_1}}{n} S \sum_{i=1}^n \mathbb{E}|Z_i'|\,. \tag{233}$$

Because of (227) we see that the second derivative of $\widetilde{F}(R_1)$ is positive so that it is convex (without this extra term $F_{n,\epsilon}(t)$ is not necessarily convex in R_1, although $f_{n,\epsilon}(t)$ is, which can be shown easily). Note that $\widetilde{f}(R_1)$ is convex too. Convexity allows us to use the following lemma (proved at the end of this section):

Lemma 31 (A bound on differences of derivatives due to convexity)**.** *Let $G(x)$ and $g(x)$ be convex functions.*

Let $\delta > 0$ and define $C_\delta^+(x) := g'(x+\delta) - g'(x) \geq 0$ and $C_\delta^-(x) := g'(x) - g'(x-\delta) \geq 0$. Then

$$|G'(x) - g'(x)| \leq \delta^{-1} \sum_{u \in \{x-\delta, x, x+\delta\}} |G(u) - g(u)| + C_\delta^+(x) + C_\delta^-(x)\,.$$

From (233)

$$\widetilde{F}(R_1) - \widetilde{f}(R_1) = F_{n,\epsilon}(t) - f_{n,\epsilon}(t) - \sqrt{R_1} S A\,, \quad \text{with} \quad A = \frac{1}{n}\sum_{i=1}^n \big(|Z_i'| - \mathbb{E}|Z_i'|\big)\,. \tag{234}$$

and from (226), (228) we obtain for the difference of derivatives (w.r.t. R_1)

$$\widetilde{F}'(R_1) - \widetilde{f}'(R_1) = \mathbb{E}\langle \mathcal{L}\rangle - \langle \mathcal{L}\rangle + \frac{\rho}{2} - \frac{1}{2n}\sum_{i=1}^n \Big((X_i^*)^2 + \frac{1}{\sqrt{R_1}} X_i^* Z_i'\Big) - \frac{SA}{2\sqrt{R_1}}\,. \tag{235}$$

From (234), (235) it is easy to show that Lemma 31 implies

$$\begin{aligned} |\langle \mathcal{L}\rangle - \mathbb{E}\langle \mathcal{L}\rangle| \leq \delta^{-1} &\sum_{u \in \{R_1-\delta, R_1, R_1+\delta\}} \big(|F_{n,\epsilon}(t, R_1 = u) - f_{n,\epsilon}(t, R_1 = u)| + S|A|\sqrt{u}\big) \\ &+ C_\delta^+(R_1) + C_\delta^-(R_1) + \frac{S|A|}{2\sqrt{\epsilon_1}} + \Big|\frac{\rho}{2} - \frac{1}{2n}\sum_{i=1}^n \Big((X_i^*)^2 + \frac{1}{\sqrt{R_1}} X_i^* Z_i'\Big)\Big| \end{aligned} \tag{236}$$

where $C_\delta^+(R_1) := \widetilde{f}'(R_1+\delta) - \widetilde{f}'(R_1) \geq 0$ and $C_\delta^-(R_1) := \widetilde{f}'(R_1) - \widetilde{f}'(R_1-\delta) \geq 0$. We used $R_1 \geq \epsilon_1$ for the term $S|A|/(2\sqrt{\epsilon_1})$. Note that δ will be chosen later on strictly smaller than s_n (namely $\delta = s_n n^{-1/4}$) so that $R_1 - \delta \geq \epsilon_1 - \delta \geq s_n - \delta$ remains positive. Remark that by independence of the noise variables $\mathbb{E}[A^2] \leq a n^{-1}$ for some constant $a > 0$; and that by independence between signal and noise, the last term in the absolute value in (236), call it B, is satisfies $\mathbb{E}[B^2] \leq b n^{-1}$ for some constant $b > 0$. We now square the identity (236) and take its expectation. Then using $(\sum_{i=1}^p v_i)^2 \leq p \sum_{i=1}^p v_i^2$ (by convexity), and that $R_1 \leq K$ ($K = 1 + \max(\rho, r_{\max})$ upper bounds both R_1 and R_2 given by (70)), as well as the free entropy concentration Theorem 6,

$$\frac{1}{10}\mathbb{E}\big[\big(\langle \mathcal{L}\rangle - \mathbb{E}\langle \mathcal{L}\rangle\big)^2\big] \leq 3\delta^{-2}\big(C + aS^2(K+\delta)\big)\frac{1}{n} + C_\delta^+(R_1)^2 + C_\delta^-(R_1)^2 + \frac{S^2 a}{4\epsilon_1 n} + \frac{b}{n}\,. \tag{237}$$

where $C = C(\varphi, S, \alpha)$ is a positive constant depending only on φ, S and α that comes from the use of Theorem 6. Recall $|C_\delta^\pm(R_1)| = |\widetilde{f}'(R_1 \pm \delta) - \widetilde{f}'(R_1)|$. We have

$$|\widetilde{f}'(R_1)| \leq \frac{1}{2}\Big(\rho + \frac{S}{\sqrt{R_1}}\Big) \leq \frac{1}{2}\Big(\rho + \frac{S}{\sqrt{\epsilon_1}}\Big) \tag{238}$$

from (228), (233) and $R_1 \geq \epsilon_1$. This implies $|C_\delta^\pm(R_1)| \leq \rho + S/\sqrt{\epsilon_1} \leq \rho + S/\sqrt{s_n}$ as $\epsilon_1 \geq s_n$. Recall also

that $\mathcal{B}_n := [s_n, 2s_n]^2$. Then

$$\begin{aligned}
\int_{\mathcal{B}_n} d\epsilon \left\{ C_\delta^+(R_1(t,\epsilon))^2 + C_\delta^-(R_1(t,\epsilon))^2 \right\} \\
&\leq \left(\rho + \frac{S}{\sqrt{s_n}}\right) \int_{\mathcal{B}_n} d\epsilon \left\{ C_\delta^+(R_1(t,\epsilon)) + C_\delta^-(R_1(t,\epsilon)) \right\} \\
&= \left(\rho + \frac{S}{\sqrt{s_n}}\right) \int_{R^t(\mathcal{B}_n)} \frac{dR_1 dR_2}{J(R^t)} \left\{ C_\delta^+(R_1) + C_\delta^-(R_1) \right\} \\
&\leq \left(\rho + \frac{S}{\sqrt{s_n}}\right) \int_{R^t(\mathcal{B}_n)} dR_1 dR_2 \left\{ C_\delta^+(R_1) + C_\delta^-(R_1) \right\} \\
&\leq \left(\rho + \frac{S}{\sqrt{s_n}}\right) \int_{s_n}^{2s_n+\rho} dR_2 \Big[\Big(\widetilde{f}(2s_n + r_{\max} + \delta) - \widetilde{f}(2s_n + r_{\max} - \delta) \Big) \\
&\qquad\qquad + \Big(\widetilde{f}(s_n - \delta) - \widetilde{f}(s_n + \delta) \Big) \Big] . \qquad (239)
\end{aligned}$$

where we used that the Jacobian $J(R^t)$ of the $\mathcal{C}^1$ diffeomorphism $R^t : (\epsilon_1, \epsilon_2) \mapsto (R_1(t,\epsilon), R_2(t,\epsilon))$ is greater or equal to 1 (by regularity of the interpolation functions q and r) and $R^t(\mathcal{B}_n) \subset [s_n, 2s_n + r_{\max}] \times [s_n, 2s_n + \rho]$. The mean value theorem and (238) imply $|\widetilde{f}(R_1 - \delta) - \widetilde{f}(R_1 + \delta)| \leq \delta(\rho + S/\sqrt{s_n})$ uniformly in R_2. Therefore

$$\int_{\mathcal{B}_n} d\epsilon \left\{ C_\delta^+(R_1(t,\epsilon))^2 + C_\delta^-(R_1(t,\epsilon))^2 \right\} \leq 2\delta(s_n + \rho)\left(\rho + \frac{S}{\sqrt{s_n}}\right)^2 . \qquad (240)$$

Thus, integrating (237) over $\epsilon \in \mathcal{B}_n$ yields (using $\mathrm{Vol}(\mathcal{B}_n) = s_n^2$ and $s_n \leq 1/2$)

$$\begin{aligned}
&\int_{\mathcal{B}_n} d\epsilon\, \mathbb{E}\big[\big(\langle \mathcal{L} \rangle - \mathbb{E}[\langle \mathcal{L} \rangle) \big)^2 \big] \\
&\quad \leq 30\big(C + aS^2\sqrt{K+\delta}\big)\delta^{-2} s_n^2 n^{-1} + 20\delta(1/2 + \rho)\left(\rho + \frac{S}{\sqrt{s_n}}\right)^2 + 5S^2 a \frac{\ln 2}{2}\frac{s_n}{n} + \frac{b s_n^2}{n} .
\end{aligned}$$

Finally we choose $\delta = s_n n^{-1/4}$ and obtain the desired result. □

Proof of Lemma 31: Convexity implies that for any $\delta > 0$ we have

$$\begin{aligned}
G'(x) - g'(x) &\leq \frac{G(x+\delta) - G(x)}{\delta} - g'(x) \\
&\leq \frac{G(x+\delta) - G(x)}{\delta} - g'(x) + g'(x+\delta) - \frac{g(x+\delta) - g(x)}{\delta} \\
&= \frac{G(x+\delta) - g(x+\delta)}{\delta} - \frac{G(x) - g(x)}{\delta} + C_\delta^+(x) , \\
G'(x) - g'(x) &\geq \frac{G(x) - G(x-\delta)}{\delta} - g'(x) + g'(x-\delta) - \frac{g(x) - g(x-\delta)}{\delta} \\
&= \frac{G(x) - g(x)}{\delta} - \frac{G(x-\delta) - g(x-\delta)}{\delta} - C_\delta^-(x) .
\end{aligned}$$

Combining these two inequalities ends the proof. □

Appendix F: Details on numerics

Most of our experiments and codes are provided on the associated GitHub repository [26], with codes in the Julia programming language [63] (with a Jupyter notebook interface) and in matlab. In this appendix, we shall give additional details on how the plots have been obtained.

F.1 General purpose algorithms

We have been using free available softwares in our experiments: Standard machine learning tasks such as LASSO or logistic regression were done using scikit-learn [33]. Keras [34], with a tensorflow backend [64], was used for neural networks. We also used CVXPY, a python-embedded language for performing convex optimization [38], as well as PhaseMax for phase retrieval [39] experiments.

Figure 2 (in both the main text and SI) constrasts results of these general purpose algorithms with the optimal generalization error in three classification problems. In the left pannel of Fig. 2 (for the binary perceptron), we used logistic regression with hand-tuned ℓ_2 regularization (basically, we have hand-selected the regularization parameter in order to obtain the best results) with the function Logistic Regression in the software scikit-learn [33]. In the center pannel of Fig. 2 (this time for a sparse signal), we used the same software but this time with a sparsity enhancing ℓ_1 regularization, again fine-tuned by hand. In the right pannel of Fig. 2, we show, in the inset, how a neural network with 2 hidden layers was able to learn only approximately the "symmetric door" rule. In this experiment, we used Keras with a tensorflow backend.

The data are created with a signal-vector of dimension $n = 2500$ and are sent into a network made with a first layer of dimension 2500×64 followed by a rectified linear unit (ReLu), and a dropout layer with fraction 0.2 for regularization. This is followed by a second layer of dimension 64×64, this time with a sigmoid activation and again a dropout layer. Finally, we classify with a final output layer with a softmax, using the categorical cross-entropy as the loss function. The minimization is done using the RMSprop optimizer for 1000 epochs. The code is shown in the GitHub repository [26].

We have tried many variations around this network. Interestingly, the dropout layers have a strong effect on the regularization, and help significantly in improving the generalization error. Also interestingly, the number of epochs used for fitting was an important parameter. Indeed, the quality of the fit improves drastically as the number of epochs is increased: It seems that it actually takes a lot of time to escape the initial point, where prediction is just as bad as random. Finally, we also tried to increase the depth of the neural nets. Interestingly this did not affect the performance and the network was fitting the data and generalizing just as well with deeper and deeper networks. We believe that it should be very instructive to further study empirically this problem.

In Fig. 3 in this SI, simular plots are shown for three regression problems. In the left pannel, we used the LASSO function in the software scikit-learn [33] with its sparsity enhancing ℓ_1 regularization, again fine-tuned by hand.

For the middle pannel, we had to turn to a different software. In this case, the idea was to solve $\mathbf{Y} = \mathrm{Relu}(\mathbf{\Phi x}) = \max(0, \mathbf{\Phi x})$ (componentwise) subject to a sparse penalty on $\mathbf{x}$. Luckily, this can be turned into a linear programming framework: Minimize the ℓ_1 norm of $\mathbf{x}$ subject to the constraint that $\mathbf{Y} = \mathrm{Relu}(\mathbf{\Phi x})$ which can be implemented by enforcing $Y_\mu = \mathbf{\Phi}_\mu \cdot \mathbf{x}$ for μ's such that $Y_\mu > 0$, and $\mathbf{\Phi}_\mu \cdot \mathbf{x} < 0$ for μ's such that $Y_\mu = 0$. This linear program is solved with CVXPY [38] (CVX-2). We also show the results when only the indices μ associated with positive $Y_\mu > 0$ are used for comparaison (CVX-1). Finally, for the right figure, we used PhaseMax [39] out-of-the-box to solve the problem.

F.2 Evaluating the replica formula

In order to evaluate numerically the replica formula (22) we proceed as it is common in the statistical physics literature since the early papers on spin glasses. First, we found the critical points (23) by iterating the state evolution equations (48) —also called the replica self-consistent equations— starting from two different initial conditions ($q^{t=0} = 0$ and $q^{t=0} = \rho$). Next we computed the associated value of the free entropy and then selected, if two different fixed points were found, the correct one following the prescription given by Theorem 1. We also took special care in checking that we could not identify other fixed points. An example of such a procedure is shown in the GitHub repository [26], with codes in the Julia programming language [63], for

the perceptron problem.

F.3 Breaking the symmetry in GAMP

A last notable point concerns the symmetry issue in GAMP. Indeed, when $q = 0$ is a fixed point of the state evolution (which is the case if the prior has zero mean and the channel is symmetric), then GAMP should stay in this fixed point forever. This is the case, for instance, for two problems considered in the present paper: The symmetric door output function $\varphi(z) = \mathrm{sgn}(|z| - K)$ with a Rademacher prior ± 1, and for the sign-less channel $\varphi(z) = |z|$. In both cases, both z and $-z$ are giving the same output, and therefore so does both $\mathbf{X}^*$ and $-\mathbf{X}^*$. Notice, however, that this is not a problem in the computation of the free entropy. Here, one has to compute all the fixed points anyway. It is also not a problem if the prior is breaking the symmetry (for instance if one is working with a binary signal where $X_i^* = 1$ with probability $p_+ = 1/2 + \epsilon$ and $X_i^* = -1$ with probability $p_- = 1/2 - \epsilon$ with, say, $\epsilon = 10^{-7}$. In this case the symmetry is broken, GAMP works, and the state evolution predicts its behavior correctly. Even though this problem is thus restricted to a very small class of channels and priors, and even though perturbations solve it, it is still an interesting mathematical challenge, especially from the rigorous point of view. Indeed, this problem has attracted attention recently where initializations based on spectral algorithms were analyzed [24].

In the present paper, we adopted a pragmatic point of view. We did not break the symmetry in the data generative model (as that would make the problem slightly easier), instead we broke the symmetry in the GAMP solver, thus making it slightly but un-noticeably suboptimal. An example of our code is given in the associated GitHub repository [26]. We created the data in the symmetric manner, but when we ran GAMP to solve the problem, we broke the channel symmetry slightly. For instance, instead of solving with a door function that returns 1 only for $-0.674489 < z < 0.674489$, we use a function that instead returned 1 for $-0.674489 < z < 0.6745$. The same strategy was used for the absolute value function, which can be replaced by $\varphi(z) = z$ for $x > -\epsilon$ and $-z$ otherwise. Again, when ϵ is small, this makes only an un-noticeable difference in the figures. This trick allowed GAMP to solve symmetric problems without trouble in practice, and to reach perfect recovery even in the symmetric problems as close to the theoretical threshold as numerically desired.

References

[1] M. Mézard, G. Parisi, and M.-A. Virasoro. *Spin glass theory and beyond.* World Scientific Publishing, 1987.

[2] M. Mezard and A. Montanari. *Information, physics, and computation.* Oxford University Press, 2009.

[3] E. Gardner and B. Derrida. Three unfinished works on the optimal storage capacity of networks. *Journal of Physics A: Mathematical and General*, 22(12):1983, 1989.

[4] P. T. Boufounos and R. G. Baraniuk. 1-bit compressive sensing. In *42nd Annual Conference on Information Sciences and Systems (CISS)*, pages 16–21. IEEE, 2008.

[5] Y. Xu, Y. Kabashima, and L. Zdeborová. Bayesian signal reconstruction for 1-bit compressed sensing. *Journal of Statistical Mechanics: Theory and Experiment*, 2014(11):P11015, 2014.

[6] D. J. Thouless, P. W. Anderson, and R. G. Palmer. Solution of "solvable model of a spin glass". *Philosophical Magazine*, 35(3):593–601, 1977.

[7] M. Mézard. The space of interactions in neural networks: Gardner's computation with the cavity method. *Journal of Physics A: Mathematical and General*, 22(12):2181–2190, 1989.

[8] Y. Kabashima. Inference from correlated patterns: a unified theory for perceptron learning and linear vector channels. *Journal of Physics: Conference Series*, 95(1):012001, 2008.

[9] C. Baldassi, A. Braunstein, N. Brunel, and R. Zecchina. Efficient supervised learning in networks with binary synapses. *Proceedings of the National Academy of Sciences*, 104(26):11079–11084, 2007.

[10] T. Richardson and R. Urbanke. *Modern coding theory*. Cambridge university press, 2008.

[11] M. J. Wainwright, M. I. Jordan, et al. Graphical models, exponential families, and variational inference. *Foundations and Trends® in Machine Learning*, 1(1–2):1–305, 2008.

[12] D. L. Donoho, A. Maleki, and A. Montanari. Message-passing algorithms for compressed sensing. *Proceedings of the National Academy of Sciences*, 106(45):18914–18919, Nov 2009.

[13] F. Krzakala, M. Mézard, F. Sausset, Y. Sun, and L. Zdeborová. Statistical-physics-based reconstruction in compressed sensing. *Phys. Rev. X*, 2:021005(18), May 2012.

[14] J. P. Vila and P. Schniter. Expectation-maximization gaussian-mixture approximate message passing. *IEEE Transactions on Signal Processing*, 61(19):4658–4672, 2013.

[15] S. Rangan. Generalized approximate message passing for estimation with random linear mixing. In *IEEE International Symposium on Information Theory Proceedings (ISIT)*, pages 2168–2172, July 2011.

[16] J. Ziniel, P. Schniter, and P. Sederberg. Binary linear classification and feature selection via generalized approximate message passing. In *Information Sciences and Systems (CISS), 2014 48th Annual Conference on*, pages 1–6. IEEE, 2014.

[17] M. Bayati and A. Montanari. The dynamics of message passing on dense graphs, with applications to compressed sensing. *IEEE Transactions on Information Theory*, 57(2):764–785, Feb 2011.

[18] M. Bayati, M. Lelarge, and A. Montanari. Universality in polytope phase transitions and message passing algorithms. *The Annals of Applied Probability*, 25(2):753–822, 2015.

[19] A. Javanmard and A. Montanari. State evolution for general approximate message passing algorithms, with applications to spatial coupling. *Information and Inference: A Journal of the IMA*, 2(2):115–144, 2013.

[20] J. Barbier, M. Dia, N. Macris, and F. Krzakala. The mutual information in random linear estimation. In *54th Annual Allerton Conference on Communication, Control, and Computing*, pages 625–632. IEEE, 2016.

[21] J. Barbier, N. Macris, M. Dia, and F. Krzakala. Mutual information and optimality of approximate message-passing in random linear estimation. *arXiv:1701.05823*, 2017.

[22] G. Reeves and H. D. Pfister. The replica-symmetric prediction for compressed sensing with gaussian matrices is exact. In *IEEE International Symposium on Information Theory (ISIT)*, pages 665–669, 2016.

[23] L. Zdeborová and F. Krzakala. Statistical physics of inference: thresholds and algorithms. *Advances in Physics*, 65(5):453–552, 2016.

[24] M. Mondelli and A. Montanari. Fundamental limits of weak recovery with applications to phase retrieval. *arXiv preprint arXiv:1708.05932*, 2017.

[25] F. Krzakala, M. Mézard, F. Sausset, Y. Sun, and L. Zdeborová. Probabilistic reconstruction in compressed sensing: algorithms, phase diagrams, and threshold achieving matrices. *Journal of Statistical Mechanics: Theory and Experiment*, 2012(08):P08009(57), 2012.

[26] Github repository of numerical experiments for the current paper. `https://github.com/sphinxteam/GeneralizedLinearModel2017`, 2017.

[27] E. J. Candes and T. Tao. Near-optimal signal recovery from random projections: Universal encoding strategies? *IEEE Transactions on Information Theory*, 52(12):5406–5425, Dec 2006.

[28] D. Donoho and J. Tanner. Observed universality of phase transitions in high-dimensional geometry, with implications for modern data analysis and signal processing. *Philosophical Transactions of the Royal Society of London A: Mathematical, Physical and Engineering Sciences*, 367(1906):4273–4293, 2009.

[29] S. Oymak, A. Jalali, M. Fazel, Y. C. Eldar, and B. Hassibi. Simultaneously structured models with application to sparse and low-rank matrices. *IEEE Transactions on Information Theory*, 61(5):2886–2908, 2015.

[30] M. Soltanolkotabi. Structured signal recovery from quadratic measurements: Breaking sample complexity barriers via nonconvex optimization. *arXiv preprint arXiv:1702.06175*, 2017.

[31] D. Hansel, G. Mato, and C. Meunier. Memorization without generalization in a multilayered neural network. *EPL (Europhysics Letters)*, 20(5):471, 1992.

[32] T. Hosaka, Y. Kabashima, and H. Nishimori. Statistical mechanics of lossy data compression using a nonmonotonic perceptron. *Physical Review E*, 66(6):066126, 2002.

[33] F. Pedregosa, G. Varoquaux, A. Gramfort, V. Michel, B. Thirion, O. Grisel, M. Blondel, P. Prettenhofer, R. Weiss, V. Dubourg, J. Vanderplas, A. Passos, D. Cournapeau, M. Brucher, M. Perrot, and E. Duchesnay. Scikit-learn: Machine learning in Python. *Journal of Machine Learning Research*, 12:2825–2830, 2011.

[34] F. Chollet. keras. https://github.com/fchollet/keras, 2015.

[35] E. B. Baum and Y.-D. Lyuu. The transition to perfect generalization in perceptrons. *Neural computation*, 3(3):386–401, 1991.

[36] M. Opper and D. Haussler. Generalization performance of bayes optimal classification algorithm for learning a perceptron. *Physical Review Letters*, 66(20):2677, 1991.

[37] H. S. Seung, H. Sompolinsky, and N. Tishby. Statistical mechanics of learning from examples. *Phys. Rev. A*, 45:6056–6091, Apr 1992.

[38] S. Diamond and S. Boyd. CVXPY: A Python-embedded modeling language for convex optimization. *Journal of Machine Learning Research*, 2016. To appear.

[39] T. Goldstein and C. Studer. Phasemax: Convex phase retrieval via basis pursuit. *arXiv preprint arXiv:1610.07531*, 2016.

[40] Y. LeCun, Y. Bengio, and G. Hinton. Deep learning. *Nature*, 521(7553):436–444, 2015.

[41] J. Barbier and N. Macris. The adaptive interpolation method: a simple scheme to prove replica formulas in bayesian inference. *Probability Theory and Related Fields*, Oct 2018.

[42] F. Guerra and F. L. Toninelli. The thermodynamic limit in mean field spin glass models. *Communications in Mathematical Physics*, 230(1):71–79, 2002.

[43] J. Barbier, N. Macris, and L. Miolane. The Layered Structure of Tensor Estimation and its Mutual Information. In *47th Annual Allerton Conference on Communication, Control, and Computing (Allerton)*, September 2017.

[44] M. Talagrand. *Mean field models for spin glasses: Volume I: Basic examples*, volume 54. Springer Science & Business Media, 2010.

[45] A. Montanari. Estimating random variables from random sparse observations. *European Transactions on Telecommunications*, 19(4):385–403, 2008.

[46] S. B. Korada and N. Macris. Exact solution of the gauge symmetric p-spin glass model on a complete graph. *Journal of Statistical Physics*, 136(2):205–230, 2009.

[47] Y. Wu and S. Verdú. Optimal phase transitions in compressed sensing. *IEEE Transactions on Information Theory*, 58(10):6241–6263, Oct 2012.

[48] A. Coja-Oghlan, F. Krzakala, W. Perkins, and L. Zdeborova. Information-theoretic thresholds from the cavity method. In *Proceedings of the 49th Annual ACM SIGACT Symposium on Theory of Computing (STOC)*, pages 146–157, 2017.

[49] N. Macris. Griffith-kelly-sherman correlation inequalities: A useful tool in the theory of error correcting codes. *IEEE Transactions on Information Theory*, 53(2):664–683, Feb 2007.

[50] S. B. Korada and N. Macris. Tight bounds on the capacity of binary input random cdma systems. *IEEE Transactions on Information Theory*, 56(11):5590–5613, Nov 2010.

[51] P. Hartman. *Ordinary Differential Equations*. Society for Industrial and Applied Mathematics, 2002, 1964.

[52] P. Milgrom and I. Segal. Envelope theorems for arbitrary choice sets. *Econometrica*, 70(2):583–601, 2002.

[53] D. Guo, Y. Wu, S. S. Shitz, and S. Verdú. Estimation in gaussian noise: Properties of the minimum mean-square error. *IEEE Transactions on Information Theory*, 57(4):2371–2385, 2011.

[54] S. B. Korada and A. Montanari. Applications of the Lindeberg principle in communications and statistical learning. *IEEE Transactions on Information Theory*, 57(4):2440–2450, 2011.

[55] D. Guo, S. Shamai, and S. Verdú. Mutual information and minimum mean-square error in gaussian channels. *IEEE Transactions on Information Theory*, 51(4):1261–1282, April 2005.

[56] Y. Wu and S. Verdú. Functional properties of minimum mean-square error and mutual information. *IEEE Transactions on Information Theory*, 58(3):1289–1301, 2012.

[57] L. Miolane. Phase transitions in spiked matrix estimation: information-theoretic analysis. *arXiv preprint arXiv:1806.04343*, 2018.

[58] V. I. Bogachev. *Measure theory*, volume 1. Springer Science & Business Media, 2007.

[59] M. Lelarge and L. Miolane. Fundamental limits of symmetric low-rank matrix estimation. *ArXiv e-prints*, November 2016.

[60] S. Chatterjee et al. A generalization of the lindeberg principle. *The Annals of Probability*, 34(6):2061–2076, 2006.

[61] R. T. Rockafellar. *Convex analysis*. Princeton university press, 2015.

[62] S. Boucheron, G. Lugosi, and O. Bousquet. Concentration inequalities. In *Advanced Lectures on Machine Learning*, pages 208–240. Springer, 2004.

[63] J. Bezanzon, S. Karpinski, V. Shah, and A. Edelman. Julia: A fast dynamic language for technical computing. In *Lang.NEXT*, April 2012.

[64] M. Abadi, A. Agarwal, P. Barham, E. Brevdo, Z. Chen, C. Citro, G. S. Corrado, A. Davis, J. Dean, M. Devin, S. Ghemawat, I. Goodfellow, A. Harp, G. Irving, M. Isard, Y. Jia, R. Jozefowicz, L. Kaiser, M. Kudlur, J. Levenberg, D. Mané, R. Monga, S. Moore, D. Murray, C. Olah, M. Schuster, J. Shlens, B. Steiner, I. Sutskever, K. Talwar, P. Tucker, V. Vanhoucke, V. Vasudevan, F. Viégas, O. Vinyals, P. Warden, M. Wattenberg, M. Wicke, Y. Yu, and X. Zheng. TensorFlow: Large-scale machine learning on heterogeneous systems, 2015. Software available from tensorflow.org.
